# Mining genes, genomic selection and simulation breeding on hundred-seed weight using a four-way RIL population

**DOI:** 10.3389/fpls.2026.1812087

**Published:** 2026-04-23

**Authors:** Shuchao Liu, Xianmin Lu, Jiufeng Zhao, Fulin Shan, Zhiyuan Yu, Xu Sun, Guizheng Wang, Haojie Xie, Yanming Zhou, Bo Hu, Wen-Xia Li, Hailong Ning

**Affiliations:** 1 Key Laboratory of Soybean Biology, Ministry of Education, Key Laboratory of Soybean Biology and Breeding/Genetics, Ministry of Agriculture, Northeast Agricultural University, Harbin, Heilongjiang, China; 2 Zhongnongfa Wudalianchi Agricultural Technology Co., Ltd., Wudalianchi, Heilongjiang, China

**Keywords:** genomic selection, QTL, QTN, simulation breeding, soybean hundred-seed weight

## Abstract

Hundred-seed weight (HSW) is a critical determinant of soybean yield potential, yet its genetic dissection is often limited by the restricted allelic diversity of traditional biparental populations. In this study, we developed a four-way recombinant inbred line (FW-RIL) population comprising 144 lines derived from four founders with distinct HSW phenotypes to enhance mapping resolution and allelic richness. By integrating a high-density genetic linkage map with five multi-locus genome-wide association study (GWAS) models, we systemically analyzed the genetic basis of HSW across multiple environments. A total of 16 quantitative trait loci (QTLs) and 40 quantitative trait nucleotides (QTNs) were identified, among which three QTLs and two QTNs were consistently detected across environments and analytical approaches. Co-localization analysis, combined with linkage disequilibrium (LD) mapping, haplotype effect evaluation, and gene expression profiling, prioritized *Glyma.02G047200* as the key candidate gene within the stable major-effect locus *qHSW-2-1*. This gene encodes an oligopeptide transporter, and haplotype analyses demonstrated that elite alleles significantly increase seed weight across diverse genetic backgrounds. Furthermore, comparative evaluation of multiple genomic selection (GS) models revealed that the Light Gradient Boosting Machine (LightGBM) achieved the highest predictive accuracy for HSW. Using this model, breeding simulations identified several high-yielding hybrid combinations pyramiding multiple superior allelic variants. Overall, this study elucidates the genetic determinants of soybean HSW and highlights the effectiveness of integrating multi-parent populations, genomic selection, and molecular design breeding to accelerate yield improvement.

## 1 Introduction

Soybean (*Glycine max* L.) is the world’s leading source of vegetable oil and plant-based protein. Soybean yield is a complex quantitative trait governed by various factors, among which seed number per plant and seed weight are the primary determinants (Tian et al., 2025). As a key indicator of seed weight, hundred-seed weight (HSW) directly reflects the efficiency and extent of grain filling, playing a crucial role in enhancing soybean yield potential (Hu et al., 2023). Therefore, elucidating the genetic architecture underlying HSW is essential for the targeted improvement of soybean yield through modern molecular breeding approaches.

HSW is a typical polygenic trait characterized by complex inheritance patterns and strong environmental sensitivity. To date, the SoyBase database has reported 310 quantitative trait loci (QTLs) associated with HSW, distributed across all 20 soybean chromosomes, providing a substantial foundation for genetic improvement. An integrative analysis combining linkage mapping and genome-wide association studies (GWAS) has identified multiple genes critical for seed development. For example, the major-effect QTL *qSw17–1* harbors *GmKIX8-1*, a key regulator of organ size (Nguyen et al., 2021). Likewise, the *Dt1* locus within the *SW19* region interacts with *GmSWEET10a*, thereby negatively regulating sucrose transport to the embryo under long-day conditions (Li et al., 2024a). In addition, GWAS conducted in natural populations have identified genes such as *GmSW17*, *SW14*, and *POWR1*, shedding light on the roles of cell proliferation and domestication-related selection in seed weight regulation (Goettel et al., 2022; Liang et al., 2024; Zhang et al., 2025a). Notably, the application of multi-parent populations, including Recombinant Inbred Lines (RILs), in GWAS can effectively reduce population structure bias and improve the detection power for rare alleles (Luo et al., 2023; Xu et al., 2023). Taking this concept further, Multi-parent Advanced Generation Inter-Cross (MAGIC) populations have recently been developed as powerful platforms in soybean genetics. By intercrossing multiple diverse founders, MAGIC populations significantly expand allelic richness and facilitate high-resolution mapping of complex traits (Shivakumar et al., 2018; Hashemi et al., 2022). While MAGIC populations offer unprecedented genetic diversity, four-way recombinant inbred line (FW-RIL) populations present a highly efficient and practical alternative that captures multi-founder diversity with a more streamlined crossing scheme. Despite these advances, most reported QTLs or genes exhibit limited allelic diversity and poor stability across environments or genetic backgrounds (Elattar et al., 2021; Huang et al., 2021; Yamaguchi et al., 2021; Ayalew et al., 2022; Kumar et al., 2023; Wei et al., 2023; Gao et al., 2024; Li et al., 2024b; Zhang et al., 2024; Bhat et al., 2025; Chen et al., 2025; Patel et al., 2025; Luo et al., 2026), which restricts their practical application in soybean breeding programs.

Genomic Selection (GS) has emerged as a powerful breeding strategy that uses genome-wide molecular markers to predict genomic estimated breeding values (GEBVs). This approach enables accurate selection in early generations and substantially accelerates breeding cycles (Chen et al., 2023; Yoon et al., 2025). For highly heritable polygenic traits, such as HSW, GS often outperforms traditional marker-assisted selection (MAS) in predictive accuracy. Consequently, the development of robust and high-precision GS models has become a key objective for accelerating the breeding of high-yielding and stable soybean cultivars (Belzile et al., 2022).

To enhance the reliability and breeding relevance of locus identification, we developed a four-way recombinant inbred line (FW-RIL) population consisting of 144 lines derived from founders with distinct HSW phenotypes. By integrating a high-density genetic linkage map with five multi-locus GWAS models, we dissected the genetic basis of HSW across multiple environments and identified stable loci associated with this trait. Haplotype analysis and candidate gene prediction were subsequently conducted for these stable loci, followed by validation in a natural population. In addition, we evaluated the efficacy of various GS models for HSW prediction and parental selection. This study aims to elucidate novel genetic resources controlling soybean seed weight and to propose elite parental combinations and actionable strategies for molecular design breeding.

## 2 Materials and methods

### 2.1 Genetic population

A four-way recombinant inbred line (FW-RIL) population was developed from four soybean cultivars exhibiting significant phenotypic divergence in hundred-seed weight (HSW): Kenfeng 14 (21 g), Kenfeng 15 (18 g), Kenfeng 19 (19 g), and Heinong 48 (25 g). In 2008, two independent single crosses: Kenfeng 14 × Kenfeng 15 and Kenfeng 19 × Heinong 48, were generated. In 2009, F_1_ plants from these crosses were intercrossed to produce double-cross F_1_ seeds [(Kenfeng 14 × Kenfeng 15) × (Kenfeng 19 × Heinong 48)]. In 2010, a total of 204 seeds were harvested from the self-pollinated double-cross F1 plants.

From the subsequent generation onward, the single-seed descent (SSD) method was applied for seven consecutive generations of selfing. Ultimately, a stable FW-RIL population at the F_8_ generation consisting of 144 homozygous lines was obtained. This multi-parent population served as the experimental platform for high-resolution QTL mapping and genome-wide association studies (GWAS).

### 2.2 Field trials and phenotypic evaluation

From 2013 to 2020, the FW-RIL population and its four founding parents were evaluated at two experimental sites in Heilongjiang Province, China: Xiangyang (126.73°E, 45.75°N) and Acheng (126.95°E, 45.52°N). By varying sowing dates and planting densities, four distinct environments were established for phenotypic evaluation (**Table 1**). All trials were conducted using a randomized complete block design (RCBD) with three biological replicates. Each plot consisted of rows 3.0 m in length, with an inter-row spacing of 0.7 m and an intra-row spacing of 0.07 m. Standard local agronomic practices for soybean cultivation were applied uniformly across all environments.

**Table 1:** Details of planting conditions in field experiments.

Environment	Location	Experimental treatment		
		Density (×10^5^plant/hm^2^)	N/P_2_O_5_/K_2_O Fertilizer(kg/hm^2^)	Sowing date
E1	Xiangyang	28	18/46/30	2015.05.10
E2	Xiangyang	22	18/46/30	2015.05.10
E3	Acheng	22	18/46/30	2016.5.12
E4	Acheng	28	18/46/30	2016.5.12

At physiological maturity, five representative plants were randomly sampled from the interior rows of each plot to eliminate edge effects. Hundred-seed weight (HSW) was measured using a high-precision electronic balance (0.01 g accuracy; Wantai, Changzhou, China). The mean HSW value of the five sampled plants per plot was used for subsequent statistical and genetic analyses.

### 2.3 Statistical analysis of phenotypic data and heritability estimation

Descriptive statistical analyses of HSW across the four environments were performed using SAS version 9.2 (SAS Institute, Cary, NC, USA). Parameters including the mean, coefficient of variation (CV), skewness, and kurtosis were calculated, and the phenotypic distribution was assessed for normality. Analysis of variance (ANOVA) was conducted under both single-environment and multi-environment models.

For multi-environment analysis, a mixed linear model was applied to estimate the effects of genotype (G), environment (E), and genotype × environment (G × E) interaction. The multi-environment model is formulated as: Yijk=μ+Gi+Ej+(GE)ij+Rk(j)+ϵijk, where Yijk is the phenotypic observation of the *i*th genotype in the *j*th environment within the *k*th replicate; μ is the overall grand mean; Gi is the effect of the *i*th genotype; Ej is the effect of the *j*th environment; (GE)ij is the genotype-by-environment interaction effect; Rk(j) is the effect of the *k*th replicate nested within the *j*th environment; and ϵijk is the random residual error. In the model, Genotype and G × E interaction were treated as random effects to estimate variance components. Broad-sense heritability $h^{2}$ across multiple environments was estimated on a genotypic mean basis using the following formula: Genotype and G×E interaction were treated as random effects to estimate genotypic variance ( ${\text{σG}}_{2}$), G×E interaction variance ( ${\text{σ}}_{\text{GE}}^{2}$), and error variance ( ${\text{σϵ}}_{2}$). Broad-sense heritability (h^2^) across multiple environments was estimated using the following formula (Piepho and Moehring, 2007; Schmidt et al., 2019).


$$h^{2}=\frac{\sigma_{G}^{2}}{\sigma_{G}^{2}+\frac{\sigma_{GE}^{2}}{e}+\frac{\sigma_{\epsilon }^{2}}{re}}$$

Where. ${\text{σG}}_{2}$ is genotype variance, ${\text{σ}}_{\text{GE}}^{2}$ is genotype×environment interaction effect variance,

For single-environment analysis, the model $Yij=\mu +G_{i}+R_{j}+\epsilon ij$ was used, with genotype treated as a random effect. Broad-sense heritability within a single environment was calculated as:


$$h^{2}=\frac{\sigma_{G}^{2}}{\sigma_{G}^{2}+\sigma_{\epsilon }^{2}}$$

### 2.4 QTL mapping strategy

QTL analysis was performed using a previously constructed high-density genetic linkage map. This map was developed by our research group using the exact same FW-RIL population (Wang et al., 2022). The mapping analysis was executed using the GAPL v1.2 software. Two complementary methods, Interval Mapping (IM-ADD) and Inclusive Composite Interval Mapping (ICIM-ADD), were employed to ensure robust QTL detection. Genome-wide scanning was performed with a step size of 1 cM, and a logarithm of odds (LOD) threshold of 3.0 was used to declare significant QTLs. For ICIM-ADD, the probability of inclusion (PIN) for background markers was set to 0.001. QTL nomenclature followed the convention proposed by McCough and Doerge. (1995), using the format *qHSW-Chr-No*. (e.g., *qHSW-1-1*), where “*q*” indicates a QTL, “*HSW*” represents hundred-seed weight, “*Chr*” denotes the chromosome number, and “*No.*” refers to the serial number of the QTL on that chromosome.

### 2.5 Genome-wide association study

A total of 109,676 high-quality single-nucleotide polymorphism (SNP) markers previously developed from the SoySNP660K chip were used for GWAS and linkage map construction. Detailed protocols for genomic DNA extraction, SNP genotyping via the SoySNP660K array, and quality control for this specific FW-RIL population have been described in our previous study (Li et al., 2020). Based on the linkage disequilibrium (LD) decay distance of the FW-RIL population (Zhang et al., 2025b), candidate gene search regions were defined as 100 kb upstream and downstream of each significant quantitative trait nucleotide (QTN).

GWAS analyses across four environments were conducted using the R package mrMLM.GUI, incorporating five multi-locus models: mrMLM, FASTmrMLM, FASTmrEMMA, pLARmEB, and ISIS EM-BLASSO (Wang et al., 2016; Tamba et al., 2017; Zhang et al., 2017; Wen et al., 2018). The critical P-value was set to 0.005 for FASTmrEMMA and 0.01 for the remaining four models, with LOD_mrMLM.GUI_≥3.0 used as the threshold for significant QTNs. Association results were visualized using TBtools-II (v2.119).

### 2.6 Candidate gene prediction and functional prioritization

Stable genetic loci consistently detected across multiple environments or analytical methods were selected for candidate gene analysis. Based on the LD decay distance (200 kb), a 100 kb window upstream and downstream of each peak SNP or marker was defined as the candidate region. Gene annotations within these regions were retrieved from the Phytozome database.

Transcriptomic data (RNA-seq) from the SoyOmics database were analyzed to identify genes highly expressed during seed development. Furthermore, resequencing data from the FW-RIL parental lines were analyzed to identify sequence polymorphisms in the coding sequence (CDS) and promoter region (3 kb upstream) of candidate genes. Particular attention was given to non-synonymous SNPs and variants affecting cis-regulatory elements to prioritize key candidate genes.

### 2.7 Haplotype analysis and validation in natural populations

Haplotype analysis was conducted in the FW-RIL population to assess the effects of candidate genes on HSW. Based on sequence variation within exon regions, the 144 FW-RIL lines were classified into distinct haplotypes. Differences in HSW among haplotypes were evaluated using ANOVA.

To further validate candidate gene effects in broader genetic backgrounds, haplotype analyses were performed in a natural soybean population (GPP) comprising 2,883 accessions obtained from the SoyOmics database. Resequencing data and corresponding HSW phenotypes were used to evaluate the consistency and superiority of haplotypes across diverse germplasm.

### 2.8 Evaluation of selection signatures and allelic distribution

The distribution of candidate genes in soybean germplasm at different improvement stages was analyzed using the GPP natural population, which included 102 wild soybeans (*Glycine soja*), 1,048 landraces, and 1,733 modern cultivars. The frequency distribution of candidate haplotypes across these three subpopulations was calculated and compared. Pie charts illustrating allele frequency distributions were generated using R to assess selection footprints during soybean domestication and improvement.

### 2.9 Development and evaluation of genomic selection models

Thirteen genomic selection (GS) models were evaluated for HSW prediction, including seven parametric models (GBLUP, rrBLUP, BayesA, BayesB, BayesC, Bayesian LASSO, and Bayesian Ridge Regression (BRR)) and six non-parametric or machine learning models (Support Vector Regression (SVR), Random Forest (RF), Light Gradient Boosting Machine (LightGBM), eXtreme Gradient Boosting (XGBoost), Support Vector Machine (SVM), and Kernel Ridge Regression (KRR)).

For machine learning models, hyperparameters were optimized using Bayesian optimization within a five-fold cross-validation framework to ensure model robustness (Breiman, 2001; Meuwissen et al., 2009; Calus et al., 2011; Endelman, 2011; de los Campos et al., 2013; Yunting et al., 2023). To reduce computational complexity, redundant SNPs were removed based on the LD decay distance. The final feature set included lead QTNs identified by GWAS and SNPs in high LD regions, with Best Linear Unbiased Prediction (BLUP) values of HSW used as response variables. Model performance was evaluated using 10-fold cross-validation, repeated 100 times. Predictive efficacy was benchmarked with five metrics: Pearson correlation coefficient (Pearson), Root Mean Square Error (RMSE), Mean Absolute Error (MAE), Coefficient of Determination (R^2^), and Bias. The finalized models were further validated using an independent population.

For non-parametric models, feature importance was interpreted using SHapley Additive exPlanations (SHAP) method, which quantified the contribution and directionality of individual markers. All analyses were performed in R using packages including rrBLUP, BGLR, xgboost, gbm, lightgbm, randomForest, e1071, and catboost. Data visualization was conducted using the ggplot2, ggthemes, viridis, and RColorBrewer packages.

### 2.10 Breeding simulation and cross prediction

For each significant QTN, SNP genotypes were coded as -1 and 1, and the results from the best-performing GS model were applied. Superior alleles were defined based on QTN effect estimates; when the effect value was positive, the genotype coded as 1 was considered the superior allele. To enhance HSW, hybrid combinations pyramiding a greater number of superior alleles were screened.

**Figure 1:**
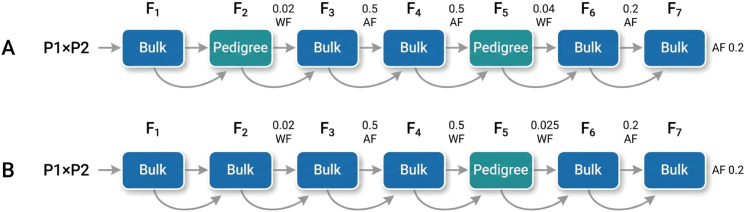
Flowchart of simulated breeding schemes using single-cross combinations **(A, B)** with the progeny pedigree method and bulk selection method. Pedigree represents a single harvest of selected plants within a family, and bulk represents a mixed harvest of selected plants within a family. Ten plants were selected for each F_1_ hybrid; 200, 500, or 800 plants for each F_2_ line; 30 plants for each F_3_ and F_4_ line; and 50 plants for each F_5_–F_7_ line. AF represents interfamily selection, WF represents intrafamily selection, and decimals represent selection proportions. Families with the greatest phenotype were selected according to a given proportion in each generation.

Following a semi-diallel crossing design, all 144 parental lines were paired to generate single-cross combinations. Modified pedigree (Ped) and bulk selection (Blk) methods were applied following hybridization. Breeding simulations were implemented using the B4L function of ISB software (Sun et al., 2025), and the overall workflow is illustrated in **Figure 1**.

## 3 Results

### 3.1 Phenotypic variation and genetic basis of HSW in the FW-RIL population

Phenotypic statistics and ANOVA for HSW across four environments are summarized in **Table 2** and **Supplementary Table 1**. Significant variation was observed among FW-RIL lines, with genotype effects reaching statistical significance. Kurtosis and skewness indicated that HSW was approximately normally distributed in each environment (**Supplementary Figure 1**), consistent with polygenic control. Parental HSW values fell within the FW-RIL range, indicating transgressive segregation. Environmental differences were pronounced, reflected in variations in mean, standard deviation, and kurtosis, and the genotype × environment (G × E) interactions were highly significant. Broad-sense heritability ranged from 49% to 79%, highlighting substantial environmental influence on the expression of HSW-related genes (**Table 3**).

**Table 2:** Joint ANOVA and heritability of HSW in 4 environments.

Source^a^	DF^b^	SS^c^	MS^d^	F^e^	Pr >F^f^	Variance components^g^
Env	3	1488.14	496.05	26.89	<.0001	1.81	
Block(Env)	8	24.34	3.04	0.16	1.0000		
Gen	142	8092.72	56.99	3.09	<.0001	4.02	
Env*Gen	318	5866.16	18.45	4.73	<.0001	4.84	
Error	920	3586.07	3.90			3.89	
Total	1391	19454.23					
h^2^						0.72	

a Sources of variation partitioned based on the Linear Mixed Model (LMM). Env, environment (4 locations/years); Block(Env), block (replicate) effect nested within environments; Gen, genotype; EnvGen, genotype-by-environment interaction effect; Error, residual variation unexplained by the model; Total, total variation of all observations; h^2^, generalized heritability (broad-sense heritability).

b Degrees of freedom (df) representing the independent information for each source of variation. The df for Env*Gen (318) is lower than the theoretical value due to an unbalanced dataset caused by missing observations.

c Sum of Squares (SS) of deviations for each source of variation.

d Mean Square (MS), providing unbiased estimates of variance.

e F-value, the statistic used to test the significance of each effect. Under the random effects model, the F-values for the main effects of Env and Gen were calculated using the MS of Env*Gen as the denominator.

f P-value, the probability of observing the current F-value or one more extreme.

g Variance components for each random effect estimated via the Expected Mean Square (EMS) method (i.e., ${\text{σG}}_{2}$, ${\text{σ}}_{\text{GE}}^{2}$, and ${\text{σϵ}}_{2}$).

**Table 3:** Estimates of genetic variation and heritability across different environments.

Environment	DF_G_^a^	DF_e_^b^	MS_G_^c^	MS_e_^d^	F^e^	Pr>F^f^	V_G_^g^	V_E_^h^	h^2^^i^
E1	129	258	41.73	4.44	9.39	<0.0001	12.43	4.45	0.74
E2	129	258	42.37	3.48	12.16	<0.0001	12.96	3.48	0.79
E3	120	240	15.31	3.91	3.92	<0.0001	3.80	3.90	0.49
E4	82	164	15.51	3.68	4.21	<0.0001	3.94	3.68	0.52

a DF_G_, degrees of freedom for genotype.

b DF_e_, degrees of freedom for residual error.

c MS_G_, mean square for genotype.

d MS_e_, mean square for residual error.

e F, F-statistic, used to test the significance of genotypic differences.

f Pr>F, the probability associated with the F-statistic.

g V_G_, genotypic variance component, representing the phenotypic variation attributed to genetic differences.

h V_E_, residual variance component, encompassing phenotypic variation caused by genotype-by-environment (G × E) interactions and random errors.

i h^2^, broad-sense heritability, representing the proportion of the total phenotypic variation that is attributable to genetic effects.

### 3.2 Identification of QTLs associated with soybean HSW

Across the four environments (E1-E4), 16 QTLs were identified as significantly associated with HSW (**Table 4**). Eight QTLs were detected by multiple mapping methods, and two were consistently expressed across multiple environments. Notably, one QTL satisfied both multiple-method and multiple-environment stability criteria. The number of QTLs varied across environments: six each in E3 and E4, and three each in E1 and E2. The 16 QTLs were distributed across eight chromosomes, with chromosomes 17 and 18 each harboring three QTLs. LOD values ranged from 3.06 to 4.47, and phenotypic variation explained (PVE) ranged from 6.21% to 14.59%. Six QTLs had a contribution > 10% and were defined as major QTLs, representing key loci for HSW enhancement (**Supplementary Figure 2**; **Supplementary Table 2**).

**Table 4:** QTLs associated with HSW identified in the FW-RIL population across four environments.

Name	Chr	Method	Environment	Left position (bp)	Right position (bp)	LOD^a^	PVE (%) b
*qHSW-2-1*	2	IM&ICIM	E3	5560717	3952555	3.2951-3.8556	6.6948-14.5906
*qHSW-8-1*	8	IM	E3	4528693	4265916	3.2261	6.7887
*qHSW-9-1*	9	IM&ICIM	E4	1206616	32032649	4.4674	12.3194
*qHSW-9-2*	9	IM&ICIM	E4	6256462	5866504	3.8642	8.2294
*qHSW-12-1*	12	IM&ICIM	E1&E2	35990600	36275137	3.1848-4.3844	10.8302-13.4527
*qHSW-12-2*	12	IM&ICIM	E4	16069251	13868525	3.3131	7.5335
*qHSW-15-1*	15	IM	E3	50600618	48703253	3.5944	9.4347
*qHSW-15-2*	15	IM	E3	15781408	15129437	3.9193	8.8058
*qHSW-17-1*	17	IM	E1	34983373	37050676	3.7279	13.0765
*qHSW-17-2*	17	ICIM	E1&E2	36850271	36557987	4.0372-4.3057	11.4503-12.7187
*qHSW-17-3*	17	ICIM	E2	10433088	7565828	3.0587	7.8931
*qHSW-18-1*	18	IM&ICIM	E4	2759157	10333228	3.0867	6.2146
*qHSW-18-2*	18	IM	E3	55369435	55472624	3.287	6.9354
*qHSW-18-3*	18	IM&ICIM	E4	1553751	1764099	3.4299	6.9375
*qHSW-19-1*	19	IM&ICIM	E4	30930458	35961145	3.1766	12.2647
*qHSW-19-2*	19	IM	E3	50284807	38866655	3.6974	7.4152

a Logarithm of the Odds score. It indicates the strength of statistical evidence for the presence of a QTL at the given genomic location.

b The percentage of Phenotypic Variance Explained by the identified QTL. It represents the magnitude of the effect of the locus on the HSW trait.

### 3.3 Genome-wide identification of QTNs associated with soybean HSW

**Figure 2:**
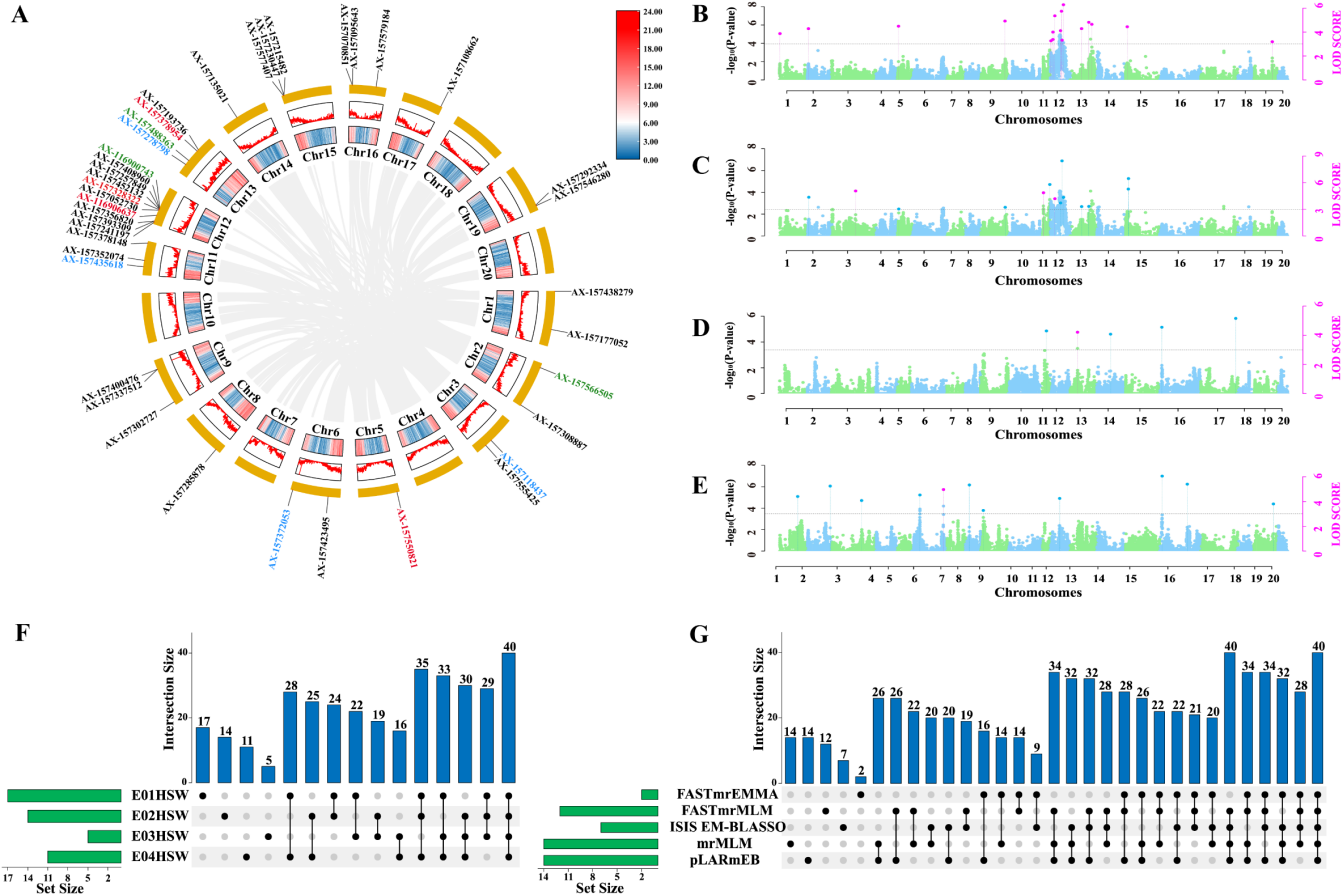
GWAS analysis for HSW in Soybean FW-RIL population. **(A)** Distribution of QTNs associated with soybean HSW across 20 chromosomes. The black markings indicate QTNs detected by a single method in a single environment; the blue markings indicate QTNs detected by multiple methods in a single environment; the green markings indicate QTNs detected by a single method across multiple environments; and the red markings indicate QTNs detected by multiple methods across multiple environments. **(B)** HSW QTN Manhattan diagram of soybean under environment E1. **(C)** HSW QTN Manhattan diagram of soybean under environment E2. **(D)** HSW QTN Manhattan diagram of soybean under environment E3. **(E)** HSW QTN Manhattan diagram of soybean under environment E4. **(F)** Upset diagram representing the number of HSW QTN identified by the five GWAS methods. **(G)** UpSet diagram representing the number of HSW QTNs identified in the four environments.

Using five multi-locus GWAS models, 40 QTNs significantly associated with HSW were identified genome-wide (**Supplementary Table 3**). Individual QTNs explained 4.34% to 35.84% of the phenotypic variation (PVE). The distribution across methods was as follows: mrMLM (15 QTNs), FASTmrMLM (12), pLARmEB (16), FASTmrEMMA (2), and ISIS EM-BLASSO (8). Among these, eight QTNs were detected by multiple methods, and seven were stable across environments. Four QTNs satisfied both multi-environment stability and multi-method co-detection criteria (**Figure 2**), with LOD values ranging from 4.12 to 6.46 and PVE values from 8.12% to 35.84%, indicating that they represent major genetic loci influencing seed weight.

### 3.4 Candidate gene identification and functional prioritization for HSW

**Figure 3:**
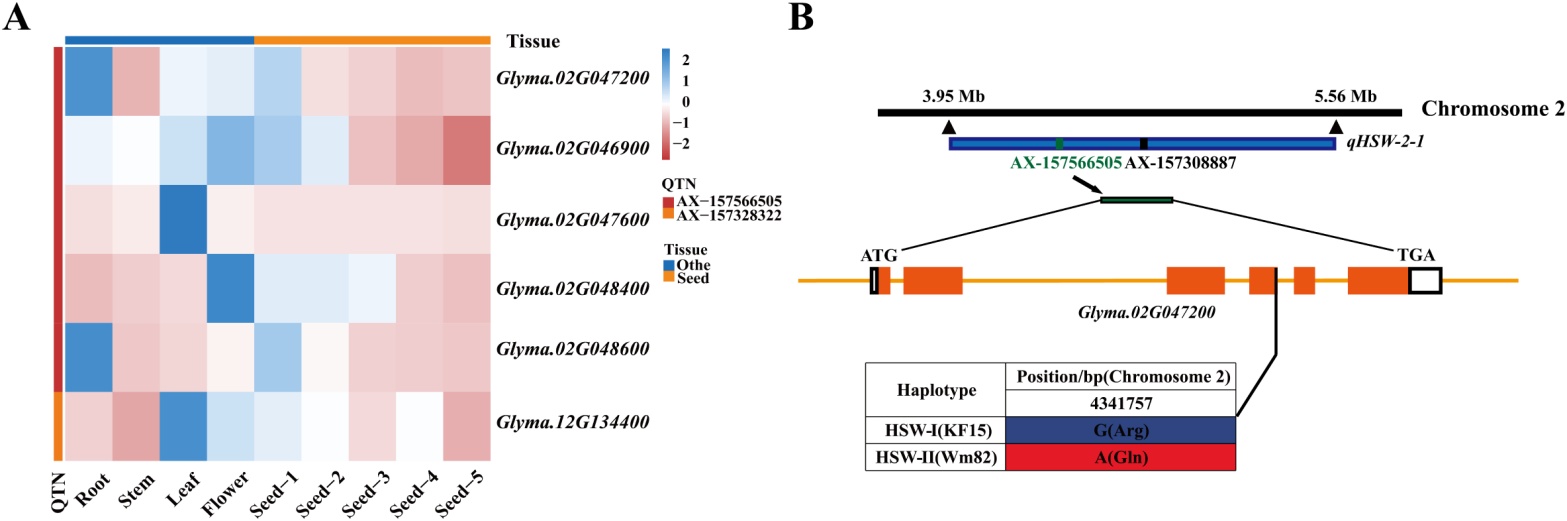
Screening and identification of candidate genes for soybean HSW. **(A)** Expression levels of 6 candidate genes in roots, stems, leaves, flowers, and seeds. **(B)** Gene sequence variation diagram.

Co-localization of the 40 identified QTNs with 16 QTLs showed that six QTNs were located within four QTL intervals (**Supplementary Figure 3**). In high-confidence regions repeatedly detected across multiple environments or methods, two core QTNs, AX-157566505 and AX-157328322, co-localized with stable major QTLs *qHSW-2–1* and *qHSW-12-2*, respectively. Using a 200 kb LD decay window, 38 candidate genes were identified within 100 kb upstream and downstream of these core QTNs (**Supplementary Table 4**). Functional annotation in Phytozome highlighted six genes potentially related to HSW. RNA-seq analysis from the SoyOmics database revealed that four genes (*Glyma.02G047200*, *Glyma.02G046900*, *Glyma.02G048400*, and *Glyma.02G048600*) exhibited seed-specific high expression (**Figure 3A**). Sequence variation analysis among parental lines showed that only *Glyma.02G047200* harbored variations in the coding sequence (CDS) (**Figure 3B**), identifying it as the key candidate gene regulating HSW in soybean.

### 3.5 Haplotype analysis and functional validation of *Glyma.02G047200*

**Figure 4:**
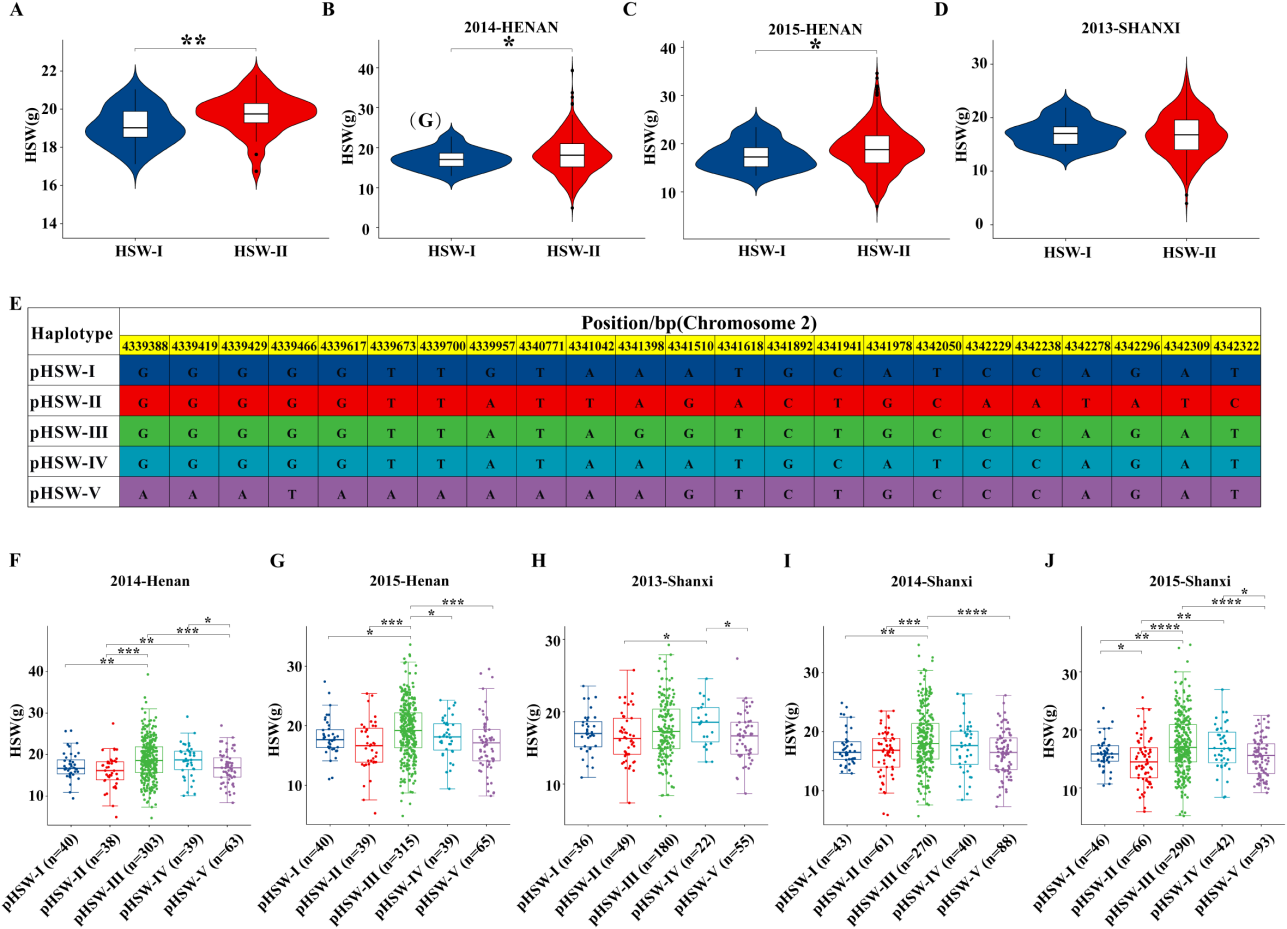
Haplotype analysis of the candidate gene. **(A)** Haplotype validation in the FW-RIL population (P = 0.0043). **(B)** Haplotype validation of the candidate gene in the GPP population under e2 (P = 0.044). **(C)** Haplotype validation of the candidate gene in the GPP population under e3 (P = 0.042). **(D)** Haplotype validation of the candidate gene in the GPP population under e6 (P = 0.061). **(E)** Five haplotypes identified in the promoter region of the candidate gene *Glyma.02G047200.* **(F)** Haplotype validation of the candidate gene promoter region in the GPP population under e2. **(G)** Haplotype validation of the candidate gene promoter region in the GPP population under e3. **(H)** Haplotype validation of the candidate gene promoter region in the GPP population under e6. **(I)** Haplotype validation of the candidate gene promoter region in the GPP population under e7. **(J)** Haplotype validation of the candidate gene promoter region in the GPP population under e8. *, **, ***, and **** indicate significant differences at P < 0.05, P < 0.01, P < 0.001, and P < 0.0001, respectively.

Haplotype analysis in the FW-RIL population identified a single-base mutation (A/G) at position 4,341,757 bp of the CDS in 86 lines, resulting in an amino acid change from Arg to Gln. Based on this, two haplotypes were defined: HSW-I (variant) and HSW-II (Williams 82 type). Lines carrying HSW-II (n=45) exhibited significantly higher mean HSW than HSW-I lines (n=41) (**Figure 4A**), indicating that this non-synonymous mutation significantly affects seed weight or HSW.

To evaluate the effect of *Glyma.02G047200* across different genetic backgrounds, 1,487 materials from the GPP population were tested in eight environments across four regions (**Supplementary Table 5**). In environments E2 and E3, HSW-II exhibited significantly higher seed weight than HSW-I (**Figures 4B, C**). In E6, the difference was not significant, but HSW-II remained higher, indicating a consistent trend (**Figure 4D**). These results suggest that HSW-II is a favorable haplotype.

Promoter variation analysis based on GPP resequencing revealed five haplotypes (pHSW-I to pHSW-V; **Figure 4E**). Across five environments, pHSW-III exhibited the highest HSW, significantly outperforming other haplotypes (**Figures 4F–J**), indicating that promoter variation also contributes to seed weight regulation.

### 3.6 Domestication-related selection and geographic distribution patterns

**Figure 5:**
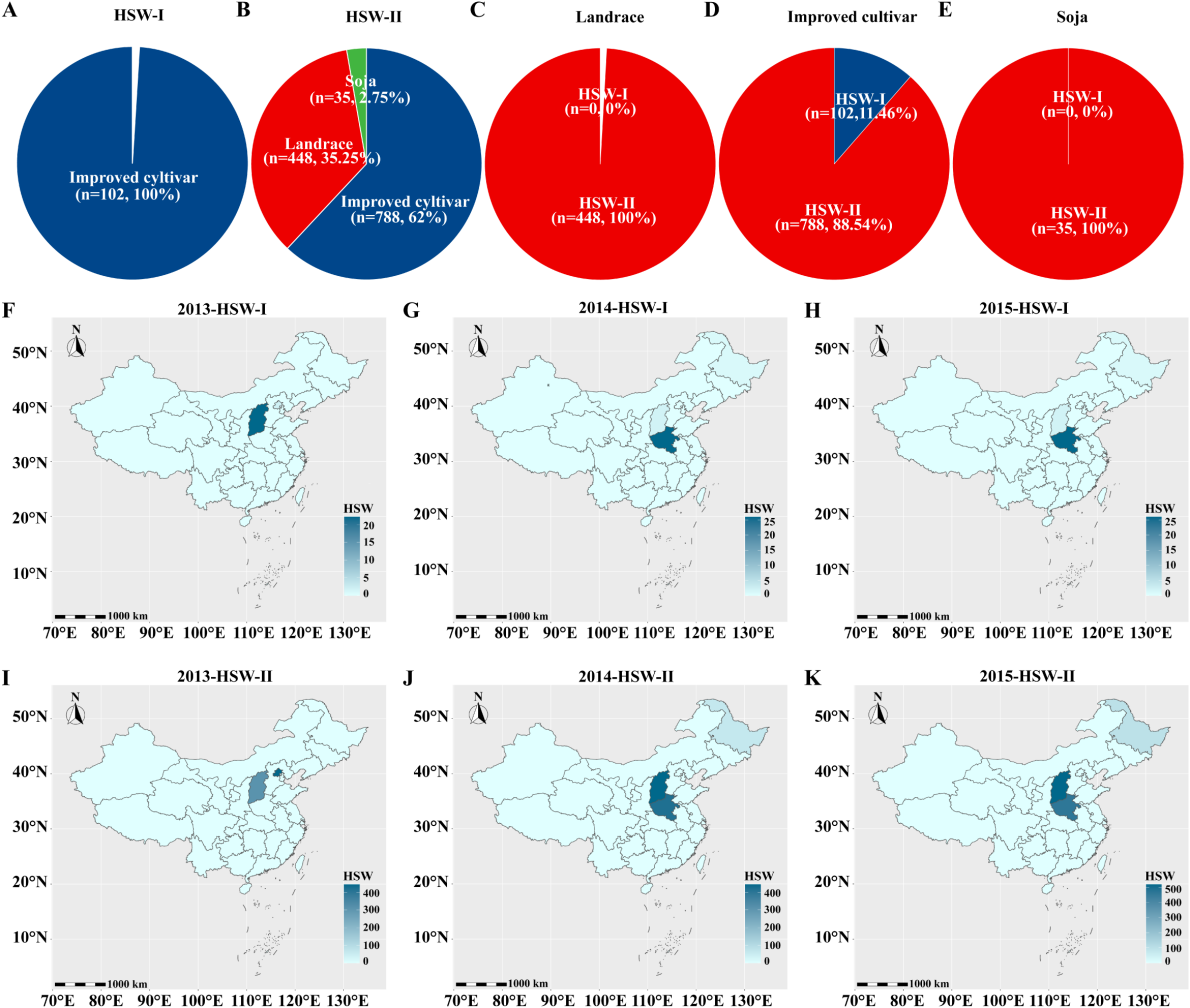
Distribution of distinct haplotypes of the candidate gene *Glyma.02G047200* among three varietal types and across geographical regions. **(A)** Distribution of the HSW-I genotype of the *Glyma.02G047200* gene among the three varietal types. **(B)** Distribution of the HSW-I genotype of the *Glyma.02G047200* gene among the three varietal types. **(C)** Distribution of the two genotypes of the *Glyma.02G047200* gene in the Landrace type. **(D)** Distribution of the two genotypes of the *Glyma.02G047200* gene in the Improved cultivar type. **(E)** Distribution of the two genotypes of the *Glyma.02G047200* gene in the Soja type. **(F)** Geographical distribution of the HSW-I haplotype of the *Glyma.02G047200* gene in 2013. **(G)** Geographical distribution of the HSW-I haplotype of the *Glyma.02G047200* gene in 2014. **(H)** Geographical distribution of the HSW-I haplotype of the *Glyma.02G047200* gene in 2015. **(I)** Geographical distribution of the HSW-II haplotype of the *Glyma.02G047200* gene in 2013. **(J)** Geographical distribution of the HSW-II haplotype of the *Glyma.02G047200* gene in 2014. **(K)** Geographical distribution of the HSW-II haplotype of the *Glyma.02G047200* gene in 2015.

To explore the evolutionary pattern of *Glyma.02G047200* during soybean improvement, haplotype frequencies were analyzed using resequencing data from 2,883 GPP accessions, including wild soybean (*G. soja*), landraces, and improved cultivars (**Figure 5**). The favorable haplotype HSW-II was fixed (100%) in wild soybean and landraces, indicating strong selection during early domestication and farmer breeding. In contrast, its frequency declined to 88.54% in improved cultivars, suggesting the introduction of the low seed weight haplotype HSW-I or shifts in allele frequency during modern breeding.

Geographic distribution analysis of 1,982 accessions revealed that HSW-I was largely confined to the Huang-Huai-Hai region (Shanxi, Henan), while HSW-II exhibited broader adaptation across Shanxi, Henan, Beijing, and dominated Northeast China. These results suggest that HSW-II may confer better adaptation to higher latitudes or specific environments, contributing to its maintenance during improvement.

### 3.7 Comparative analysis of genomic selection models and feature selection strategies

To evaluate the influence of feature selection strategies on predictive accuracy, genotype sets were constructed using five genomic windows (100 bp–100 kb) based on LD decay. Increasing the window size incorporated more markers, from 40 (GWAS-identified QTNs only) to 1,436 markers.

**Figure 6:**
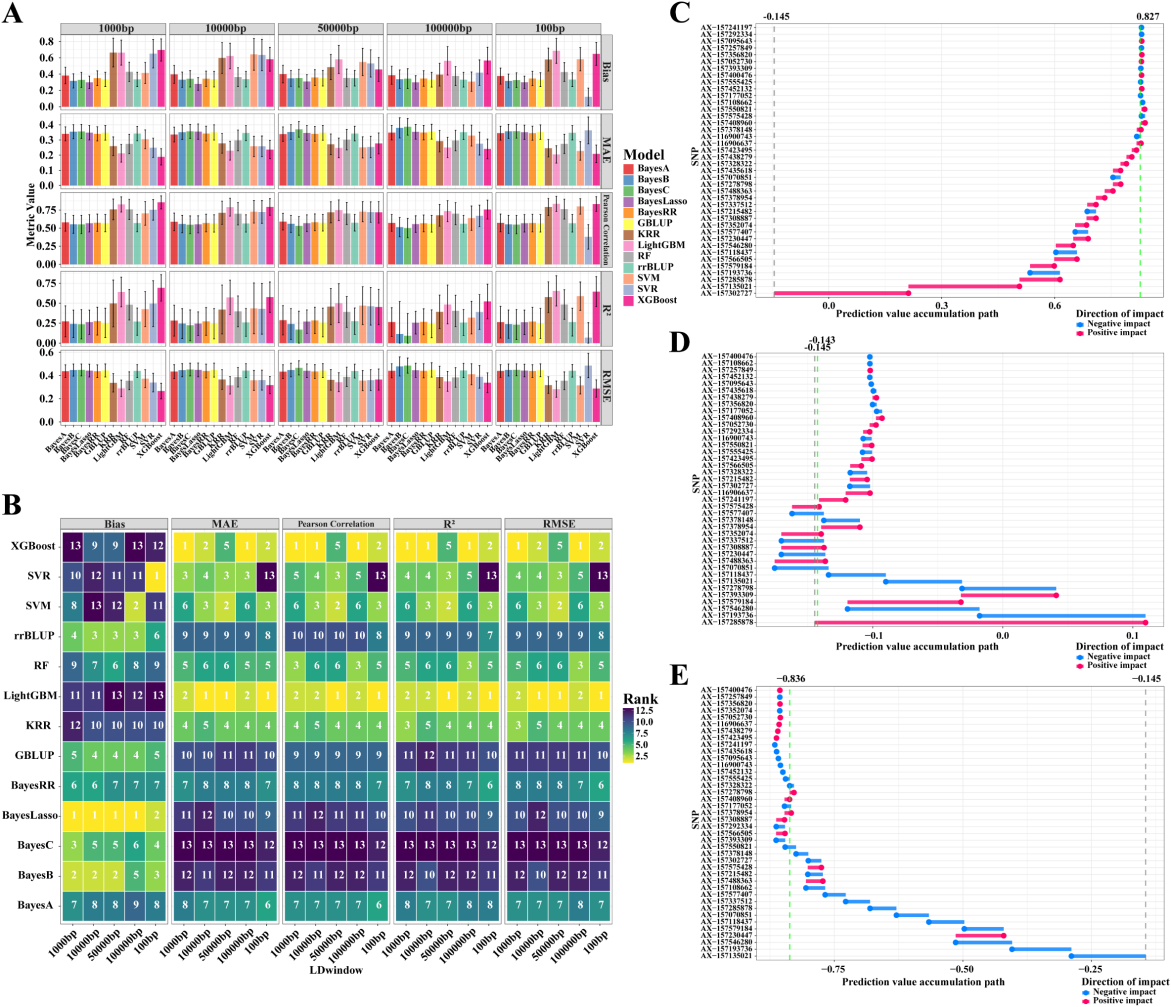
Ranking of 13 genomic selection models for HSW in the FW-RIL population. **(A)** Comprehensive comparison of models based on five evaluation metrics. **(B)** Performance ranking of 13 models. **(C)** Impact of 40 QTNs in accessions with high HSW breeding values. **(D)** Impact of 40 QTNs in accessions with medium HSW breeding values. **(E)** Impact of 40 QTNs in accessions with low HSW breeding values. In **(C–E)**, the black vertical lines represent the baseline breeding value (the mean GEBV of the FW-RIL population), and the green vertical lines represent the breeding values resulting from the cumulative impact of the 40 QTNs.

Cross-validation results (**Figure 6A**) showed that increasing marker density led to a general decline in predictive performance, as reflected by decreases in the Pearson correlation coefficient (Pearson) and coefficient of determination (R^2^) across most models, whereas Bias values fluctuated. These results indicate that the core QTNs identified within the 100 bp window harbor the primary genetic information governing hundred-seed weight (HSW), and that the inclusion of excessive, redundant markers introduces stochastic noise that reduces predictive efficiency.

Among the 13 evaluated models, the 100 bp (QTN-only) window produced optimal predictions, with all models achieving Pearson correlation coefficients exceeding 0.54 (minimum of 0.548 for the Bayes C model). Notably, the Light Gradient Boosting Machine (LightGBM) model showed the highest predictive accuracy (**Figure 6B**). Across various feature windows, LightGBM maintained stable performance, with Pearson coefficients ranging from 0.735 to 0.835 and R2 values between 0.484 and 0.654. Compared with other parametric and non-parametric models, LightGBM exhibited a robust capacity to effectively capture critical genetic variations, even in genotypic datasets affected by environmental or technical noise.

To further elucidate the contribution of individual markers to HSW, SHapley Additive exPlanations (SHAP) analysis was applied to the high-performing LightGBM model based on the 100 bp window. Germplasm accessions were categorized into high-, medium-, and low-HSW groups according to their Genomic Estimated Breeding Values (GEBVs) (**Supplementary Table 6**).

SHAP analysis revealed clear differences in the direction and magnitude of marker effects among groups. In the high-GEBV group, most markers exhibited positive SHAP values, indicating cumulative contributions to increased seed weight (**Figure 6C**). In contrast, markers in the medium-GEBV group displayed a balance of positive and negative contributions (**Figure 6D**), whereas the low-GEBV group predominantly showed negative effects (**Figure 6E**). Notably, marker AX-157566505 consistently demonstrated a significant positive contribution across SHAP evaluations (**Supplementary Table 7**; **Supplementary Figure 4**).

These machine-learning-based results are highly consistent with the QTL/GWAS co-localization and haplotype validation analyses, providing convergent evidence that AX-157566505 and its associated candidate gene, *Glyma.02G047200*, represent key genetic determinants of soybean HSW.

### 3.8 Reconstruction and high-impact breeding simulation

This study integrated 40 QTNs identified from GWAS and GS models to develop a molecular design breeding strategy. Marker effects were quantified using SHAP values, and QTNs with MeanSHAP > 0 were defined as superior alleles (SAs) (**Supplementary Table 7**). A total of 10,296 single-cross combinations were generated using 144 FW-RIL lines as parental materials. Selection efficiency and genetic gain were simulated and compared between the Pedigree and Bulk breeding methods under three F_2_ population sizes (N = 200, 500, and 800) using the B4L module of the ISB software (**Table 5**).

**Table 5:** Number of hybrids, genotype values and the number of allele variants under each breeding scheme.

Sample size	Methods	Bi-parental crosses		
		N	Genotypic value	NSA
200	Ped	824	14.7106-30.1417	17-32
200	Bulk	2059	13.471-31.3646	13-33
500	Ped	2059	10.9768-29.5722	12-33
500	Bulk	2059	12.9441-29.9619	13-34
800	Ped	3295	12.4095-29.8625	13-33
800	Bulk	2059	12.7499-30.4349	14-33
Parent	Ped&Bulk	144	9.31-25.58	14-30

NSA: Number of Superior Alleles.

The results indicated that population size could significantly influence selection efficiency (**Supplementary Tables 8-10**). At N = 200, the Bulk method outperformed the Pedigree method (2,059 vs. 824 effective combinations), whereas at N = 800, the Pedigree method showed higher efficiency (3,295 combinations). Genetic values (GVs) of the offspring were significantly higher than those of the parents, with the Bulk method exhibiting slightly higher genetic gain potential. Notably, under F_2_ = 200, the Bulk cross HN116 × HN129 achieved the highest GV (31.3646).

Phenotypic improvement was primarily driven by superior allele pyramiding, with offspring accumulating 12–34 SAs, exceeding the parental range. For example, at F_2_ = 500, the Bulk cross HN012 × HN083 aggregated 34 SAs. Ultimately, nine promising hybrid combinations were identified, providing a theoretical basis for high-yield molecular design breeding in soybean.

## 4 Discussion

This study employed a four-way recombinant inbred line (FW-RIL) population and an integrated strategy combining high-density linkage QTL mapping with multi-locus GWAS to dissect the genetic architecture of HSW in soybean. Several stable genetic loci were consistently detected across environments and methods, and a major candidate gene, *Glyma.02G047200*, was functionally validated. Building on these findings, the performance of GS models for HSW prediction was evaluated, and simulated breeding was conducted to identify potential high-yield hybrid combinations harboring multiple superior alleles. Together, these results provide a theoretical and practical framework for molecular breeding aimed at improving soybean seed weight.

### 4.1 Advantages of FW-RIL populations in dissecting soybean yield-related traits

Conventional biparental RIL populations are often constrained by limited allelic diversity, restricting their ability to fully capture the genetic basis of complex traits (Vieira et al., 2006; Chen et al., 2007; Liu et al., 2011). In contrast, our FW-RIL population integrates alleles from four founding lines exhibiting significant HSW variations. This multi-parent design effectively expanded the allelic spectrum, resulting in continuous phenotypic variation and pronounced transgressive segregation. Consequently, it improved both the mapping resolution and the detection power for previously hidden rare alleles, demonstrating its superiority for dissecting complex yield-related traits (Bhat et al., 2025).

### 4.2 Environmental Interactions and Co-localization of Genetic Loci

Hundred-seed weight is a complex trait sensitive to environmental fluctuations. In our study, the broad-sense heritability of HSW varied from 49% to 79% across the four environments, accompanied by highly significant G × E interactions (**Table 2**). Such strong interactions profoundly affect mapping results by modulating gene expression under specific conditions, which explains the detection of environment-specific loci (e.g., *qHSW-17–1* and *qHSW-15-1*). This highlights the critical need for multi-environment trials to identify stable loci.

In addition to environmental factors, mapping methodologies also influence locus detection. Although we identified 40 QTNs and 16 QTLs, only a small proportion overlapped (six QTNs within four QTLs). This limited overlap is a common phenomenon when integrating linkage mapping and GWAS, arising from their distinct mapping principles. Linkage mapping in our FW-RIL population captures recent recombination events and founder-specific alleles within broader intervals, whereas GWAS exploits historical recombination, offering higher resolution but occasionally missing rare variants due to strict minor allele frequency thresholds. Therefore, these two approaches are highly complementary. Loci robustly detected across multiple environments and by both methods, such as *qHSW-2-1*, represent exceptionally reliable genetic regions. Chromosome 2 has been widely recognized as a major hotspot regulating soybean seed weight. The *qHSW-2–1* locus identified here substantially overlaps with *qSW-2–1* reported previously (He et al., 2024), and our high-resolution mapping further identified novel superior allelic variations within this hotspot that have not been fully exploited in previous breeding efforts.

### 4.3 Functional prioritization and mechanistic insights into *Glyma.02G047200*

Within the stable *qHSW-2–1* interval, *Glyma.02G047200* emerged as the core candidate gene. Encoding an oligopeptide transporter of the PTR family, it mediates the transport of nitrogenous compounds crucial for nutrient accumulation in sink organs (Song et al., 1996; Delrot et al., 2000; Gallais and Hirel, 2004). Consistent with this function, our RNA-seq analysis confirmed its high, seed-specific expression during the grain-filling stage, suggesting it acts as a rate-limiting factor for protein synthesis and photosynthate accumulation during seed development. Furthermore, our sequence variation analysis provided a dual mechanistic insight: a missense mutation (Arg to Gln) in the coding region likely alters transport efficiency, while promoter variation contributes to transcriptional regulation. This synergy confers a significant seed weight advantage to the elite haplotype HSW-II across diverse backgrounds.

### 4.4 Prospects of genomic selection for predicting and improving soybean HSW

Genomic selection (GS) has the potential to substantially accelerate genetic gain. Among the 13 models evaluated in our study, LightGBM consistently outperformed others in predicting HSW, maintaining high accuracy across different marker sets. This aligns with findings in other crops where optimized marker sets enhanced prediction accuracy (Zhang et al., 2025c). Rather than relying on purely theoretical platforms, our SHAP-based interpretability analysis grounded the model by revealing the specific direction and magnitude of marker effects (e.g., AX-157566505) across distinct HSW genotypes. By demonstrating that marker sets derived from our GWAS loci and LD regions achieved competitive prediction accuracy, we offer a highly cost-effective and crop-specific strategy for implementing GS in soybean (Ma et al., 2024; Han et al., 2026).

### 4.5 Simulated breeding as a framework for parental selection and cross optimization

By integrating empirically derived QTNs and their effect values, we simulated HSW improvement using the B4L module. Unlike simulations based on theoretical assumptions, our genetic architecture was grounded in real multi-environment field data, significantly increasing its practical breeding value (Chiaravallotti et al., 2024). The simulation outcomes translated our locus discoveries into actionable crossing schemes, demonstrating that parental selection based on favorable allele pyramiding significantly increased the predicted genetic gain. Ultimately, we identified nine optimal parental combinations aggregating 27–34 key loci, successfully converting molecular findings into a concrete blueprint for high-yield molecular design breeding in soybean.

## 5 Conclusions

Using an FW-RIL population and an integrated linkage mapping and GWAS strategy, this study systematically dissected the genetic architecture of soybean HSW. Three stable QTLs and two key QTNs were identified, and a major candidate gene, *Glyma.02G047200*, encoding an oligopeptide transporter, was functionally anchored. The elite haplotype of this gene exhibited significant and stable effects across diverse genetic backgrounds, making it a promising target for marker-assisted selection. In addition, this study established the superiority of the LightGBM model for HSW prediction and developed optimized parental crossing schemes to pyramid multiple favorable alleles. Overall, this study establishes a coherent pipeline from “gene mining” to “design breeding,” providing genetic resources, prediction models, and crossing schemes to overcome yield limitations in soybean.

## Data Availability

The original contributions presented in the study are included in the article/**Supplementary Material**. Further inquiries can be directed to the corresponding authors.
