# Supplementary material for: Mining genes, genomic selection and simulation breeding on hundred-seed weight using a four-way RIL population
Source: Front Plant Sci. 2026 Apr 23;17:1812087. doi: 10.3389/fpls.2026.1812087 (PMC13149278; doi:10.3389/fpls.2026.1812087)
Supplement: Supplementary file 1 [file DataSheet1.docx]

Supplementary Material

# Supplementary Figures and Tables

## Supplementary Figures

**
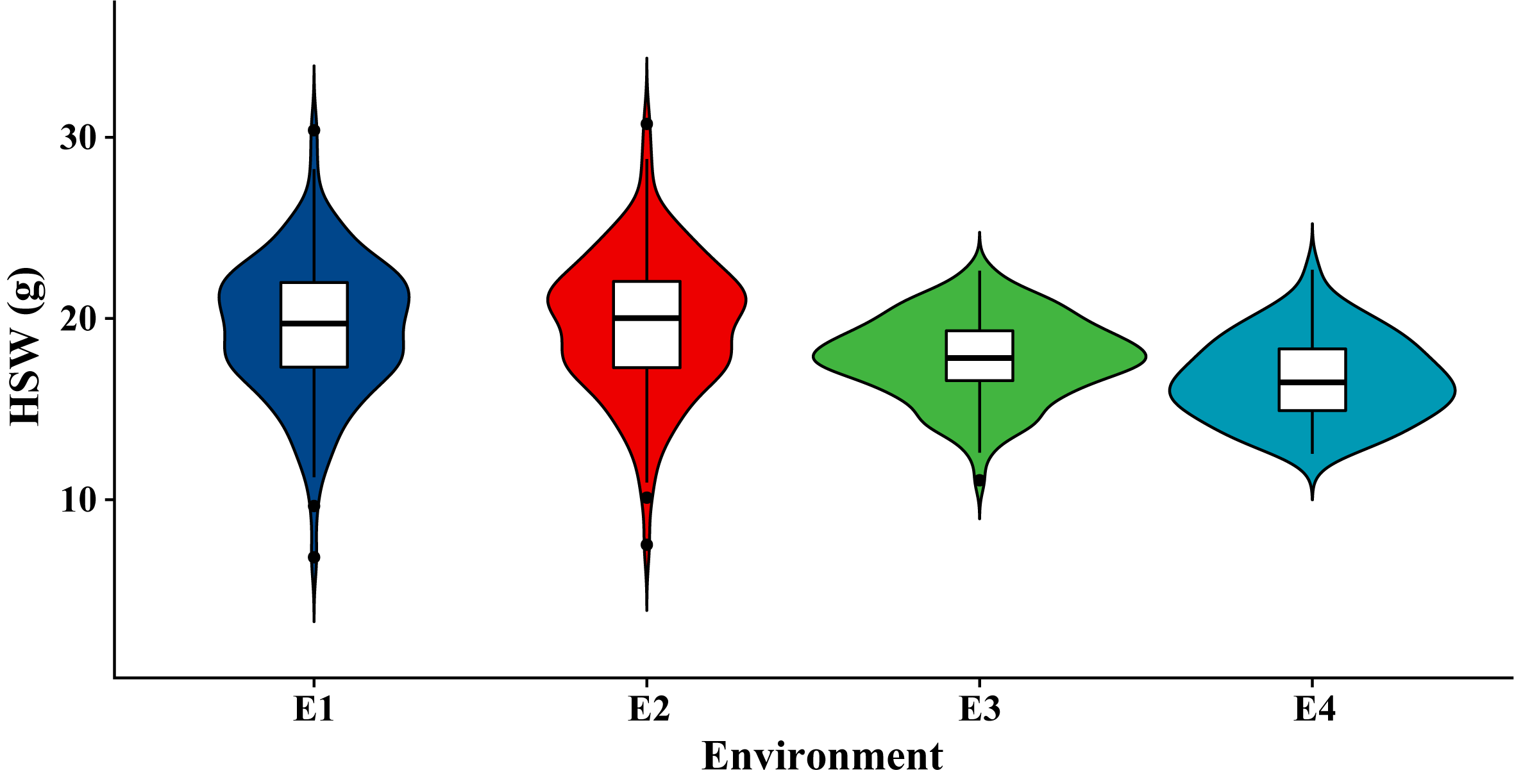
**

**Supplementary Figure 1.** Phenotypic variation in HSW in the FW-RIL across 4 environments.

**Supplementary Figure 2.** QTL analysis of HSW in the FW-RIL. Distribution of QTLs associated with soybean HSW across 20 chromosomes. The black markings indicate QTLs detected by a single method in a single environment, the blue markings indicate QTLs detected by multiple methods in a single environment, the green markings indicate QTLs detected by a single method across multiple environments, and the red markings indicate QTLs detected by multiple methods across multiple environments.

**Supplementary Figure 3.** Positional comparison of identified QTNs with previously reported loci. The red markings indicate QTLs and QTNs overlapping with previously reported loci, the blue markings indicate QTLs and QTNs that do not overlap with previously reported loci, and the black markings indicate the previously reported QTLs and QTNs.

**Supplementary Figure 4.** Feature importance ranking of SNPs based on mean absolute SHAP values derived from the optimal Light Gradient-Boosting Machine model (100 bp window). (**A**) Feature importance ranking of the 40 QTNs based on mean absolute SHAP values. (**B**) Direction of cumulative SHAP impact for the 40 QTNs.

**Supplementary Table 1.** descriptive statistical analysis on HSW in FW-RIL.

| Environment | Parents | | | | | FW-RIL | | | | | | |
| --- | --- | --- | --- | --- | --- | --- | --- | --- | --- | --- | --- | --- |
|  | Kenfeng14 | Kenfeng15 | Heinong48 | Kenfeng19 | Range | Min | Max | Range | Mean | Std^a^ | Skewness^b^ | Kurtosis^c^ |
| E1 |  |  |  |  |  | 6.82 | 30.39 | 23.57 | 19.44 | 3.73 | -0.30 | 0.74 |
| E2 |  |  |  |  |  | 7.51 | 30.74 | 23.23 | 19.65 | 3.76 | -0.20 | 0.64 |
| E3 | 18.28 | 17.24 | 22.63 | 11.07 | 11.56 | 11.07 | 22.63 | 11.56 | 17.81 | 2.26 | -0.31 | -0.09 |
| E4 |  |  |  |  |  | 12.53 | 22.69 | 10.16 | 16.64 | 2.27 | 0.29 | -0.50 |

a, Standard Deviation, indicating the phenotypic diversity of HSW within the mapping population. b, Indicates the deviation of the HSW distribution from symmetry. c, Indicates the peakedness of the HSW distribution.

**Supplementary Table 2.** QTL controlling HSW in FW-RIL.

| Method | Chromosome | Name | Position | LeftMarker | RightMarker | LOD^a^ | PVE(%)^b^ | Environment | Rreported QTL |
| --- | --- | --- | --- | --- | --- | --- | --- | --- | --- |
| IM | 2 | qHSW-2-1 | 1 | Gm02_5560717 | Gm02_3952555 | 3.2951 | 6.6948 | E3 | Seed weight 51-1 |
| ICIM |  |  |  |  |  | 3.8556 | 14.5906 | E3 | - |
| IM | 8 | qHSW-8-1 | 173 | Gm08_4528693 | Gm08_4265916 | 3.2261 | 6.7887 | E3 | Seed weight 1-1, Seed weight 34-10, Seed weight 49-1 |
| ICIM | 9 | qHSW-9-1 | 7 | Gm09_1206616 | Gm09_32032649 | 4.4674 | 12.3194 | E4 | Seed weight 35-6, Seed weight 10-10, Seed weight 34-6, Seed weight 36-6, Seed weight 15-6, Seed weight 30-5 |
| IM |  |  |  |  |  | 4.4674 | 12.3194 | E4 | - |
| ICIM | 9 | qHSW-9-2 | 19 | Gm09_6256462 | Gm09_5866504 | 3.8642 | 8.2294 | E4 | Seed weight 35-6 |
| IM |  |  |  |  |  |  |  |  | - |
| ICIM | 12 | qHSW-12-1 | 25 | Gm12_35990600 | Gm12_36275137 | 3.6857 | 11.2133 | E1 | Seed weight 13-8, Seed weight 34-4, Seed weight 36-4, Seed weight 41-1 |
| ICIM |  |  |  |  |  | 4.3844 | 12.9712 | E2 | - |
| IM |  |  |  |  |  | 3.1848 | 10.8302 | E1 | - |
| IM |  |  |  |  |  | 3.7979 | 13.4527 | E2 | - |
| ICIM | 12 | qHSW-12-2 | 89 | Gm12_16069251 | Gm12_13868525 | 3.3131 | 7.5335 | E4 | New |
| IM |  |  |  |  |  | 3.3131 | 7.5335 | E4 | - |
| IM | 15 | qHSW-15-1 | 82 | Gm15_50600618 | Gm15_48703253 | 3.5944 | 9.4347 | E3 | New |
| IM | 15 | qHSW-15-2 | 107 | Gm15_15781408 | Gm15_15129437 | 3.9193 | 8.8058 | E3 | Seed weight 2-3, Seed weight 34-12, Seed weight 6-1, Seed weight 33-2 |
| IM | 17 | qHSW-17-1 | 82 | Gm17_34983373 | Gm17_37050676 | 3.7279 | 13.0765 | E1 | Seed weight 47-2, Seed weight 49-10, Seed weight 34-17, Seed weight 50-3 |
| ICIM | 17 | qHSW-17-2 | 85 | Gm17_36850271 | Gm17_36557987 | 4.3057 | 12.7187 | E1 | Seed weight 47-2, Seed weight 49-10, Seed weight 34-17, Seed weight 50-3 |
| ICIM |  |  |  |  |  | 4.0372 | 11.4503 | E2 | - |
| ICIM | 17 | qHSW-17-3 | 140 | Gm17_10433088 | Gm17_7565828 | 3.0587 | 7.8931 | E2 | Seed weight 47-2, Seed weight 18-2, Seed weight 42-2, Seed weight 13-5, Seed weight 49-10, Seed weight 52-8 |
| ICIM | 18 | qHSW-18-1 | 43 | Gm18_2759157 | Gm18_10333228 | 3.0867 | 6.2146 | E4 | - |
| IM |  |  |  |  |  | 3.0867 | 6.2146 | E4 | - |
| IM | 18 | qHSW-18-2 | 74 | Gm18_55369435 | Gm18_55472624 | 3.287 | 6.9354 | E3 | New |
| ICIM | 18 | qHSW-18-3 | 128 | Gm18_1553751 | Gm18_1764099 | 3.4299 | 6.9375 | E4 | New |
| IM |  |  |  |  |  | 3.4299 | 6.9375 | E4 | - |
| ICIM | 19 | qHSW-19-1 | 51 | Gm19_30930458 | Gm19_35961145 | 3.1766 | 12.2647 | E4 | Seed weight 12-5 |
| IM |  |  |  |  |  | 3.1766 | 12.2647 | E4 | - |
| IM | 19 | qHSW-19-2 | 193 | Gm19_50284807 | Gm19_38866655 | 3.6974 | 7.4152 | E3 | Seed weight 35-7, Seed weight 5-1, Seed weight 34-7, Seed weight 15-7, Seed weight 17-1, Seed weight 36-7, Seed weight 7-7, Seed weight 43-4, Seed weight 3-5, Seed weight 42-4, Seed weight 13-9, Seed weight 12-3, Seed weight 4-6 |

a, Logarithm of the Odds score. It indicates the strength of statistical evidence for the presence of a QTL at the given genomic location. b, The percentage of Phenotypic Variance Explained by the identified QTL. It represents the magnitude of the effect of the locus on the HSW trait.

**Supplementary Table 3.** QTN associated with HSW in FW-RIL.

| Trait name | Method | Name | Chr | Marker position (bp) | QTN effect^a^ | LOD score^b^ | r^2^ (%)^c^ | Genotype for code 1 | Environment | Rported QTL |
| --- | --- | --- | --- | --- | --- | --- | --- | --- | --- | --- |
| E2HSW | mrMLM |  |  |  | 2.0361 | 4.7087 | 20.8087 | AA | E2 | no |
| E1HSW | mrMLM | AX-157438279 | 1 | 245959 | 1.2504 | 3.8744 | 7.6918 | GG | E1 | no |
| E4HSW | FASTmrMLM | AX-157177052 | 1 | 39442305 | 0.8691 | 4.4046 | 11.7583 | TT | E4 | Seed weight 18-1.1, Seed weight 52-1, Seed weight 15-2, Seed weight 35-10 |
| E1HSW | pLARmEB | AX-157566505 | 2 | 4410313 | -1.188 | 4.2881 | 8.9418 | CC | E1 | Seed weight 51-1 |
| E2HSW | pLARmEB |  |  |  | -1.1375 | 4.3535 | 8.1987 | CC | E2 |  |
| E4HSW | FASTmrMLM | AX-157308887 | 2 | 47566811 | -1.4128 | 5.2438 | 15.6261 | CC | E4 | Seed weight 50-12, Seed weight 49-9 |
| E2HSW | mrMLM | AX-157118437 | 3 | 27518531 | 1.6729 | 4.7862 | 15.871 | AA | E2 | no |
| E2HSW | FASTmrMLM |  |  |  | 1.2185 | 5.3321 | 9.6305 | AA | E2 | no |
| E4HSW | mrMLM | AX-157555425 | 3 | 34919909 | 1.3361 | 4.0787 | 18.415 | TT | E4 | no |
| E1HSW | mrMLM | AX-157550821 | 5 | 2642885 | 1.2478 | 4.4931 | 8.1233 | GG | E1 | no |
| E2HSW | pLARmEB |  |  |  | 0.9302 | 3.0327 | 4.5152 | GG | E2 | no |
| E4HSW | ISIS EM-BLASSO | AX-157423495 | 6 | 12326909 | -1.0462 | 4.524 | 19.154 | TT | E4 | no |
| E4HSW | mrMLM | AX-157372053 | 6 | 49361535 | -1.6995 | 6.4576 | 29.7933 | AA | E4 | Seed weight 49-4 |
| E4HSW | FASTmrEMMA |  |  |  | -2.8014 | 3.4729 | 20.6458 | GG | E4 | Seed weight 49-4 |
| E4HSW | FASTmrMLM | AX-157285878 | 8 | 4905525 | 0.9972 | 5.3319 | 17.1287 | AA | E4 | Seed weight 34-10, Seed weight 49-1 |
| E4HSW | FASTmrMLM | AX-157302727 | 9 | 3271495 | -0.8376 | 3.279 | 9.3701 | CC | E4 | Seed weight 35-6, Seed weight 10-10 |
| E1HSW | mrMLM | AX-157337512 | 9 | 40850058 | 1.128 | 4.9221 | 8.6931 | TT | E1 | no |
| E2HSW | ISIS EM-BLASSO | AX-157400476 | 9 | 40907289 | 0.9818 | 3.2219 | 6.5401 | TT | E2 | no |
| E2HSW | mrMLM | AX-157435618 | 11 | 8189393 | -2.1472 | 5.02 | 18.533 | AA | E2 | Seed weight 37-9, Seed weight 25-2 |
| E2HSW | FASTmrMLM |  |  |  | -1.4208 | 4.697 | 9.2811 | CC | E2 | Seed weight 37-9, Seed weight 25-2 |
| E3HSW | pLARmEB | AX-157352074 | 11 | 14879003 | 0.7201 | 4.2975 | 6.9078 | CC | E3 | Seed weight 20-1, Seed weight 20-4, Seed weight 20-3, Seed weight 4-1, Seed weight 11-1 |
| E2HSW | ISIS EM-BLASSO | AX-157378148 | 11 | 32879122 | -1.8684 | 5.7914 | 23.4225 | CC | E2 | Seed weight 34-14 |
| E1HSW | pLARmEB | AX-157241197 | 12 | 1522694 | -1.2121 | 3.2711 | 7.2278 | TT | E1 | Seed weight 16-3 |
| E1HSW | FASTmrMLM | AX-157393309 | 12 | 2812360 | -1.2877 | 3.3861 | 9.1052 | CC | E1 | Seed weight 16-3, Seed weight 43-3 |
| E1HSW | ISIS EM-BLASSO | AX-157356820 | 12 | 2844288 | -1.4666 | 3.9993 | 12.3447 | AA | E1 | Seed weight 16-3, Seed weight 43-3 |
| E1HSW | mrMLM | AX-116906637 | 12 | 9121409 | 1.5363 | 5.3608 | 13.545 | AA | E1 | no |
| E2HSW | FASTmrMLM |  |  |  | 1.2642 | 3.6702 | 9.1751 | AA | E2 | no |
| E4HSW | pLARmEB | AX-157052730 | 12 | 14744106 | -1.0949 | 4.2475 | 13.6908 | NN | E4 | no |
| E1HSW | mrMLM | AX-157328322 | 12 | 15566266 | -1.3862 | 4.1184 | 8.8319 | CC | E1 | no |
| E2HSW | FASTmrEMMA | AX-157328322 | 12 | 15566266 | -0.00001762 | 3.7116 | 3.5923e-10 | CC | E2 | no |
| E1HSW | FASTmrMLM | AX-157452132 | 12 | 16082217 | -2.0876 | 5.7492 | 18.5147 | AA | E1 | no |
| E2HSW | pLARmEB | AX-157257849 | 12 | 16317590 | 2.581 | 8.4493 | 34.7626 | CC | E2 | no |
| E1HSW | pLARmEB | AX-157408960 | 12 | 16487770 | 2.7699 | 3.3241 | 35.2642 | CC | E1 | no |
| E1HSW | ISIS EM-BLASSO | AX-116900743 | 12 | 17318628 | 2.2506 | 6.2897 | 23.2824 | CC | E1 | no |
| E2HSW | ISIS EM-BLASSO |  |  |  | 1.8569 | 4.3377 | 15.8521 | CC | E2 | no |
| E3HSW | mrMLM | AX-157278798 | 13 | 10297850 | -1.7655 | 4.2102 | 35.8397 | TT | E3 | no |
| E3HSW | pLARmEB |  |  |  | -1.3953 | 5.5109 | 18.6065 | TT | E3 | no |
| E3HSW | ISIS EM-BLASSO |  |  |  | -1.3237 | 4.2106 | 22.6847 | TT | E3 | no |
| E1HSW | pLARmEB | AX-157488363 | 13 | 15296150 | 1.2165 | 4.2893 | 8.1259 | TT | E1 | no |
| E2HSW | pLARmEB |  |  |  | 0.9295 | 3.3016 | 4.7448 | TT | E2 | no |
| E1HSW | mrMLM | AX-157378954 | 13 | 28263890 | -1.2369 | 4.8227 | 10.263 | TT | E1 | Seed weight 42-3, Seed weight 44-2 Seed weight 15-3, Seed weight 19-2, Seed weight 2-4, Seed weight 49-14, Seed weight 50-7 |
| E2HSW | FASTmrMLM |  |  |  | -0.8039 | 3.3218 | 4.3365 | TT | E2 |  |
| E1HSW | FASTmrMLM | AX-157193736 | 13 | 30177239 | 1.7678 | 4.6531 | 12.1126 | GG | E1 | Seed weight 44-2, Seed weight 49-14, Seed weight 40-1, Seed weight 41-2, Seed weight 49-13 |
| E3HSW | pLARmEB | AX-157135021 | 14 | 16998076 | 0.6163 | 4.0755 | 4.4002 | TT | E3 | Seed weight 13-2, Seed weight 23-1, Seed weight 10-4, Seed weight 13-3 |
| E1HSW | mrMLM | AX-157577407 | 15 | 1846762 | 1.0741 | 4.4476 | 7.8824 | CC | E1 | no |
| E2HSW | mrMLM | AX-157230447 | 15 | 2353940 | 1.7859 | 5.2749 | 18.9202 | AA | E2 | no |
| E2HSW | FASTmrMLM | AX-157215482 | 15 | 2400557 | 1.3121 | 6.4612 | 11.5508 | TT | E2 | no |
| E3HSW | pLARmEB | AX-157070851 | 16 | 2830814 | -0.8151 | 4.5514 | 6.3501 | AA | E3 | Seed weight 2-6, Seed weight 4-4 |
| E4HSW | pLARmEB | AX-157095643 | 16 | 2945163 | -1.532 | 6.0491 | 26.8039 | GG | E4 | Seed weight 2-6, Seed weight 4-4 |
| E4HSW | mrMLM | AX-157579184 | 16 | 27365719 | -1.1427 | 5.399 | 21.0457 | TT | E4 | Seed weight 34-18 |
| E3HSW | pLARmEB | AX-157108662 | 17 | 40105633 | -0.6601 | 5.1557 | 5.7844 | AA | E3 | Seed weight 47-2 |
| E1HSW | ISIS EM-BLASSO | AX-157292334 | 19 | 45405934 | 1.1255 | 3.1949 | 8.5914 | CC | E1 | Seed weight 7-7, Seed weight 3-5, Seed weight 42-4, Seed weight 13-9 |
| E4HSW | pLARmEB | AX-157546280 | 19 | 46155659 | -0.8124 | 3.8022 | 11.184 | AA | E4 | Seed weight 42-4, Seed weight 13-9 |

a, Additive effect. Positive values denote an increase in hundred-seed weight (HSW). b, Logarithm of the odds (LOD) score, used to assess the significance of the association; in this study, an LOD score ≥ 3.0 was defined as the threshold for significance. c, Phenotypic variation explained (PVE) by an individual QTN.

**Supplementary Table 4.** Identify genes within the positioning interval.

| QTL | QTN | Gene | marker Position(bp) | mutation | Functional annotation |
| --- | --- | --- | --- | --- | --- |
| qHSW-2-1 | AX-157566505 | *Glyma.02G046900* | - | - | regulatory particle non-ATPase 10 |
| qHSW-2-1 | AX-157566505 | *Glyma.02G047000* | - | - | sulfurtransferase 18 |
| qHSW-2-1 | AX-157566505 | *Glyma.02G047100* | - | - | S-adenosyl-L-methionine-dependent methyltransferases superfamily protein |
| qHSW-2-1 | AX-157566505 | *Glyma.02G047200* | 4342156 | KF15 Exon nonsynonymous mutation A-G | oligopeptide transporter 7 |
| qHSW-2-1 | AX-157566505 | *Glyma.02G047300* | - | - | S-adenosyl-L-methionine-dependent methyltransferases superfamily protein |
| qHSW-2-1 | AX-157566505 | *Glyma.02G047400* | - | - | Pathogenesis-related thaumatin superfamily protein |
| qHSW-2-1 | AX-157566505 | *Glyma.02G047500* | - | - | cold shock domain protein 1 |
| qHSW-2-1 | AX-157566505 | *Glyma.02G047600* | 4379130 | H48 Exon nonsynonymous mutation A-C | ferredoxin-NADP(+)-oxidoreductase 1 |
| qHSW-2-1 | AX-157566505 | *Glyma.02G047700* | - | - | armadillo repeat only 2 |
| qHSW-2-1 | AX-157566505 | *Glyma.02G047800* | - | - | Ribosomal protein S4 (RPS4A) family protein |
| qHSW-2-1 | AX-157566505 | *Glyma.02G047900* | - | - | Chaperone DnaJ-domain superfamily protein |
| qHSW-2-1 | AX-157566505 | *Glyma.02G048000* | - | - | Chaperone DnaJ-domain superfamily protein |
| qHSW-2-1 | AX-157566505 | *Glyma.02G048100* | - | - | TSL-kinase interacting protein 1 |
| qHSW-2-1 | AX-157566505 | *Glyma.02G048200* | - | - | endonuclease/exonuclease/phosphatase family protein |
| qHSW-2-1 | AX-157566505 | *Glyma.02G048300* | - | - | calcium-dependent protein kinase 28 |
| qHSW-2-1 | AX-157566505 | *Glyma.02G048400* | - | - | flavanone 3-hydroxylase |
| qHSW-2-1 | AX-157566505 | *Glyma.02G048500* | - | - | Gibberellin-regulated family protein |
| qHSW-2-1 | AX-157566505 | *Glyma.02G048600* | - | - | flavanone 3-hydroxylase |
| qHSW-2-1 | AX-157566505 | *Glyma.02G048700* | - | - | Chalcone-flavanone isomerase family protein |
| qHSW-2-1 | AX-157566505 | *Glyma.02G048800* | - | - | - |
| qHSW-2-1 | AX-157566505 | *Glyma.02G048900* | - | - | zinc finger (Ran-binding) family protein |
| qHSW-2-1 | AX-157566505 | *Glyma.02G049000* | - | - | glycosyl hydrolase 9B5 |
| qHSW-2-1 | AX-157566505 | *Glyma.02G049100* | - | - | - |
| qHSW-2-1 | AX-157566505 | *Glyma.02G049200* | - | - | SAUR-like auxin-responsive protein family |
| qHSW-2-1 | AX-157566505 | *Glyma.02G049300* | - | - | Ribosomal protein L2 family |
| qHSW-2-1 | AX-157566505 | *Glyma.02G049400* | - | - | Peptide methionine sulfoxide reductase family protein |
| qHSW-2-1 | AX-157566505 | *Glyma.02G049500* | - | - | peptidylprolyl cis/trans isomerase, NIMA-interacting 1 |
| qHSW-2-1 | AX-157566505 | *Glyma.02G049600* | - | - | Zinc finger C-x8-C-x5-C-x3-H type family protein |
| qHSW-12-2 | AX-157328322 | *Glyma.12G134000* | - | - | - |
| qHSW-12-2 | AX-157328322 | *Glyma.12G134100* | - | - | Ubiquitin carboxyl-terminal hydrolase family protein |
| qHSW-12-2 | AX-157328322 | *Glyma.12G134200* | - | - | Dynein light chain type 1 family protein |
| qHSW-12-2 | AX-157328322 | *Glyma.12G134300* | - | - | Protein of unknown function (DUF1223) |
| qHSW-12-2 | AX-157328322 | *Glyma.12G134400* | 15567559 | KF19 Exon nonsynonymous mutation G-A | electron transfer flavoprotein alpha |
| qHSW-12-2 | AX-157328322 | *Glyma.12G134500* | - | - | PDI-like 5-4 |
| qHSW-12-2 | AX-157328322 | *Glyma.12G134600* | - | - | - |
| qHSW-12-2 | AX-157328322 | *Glyma.12G134700* | - | - | - |
| qHSW-12-2 | AX-157328322 | *Glyma.12G134800* | - | - | early nodulin-like protein 9 |
| qHSW-12-2 | AX-157328322 | *Glyma.12G134900* | - | - | Ypt/Rab-GAP domain of gyp1p superfamily protein |

**Supplementary Table 5.** Description of the eight environments for the GPP population.

| Environment | Location | Sowing date | Sample |
| --- | --- | --- | --- |
| e1 | Beijing | 2013 | 806 |
| e2 | Henan | 2014 | 994 |
| e3 | Henan | 2015 | 1022 |
| e4 | Heilongjiang | 2014 | 302 |
| e5 | Heilongjiang | 2015 | 368 |
| e6 | Shanxi | 2013 | 717 |
| e7 | Shanxi | 2014 | 1099 |
| e8 | Shanxi | 2015 | 1187 |

**Supplementary Table 6.** FW-RILs breeding value.

| Breed^a^ | BLUP_Phenotype^b^ | GEBV^c^ |
| --- | --- | --- |
| HN002 | 0.521760404 | 0.523131225 |
| HN003 | 0.50294292 | 0.500424113 |
| HN004 | -0.609711707 | -0.6080828 |
| HN005 | 0.238095894 | 0.238131397 |
| HN006 | 0.221919894 | 0.222234207 |
| HN007 | -0.018910335 | -0.018979297 |
| HN008 | -0.337332338 | -0.337668803 |
| HN009 | -0.04786906 | -0.048638113 |
| HN010 | 0.04520423 | 0.044799153 |
| HN011 | -0.483643323 | -0.485225486 |
| HN012 | -0.673502147 | -0.673424338 |
| HN014 | -0.517041504 | -0.518833819 |
| HN015 | -0.048981708 | -0.048973022 |
| HN016 | 0.087328024 | 0.087494277 |
| HN017 | 0.043924607 | 0.043155778 |
| HN018 | 0.222018808 | 0.221932115 |
| HN019 | 0.021294698 | 0.021612734 |
| HN020 | -0.635956407 | -0.635639395 |
| HN021 | -0.379213363 | -0.378913583 |
| HN022 | -0.636279583 | -0.636629311 |
| HN023 | 0.226472914 | 0.226191684 |
| HN024 | -0.066549882 | -0.066695083 |
| HN025 | 0.110978641 | 0.111225616 |
| HN026 | 0.359237492 | 0.359094049 |
| HN027 | 0.607871413 | 0.607638665 |
| HN029 | -0.334377706 | -0.333091325 |
| HN030 | 0.17022565 | 0.171764666 |
| HN031 | 0.244107053 | 0.24416384 |
| HN032 | -0.501131594 | -0.501157241 |
| HN033 | -0.383922398 | -0.383892957 |
| HN034 | 0.231690824 | 0.231780755 |
| HN035 | -0.0405946 | -0.04011377 |
| HN036 | 0.512286603 | 0.511691688 |
| HN037 | 0.245021641 | 0.24397449 |
| HN038 | -0.456315458 | -0.456426729 |
| HN039 | 0.415834129 | 0.41553756 |
| HN040 | 0.444967896 | 0.445675936 |
| HN041 | -0.023820005 | -0.024059764 |
| HN042 | 0.041105896 | 0.040931713 |
| HN043 | -0.997516751 | -0.997777714 |
| HN044 | -0.254527152 | -0.254308261 |
| HN045 | 0.345209688 | 0.345788706 |
| HN046 | -0.490210682 | -0.487951488 |
| HN047 | -0.330588102 | -0.331029328 |
| HN048 | -0.098090068 | -0.098395056 |
| HN049 | 0.333280355 | 0.331762982 |
| HN050 | -0.673502147 | -0.673424338 |
| HN051 | 0.161364958 | 0.162047253 |
| HN052 | -0.09984535 | -0.099475763 |
| HN053 | -0.512611926 | -0.513035935 |
| HN054 | 0.345209688 | 0.345788706 |
| HN055 | 0.724884868 | 0.726785125 |
| HN056 | -0.10299512 | -0.103134537 |
| HN057 | 0.146945506 | 0.14797352 |
| HN058 | -0.132151678 | -0.132393239 |
| HN059 | 0.071027912 | 0.071177143 |
| HN060 | -0.490570545 | -0.490289801 |
| HN061 | 0.695539355 | 0.69509181 |
| HN062 | -0.531318605 | -0.531049123 |
| HN063 | 0.773548782 | 0.773725761 |
| HN064 | 0.087328024 | 0.087494277 |
| HN065 | -0.219665319 | -0.220359814 |
| HN066 | 0.773207903 | 0.774690363 |
| HN067 | 0.754742861 | 0.755317361 |
| HN068 | -0.425718814 | -0.425742764 |
| HN069 | -1.229804873 | -1.229336375 |
| HN070 | -0.874765575 | -0.874919655 |
| HN071 | -0.53274399 | -0.533085996 |
| HN072 | -0.648121119 | -0.647603775 |
| HN073 | -0.889192879 | -0.889094045 |
| HN074 | 0.025682937 | 0.025489499 |
| HN075 | -0.622332394 | -0.620379986 |
| HN076 | -0.386839241 | -0.388412522 |
| HN077 | 0.553593874 | 0.553773882 |
| HN079 | -0.068580002 | -0.068414275 |
| HN080 | -0.761725783 | -0.761646453 |
| HN081 | -0.456315458 | -0.456426729 |
| HN082 | 0.05305057 | 0.053394856 |
| HN083 | 0.478491783 | 0.478517656 |
| HN084 | 0.829462171 | 0.829382241 |
| HN085 | -0.817893982 | -0.81774 |
| HN086 | -0.350873321 | -0.351108447 |
| HN087 | 0.64736551 | 0.647077521 |
| HN088 | -0.015446194 | -0.015607136 |
| HN090 | -0.487111747 | -0.486765798 |
| HN091 | 0.487142116 | 0.48710158 |
| HN092 | -0.500548124 | -0.499463615 |
| HN093 | 0.222018808 | 0.221932115 |
| HN094 | -0.084923998 | -0.084859872 |
| HN095 | 0.004138988 | 0.004276323 |
| HN096 | 1.106188297 | 1.106378721 |
| HN097 | 0.345005572 | 0.345245495 |
| HN098 | 0.011269415 | 0.010864457 |
| HN100 | 0.189074323 | 0.189055525 |
| HN101 | 0.085068859 | 0.084808916 |
| HN102 | -0.09984535 | -0.099475763 |
| HN103 | 0.543556392 | 0.544037441 |
| HN104 | -0.318313241 | -0.317896598 |
| HN105 | 0.733229697 | 0.731165402 |
| HN106 | 0.05120144 | 0.051166611 |
| HN107 | 0.146945506 | 0.14797352 |
| HN108 | -0.616676748 | -0.616432195 |
| HN109 | -0.931344271 | -0.931328246 |
| HN110 | -0.021531075 | -0.021120625 |
| HN111 | -1.436303377 | -1.436295525 |
| HN112 | -0.242582217 | -0.241473923 |
| HN113 | -1.207515955 | -1.207366776 |
| HN114 | -0.202317029 | -0.202874683 |
| HN115 | 0.234720632 | 0.235583887 |
| HN116 | -1.611145496 | -1.611264993 |
| HN117 | -0.092574283 | -0.092880987 |
| HN118 | 1.368467212 | 1.368525177 |
| HN119 | 0.13944231 | 0.139401098 |
| HN120 | 0.783792853 | 0.784261047 |
| HN121 | 0.238095894 | 0.238131397 |
| HN122 | 0.42119661 | 0.421021085 |
| HN123 | 0.793095291 | 0.793531202 |
| HN124 | 0.341304392 | 0.341261077 |
| HN125 | -0.318468302 | -0.317978901 |
| HN126 | 0.070054546 | 0.069786886 |
| HN127 | -0.036877301 | -0.037799517 |
| HN128 | 0.344115108 | 0.343947039 |
| HN129 | 0.707256794 | 0.707025945 |
| HN130 | 0.154672965 | 0.154057577 |
| HN131 | 1.106188297 | 1.106378721 |
| HN132 | 0.793902934 | 0.794078468 |
| HN133 | 0.044142477 | 0.042995728 |
| HN134 | 0.553593874 | 0.553773882 |
| HN135 | 0.788651049 | 0.788396806 |
| HN136 | -0.37804693 | -0.378575745 |
| HN137 | 0.25018698 | 0.25040729 |
| HN138 | -0.649456322 | -0.65091008 |
| HN139 | 0.600985229 | 0.600996501 |
| HN140 | 0.222018808 | 0.221932115 |
| HN141 | -0.357020587 | -0.357323333 |
| HN142 | -0.77559948 | -0.775931128 |
| HN143 | 1.112606287 | 1.111533898 |
| HN144 | 0.355105251 | 0.353418214 |
| HN145 | -0.456315458 | -0.456426729 |
| HN146 | 0.226472914 | 0.226191684 |
| HN147 | 0.499269634 | 0.499119469 |
| HN148 | -0.419888198 | -0.419768103 |
| HN149 | -0.08916194 | -0.088974854 |
| HN150 | 0.577390075 | 0.577252927 |

a, The specific identification code of the FW-RILs (Freshwater Recombinant Inbred Lines). b, Estimated breeding values (EBVs) derived solely from phenotypic data using the Best Linear Unbiased Prediction (BLUP) method. c, Genomic Estimated Breeding Values predicted using whole-genome marker information (e.g., SNPs) and phenotypic data.

**Supplementary Table 7.** The SHAP values of QTN associated with HSW in FW-RIL.

| Feature^a^ | MeanAbsSHAP^b^ | MeanSHAP^c^ | chr | pos | P_value |
| --- | --- | --- | --- | --- | --- |
| AX-157177052 | 0.010231115 | 0.071396353 | 1 | 39442305 | 6.68E-06 |
| AX-157438279 | 0.013148489 | -0.42082 | 1 | 245959 | 2.40E-05 |
| AX-157308887 | 0.033111231 | -0.011 | 2 | 47566811 | 8.92E-07 |
| AX-157566505 | 0.035485149 | -0.343042 | 2 | 4410313 | 7.55E-06 |
| AX-157118437 | 0.035676493 | 0.767959316 | 3 | 27518531 | 7.22E-07 |
| AX-157555425 | 0.017252772 | -0.1869 | 3 | 34919909 | 1.47E-05 |
| AX-157550821 | 0.012371837 | 0.144653265 | 5 | 2642885 | 5.40E-06 |
| AX-157372053 | 0 | 0 | 6 | 49361535 | 4.95E-08 |
| AX-157423495 | 0.020291586 | -0.43199 | 6 | 12326909 | 5.01E-06 |
| AX-157575428 | 0.010461285 | 0.027061533 | 6 | 49361503 |  |
| AX-157285878 | 0.065254919 | 1.638599511 | 8 | 4905525 | 7.23E-07 |
| AX-157302727 | 0.048581903 | 1.465768931 | 9 | 3271495 | 0.000101976 |
| AX-157337512 | 0.018241396 | -0.494939 | 9 | 40850058 | 1.93E-06 |
| AX-157400476 | 0.000625055 | 0.001954514 | 9 | 40907289 | 0.00011722 |
| AX-157352074 | 0.022829176 | 0.030356891 | 11 | 14879003 | 8.64E-06 |
| AX-157378148 | 0.03751999 | 0.014226627 | 11 | 32879122 | 2.41E-07 |
| AX-157435618 | 0.019618614 | 0.504840797 | 11 | 8189393 | 1.52E-06 |
| AX-116900743 | 0.010234706 | -0.0374 | 12 | 17318628 | 7.37E-08 |
| AX-116906637 | 0.015548903 | 0.508295959 | 12 | 9121409 | 6.75E-07 |
| AX-157052730 | 0.003108694 | -0.21318 | 12 | 14744106 | 9.75E-06 |
| AX-157241197 | 0.008355417 | -0.262268 | 12 | 1522694 | 0.000103968 |
| AX-157257849 | 0.000895406 | -0.0296 | 12 | 16317590 | 4.44E-10 |
| AX-157328322 | 0.006888084 | -0.15122 | 12 | 15566266 | 1.33E-05 |
| AX-157356820 | 0.001422549 | 0.041042844 | 12 | 2844288 | 1.78E-05 |
| AX-157393309 | 0.012081511 | 0.137759748 | 12 | 2812360 | 7.85E-05 |
| AX-157408960 | 0.014590867 | 0.319057004 | 12 | 16487770 | 9.13E-05 |
| AX-157452132 | 0.002884249 | -0.346988 | 12 | 16082217 | 2.67E-07 |
| AX-157193736 | 0.065326898 | -0.749701 | 13 | 30177239 | 3.67E-06 |
| AX-157278798 | 0.016530984 | 0.109648138 | 13 | 10297850 | 4.71E-07 |
| AX-157378954 | 0.034718014 | -0.808562 | 13 | 28263890 | 2.45E-06 |
| AX-157488363 | 0.024132924 | -0.80538 | 13 | 15296150 | 8.82E-06 |
| AX-157135021 | 0.152261488 | -0.88672 | 14 | 16998076 | 1.48E-05 |
| AX-157215482 | 0.018226405 | 0.073456536 | 15 | 2400557 | 4.91E-08 |
| AX-157230447 | 0.050425681 | 0.287731175 | 15 | 2353940 | 8.28E-07 |
| AX-157577407 | 0.035784334 | 0.235479176 | 15 | 1846762 | 6.02E-06 |
| AX-157070851 | 0.04919976 | -0.761113 | 16 | 2830814 | 4.69E-06 |
| AX-157095643 | 0.003810178 | 0.108493917 | 16 | 2945163 | 1.31E-07 |
| AX-157579184 | 0.058954812 | 0.67330976 | 16 | 27365719 | 6.16E-07 |
| AX-157108662 | 0.016268605 | 0.069210108 | 17 | 40105633 | 1.10E-06 |
| AX-157292334 | 0.016019232 | -0.191663 | 19 | 45405934 | 0.000125199 |

a, Refers to the specific identifier of the Quantitative Trait Nucleotide (QTN) or genetic marker associated with HSW. b, The average of the absolute SHAP values . It represents the global importance of the QTN, reflecting the magnitude of its contribution to the model's prediction regardless of direction. c, The average of the raw SHAP values. It indicates the average directional effect of the QTN on the trait (HSW); a positive value suggests a positive correlation, while a negative value suggests a negative correlation.

**Supplementary Table 8.** Simulated breeding results for an F_2_ population size of 200.

| Method-Ped | | | | |  | Method-Bulk | | | | |
| --- | --- | --- | --- | --- | --- | --- | --- | --- | --- | --- |
| Individual | GenoValue | NSA | SCP1 | SCP2 |  | Individual | GenoValue | NSA | SCP1 | SCP2 |
| 1 | 23.2348 | 29 | HN002 | HN017 |  | 1 | 18.7404 | 23 | HN002 | HN011 |
| 2 | 20.2963 | 24 | HN002 | HN051 |  | 2 | 18.3731 | 20 | HN002 | HN018 |
| 3 | 18.3806 | 18 | HN002 | HN069 |  | 3 | 22.6676 | 22 | HN002 | HN020 |
| 4 | 22.4136 | 23 | HN002 | HN070 |  | 4 | 20.0978 | 27 | HN002 | HN046 |
| 5 | 16.9928 | 19 | HN002 | HN109 |  | 5 | 22.5878 | 26 | HN002 | HN051 |
| 6 | 21.3036 | 24 | HN002 | HN114 |  | 6 | 22.0493 | 27 | HN002 | HN060 |
| 7 | 24.3663 | 23 | HN002 | HN117 |  | 7 | 19.6259 | 24 | HN002 | HN062 |
| 8 | 20.3181 | 25 | HN002 | HN136 |  | 8 | 25.0387 | 28 | HN002 | HN083 |
| 9 | 21.9576 | 24 | HN002 | HN136 |  | 9 | 21.9436 | 25 | HN002 | HN090 |
| 10 | 21.5963 | 21 | HN003 | HN007 |  | 10 | 19.6058 | 21 | HN002 | HN090 |
| 11 | 22.8392 | 26 | HN003 | HN009 |  | 11 | 21.5994 | 20 | HN002 | HN097 |
| 12 | 22.987 | 21 | HN003 | HN022 |  | 12 | 19.871 | 19 | HN002 | HN097 |
| 13 | 19.4742 | 22 | HN003 | HN024 |  | 13 | 20.9361 | 23 | HN002 | HN105 |
| 14 | 22.0784 | 24 | HN003 | HN052 |  | 14 | 24.8135 | 24 | HN002 | HN113 |
| 15 | 20.5513 | 21 | HN003 | HN067 |  | 15 | 22.1162 | 23 | HN002 | HN123 |
| 16 | 25.0892 | 30 | HN003 | HN105 |  | 16 | 22.8727 | 27 | HN002 | HN129 |
| 17 | 24.462 | 27 | HN003 | HN127 |  | 17 | 22.3494 | 20 | HN003 | HN007 |
| 18 | 19.5413 | 24 | HN003 | HN129 |  | 18 | 22.7413 | 26 | HN003 | HN009 |
| 19 | 21.9085 | 21 | HN003 | HN140 |  | 19 | 25.639 | 25 | HN003 | HN012 |
| 20 | 21.0266 | 21 | HN003 | HN149 |  | 20 | 18.4987 | 15 | HN003 | HN015 |
| 21 | 21.3036 | 19 | HN004 | HN014 |  | 21 | 22.9354 | 25 | HN003 | HN024 |
| 22 | 23.0314 | 20 | HN004 | HN016 |  | 22 | 19.6055 | 19 | HN003 | HN026 |
| 23 | 21.9833 | 24 | HN004 | HN020 |  | 23 | 17.1935 | 17 | HN003 | HN029 |
| 24 | 22.3811 | 24 | HN004 | HN020 |  | 24 | 19.8765 | 20 | HN003 | HN035 |
| 25 | 25.7573 | 28 | HN004 | HN024 |  | 25 | 22.4052 | 26 | HN003 | HN037 |
| 26 | 25.4503 | 24 | HN004 | HN043 |  | 26 | 21.4818 | 22 | HN003 | HN054 |
| 27 | 22.9395 | 21 | HN004 | HN045 |  | 27 | 23.3132 | 24 | HN003 | HN065 |
| 28 | 25.0414 | 25 | HN004 | HN060 |  | 28 | 19.1891 | 19 | HN003 | HN067 |
| 29 | 25.7776 | 26 | HN004 | HN069 |  | 29 | 22.3239 | 24 | HN003 | HN072 |
| 30 | 28.8828 | 30 | HN004 | HN083 |  | 30 | 21.9231 | 20 | HN003 | HN073 |
| 31 | 20.5379 | 18 | HN004 | HN085 |  | 31 | 19.0385 | 21 | HN003 | HN074 |
| 32 | 23.4938 | 21 | HN004 | HN097 |  | 32 | 21.0181 | 22 | HN003 | HN080 |
| 33 | 19.7668 | 18 | HN004 | HN098 |  | 33 | 22.0382 | 22 | HN003 | HN080 |
| 34 | 27.3806 | 26 | HN004 | HN116 |  | 34 | 21.0181 | 22 | HN003 | HN080 |
| 35 | 22.756 | 24 | HN004 | HN122 |  | 35 | 17.2429 | 19 | HN003 | HN086 |
| 36 | 18.4824 | 22 | HN004 | HN137 |  | 36 | 15.4912 | 19 | HN003 | HN087 |
| 37 | 18.5306 | 21 | HN005 | HN072 |  | 37 | 20.9711 | 24 | HN003 | HN090 |
| 38 | 20.8063 | 20 | HN006 | HN014 |  | 38 | 22.0709 | 16 | HN003 | HN097 |
| 39 | 17.9648 | 22 | HN006 | HN054 |  | 39 | 20.8402 | 20 | HN003 | HN101 |
| 40 | 20.8524 | 23 | HN006 | HN067 |  | 40 | 20.4442 | 22 | HN003 | HN110 |
| 41 | 22.6329 | 29 | HN006 | HN083 |  | 41 | 23.14 | 24 | HN003 | HN118 |
| 42 | 21.0105 | 24 | HN006 | HN125 |  | 42 | 22.5292 | 21 | HN003 | HN120 |
| 43 | 24.1524 | 27 | HN007 | HN017 |  | 43 | 23.4396 | 24 | HN003 | HN123 |
| 44 | 23.4791 | 23 | HN007 | HN033 |  | 44 | 18.6301 | 25 | HN003 | HN128 |
| 45 | 20.305 | 23 | HN007 | HN040 |  | 45 | 23.8349 | 29 | HN003 | HN130 |
| 46 | 26.1136 | 25 | HN007 | HN043 |  | 46 | 18.6636 | 22 | HN003 | HN147 |
| 47 | 22.0795 | 23 | HN007 | HN052 |  | 47 | 21.5653 | 20 | HN003 | HN148 |
| 48 | 21.2721 | 23 | HN007 | HN056 |  | 48 | 20.4175 | 23 | HN004 | HN006 |
| 49 | 22.6799 | 23 | HN007 | HN062 |  | 49 | 23.1011 | 23 | HN004 | HN008 |
| 50 | 25.8249 | 25 | HN007 | HN073 |  | 50 | 24.3259 | 25 | HN004 | HN009 |
| 51 | 23.0413 | 25 | HN007 | HN083 |  | 51 | 26.7453 | 27 | HN004 | HN012 |
| 52 | 19.4033 | 21 | HN007 | HN090 |  | 52 | 26.3656 | 26 | HN004 | HN016 |
| 53 | 20.6185 | 21 | HN007 | HN093 |  | 53 | 21.1397 | 22 | HN004 | HN018 |
| 54 | 22.8217 | 19 | HN007 | HN104 |  | 54 | 19.3106 | 19 | HN004 | HN018 |
| 55 | 19.4018 | 22 | HN007 | HN109 |  | 55 | 25.9652 | 23 | HN004 | HN022 |
| 56 | 22.1123 | 25 | HN007 | HN112 |  | 56 | 21.225 | 23 | HN004 | HN023 |
| 57 | 23.4192 | 24 | HN007 | HN116 |  | 57 | 23.4197 | 22 | HN004 | HN024 |
| 58 | 21.4817 | 21 | HN007 | HN119 |  | 58 | 23.5624 | 23 | HN004 | HN032 |
| 59 | 20.1351 | 20 | HN007 | HN119 |  | 59 | 19.1858 | 20 | HN004 | HN033 |
| 60 | 22.8102 | 23 | HN007 | HN120 |  | 60 | 21.7534 | 23 | HN004 | HN033 |
| 61 | 23.1241 | 23 | HN007 | HN129 |  | 61 | 28.8789 | 25 | HN004 | HN034 |
| 62 | 26.155 | 28 | HN007 | HN131 |  | 62 | 25.6525 | 23 | HN004 | HN034 |
| 63 | 18.5596 | 21 | HN007 | HN132 |  | 63 | 23.3191 | 21 | HN004 | HN035 |
| 64 | 16.8748 | 19 | HN007 | HN135 |  | 64 | 22.7681 | 22 | HN004 | HN041 |
| 65 | 23.356 | 25 | HN007 | HN143 |  | 65 | 21.1889 | 20 | HN004 | HN043 |
| 66 | 21.6061 | 25 | HN008 | HN016 |  | 66 | 25.3034 | 23 | HN004 | HN043 |
| 67 | 19.628 | 25 | HN008 | HN026 |  | 67 | 24.2593 | 26 | HN004 | HN051 |
| 68 | 17.6926 | 22 | HN008 | HN128 |  | 68 | 21.8669 | 23 | HN004 | HN051 |
| 69 | 19.8271 | 23 | HN008 | HN128 |  | 69 | 24.6517 | 28 | HN004 | HN054 |
| 70 | 18.8418 | 23 | HN009 | HN010 |  | 70 | 22.9868 | 25 | HN004 | HN054 |
| 71 | 22.0099 | 26 | HN009 | HN016 |  | 71 | 23.2724 | 23 | HN004 | HN057 |
| 72 | 21.1399 | 27 | HN009 | HN017 |  | 72 | 23.0602 | 23 | HN004 | HN058 |
| 73 | 19.9019 | 22 | HN009 | HN018 |  | 73 | 26.2304 | 27 | HN004 | HN064 |
| 74 | 24.9749 | 28 | HN009 | HN073 |  | 74 | 22.6159 | 25 | HN004 | HN065 |
| 75 | 25.1899 | 27 | HN009 | HN113 |  | 75 | 23.9921 | 25 | HN004 | HN069 |
| 76 | 21.4494 | 26 | HN009 | HN115 |  | 76 | 20.6032 | 23 | HN004 | HN074 |
| 77 | 26.1022 | 28 | HN009 | HN116 |  | 77 | 20.8213 | 20 | HN004 | HN076 |
| 78 | 26.1022 | 28 | HN009 | HN116 |  | 78 | 25.7753 | 24 | HN004 | HN082 |
| 79 | 23.705 | 28 | HN009 | HN127 |  | 79 | 19.8076 | 24 | HN004 | HN083 |
| 80 | 18.8785 | 22 | HN009 | HN139 |  | 80 | 23.5334 | 24 | HN004 | HN095 |
| 81 | 24.0429 | 28 | HN009 | HN140 |  | 81 | 20.995 | 23 | HN004 | HN101 |
| 82 | 21.5342 | 22 | HN010 | HN032 |  | 82 | 23.8426 | 21 | HN004 | HN105 |
| 83 | 21.3259 | 26 | HN010 | HN047 |  | 83 | 20.5658 | 20 | HN004 | HN111 |
| 84 | 16.1875 | 22 | HN010 | HN048 |  | 84 | 23.0013 | 21 | HN004 | HN111 |
| 85 | 22.4517 | 23 | HN010 | HN048 |  | 85 | 22.8266 | 22 | HN004 | HN112 |
| 86 | 23.4075 | 27 | HN010 | HN050 |  | 86 | 25.5458 | 25 | HN004 | HN123 |
| 87 | 23.4424 | 27 | HN010 | HN060 |  | 87 | 20.8985 | 25 | HN004 | HN128 |
| 88 | 23.0094 | 26 | HN010 | HN064 |  | 88 | 24.3813 | 23 | HN004 | HN130 |
| 89 | 20.2657 | 23 | HN010 | HN068 |  | 89 | 25.8801 | 27 | HN004 | HN133 |
| 90 | 16.566 | 24 | HN010 | HN072 |  | 90 | 23.0287 | 23 | HN004 | HN136 |
| 91 | 24.1793 | 24 | HN010 | HN073 |  | 91 | 21.6879 | 19 | HN004 | HN139 |
| 92 | 18.1951 | 23 | HN010 | HN075 |  | 92 | 21.1496 | 27 | HN004 | HN147 |
| 93 | 25.0788 | 27 | HN010 | HN112 |  | 93 | 20.0965 | 20 | HN004 | HN150 |
| 94 | 21.2745 | 25 | HN010 | HN115 |  | 94 | 21.1092 | 25 | HN005 | HN021 |
| 95 | 24.6729 | 27 | HN010 | HN116 |  | 95 | 24.0102 | 25 | HN005 | HN026 |
| 96 | 22.3168 | 27 | HN011 | HN020 |  | 96 | 20.9881 | 24 | HN005 | HN035 |
| 97 | 19.3958 | 25 | HN011 | HN021 |  | 97 | 20.7451 | 25 | HN005 | HN061 |
| 98 | 19.8634 | 25 | HN011 | HN023 |  | 98 | 21.2464 | 22 | HN005 | HN064 |
| 99 | 24.2128 | 25 | HN011 | HN026 |  | 99 | 18.8887 | 22 | HN005 | HN067 |
| 100 | 19.165 | 24 | HN011 | HN077 |  | 100 | 13.471 | 20 | HN005 | HN071 |
| 101 | 21.871 | 26 | HN011 | HN082 |  | 101 | 23.109 | 24 | HN005 | HN082 |
| 102 | 27.687 | 29 | HN011 | HN082 |  | 102 | 20.8602 | 25 | HN005 | HN103 |
| 103 | 21.1474 | 28 | HN011 | HN129 |  | 103 | 21.1486 | 23 | HN005 | HN113 |
| 104 | 22.3833 | 28 | HN012 | HN017 |  | 104 | 21.1594 | 24 | HN005 | HN117 |
| 105 | 22.7432 | 25 | HN012 | HN026 |  | 105 | 22.054 | 24 | HN005 | HN129 |
| 106 | 21.8221 | 27 | HN012 | HN050 |  | 106 | 19.8978 | 26 | HN006 | HN009 |
| 107 | 23.6661 | 28 | HN012 | HN057 |  | 107 | 19.9658 | 24 | HN006 | HN016 |
| 108 | 26.6087 | 31 | HN012 | HN064 |  | 108 | 17.3601 | 21 | HN006 | HN030 |
| 109 | 26.895 | 29 | HN012 | HN073 |  | 109 | 21.8947 | 28 | HN006 | HN037 |
| 110 | 24.3868 | 28 | HN012 | HN075 |  | 110 | 20.7389 | 25 | HN006 | HN050 |
| 111 | 18.2456 | 24 | HN012 | HN081 |  | 111 | 19.6378 | 25 | HN006 | HN053 |
| 112 | 28.8892 | 30 | HN012 | HN082 |  | 112 | 19.675 | 23 | HN006 | HN053 |
| 113 | 27.055 | 33 | HN012 | HN129 |  | 113 | 19.5191 | 25 | HN006 | HN058 |
| 114 | 25.6525 | 33 | HN012 | HN129 |  | 114 | 18.533 | 20 | HN006 | HN061 |
| 115 | 21.6276 | 27 | HN012 | HN132 |  | 115 | 19.551 | 24 | HN006 | HN064 |
| 116 | 22.9553 | 25 | HN012 | HN138 |  | 116 | 16.3175 | 20 | HN006 | HN080 |
| 117 | 21.6179 | 26 | HN012 | HN149 |  | 117 | 23.0042 | 24 | HN006 | HN082 |
| 118 | 26.2759 | 23 | HN014 | HN016 |  | 118 | 15.2813 | 22 | HN006 | HN086 |
| 119 | 23.1856 | 21 | HN014 | HN020 |  | 119 | 16.1519 | 22 | HN006 | HN091 |
| 120 | 19.8873 | 18 | HN014 | HN026 |  | 120 | 22.0825 | 24 | HN006 | HN093 |
| 121 | 25.1059 | 22 | HN014 | HN034 |  | 121 | 19.6324 | 26 | HN006 | HN096 |
| 122 | 21.3273 | 20 | HN014 | HN041 |  | 122 | 14.1628 | 18 | HN006 | HN100 |
| 123 | 22.3962 | 20 | HN014 | HN048 |  | 123 | 20.3047 | 25 | HN006 | HN107 |
| 124 | 23.4993 | 24 | HN014 | HN050 |  | 124 | 20.9874 | 26 | HN006 | HN130 |
| 125 | 22.0554 | 22 | HN014 | HN069 |  | 125 | 23.5187 | 27 | HN006 | HN131 |
| 126 | 16.5316 | 18 | HN014 | HN077 |  | 126 | 22.4841 | 26 | HN007 | HN010 |
| 127 | 19.7121 | 23 | HN014 | HN079 |  | 127 | 23.8508 | 26 | HN007 | HN012 |
| 128 | 25.2176 | 22 | HN014 | HN083 |  | 128 | 23.7678 | 23 | HN007 | HN015 |
| 129 | 23.7144 | 23 | HN014 | HN093 |  | 129 | 24.2224 | 25 | HN007 | HN019 |
| 130 | 20.5058 | 20 | HN014 | HN095 |  | 130 | 23.6807 | 25 | HN007 | HN020 |
| 131 | 22.3144 | 25 | HN014 | HN105 |  | 131 | 22.2758 | 23 | HN007 | HN020 |
| 132 | 26.1 | 29 | HN014 | HN105 |  | 132 | 25.3569 | 26 | HN007 | HN020 |
| 133 | 20.1441 | 20 | HN014 | HN116 |  | 133 | 27.0442 | 24 | HN007 | HN022 |
| 134 | 25.2769 | 27 | HN015 | HN073 |  | 134 | 26.0582 | 24 | HN007 | HN022 |
| 135 | 22.4631 | 23 | HN015 | HN082 |  | 135 | 25.7427 | 28 | HN007 | HN024 |
| 136 | 23.0597 | 25 | HN015 | HN083 |  | 136 | 21.4661 | 27 | HN007 | HN024 |
| 137 | 19.5029 | 20 | HN015 | HN090 |  | 137 | 22.3837 | 25 | HN007 | HN024 |
| 138 | 16.4789 | 20 | HN015 | HN116 |  | 138 | 25.1476 | 24 | HN007 | HN026 |
| 139 | 21.4087 | 22 | HN015 | HN118 |  | 139 | 22.9844 | 22 | HN007 | HN030 |
| 140 | 22.2585 | 22 | HN015 | HN118 |  | 140 | 24.5814 | 24 | HN007 | HN037 |
| 141 | 21.0381 | 23 | HN015 | HN141 |  | 141 | 23.8975 | 24 | HN007 | HN038 |
| 142 | 25.8715 | 29 | HN016 | HN022 |  | 142 | 24.1863 | 26 | HN007 | HN039 |
| 143 | 24.4302 | 28 | HN016 | HN038 |  | 143 | 24.8613 | 25 | HN007 | HN041 |
| 144 | 17.9049 | 23 | HN016 | HN039 |  | 144 | 23.5202 | 25 | HN007 | HN050 |
| 145 | 21.9504 | 26 | HN016 | HN040 |  | 145 | 27.9919 | 28 | HN007 | HN050 |
| 146 | 24.979 | 28 | HN016 | HN043 |  | 146 | 23.4209 | 25 | HN007 | HN067 |
| 147 | 18.8809 | 22 | HN016 | HN058 |  | 147 | 22.5858 | 24 | HN007 | HN068 |
| 148 | 21.7165 | 25 | HN016 | HN060 |  | 148 | 21.223 | 20 | HN007 | HN072 |
| 149 | 22.2637 | 25 | HN016 | HN061 |  | 149 | 22.9512 | 25 | HN007 | HN072 |
| 150 | 21.7414 | 22 | HN016 | HN084 |  | 150 | 24.7795 | 26 | HN007 | HN075 |
| 151 | 24.3839 | 29 | HN016 | HN103 |  | 151 | 22.4856 | 22 | HN007 | HN080 |
| 152 | 27.2412 | 27 | HN016 | HN113 |  | 152 | 23.2099 | 24 | HN007 | HN081 |
| 153 | 22.5302 | 27 | HN016 | HN126 |  | 153 | 20.5087 | 21 | HN007 | HN091 |
| 154 | 22.41 | 25 | HN016 | HN130 |  | 154 | 24.9981 | 28 | HN007 | HN094 |
| 155 | 19.5178 | 22 | HN016 | HN143 |  | 155 | 22.2275 | 25 | HN007 | HN094 |
| 156 | 21.7756 | 24 | HN017 | HN025 |  | 156 | 22.613 | 22 | HN007 | HN095 |
| 157 | 24.3765 | 28 | HN017 | HN034 |  | 157 | 19.1911 | 16 | HN007 | HN097 |
| 158 | 20.3366 | 24 | HN017 | HN041 |  | 158 | 23.701 | 26 | HN007 | HN101 |
| 159 | 22.4389 | 28 | HN017 | HN055 |  | 159 | 21.0362 | 27 | HN007 | HN105 |
| 160 | 22.0829 | 27 | HN017 | HN064 |  | 160 | 20.0151 | 20 | HN007 | HN106 |
| 161 | 23.8475 | 25 | HN017 | HN068 |  | 161 | 21.6822 | 22 | HN007 | HN106 |
| 162 | 21.6334 | 25 | HN017 | HN068 |  | 162 | 26.4742 | 26 | HN007 | HN113 |
| 163 | 22.3124 | 28 | HN017 | HN073 |  | 163 | 26.3139 | 24 | HN007 | HN113 |
| 164 | 24.4508 | 27 | HN017 | HN082 |  | 164 | 17.6527 | 24 | HN007 | HN115 |
| 165 | 23.3513 | 30 | HN017 | HN103 |  | 165 | 23.4661 | 22 | HN007 | HN118 |
| 166 | 21.9441 | 25 | HN017 | HN106 |  | 166 | 19.7779 | 22 | HN007 | HN120 |
| 167 | 24.3242 | 30 | HN017 | HN125 |  | 167 | 23.1326 | 23 | HN007 | HN123 |
| 168 | 20.9398 | 27 | HN017 | HN128 |  | 168 | 23.6254 | 23 | HN007 | HN124 |
| 169 | 22.6576 | 29 | HN017 | HN132 |  | 169 | 24.3025 | 26 | HN007 | HN125 |
| 170 | 24.2624 | 23 | HN018 | HN022 |  | 170 | 23.1354 | 25 | HN007 | HN126 |
| 171 | 19.5046 | 22 | HN018 | HN027 |  | 171 | 21.2155 | 22 | HN007 | HN133 |
| 172 | 19.5259 | 21 | HN018 | HN063 |  | 172 | 21.024 | 23 | HN007 | HN134 |
| 173 | 22.6882 | 27 | HN018 | HN065 |  | 173 | 21.6456 | 20 | HN007 | HN139 |
| 174 | 22.4256 | 24 | HN018 | HN068 |  | 174 | 22.7676 | 24 | HN008 | HN020 |
| 175 | 18.2704 | 18 | HN018 | HN077 |  | 175 | 22.4574 | 21 | HN008 | HN022 |
| 176 | 25.2366 | 31 | HN018 | HN083 |  | 176 | 23.181 | 25 | HN008 | HN053 |
| 177 | 26.2184 | 28 | HN018 | HN129 |  | 177 | 18.1194 | 22 | HN008 | HN059 |
| 178 | 19.4761 | 21 | HN018 | HN140 |  | 178 | 21.7717 | 26 | HN008 | HN062 |
| 179 | 27.7095 | 30 | HN019 | HN022 |  | 179 | 15.6332 | 23 | HN008 | HN063 |
| 180 | 22.4017 | 22 | HN019 | HN058 |  | 180 | 22.2023 | 25 | HN008 | HN067 |
| 181 | 19.0988 | 26 | HN019 | HN125 |  | 181 | 17.2293 | 22 | HN008 | HN081 |
| 182 | 20.9268 | 26 | HN019 | HN147 |  | 182 | 21.1097 | 23 | HN008 | HN101 |
| 183 | 24.3096 | 25 | HN020 | HN032 |  | 183 | 18.1509 | 22 | HN008 | HN110 |
| 184 | 21.5657 | 21 | HN020 | HN046 |  | 184 | 26.1428 | 28 | HN008 | HN116 |
| 185 | 22.5188 | 24 | HN020 | HN049 |  | 185 | 21.8096 | 26 | HN008 | HN128 |
| 186 | 20.2973 | 23 | HN020 | HN054 |  | 186 | 23.9264 | 29 | HN008 | HN131 |
| 187 | 23.5243 | 25 | HN020 | HN056 |  | 187 | 18.8709 | 20 | HN008 | HN145 |
| 188 | 24.0499 | 23 | HN020 | HN058 |  | 188 | 21.3439 | 25 | HN009 | HN016 |
| 189 | 23.5751 | 26 | HN020 | HN059 |  | 189 | 21.7507 | 26 | HN009 | HN019 |
| 190 | 24.1785 | 28 | HN020 | HN059 |  | 190 | 23.0131 | 26 | HN009 | HN020 |
| 191 | 21.838 | 25 | HN020 | HN060 |  | 191 | 20.2808 | 25 | HN009 | HN021 |
| 192 | 22.7074 | 22 | HN020 | HN061 |  | 192 | 21.8337 | 29 | HN009 | HN024 |
| 193 | 24.6697 | 26 | HN020 | HN061 |  | 193 | 20.6949 | 25 | HN009 | HN025 |
| 194 | 25.3821 | 28 | HN020 | HN065 |  | 194 | 22.9038 | 28 | HN009 | HN041 |
| 195 | 23.7376 | 22 | HN020 | HN068 |  | 195 | 23.4022 | 28 | HN009 | HN051 |
| 196 | 23.4851 | 23 | HN020 | HN074 |  | 196 | 20.6307 | 25 | HN009 | HN052 |
| 197 | 26.64 | 26 | HN020 | HN082 |  | 197 | 19.257 | 22 | HN009 | HN059 |
| 198 | 25.3873 | 29 | HN020 | HN083 |  | 198 | 20.0395 | 24 | HN009 | HN064 |
| 199 | 20.6597 | 20 | HN020 | HN088 |  | 199 | 22.0318 | 27 | HN009 | HN064 |
| 200 | 26.5325 | 29 | HN020 | HN096 |  | 200 | 20.034 | 24 | HN009 | HN067 |
| 201 | 22.6508 | 26 | HN020 | HN101 |  | 201 | 23.6115 | 29 | HN009 | HN069 |
| 202 | 27.8474 | 33 | HN020 | HN105 |  | 202 | 19.7541 | 24 | HN009 | HN086 |
| 203 | 27.8474 | 33 | HN020 | HN105 |  | 203 | 22.232 | 27 | HN009 | HN103 |
| 204 | 25.2697 | 27 | HN020 | HN116 |  | 204 | 21.2931 | 29 | HN009 | HN105 |
| 205 | 20.4432 | 23 | HN020 | HN121 |  | 205 | 21.4649 | 26 | HN009 | HN109 |
| 206 | 23.3288 | 25 | HN020 | HN124 |  | 206 | 19.1865 | 23 | HN009 | HN110 |
| 207 | 26.6177 | 31 | HN020 | HN129 |  | 207 | 22.8135 | 25 | HN009 | HN113 |
| 208 | 23.1185 | 26 | HN020 | HN131 |  | 208 | 22.9503 | 27 | HN009 | HN131 |
| 209 | 23.1342 | 24 | HN020 | HN136 |  | 209 | 21.0841 | 25 | HN009 | HN136 |
| 210 | 24.0684 | 28 | HN020 | HN137 |  | 210 | 21.4775 | 29 | HN009 | HN147 |
| 211 | 23.6585 | 25 | HN020 | HN143 |  | 211 | 22.2834 | 22 | HN009 | HN148 |
| 212 | 20.9964 | 21 | HN021 | HN032 |  | 212 | 22.1325 | 26 | HN010 | HN019 |
| 213 | 22.69 | 23 | HN021 | HN041 |  | 213 | 23.0448 | 24 | HN010 | HN020 |
| 214 | 24.8252 | 27 | HN021 | HN057 |  | 214 | 20.5817 | 26 | HN010 | HN036 |
| 215 | 23.0395 | 26 | HN021 | HN069 |  | 215 | 21.6134 | 26 | HN010 | HN037 |
| 216 | 24.8847 | 24 | HN021 | HN082 |  | 216 | 19.6074 | 26 | HN010 | HN046 |
| 217 | 22.7368 | 26 | HN021 | HN103 |  | 217 | 20.9957 | 24 | HN010 | HN049 |
| 218 | 21.0988 | 21 | HN021 | HN108 |  | 218 | 24.22 | 29 | HN010 | HN050 |
| 219 | 20.9846 | 25 | HN021 | HN115 |  | 219 | 24.3116 | 27 | HN010 | HN050 |
| 220 | 23.4815 | 25 | HN021 | HN130 |  | 220 | 21.782 | 24 | HN010 | HN051 |
| 221 | 25.069 | 26 | HN022 | HN023 |  | 221 | 20.1535 | 27 | HN010 | HN053 |
| 222 | 27.6479 | 27 | HN022 | HN025 |  | 222 | 21.906 | 27 | HN010 | HN057 |
| 223 | 23.2678 | 22 | HN022 | HN025 |  | 223 | 23.3501 | 25 | HN010 | HN058 |
| 224 | 28.4128 | 31 | HN022 | HN034 |  | 224 | 20.4103 | 26 | HN010 | HN065 |
| 225 | 27.7427 | 30 | HN022 | HN046 |  | 225 | 24.192 | 27 | HN010 | HN068 |
| 226 | 23.1311 | 21 | HN022 | HN049 |  | 226 | 25.1054 | 28 | HN010 | HN069 |
| 227 | 27.954 | 31 | HN022 | HN050 |  | 227 | 26.1859 | 27 | HN010 | HN073 |
| 228 | 27.9501 | 30 | HN022 | HN050 |  | 228 | 19.9195 | 22 | HN010 | HN077 |
| 229 | 25.6416 | 30 | HN022 | HN051 |  | 229 | 20.4601 | 21 | HN010 | HN080 |
| 230 | 22.3801 | 24 | HN022 | HN060 |  | 230 | 23.2739 | 26 | HN010 | HN080 |
| 231 | 24.7598 | 24 | HN022 | HN068 |  | 231 | 19.5733 | 20 | HN010 | HN080 |
| 232 | 24.0493 | 22 | HN022 | HN071 |  | 232 | 21.9687 | 25 | HN010 | HN090 |
| 233 | 25.2947 | 24 | HN022 | HN073 |  | 233 | 22.6084 | 25 | HN010 | HN090 |
| 234 | 23.7253 | 23 | HN022 | HN073 |  | 234 | 23.5464 | 29 | HN010 | HN096 |
| 235 | 21.0375 | 24 | HN022 | HN074 |  | 235 | 21.8218 | 27 | HN010 | HN105 |
| 236 | 24.8993 | 23 | HN022 | HN082 |  | 236 | 23.2769 | 26 | HN010 | HN109 |
| 237 | 24.7905 | 23 | HN022 | HN088 |  | 237 | 21.7196 | 22 | HN010 | HN117 |
| 238 | 24.6227 | 24 | HN022 | HN093 |  | 238 | 21.9381 | 28 | HN010 | HN130 |
| 239 | 26.9473 | 27 | HN022 | HN096 |  | 239 | 21.1152 | 28 | HN010 | HN131 |
| 240 | 20.3295 | 23 | HN022 | HN096 |  | 240 | 20.0702 | 20 | HN010 | HN132 |
| 241 | 24.8809 | 18 | HN022 | HN097 |  | 241 | 21.3546 | 24 | HN010 | HN132 |
| 242 | 26.8213 | 26 | HN022 | HN102 |  | 242 | 21.037 | 23 | HN010 | HN143 |
| 243 | 26.7311 | 25 | HN022 | HN117 |  | 243 | 20.1952 | 25 | HN010 | HN150 |
| 244 | 25.0994 | 23 | HN022 | HN122 |  | 244 | 24.0081 | 26 | HN011 | HN020 |
| 245 | 25.0243 | 24 | HN022 | HN122 |  | 245 | 25.2265 | 27 | HN011 | HN020 |
| 246 | 19.4562 | 20 | HN022 | HN123 |  | 246 | 23.4494 | 25 | HN011 | HN026 |
| 247 | 21.6429 | 25 | HN022 | HN133 |  | 247 | 20.7634 | 23 | HN011 | HN033 |
| 248 | 22.2055 | 24 | HN022 | HN134 |  | 248 | 15.5406 | 25 | HN011 | HN039 |
| 249 | 22.9322 | 25 | HN022 | HN136 |  | 249 | 26.3928 | 29 | HN011 | HN043 |
| 250 | 26.5253 | 27 | HN022 | HN137 |  | 250 | 20.3984 | 25 | HN011 | HN060 |
| 251 | 27.2048 | 27 | HN022 | HN147 |  | 251 | 21.5296 | 25 | HN011 | HN073 |
| 252 | 21.6089 | 29 | HN023 | HN055 |  | 252 | 25.6774 | 26 | HN011 | HN073 |
| 253 | 21.767 | 25 | HN023 | HN073 |  | 253 | 18.9365 | 22 | HN011 | HN077 |
| 254 | 17.6843 | 23 | HN024 | HN032 |  | 254 | 27.257 | 31 | HN011 | HN082 |
| 255 | 22.3708 | 29 | HN024 | HN039 |  | 255 | 16.6192 | 25 | HN011 | HN083 |
| 256 | 23.6513 | 29 | HN024 | HN055 |  | 256 | 25.7413 | 32 | HN011 | HN083 |
| 257 | 18.6682 | 24 | HN024 | HN056 |  | 257 | 17.0268 | 26 | HN011 | HN083 |
| 258 | 18.6951 | 25 | HN024 | HN070 |  | 258 | 21.613 | 27 | HN011 | HN103 |
| 259 | 22.2987 | 26 | HN024 | HN098 |  | 259 | 20.83 | 26 | HN011 | HN107 |
| 260 | 21.4902 | 25 | HN024 | HN113 |  | 260 | 19.4893 | 25 | HN011 | HN115 |
| 261 | 21.5023 | 28 | HN024 | HN137 |  | 261 | 23.7924 | 27 | HN011 | HN116 |
| 262 | 21.361 | 22 | HN025 | HN026 |  | 262 | 20.918 | 21 | HN011 | HN117 |
| 263 | 23.0884 | 27 | HN025 | HN055 |  | 263 | 22.945 | 28 | HN011 | HN143 |
| 264 | 21.6858 | 26 | HN025 | HN090 |  | 264 | 20.6442 | 21 | HN011 | HN148 |
| 265 | 23.8409 | 27 | HN025 | HN090 |  | 265 | 24.566 | 27 | HN012 | HN021 |
| 266 | 24.5169 | 25 | HN025 | HN094 |  | 266 | 25.1878 | 27 | HN012 | HN021 |
| 267 | 17.685 | 20 | HN025 | HN110 |  | 267 | 26.1287 | 27 | HN012 | HN035 |
| 268 | 23.8293 | 25 | HN025 | HN113 |  | 268 | 23.7108 | 28 | HN012 | HN039 |
| 269 | 20.4716 | 23 | HN025 | HN116 |  | 269 | 21.696 | 27 | HN012 | HN041 |
| 270 | 16.2145 | 25 | HN025 | HN127 |  | 270 | 23.0879 | 27 | HN012 | HN045 |
| 271 | 27.5511 | 27 | HN026 | HN043 |  | 271 | 18.9574 | 25 | HN012 | HN047 |
| 272 | 24.2779 | 26 | HN026 | HN043 |  | 272 | 23.0361 | 27 | HN012 | HN057 |
| 273 | 24.2779 | 26 | HN026 | HN043 |  | 273 | 22.5738 | 25 | HN012 | HN061 |
| 274 | 23.81 | 21 | HN026 | HN043 |  | 274 | 21.0747 | 26 | HN012 | HN062 |
| 275 | 24.762 | 28 | HN026 | HN045 |  | 275 | 22.618 | 29 | HN012 | HN065 |
| 276 | 24.762 | 28 | HN026 | HN045 |  | 276 | 24.7669 | 31 | HN012 | HN067 |
| 277 | 22.6408 | 23 | HN026 | HN057 |  | 277 | 23.1826 | 26 | HN012 | HN075 |
| 278 | 23.3488 | 25 | HN026 | HN059 |  | 278 | 23.8055 | 27 | HN012 | HN082 |
| 279 | 21.9768 | 21 | HN026 | HN061 |  | 279 | 20.1951 | 25 | HN012 | HN098 |
| 280 | 22.9551 | 22 | HN026 | HN067 |  | 280 | 23.5975 | 27 | HN012 | HN098 |
| 281 | 22.4686 | 24 | HN026 | HN069 |  | 281 | 24.8763 | 29 | HN012 | HN105 |
| 282 | 27.5727 | 31 | HN026 | HN072 |  | 282 | 21.7128 | 23 | HN012 | HN113 |
| 283 | 27.0252 | 29 | HN026 | HN080 |  | 283 | 24.6035 | 30 | HN012 | HN118 |
| 284 | 28.0608 | 29 | HN026 | HN082 |  | 284 | 21.2224 | 24 | HN012 | HN121 |
| 285 | 25.7216 | 28 | HN026 | HN090 |  | 285 | 20.0475 | 24 | HN012 | HN122 |
| 286 | 23.4437 | 22 | HN026 | HN106 |  | 286 | 20.7529 | 26 | HN012 | HN122 |
| 287 | 25.9698 | 29 | HN026 | HN109 |  | 287 | 20.9582 | 26 | HN012 | HN124 |
| 288 | 24.1619 | 25 | HN026 | HN120 |  | 288 | 25.532 | 31 | HN012 | HN128 |
| 289 | 23.6307 | 24 | HN026 | HN121 |  | 289 | 23.6956 | 31 | HN012 | HN131 |
| 290 | 23.0175 | 25 | HN026 | HN124 |  | 290 | 20.1904 | 24 | HN012 | HN132 |
| 291 | 24.7355 | 27 | HN026 | HN127 |  | 291 | 24.0612 | 29 | HN012 | HN140 |
| 292 | 24.8816 | 27 | HN026 | HN146 |  | 292 | 22.0587 | 24 | HN012 | HN143 |
| 293 | 20.6818 | 23 | HN027 | HN042 |  | 293 | 22.5795 | 27 | HN012 | HN147 |
| 294 | 22.7577 | 25 | HN027 | HN054 |  | 294 | 22.4778 | 24 | HN012 | HN148 |
| 295 | 23.0783 | 27 | HN027 | HN083 |  | 295 | 23.0581 | 23 | HN014 | HN015 |
| 296 | 24.6786 | 28 | HN027 | HN096 |  | 296 | 22.4207 | 22 | HN014 | HN018 |
| 297 | 23.9078 | 21 | HN027 | HN097 |  | 297 | 23.4834 | 23 | HN014 | HN019 |
| 298 | 23.5437 | 19 | HN027 | HN097 |  | 298 | 22.5204 | 23 | HN014 | HN020 |
| 299 | 23.4035 | 25 | HN027 | HN130 |  | 299 | 22.0024 | 19 | HN014 | HN021 |
| 300 | 21.6858 | 23 | HN027 | HN130 |  | 300 | 23.5158 | 22 | HN014 | HN022 |
| 301 | 24.1809 | 25 | HN029 | HN034 |  | 301 | 22.8993 | 20 | HN014 | HN036 |
| 302 | 18.4156 | 20 | HN029 | HN116 |  | 302 | 27.5451 | 26 | HN014 | HN039 |
| 303 | 24.0407 | 27 | HN029 | HN127 |  | 303 | 23.4471 | 19 | HN014 | HN043 |
| 304 | 23.5237 | 27 | HN029 | HN131 |  | 304 | 18.1703 | 16 | HN014 | HN049 |
| 305 | 25.3251 | 26 | HN030 | HN034 |  | 305 | 18.9708 | 17 | HN014 | HN049 |
| 306 | 21.8471 | 21 | HN030 | HN061 |  | 306 | 23.844 | 24 | HN014 | HN051 |
| 307 | 17.8493 | 18 | HN030 | HN095 |  | 307 | 22.0197 | 22 | HN014 | HN052 |
| 308 | 19.6338 | 24 | HN030 | HN096 |  | 308 | 23.7326 | 21 | HN014 | HN066 |
| 309 | 17.4672 | 20 | HN030 | HN123 |  | 309 | 24.29 | 25 | HN014 | HN068 |
| 310 | 17.9875 | 19 | HN030 | HN124 |  | 310 | 21.7691 | 23 | HN014 | HN072 |
| 311 | 26.361 | 27 | HN030 | HN131 |  | 311 | 26.0788 | 26 | HN014 | HN073 |
| 312 | 18.8738 | 18 | HN030 | HN140 |  | 312 | 24.6167 | 25 | HN014 | HN079 |
| 313 | 19.1788 | 22 | HN031 | HN037 |  | 313 | 22.8859 | 22 | HN014 | HN081 |
| 314 | 21.5029 | 21 | HN031 | HN113 |  | 314 | 22.8547 | 22 | HN014 | HN085 |
| 315 | 24.6947 | 27 | HN032 | HN096 |  | 315 | 25.8633 | 24 | HN014 | HN090 |
| 316 | 21.7793 | 23 | HN032 | HN110 |  | 316 | 20.9921 | 23 | HN014 | HN098 |
| 317 | 20.9599 | 24 | HN032 | HN127 |  | 317 | 21.7802 | 23 | HN014 | HN101 |
| 318 | 24.3298 | 25 | HN032 | HN129 |  | 318 | 27.0867 | 25 | HN014 | HN105 |
| 319 | 23.0857 | 22 | HN032 | HN148 |  | 319 | 23.5392 | 25 | HN014 | HN105 |
| 320 | 21.2584 | 25 | HN033 | HN064 |  | 320 | 26.1049 | 24 | HN014 | HN111 |
| 321 | 17.1072 | 20 | HN033 | HN085 |  | 321 | 26.709 | 26 | HN014 | HN116 |
| 322 | 20.6983 | 24 | HN033 | HN108 |  | 322 | 23.3997 | 25 | HN014 | HN125 |
| 323 | 21.1206 | 21 | HN033 | HN116 |  | 323 | 20.7446 | 21 | HN014 | HN130 |
| 324 | 21.4009 | 23 | HN033 | HN117 |  | 324 | 21.0554 | 25 | HN014 | HN136 |
| 325 | 20.5977 | 24 | HN033 | HN144 |  | 325 | 22.6784 | 22 | HN014 | HN137 |
| 326 | 24.2173 | 25 | HN034 | HN035 |  | 326 | 26.5199 | 25 | HN014 | HN138 |
| 327 | 23.6864 | 24 | HN034 | HN042 |  | 327 | 23.31 | 23 | HN014 | HN138 |
| 328 | 22.5246 | 24 | HN034 | HN043 |  | 328 | 21.2268 | 21 | HN014 | HN140 |
| 329 | 24.9485 | 26 | HN034 | HN050 |  | 329 | 22.7667 | 24 | HN014 | HN143 |
| 330 | 26.0779 | 28 | HN034 | HN056 |  | 330 | 19.1245 | 22 | HN014 | HN143 |
| 331 | 23.7986 | 27 | HN034 | HN059 |  | 331 | 19.792 | 24 | HN014 | HN147 |
| 332 | 25.2656 | 29 | HN034 | HN060 |  | 332 | 19.184 | 18 | HN014 | HN150 |
| 333 | 24.053 | 27 | HN034 | HN060 |  | 333 | 24.7979 | 22 | HN015 | HN022 |
| 334 | 22.3913 | 25 | HN034 | HN060 |  | 334 | 18.9844 | 23 | HN015 | HN047 |
| 335 | 24.2267 | 28 | HN034 | HN061 |  | 335 | 22.21 | 25 | HN015 | HN050 |
| 336 | 23.0137 | 27 | HN034 | HN064 |  | 336 | 19.2564 | 25 | HN015 | HN054 |
| 337 | 26.2191 | 31 | HN034 | HN065 |  | 337 | 19.5393 | 23 | HN015 | HN056 |
| 338 | 21.4177 | 24 | HN034 | HN074 |  | 338 | 20.3436 | 20 | HN015 | HN061 |
| 339 | 25.8757 | 30 | HN034 | HN083 |  | 339 | 21.6401 | 24 | HN015 | HN067 |
| 340 | 23.7134 | 25 | HN034 | HN087 |  | 340 | 20.5587 | 24 | HN015 | HN080 |
| 341 | 26.4518 | 28 | HN034 | HN090 |  | 341 | 18.9398 | 21 | HN015 | HN081 |
| 342 | 25.2774 | 28 | HN034 | HN109 |  | 342 | 19.6479 | 22 | HN015 | HN083 |
| 343 | 25.2309 | 27 | HN034 | HN111 |  | 343 | 20.0204 | 20 | HN015 | HN084 |
| 344 | 30.1417 | 29 | HN034 | HN116 |  | 344 | 20.6359 | 24 | HN015 | HN090 |
| 345 | 23.0667 | 25 | HN034 | HN119 |  | 345 | 19.8287 | 18 | HN015 | HN098 |
| 346 | 25.0718 | 26 | HN034 | HN121 |  | 346 | 18.5699 | 21 | HN015 | HN126 |
| 347 | 24.9704 | 28 | HN034 | HN123 |  | 347 | 17.0744 | 21 | HN015 | HN147 |
| 348 | 24.3529 | 28 | HN034 | HN128 |  | 348 | 22.5096 | 25 | HN016 | HN019 |
| 349 | 23.7126 | 27 | HN034 | HN129 |  | 349 | 23.629 | 27 | HN016 | HN021 |
| 350 | 22.6435 | 28 | HN034 | HN130 |  | 350 | 20.8067 | 24 | HN016 | HN023 |
| 351 | 24.5117 | 28 | HN034 | HN130 |  | 351 | 22.4567 | 28 | HN016 | HN023 |
| 352 | 21.2134 | 28 | HN034 | HN131 |  | 352 | 24.0741 | 27 | HN016 | HN035 |
| 353 | 23.3699 | 27 | HN034 | HN136 |  | 353 | 21.542 | 24 | HN016 | HN038 |
| 354 | 24.089 | 24 | HN034 | HN139 |  | 354 | 22.4979 | 26 | HN016 | HN046 |
| 355 | 23.3551 | 24 | HN034 | HN149 |  | 355 | 24.1049 | 25 | HN016 | HN047 |
| 356 | 21.947 | 21 | HN035 | HN087 |  | 356 | 23.8599 | 26 | HN016 | HN049 |
| 357 | 21.9929 | 24 | HN035 | HN108 |  | 357 | 25.5174 | 28 | HN016 | HN050 |
| 358 | 22.7772 | 24 | HN035 | HN112 |  | 358 | 25.3836 | 30 | HN016 | HN054 |
| 359 | 20.9156 | 27 | HN035 | HN129 |  | 359 | 22.7257 | 25 | HN016 | HN067 |
| 360 | 20.2095 | 22 | HN035 | HN140 |  | 360 | 19.5977 | 20 | HN016 | HN070 |
| 361 | 22.6402 | 22 | HN035 | HN144 |  | 361 | 24.4225 | 26 | HN016 | HN082 |
| 362 | 19.4954 | 23 | HN036 | HN054 |  | 362 | 20.9831 | 25 | HN016 | HN083 |
| 363 | 20.3728 | 23 | HN036 | HN064 |  | 363 | 23.4105 | 23 | HN016 | HN084 |
| 364 | 18.5076 | 25 | HN037 | HN039 |  | 364 | 23.4105 | 23 | HN016 | HN084 |
| 365 | 19.7781 | 25 | HN037 | HN056 |  | 365 | 22.5173 | 23 | HN016 | HN085 |
| 366 | 25.16 | 29 | HN037 | HN079 |  | 366 | 23.5119 | 26 | HN016 | HN085 |
| 367 | 26.5885 | 28 | HN037 | HN082 |  | 367 | 22.557 | 25 | HN016 | HN090 |
| 368 | 20.5353 | 27 | HN037 | HN125 |  | 368 | 23.1702 | 22 | HN016 | HN093 |
| 369 | 22.3417 | 29 | HN037 | HN127 |  | 369 | 25.5731 | 30 | HN016 | HN096 |
| 370 | 22.0058 | 29 | HN037 | HN127 |  | 370 | 17.8086 | 24 | HN016 | HN102 |
| 371 | 18.4131 | 24 | HN037 | HN128 |  | 371 | 22.0862 | 25 | HN016 | HN102 |
| 372 | 21.9859 | 29 | HN037 | HN129 |  | 372 | 23.0063 | 22 | HN016 | HN104 |
| 373 | 20.3024 | 26 | HN038 | HN057 |  | 373 | 23.7191 | 25 | HN016 | HN104 |
| 374 | 19.5806 | 25 | HN038 | HN114 |  | 374 | 22.3544 | 26 | HN016 | HN105 |
| 375 | 18.576 | 23 | HN038 | HN118 |  | 375 | 26.9417 | 27 | HN016 | HN106 |
| 376 | 16.5118 | 19 | HN038 | HN143 |  | 376 | 22.7611 | 23 | HN016 | HN108 |
| 377 | 21.4003 | 25 | HN040 | HN082 |  | 377 | 22.136 | 25 | HN016 | HN110 |
| 378 | 22.1877 | 24 | HN040 | HN118 |  | 378 | 20.2911 | 25 | HN016 | HN112 |
| 379 | 18.2636 | 18 | HN040 | HN140 |  | 379 | 25.7547 | 27 | HN016 | HN113 |
| 380 | 19.8835 | 25 | HN041 | HN063 |  | 380 | 22.0107 | 22 | HN016 | HN116 |
| 381 | 21.0872 | 25 | HN041 | HN103 |  | 381 | 20.5246 | 22 | HN016 | HN117 |
| 382 | 22.4663 | 28 | HN041 | HN103 |  | 382 | 21.1647 | 24 | HN016 | HN128 |
| 383 | 20.9153 | 23 | HN041 | HN110 |  | 383 | 25.744 | 30 | HN016 | HN130 |
| 384 | 16.6348 | 18 | HN041 | HN118 |  | 384 | 24.2413 | 29 | HN016 | HN131 |
| 385 | 22.7509 | 27 | HN041 | HN125 |  | 385 | 21.7169 | 25 | HN016 | HN135 |
| 386 | 24.6025 | 28 | HN042 | HN082 |  | 386 | 24.5703 | 28 | HN016 | HN137 |
| 387 | 24.5986 | 27 | HN042 | HN082 |  | 387 | 18.224 | 20 | HN016 | HN139 |
| 388 | 23.7101 | 24 | HN042 | HN113 |  | 388 | 24.0247 | 28 | HN016 | HN140 |
| 389 | 19.7611 | 22 | HN042 | HN118 |  | 389 | 25.2685 | 25 | HN016 | HN140 |
| 390 | 23.1501 | 28 | HN042 | HN130 |  | 390 | 19.7318 | 23 | HN016 | HN143 |
| 391 | 22.5063 | 27 | HN043 | HN051 |  | 391 | 23.673 | 26 | HN016 | HN145 |
| 392 | 24.827 | 23 | HN043 | HN064 |  | 392 | 22.0772 | 28 | HN017 | HN024 |
| 393 | 24.3549 | 24 | HN043 | HN082 |  | 393 | 20.2827 | 23 | HN017 | HN032 |
| 394 | 25.6537 | 30 | HN043 | HN096 |  | 394 | 23.5892 | 28 | HN017 | HN034 |
| 395 | 20.3127 | 18 | HN043 | HN104 |  | 395 | 20.9591 | 25 | HN017 | HN039 |
| 396 | 23.4292 | 24 | HN043 | HN112 |  | 396 | 21.4552 | 27 | HN017 | HN052 |
| 397 | 23.9428 | 23 | HN043 | HN116 |  | 397 | 18.9971 | 25 | HN017 | HN059 |
| 398 | 26.6256 | 29 | HN043 | HN129 |  | 398 | 20.2186 | 24 | HN017 | HN064 |
| 399 | 24.5431 | 27 | HN043 | HN130 |  | 399 | 20.3949 | 26 | HN017 | HN074 |
| 400 | 24.4712 | 25 | HN043 | HN137 |  | 400 | 21.2244 | 27 | HN017 | HN075 |
| 401 | 22.9587 | 22 | HN043 | HN149 |  | 401 | 22.3282 | 22 | HN017 | HN076 |
| 402 | 16.8325 | 26 | HN044 | HN138 |  | 402 | 21.3549 | 27 | HN017 | HN079 |
| 403 | 20.152 | 27 | HN045 | HN047 |  | 403 | 21.4101 | 24 | HN017 | HN084 |
| 404 | 22.6813 | 27 | HN045 | HN063 |  | 404 | 23.3626 | 28 | HN017 | HN097 |
| 405 | 21.7153 | 26 | HN046 | HN082 |  | 405 | 19.8969 | 26 | HN017 | HN098 |
| 406 | 20.1285 | 26 | HN046 | HN117 |  | 406 | 24.4476 | 27 | HN017 | HN104 |
| 407 | 18.3968 | 22 | HN046 | HN122 |  | 407 | 23.7929 | 27 | HN017 | HN104 |
| 408 | 19.9379 | 22 | HN046 | HN136 |  | 408 | 21.4938 | 21 | HN017 | HN106 |
| 409 | 20.0113 | 26 | HN047 | HN079 |  | 409 | 22.0591 | 26 | HN017 | HN108 |
| 410 | 21.7896 | 28 | HN047 | HN105 |  | 410 | 23.1764 | 28 | HN017 | HN109 |
| 411 | 19.6171 | 24 | HN047 | HN116 |  | 411 | 23.2117 | 28 | HN017 | HN112 |
| 412 | 19.9473 | 24 | HN047 | HN117 |  | 412 | 21.4109 | 24 | HN017 | HN114 |
| 413 | 14.7106 | 21 | HN047 | HN139 |  | 413 | 21.8595 | 27 | HN017 | HN121 |
| 414 | 24.7927 | 25 | HN048 | HN073 |  | 414 | 20.6189 | 27 | HN017 | HN130 |
| 415 | 20.8699 | 20 | HN048 | HN073 |  | 415 | 24.1208 | 29 | HN017 | HN130 |
| 416 | 22.7297 | 23 | HN048 | HN075 |  | 416 | 21.7181 | 28 | HN017 | HN131 |
| 417 | 22.1601 | 27 | HN048 | HN083 |  | 417 | 21.5388 | 27 | HN017 | HN132 |
| 418 | 19.2513 | 20 | HN048 | HN093 |  | 418 | 20.3655 | 24 | HN017 | HN133 |
| 419 | 24.0661 | 24 | HN048 | HN113 |  | 419 | 22.0259 | 27 | HN017 | HN133 |
| 420 | 19.6098 | 21 | HN048 | HN114 |  | 420 | 19.3199 | 20 | HN017 | HN142 |
| 421 | 24.2682 | 22 | HN048 | HN116 |  | 421 | 23.509 | 29 | HN017 | HN147 |
| 422 | 21.6166 | 24 | HN049 | HN061 |  | 422 | 18.6162 | 24 | HN017 | HN149 |
| 423 | 22.769 | 25 | HN049 | HN064 |  | 423 | 21.4786 | 24 | HN017 | HN149 |
| 424 | 24.8085 | 29 | HN049 | HN090 |  | 424 | 25.0808 | 27 | HN018 | HN020 |
| 425 | 25.1799 | 28 | HN049 | HN105 |  | 425 | 24.2422 | 24 | HN018 | HN026 |
| 426 | 18.3615 | 19 | HN049 | HN132 |  | 426 | 24.1895 | 26 | HN018 | HN034 |
| 427 | 25.0915 | 27 | HN050 | HN064 |  | 427 | 24.7204 | 24 | HN018 | HN039 |
| 428 | 19.9617 | 24 | HN050 | HN064 |  | 428 | 22.4967 | 25 | HN018 | HN050 |
| 429 | 22.6879 | 25 | HN050 | HN080 |  | 429 | 21.5138 | 24 | HN018 | HN051 |
| 430 | 20.7623 | 28 | HN050 | HN083 |  | 430 | 16.7785 | 21 | HN018 | HN054 |
| 431 | 25.6205 | 27 | HN050 | HN090 |  | 431 | 22.5719 | 23 | HN018 | HN057 |
| 432 | 25.5556 | 26 | HN050 | HN106 |  | 432 | 20.9286 | 23 | HN018 | HN072 |
| 433 | 26.0243 | 28 | HN050 | HN106 |  | 433 | 20.6197 | 23 | HN018 | HN072 |
| 434 | 23.5368 | 27 | HN050 | HN108 |  | 434 | 28.3373 | 29 | HN018 | HN082 |
| 435 | 17.0982 | 21 | HN050 | HN122 |  | 435 | 21.0033 | 22 | HN018 | HN090 |
| 436 | 21.2984 | 29 | HN050 | HN127 |  | 436 | 19.4761 | 21 | HN018 | HN093 |
| 437 | 19.3911 | 26 | HN050 | HN128 |  | 437 | 20.1967 | 21 | HN018 | HN095 |
| 438 | 28.7885 | 32 | HN050 | HN129 |  | 438 | 25.8177 | 28 | HN018 | HN096 |
| 439 | 25.8789 | 28 | HN050 | HN129 |  | 439 | 24.8702 | 25 | HN018 | HN105 |
| 440 | 23.2832 | 27 | HN050 | HN130 |  | 440 | 22.7733 | 24 | HN018 | HN107 |
| 441 | 20.7717 | 25 | HN050 | HN141 |  | 441 | 21.6721 | 23 | HN018 | HN108 |
| 442 | 19.6712 | 24 | HN051 | HN055 |  | 442 | 20.6884 | 24 | HN018 | HN109 |
| 443 | 24.5094 | 29 | HN051 | HN067 |  | 443 | 20.9769 | 21 | HN018 | HN110 |
| 444 | 25.4332 | 26 | HN051 | HN073 |  | 444 | 26.2211 | 27 | HN018 | HN113 |
| 445 | 27.6025 | 32 | HN051 | HN082 |  | 445 | 19.7185 | 22 | HN018 | HN115 |
| 446 | 26.0883 | 29 | HN051 | HN082 |  | 446 | 24.084 | 23 | HN018 | HN116 |
| 447 | 23.3399 | 29 | HN051 | HN082 |  | 447 | 17.744 | 18 | HN018 | HN119 |
| 448 | 25.0503 | 30 | HN051 | HN083 |  | 448 | 19.4009 | 21 | HN018 | HN124 |
| 449 | 24.8159 | 30 | HN051 | HN096 |  | 449 | 22.6644 | 25 | HN018 | HN126 |
| 450 | 22.0385 | 28 | HN051 | HN107 |  | 450 | 27.957 | 30 | HN018 | HN131 |
| 451 | 23.0594 | 29 | HN051 | HN112 |  | 451 | 23.4946 | 22 | HN018 | HN132 |
| 452 | 20.7984 | 26 | HN051 | HN136 |  | 452 | 25.2021 | 26 | HN018 | HN132 |
| 453 | 22.1518 | 28 | HN051 | HN148 |  | 453 | 23.3774 | 22 | HN018 | HN138 |
| 454 | 21.1738 | 28 | HN052 | HN068 |  | 454 | 26.3935 | 26 | HN019 | HN022 |
| 455 | 19.1583 | 23 | HN052 | HN090 |  | 455 | 18.3139 | 20 | HN019 | HN029 |
| 456 | 16.3015 | 19 | HN052 | HN095 |  | 456 | 17.5495 | 23 | HN019 | HN034 |
| 457 | 19.1444 | 18 | HN052 | HN104 |  | 457 | 13.5035 | 21 | HN019 | HN042 |
| 458 | 23.9336 | 24 | HN052 | HN116 |  | 458 | 21.8932 | 25 | HN019 | HN051 |
| 459 | 20.4102 | 24 | HN052 | HN132 |  | 459 | 21.7693 | 25 | HN019 | HN051 |
| 460 | 21.5307 | 24 | HN052 | HN140 |  | 460 | 18.7594 | 23 | HN019 | HN054 |
| 461 | 18.454 | 23 | HN052 | HN145 |  | 461 | 22.6661 | 24 | HN019 | HN064 |
| 462 | 22.6476 | 27 | HN053 | HN064 |  | 462 | 21.2397 | 27 | HN019 | HN068 |
| 463 | 23.1613 | 28 | HN053 | HN067 |  | 463 | 23.8517 | 26 | HN019 | HN068 |
| 464 | 24.6729 | 31 | HN053 | HN073 |  | 464 | 22.5977 | 27 | HN019 | HN073 |
| 465 | 24.7454 | 23 | HN053 | HN106 |  | 465 | 19.4555 | 22 | HN019 | HN075 |
| 466 | 18.7196 | 25 | HN054 | HN057 |  | 466 | 22.9283 | 27 | HN019 | HN083 |
| 467 | 23.3527 | 29 | HN054 | HN060 |  | 467 | 23.297 | 29 | HN019 | HN094 |
| 468 | 24.1595 | 23 | HN054 | HN116 |  | 468 | 19.613 | 26 | HN019 | HN098 |
| 469 | 24.4463 | 28 | HN054 | HN129 |  | 469 | 21.0754 | 25 | HN019 | HN110 |
| 470 | 21.5578 | 26 | HN054 | HN138 |  | 470 | 22.3486 | 23 | HN019 | HN111 |
| 471 | 21.1158 | 28 | HN055 | HN057 |  | 471 | 18.6818 | 24 | HN019 | HN115 |
| 472 | 22.7262 | 26 | HN055 | HN073 |  | 472 | 22.9334 | 25 | HN019 | HN116 |
| 473 | 24.3077 | 30 | HN055 | HN083 |  | 473 | 18.3425 | 23 | HN019 | HN123 |
| 474 | 23.404 | 28 | HN055 | HN083 |  | 474 | 20.4107 | 24 | HN019 | HN125 |
| 475 | 19.68 | 26 | HN055 | HN103 |  | 475 | 22.2247 | 26 | HN019 | HN131 |
| 476 | 24.2564 | 27 | HN055 | HN116 |  | 476 | 25.0342 | 26 | HN020 | HN022 |
| 477 | 14.7679 | 20 | HN055 | HN122 |  | 477 | 23.8372 | 22 | HN020 | HN022 |
| 478 | 26.2254 | 30 | HN055 | HN125 |  | 478 | 24.8286 | 26 | HN020 | HN032 |
| 479 | 26.3682 | 31 | HN055 | HN125 |  | 479 | 23.7228 | 28 | HN020 | HN033 |
| 480 | 21.3882 | 27 | HN055 | HN128 |  | 480 | 23.349 | 27 | HN020 | HN033 |
| 481 | 22.1443 | 27 | HN055 | HN129 |  | 481 | 22.3543 | 26 | HN020 | HN040 |
| 482 | 22.8997 | 27 | HN055 | HN129 |  | 482 | 23.8426 | 25 | HN020 | HN044 |
| 483 | 18.1151 | 23 | HN056 | HN079 |  | 483 | 22.2698 | 25 | HN020 | HN044 |
| 484 | 26.5654 | 27 | HN056 | HN082 |  | 484 | 21.9135 | 24 | HN020 | HN045 |
| 485 | 21.9441 | 26 | HN056 | HN087 |  | 485 | 22.2189 | 29 | HN020 | HN047 |
| 486 | 22.4247 | 27 | HN056 | HN115 |  | 486 | 25.4379 | 27 | HN020 | HN050 |
| 487 | 21.4411 | 25 | HN056 | HN129 |  | 487 | 20.8256 | 23 | HN020 | HN053 |
| 488 | 22.2502 | 26 | HN057 | HN058 |  | 488 | 20.8774 | 26 | HN020 | HN054 |
| 489 | 20.2645 | 23 | HN057 | HN058 |  | 489 | 23.1379 | 25 | HN020 | HN061 |
| 490 | 21.4596 | 22 | HN057 | HN068 |  | 490 | 21.9275 | 28 | HN020 | HN063 |
| 491 | 20.8859 | 27 | HN057 | HN079 |  | 491 | 22.4976 | 24 | HN020 | HN071 |
| 492 | 23.7031 | 31 | HN057 | HN083 |  | 492 | 22.4222 | 28 | HN020 | HN072 |
| 493 | 22.8524 | 28 | HN057 | HN090 |  | 493 | 20.3729 | 23 | HN020 | HN074 |
| 494 | 24.2409 | 25 | HN057 | HN116 |  | 494 | 21.9713 | 26 | HN020 | HN075 |
| 495 | 21.1488 | 26 | HN057 | HN118 |  | 495 | 20.7312 | 25 | HN020 | HN075 |
| 496 | 23.1195 | 28 | HN057 | HN131 |  | 496 | 20.8664 | 22 | HN020 | HN088 |
| 497 | 20.6959 | 21 | HN058 | HN064 |  | 497 | 25.8809 | 27 | HN020 | HN090 |
| 498 | 19.5553 | 22 | HN058 | HN075 |  | 498 | 22.8242 | 28 | HN020 | HN090 |
| 499 | 23.6332 | 25 | HN058 | HN080 |  | 499 | 22.8651 | 25 | HN020 | HN093 |
| 500 | 21.3808 | 22 | HN058 | HN088 |  | 500 | 23.6885 | 23 | HN020 | HN097 |
| 501 | 21.8221 | 23 | HN058 | HN114 |  | 501 | 17.7676 | 19 | HN020 | HN100 |
| 502 | 18.9299 | 21 | HN058 | HN122 |  | 502 | 16.5798 | 21 | HN020 | HN102 |
| 503 | 23.339 | 25 | HN058 | HN132 |  | 503 | 27.5618 | 27 | HN020 | HN104 |
| 504 | 19.4463 | 24 | HN058 | HN141 |  | 504 | 27.5618 | 27 | HN020 | HN104 |
| 505 | 21.9841 | 24 | HN058 | HN143 |  | 505 | 22.6836 | 26 | HN020 | HN108 |
| 506 | 19.7234 | 25 | HN059 | HN060 |  | 506 | 21.3971 | 25 | HN020 | HN109 |
| 507 | 18.2776 | 25 | HN059 | HN103 |  | 507 | 22.9147 | 24 | HN020 | HN110 |
| 508 | 23.9833 | 30 | HN060 | HN068 |  | 508 | 23.0827 | 26 | HN020 | HN110 |
| 509 | 20.2289 | 23 | HN060 | HN070 |  | 509 | 22.0241 | 25 | HN020 | HN112 |
| 510 | 26.2915 | 29 | HN060 | HN073 |  | 510 | 24.6044 | 23 | HN020 | HN118 |
| 511 | 20.885 | 25 | HN060 | HN079 |  | 511 | 23.8797 | 23 | HN020 | HN118 |
| 512 | 20.5985 | 22 | HN060 | HN080 |  | 512 | 21.7417 | 26 | HN020 | HN119 |
| 513 | 23.9074 | 28 | HN060 | HN096 |  | 513 | 22.3504 | 23 | HN020 | HN122 |
| 514 | 24.3955 | 24 | HN060 | HN097 |  | 514 | 22.0519 | 25 | HN020 | HN128 |
| 515 | 22.6012 | 28 | HN060 | HN105 |  | 515 | 22.1214 | 26 | HN020 | HN131 |
| 516 | 23.7522 | 27 | HN060 | HN115 |  | 516 | 24.7975 | 27 | HN020 | HN131 |
| 517 | 22.8639 | 28 | HN060 | HN119 |  | 517 | 23.5297 | 26 | HN020 | HN136 |
| 518 | 22.1959 | 26 | HN060 | HN122 |  | 518 | 25.6212 | 30 | HN020 | HN137 |
| 519 | 19.6489 | 25 | HN060 | HN126 |  | 519 | 22.6029 | 24 | HN020 | HN140 |
| 520 | 17.1554 | 24 | HN060 | HN138 |  | 520 | 24.3157 | 26 | HN020 | HN140 |
| 521 | 21.4291 | 23 | HN060 | HN148 |  | 521 | 26.093 | 27 | HN020 | HN141 |
| 522 | 24.4031 | 27 | HN061 | HN070 |  | 522 | 23.4925 | 26 | HN020 | HN144 |
| 523 | 22.7223 | 23 | HN061 | HN073 |  | 523 | 24.1526 | 28 | HN020 | HN146 |
| 524 | 22.118 | 24 | HN061 | HN083 |  | 524 | 21.3894 | 22 | HN020 | HN150 |
| 525 | 23.9133 | 26 | HN061 | HN083 |  | 525 | 20.3415 | 26 | HN021 | HN033 |
| 526 | 23.0066 | 24 | HN061 | HN101 |  | 526 | 24.7756 | 24 | HN021 | HN033 |
| 527 | 25.4113 | 23 | HN061 | HN106 |  | 527 | 23.5401 | 28 | HN021 | HN034 |
| 528 | 20.4872 | 24 | HN061 | HN109 |  | 528 | 25.2276 | 28 | HN021 | HN034 |
| 529 | 24.1664 | 24 | HN061 | HN111 |  | 529 | 21.1625 | 23 | HN021 | HN050 |
| 530 | 24.6423 | 24 | HN061 | HN113 |  | 530 | 23.2285 | 24 | HN021 | HN055 |
| 531 | 20.6583 | 21 | HN061 | HN114 |  | 531 | 23.6937 | 28 | HN021 | HN059 |
| 532 | 21.3073 | 24 | HN061 | HN125 |  | 532 | 23.5243 | 26 | HN021 | HN068 |
| 533 | 20.0968 | 24 | HN061 | HN136 |  | 533 | 21.2743 | 20 | HN021 | HN070 |
| 534 | 22.236 | 24 | HN061 | HN140 |  | 534 | 26.4241 | 24 | HN021 | HN073 |
| 535 | 15.4606 | 20 | HN062 | HN064 |  | 535 | 26.8129 | 28 | HN021 | HN073 |
| 536 | 17.8417 | 18 | HN062 | HN070 |  | 536 | 22.5952 | 24 | HN021 | HN074 |
| 537 | 26.1015 | 28 | HN062 | HN073 |  | 537 | 23.4835 | 27 | HN021 | HN079 |
| 538 | 21.1587 | 24 | HN062 | HN112 |  | 538 | 29.0998 | 32 | HN021 | HN083 |
| 539 | 22.5692 | 23 | HN062 | HN148 |  | 539 | 20.5001 | 21 | HN021 | HN085 |
| 540 | 18.3169 | 24 | HN063 | HN068 |  | 540 | 22.7312 | 25 | HN021 | HN087 |
| 541 | 22.4485 | 25 | HN063 | HN080 |  | 541 | 22.3847 | 23 | HN021 | HN093 |
| 542 | 21.5188 | 26 | HN063 | HN103 |  | 542 | 27.0606 | 30 | HN021 | HN105 |
| 543 | 19.6944 | 22 | HN064 | HN067 |  | 543 | 24.4688 | 23 | HN021 | HN106 |
| 544 | 21.5124 | 25 | HN064 | HN079 |  | 544 | 18.4689 | 19 | HN021 | HN107 |
| 545 | 18.3917 | 20 | HN064 | HN093 |  | 545 | 22.8009 | 24 | HN021 | HN110 |
| 546 | 21.6724 | 26 | HN064 | HN107 |  | 546 | 25.1473 | 24 | HN021 | HN112 |
| 547 | 24.5871 | 24 | HN064 | HN116 |  | 547 | 25.3748 | 28 | HN021 | HN112 |
| 548 | 26.2572 | 30 | HN064 | HN129 |  | 548 | 23.794 | 24 | HN021 | HN113 |
| 549 | 23.1462 | 25 | HN064 | HN129 |  | 549 | 22.5058 | 25 | HN021 | HN122 |
| 550 | 23.8475 | 25 | HN064 | HN130 |  | 550 | 24.9769 | 27 | HN021 | HN125 |
| 551 | 22.909 | 21 | HN064 | HN140 |  | 551 | 21.4403 | 23 | HN021 | HN126 |
| 552 | 24.4978 | 27 | HN064 | HN141 |  | 552 | 21.9656 | 29 | HN021 | HN130 |
| 553 | 21.9401 | 26 | HN064 | HN143 |  | 553 | 22.5467 | 28 | HN021 | HN137 |
| 554 | 24.247 | 28 | HN064 | HN147 |  | 554 | 22.5467 | 28 | HN021 | HN137 |
| 555 | 22.0481 | 24 | HN065 | HN066 |  | 555 | 15.3388 | 20 | HN021 | HN139 |
| 556 | 21.1668 | 27 | HN065 | HN067 |  | 556 | 20.6339 | 23 | HN021 | HN146 |
| 557 | 23.3469 | 30 | HN065 | HN068 |  | 557 | 21.7078 | 24 | HN022 | HN025 |
| 558 | 21.1717 | 27 | HN065 | HN071 |  | 558 | 25.4076 | 27 | HN022 | HN026 |
| 559 | 23.2942 | 31 | HN065 | HN090 |  | 559 | 20.6994 | 20 | HN022 | HN029 |
| 560 | 26.1741 | 29 | HN065 | HN096 |  | 560 | 26.9915 | 28 | HN022 | HN032 |
| 561 | 23.3181 | 26 | HN065 | HN115 |  | 561 | 22.2863 | 24 | HN022 | HN033 |
| 562 | 18.8523 | 24 | HN065 | HN118 |  | 562 | 27.107 | 30 | HN022 | HN034 |
| 563 | 26.6103 | 31 | HN065 | HN129 |  | 563 | 25.1419 | 23 | HN022 | HN036 |
| 564 | 22.6101 | 28 | HN065 | HN130 |  | 564 | 24.4491 | 25 | HN022 | HN037 |
| 565 | 21.3668 | 23 | HN066 | HN067 |  | 565 | 26.1042 | 26 | HN022 | HN041 |
| 566 | 23.502 | 26 | HN066 | HN068 |  | 566 | 23.6447 | 23 | HN022 | HN048 |
| 567 | 21.661 | 25 | HN066 | HN079 |  | 567 | 20.9201 | 26 | HN022 | HN053 |
| 568 | 18.06 | 25 | HN066 | HN079 |  | 568 | 24.5873 | 28 | HN022 | HN054 |
| 569 | 25.3277 | 29 | HN066 | HN129 |  | 569 | 26.1484 | 28 | HN022 | HN060 |
| 570 | 16.8345 | 21 | HN066 | HN137 |  | 570 | 27.1861 | 31 | HN022 | HN060 |
| 571 | 22.8126 | 26 | HN067 | HN068 |  | 571 | 25.9894 | 24 | HN022 | HN061 |
| 572 | 26.1388 | 27 | HN067 | HN073 |  | 572 | 25.9894 | 24 | HN022 | HN061 |
| 573 | 22.6094 | 26 | HN067 | HN118 |  | 573 | 30.2239 | 31 | HN022 | HN065 |
| 574 | 19.7489 | 22 | HN067 | HN149 |  | 574 | 22.9445 | 24 | HN022 | HN068 |
| 575 | 23.8147 | 29 | HN068 | HN081 |  | 575 | 26.0699 | 28 | HN022 | HN068 |
| 576 | 20.6775 | 23 | HN068 | HN090 |  | 576 | 22.0312 | 21 | HN022 | HN072 |
| 577 | 16.7769 | 19 | HN068 | HN100 |  | 577 | 23.9923 | 24 | HN022 | HN075 |
| 578 | 23.2129 | 25 | HN068 | HN101 |  | 578 | 22.6911 | 22 | HN022 | HN077 |
| 579 | 21.5338 | 22 | HN068 | HN106 |  | 579 | 23.699 | 23 | HN022 | HN077 |
| 580 | 25.808 | 28 | HN068 | HN113 |  | 580 | 24.322 | 25 | HN022 | HN082 |
| 581 | 24.5327 | 27 | HN068 | HN113 |  | 581 | 26.0027 | 26 | HN022 | HN083 |
| 582 | 25.4526 | 26 | HN068 | HN113 |  | 582 | 21.3499 | 29 | HN022 | HN090 |
| 583 | 21.6007 | 24 | HN068 | HN117 |  | 583 | 23.0118 | 20 | HN022 | HN092 |
| 584 | 25.4386 | 27 | HN068 | HN118 |  | 584 | 23.6054 | 26 | HN022 | HN094 |
| 585 | 23.2355 | 28 | HN068 | HN125 |  | 585 | 24.0715 | 26 | HN022 | HN094 |
| 586 | 22.1938 | 28 | HN068 | HN131 |  | 586 | 26.3198 | 31 | HN022 | HN096 |
| 587 | 27.7084 | 31 | HN068 | HN138 |  | 587 | 26.6793 | 23 | HN022 | HN097 |
| 588 | 25.3331 | 33 | HN069 | HN083 |  | 588 | 23.479 | 22 | HN022 | HN101 |
| 589 | 23.4653 | 27 | HN069 | HN090 |  | 589 | 24.6894 | 25 | HN022 | HN101 |
| 590 | 26.8316 | 30 | HN069 | HN090 |  | 590 | 26.5716 | 32 | HN022 | HN103 |
| 591 | 23.5876 | 24 | HN069 | HN113 |  | 591 | 26.9918 | 23 | HN022 | HN104 |
| 592 | 26.3482 | 27 | HN069 | HN116 |  | 592 | 28.9868 | 31 | HN022 | HN105 |
| 593 | 21.9367 | 23 | HN069 | HN123 |  | 593 | 23.1286 | 21 | HN022 | HN106 |
| 594 | 26.4487 | 32 | HN069 | HN129 |  | 594 | 20.6623 | 21 | HN022 | HN110 |
| 595 | 17.7606 | 21 | HN069 | HN149 |  | 595 | 23.6316 | 23 | HN022 | HN113 |
| 596 | 21.0631 | 21 | HN070 | HN097 |  | 596 | 16.6227 | 19 | HN022 | HN114 |
| 597 | 15.4394 | 24 | HN070 | HN098 |  | 597 | 26.4163 | 22 | HN022 | HN117 |
| 598 | 22.555 | 21 | HN070 | HN116 |  | 598 | 25.0638 | 27 | HN022 | HN118 |
| 599 | 25.5935 | 31 | HN070 | HN125 |  | 599 | 25.8633 | 25 | HN022 | HN121 |
| 600 | 19.3685 | 23 | HN071 | HN077 |  | 600 | 24.1349 | 23 | HN022 | HN121 |
| 601 | 17.8336 | 24 | HN071 | HN109 |  | 601 | 24.2395 | 22 | HN022 | HN122 |
| 602 | 18.4193 | 21 | HN071 | HN122 |  | 602 | 27.4818 | 30 | HN022 | HN129 |
| 603 | 23.6577 | 28 | HN071 | HN130 |  | 603 | 25.5637 | 30 | HN022 | HN130 |
| 604 | 22.9509 | 25 | HN072 | HN087 |  | 604 | 26.5775 | 28 | HN022 | HN130 |
| 605 | 20.4246 | 24 | HN072 | HN091 |  | 605 | 26.5449 | 26 | HN022 | HN130 |
| 606 | 24.2981 | 26 | HN072 | HN094 |  | 606 | 27.0526 | 29 | HN022 | HN130 |
| 607 | 25.1548 | 27 | HN072 | HN105 |  | 607 | 22.3102 | 21 | HN022 | HN134 |
| 608 | 23.5547 | 26 | HN072 | HN115 |  | 608 | 23.9354 | 22 | HN022 | HN137 |
| 609 | 21.4234 | 22 | HN072 | HN116 |  | 609 | 26.0226 | 21 | HN022 | HN139 |
| 610 | 18.5401 | 20 | HN072 | HN132 |  | 610 | 24.7644 | 22 | HN022 | HN142 |
| 611 | 16.7549 | 19 | HN072 | HN146 |  | 611 | 25.6193 | 25 | HN022 | HN142 |
| 612 | 28.8696 | 28 | HN073 | HN082 |  | 612 | 25.692 | 30 | HN022 | HN143 |
| 613 | 25.0759 | 25 | HN073 | HN082 |  | 613 | 24.94 | 24 | HN022 | HN145 |
| 614 | 23.6624 | 28 | HN073 | HN094 |  | 614 | 23.1578 | 21 | HN022 | HN145 |
| 615 | 26.8814 | 30 | HN073 | HN105 |  | 615 | 23.069 | 29 | HN022 | HN147 |
| 616 | 22.2731 | 25 | HN073 | HN108 |  | 616 | 25.381 | 27 | HN022 | HN147 |
| 617 | 22.5996 | 24 | HN073 | HN110 |  | 617 | 23.5555 | 22 | HN022 | HN148 |
| 618 | 24.1952 | 25 | HN073 | HN110 |  | 618 | 23.7712 | 26 | HN022 | HN150 |
| 619 | 19.5164 | 25 | HN073 | HN110 |  | 619 | 18.8916 | 21 | HN023 | HN025 |
| 620 | 23.0583 | 26 | HN073 | HN118 |  | 620 | 15.6781 | 20 | HN023 | HN063 |
| 621 | 27.5579 | 30 | HN073 | HN131 |  | 621 | 19.5633 | 22 | HN023 | HN064 |
| 622 | 24.213 | 27 | HN073 | HN137 |  | 622 | 18.1665 | 21 | HN023 | HN069 |
| 623 | 23.4943 | 25 | HN073 | HN139 |  | 623 | 20.9043 | 26 | HN023 | HN079 |
| 624 | 22.187 | 28 | HN073 | HN147 |  | 624 | 15.1839 | 16 | HN023 | HN092 |
| 625 | 25.658 | 28 | HN073 | HN148 |  | 625 | 23.1425 | 28 | HN023 | HN096 |
| 626 | 23.5565 | 24 | HN073 | HN149 |  | 626 | 19.2447 | 21 | HN023 | HN101 |
| 627 | 24.3619 | 26 | HN073 | HN149 |  | 627 | 22.2124 | 26 | HN023 | HN105 |
| 628 | 20.4948 | 26 | HN074 | HN083 |  | 628 | 18.9857 | 26 | HN023 | HN109 |
| 629 | 18.6516 | 21 | HN074 | HN087 |  | 629 | 19.9804 | 22 | HN023 | HN111 |
| 630 | 18.0959 | 24 | HN074 | HN096 |  | 630 | 17.8718 | 20 | HN023 | HN140 |
| 631 | 19.3254 | 23 | HN074 | HN098 |  | 631 | 20.8788 | 23 | HN023 | HN148 |
| 632 | 22.6602 | 25 | HN074 | HN148 |  | 632 | 26.0399 | 30 | HN024 | HN027 |
| 633 | 23.5412 | 26 | HN075 | HN080 |  | 633 | 25.3853 | 27 | HN024 | HN034 |
| 634 | 25.5989 | 31 | HN075 | HN083 |  | 634 | 19.1799 | 25 | HN024 | HN036 |
| 635 | 21.3746 | 25 | HN075 | HN091 |  | 635 | 20.9834 | 22 | HN024 | HN049 |
| 636 | 19.6251 | 22 | HN075 | HN117 |  | 636 | 22.2869 | 26 | HN024 | HN053 |
| 637 | 22.5439 | 23 | HN075 | HN117 |  | 637 | 25.9698 | 30 | HN024 | HN096 |
| 638 | 22.1502 | 26 | HN075 | HN124 |  | 638 | 18.0115 | 20 | HN024 | HN106 |
| 639 | 25.1959 | 26 | HN075 | HN125 |  | 639 | 23.7083 | 28 | HN024 | HN107 |
| 640 | 14.7222 | 19 | HN075 | HN142 |  | 640 | 18.4912 | 24 | HN024 | HN108 |
| 641 | 22.5732 | 27 | HN077 | HN083 |  | 641 | 19.9472 | 26 | HN024 | HN120 |
| 642 | 18.1997 | 20 | HN077 | HN108 |  | 642 | 22.4357 | 26 | HN024 | HN138 |
| 643 | 24.0956 | 24 | HN077 | HN116 |  | 643 | 19.1339 | 20 | HN025 | HN026 |
| 644 | 19.0964 | 21 | HN077 | HN125 |  | 644 | 19.2126 | 20 | HN025 | HN031 |
| 645 | 18.2382 | 24 | HN077 | HN126 |  | 645 | 21.4255 | 24 | HN025 | HN034 |
| 646 | 18.8401 | 26 | HN077 | HN130 |  | 646 | 18.9238 | 21 | HN025 | HN059 |
| 647 | 18.2842 | 23 | HN077 | HN132 |  | 647 | 21.0006 | 22 | HN025 | HN060 |
| 648 | 22.2179 | 22 | HN079 | HN113 |  | 648 | 22.166 | 22 | HN025 | HN061 |
| 649 | 20.6549 | 25 | HN079 | HN117 |  | 649 | 23.6898 | 24 | HN025 | HN064 |
| 650 | 20.5831 | 27 | HN079 | HN131 |  | 650 | 24.2611 | 25 | HN025 | HN064 |
| 651 | 22.0942 | 29 | HN081 | HN083 |  | 651 | 17.7087 | 21 | HN025 | HN067 |
| 652 | 22.2431 | 27 | HN081 | HN103 |  | 652 | 19.6101 | 22 | HN025 | HN075 |
| 653 | 21.4986 | 25 | HN081 | HN103 |  | 653 | 23.6667 | 27 | HN025 | HN105 |
| 654 | 22.0562 | 21 | HN082 | HN093 |  | 654 | 24.4612 | 26 | HN025 | HN105 |
| 655 | 20.5548 | 23 | HN082 | HN100 |  | 655 | 18.1321 | 22 | HN025 | HN117 |
| 656 | 25.3748 | 27 | HN082 | HN100 |  | 656 | 22.8157 | 26 | HN025 | HN127 |
| 657 | 17.9976 | 20 | HN082 | HN106 |  | 657 | 21.8767 | 24 | HN025 | HN127 |
| 658 | 25.0568 | 26 | HN082 | HN110 |  | 658 | 19.3429 | 22 | HN025 | HN137 |
| 659 | 23.8587 | 23 | HN082 | HN111 |  | 659 | 20.246 | 24 | HN025 | HN137 |
| 660 | 25.0787 | 24 | HN082 | HN114 |  | 660 | 17.282 | 20 | HN025 | HN141 |
| 661 | 26.9765 | 27 | HN082 | HN116 |  | 661 | 22.8175 | 25 | HN025 | HN143 |
| 662 | 25.267 | 28 | HN082 | HN123 |  | 662 | 14.4029 | 19 | HN025 | HN145 |
| 663 | 27.7777 | 29 | HN082 | HN125 |  | 663 | 24.4285 | 24 | HN026 | HN030 |
| 664 | 22.6523 | 26 | HN082 | HN131 |  | 664 | 24.6195 | 28 | HN026 | HN042 |
| 665 | 26.6539 | 26 | HN082 | HN140 |  | 665 | 24.5851 | 26 | HN026 | HN046 |
| 666 | 25.2513 | 27 | HN082 | HN140 |  | 666 | 25.4126 | 27 | HN026 | HN047 |
| 667 | 24.6028 | 27 | HN083 | HN085 |  | 667 | 21.7938 | 24 | HN026 | HN051 |
| 668 | 23.9159 | 30 | HN083 | HN102 |  | 668 | 22.592 | 25 | HN026 | HN057 |
| 669 | 22.2627 | 27 | HN083 | HN104 |  | 669 | 22.0252 | 26 | HN026 | HN059 |
| 670 | 23.4977 | 27 | HN083 | HN104 |  | 670 | 22.1038 | 25 | HN026 | HN061 |
| 671 | 25.7129 | 28 | HN083 | HN111 |  | 671 | 22.3515 | 22 | HN026 | HN062 |
| 672 | 24.6172 | 27 | HN083 | HN113 |  | 672 | 27.1317 | 27 | HN026 | HN065 |
| 673 | 22.523 | 31 | HN083 | HN114 |  | 673 | 25.3719 | 26 | HN026 | HN069 |
| 674 | 22.0001 | 27 | HN083 | HN124 |  | 674 | 22.7498 | 28 | HN026 | HN072 |
| 675 | 22.9837 | 29 | HN083 | HN127 |  | 675 | 26.7068 | 28 | HN026 | HN073 |
| 676 | 25.2087 | 31 | HN083 | HN127 |  | 676 | 22.0613 | 24 | HN026 | HN076 |
| 677 | 22.4197 | 29 | HN083 | HN130 |  | 677 | 28.0588 | 29 | HN026 | HN082 |
| 678 | 27.093 | 32 | HN083 | HN140 |  | 678 | 25.1175 | 24 | HN026 | HN085 |
| 679 | 23.1569 | 29 | HN083 | HN140 |  | 679 | 24.7542 | 22 | HN026 | HN088 |
| 680 | 20.2764 | 28 | HN083 | HN141 |  | 680 | 23.3648 | 25 | HN026 | HN090 |
| 681 | 24.4003 | 26 | HN083 | HN149 |  | 681 | 26.1445 | 26 | HN026 | HN090 |
| 682 | 22.036 | 29 | HN084 | HN105 |  | 682 | 22.0094 | 22 | HN026 | HN092 |
| 683 | 22.5993 | 26 | HN085 | HN090 |  | 683 | 21.4358 | 23 | HN026 | HN092 |
| 684 | 22.3847 | 19 | HN085 | HN097 |  | 684 | 24.8867 | 22 | HN026 | HN097 |
| 685 | 23.8651 | 25 | HN085 | HN116 |  | 685 | 27.5352 | 26 | HN026 | HN116 |
| 686 | 21.7507 | 27 | HN085 | HN130 |  | 686 | 21.7198 | 22 | HN026 | HN116 |
| 687 | 20.9321 | 23 | HN085 | HN144 |  | 687 | 24.9664 | 22 | HN026 | HN117 |
| 688 | 22.342 | 24 | HN086 | HN113 |  | 688 | 23.7122 | 24 | HN026 | HN119 |
| 689 | 18.7388 | 25 | HN086 | HN127 |  | 689 | 21.1186 | 22 | HN026 | HN122 |
| 690 | 19.8861 | 18 | HN086 | HN148 |  | 690 | 20.1557 | 22 | HN026 | HN122 |
| 691 | 22.4202 | 24 | HN087 | HN140 |  | 691 | 25.2988 | 27 | HN026 | HN123 |
| 692 | 22.8429 | 26 | HN087 | HN143 |  | 692 | 26.5032 | 27 | HN026 | HN125 |
| 693 | 20.5515 | 24 | HN090 | HN091 |  | 693 | 24.0357 | 26 | HN026 | HN126 |
| 694 | 29.1618 | 28 | HN090 | HN116 |  | 694 | 25.7021 | 28 | HN026 | HN127 |
| 695 | 23.8283 | 24 | HN090 | HN125 |  | 695 | 25.9656 | 28 | HN026 | HN129 |
| 696 | 24.1395 | 27 | HN090 | HN125 |  | 696 | 23.2953 | 28 | HN026 | HN130 |
| 697 | 18.6262 | 27 | HN090 | HN126 |  | 697 | 26.2852 | 29 | HN026 | HN130 |
| 698 | 25.1537 | 28 | HN090 | HN130 |  | 698 | 22.3971 | 25 | HN026 | HN131 |
| 699 | 25.1204 | 28 | HN090 | HN131 |  | 699 | 24.6495 | 25 | HN026 | HN136 |
| 700 | 23.039 | 28 | HN090 | HN140 |  | 700 | 22.6838 | 25 | HN026 | HN138 |
| 701 | 23.7502 | 29 | HN091 | HN129 |  | 701 | 22.6267 | 23 | HN026 | HN142 |
| 702 | 19.4452 | 21 | HN092 | HN123 |  | 702 | 24.1546 | 23 | HN026 | HN149 |
| 703 | 18.1333 | 22 | HN092 | HN138 |  | 703 | 23.0032 | 21 | HN026 | HN149 |
| 704 | 21.3345 | 24 | HN093 | HN094 |  | 704 | 21.623 | 21 | HN027 | HN030 |
| 705 | 24.2656 | 28 | HN093 | HN105 |  | 705 | 18.3541 | 20 | HN027 | HN041 |
| 706 | 22.8445 | 24 | HN093 | HN112 |  | 706 | 21.7053 | 24 | HN027 | HN048 |
| 707 | 19.6147 | 22 | HN093 | HN114 |  | 707 | 19.8695 | 21 | HN027 | HN064 |
| 708 | 20.9308 | 22 | HN093 | HN118 |  | 708 | 21.9963 | 23 | HN027 | HN067 |
| 709 | 24.2813 | 24 | HN093 | HN125 |  | 709 | 25.6453 | 26 | HN027 | HN068 |
| 710 | 23.6978 | 29 | HN094 | HN107 |  | 710 | 25.6234 | 25 | HN027 | HN068 |
| 711 | 20.5164 | 23 | HN094 | HN111 |  | 711 | 23.2127 | 27 | HN027 | HN080 |
| 712 | 22.7672 | 27 | HN094 | HN117 |  | 712 | 16.5946 | 21 | HN027 | HN096 |
| 713 | 25.5633 | 29 | HN094 | HN125 |  | 713 | 20.365 | 17 | HN027 | HN097 |
| 714 | 23.7823 | 29 | HN094 | HN126 |  | 714 | 21.3455 | 24 | HN027 | HN101 |
| 715 | 25.2448 | 28 | HN094 | HN130 |  | 715 | 25.5 | 23 | HN027 | HN111 |
| 716 | 25.9354 | 32 | HN094 | HN130 |  | 716 | 26.9663 | 26 | HN027 | HN111 |
| 717 | 24.7971 | 28 | HN094 | HN131 |  | 717 | 22.6153 | 25 | HN027 | HN125 |
| 718 | 23.7547 | 29 | HN094 | HN131 |  | 718 | 19.1402 | 20 | HN027 | HN127 |
| 719 | 23.7288 | 28 | HN094 | HN133 |  | 719 | 22.3997 | 26 | HN027 | HN131 |
| 720 | 21.8897 | 28 | HN094 | HN134 |  | 720 | 17.8259 | 21 | HN027 | HN141 |
| 721 | 25.0882 | 28 | HN094 | HN141 |  | 721 | 20.2393 | 20 | HN027 | HN141 |
| 722 | 23.2403 | 29 | HN094 | HN141 |  | 722 | 19.139 | 21 | HN029 | HN033 |
| 723 | 20.3283 | 20 | HN094 | HN142 |  | 723 | 19.7272 | 23 | HN029 | HN053 |
| 724 | 20.6063 | 23 | HN094 | HN149 |  | 724 | 22.553 | 22 | HN029 | HN058 |
| 725 | 19.7998 | 24 | HN095 | HN096 |  | 725 | 18.6781 | 19 | HN029 | HN064 |
| 726 | 20.023 | 24 | HN096 | HN104 |  | 726 | 17.7643 | 25 | HN029 | HN065 |
| 727 | 25.1773 | 31 | HN096 | HN105 |  | 727 | 23.6041 | 28 | HN029 | HN083 |
| 728 | 22.6312 | 29 | HN096 | HN105 |  | 728 | 19.6255 | 20 | HN029 | HN105 |
| 729 | 24.0531 | 24 | HN096 | HN106 |  | 729 | 24.9403 | 25 | HN029 | HN113 |
| 730 | 18.4211 | 26 | HN096 | HN118 |  | 730 | 22.0116 | 26 | HN029 | HN131 |
| 731 | 23.8635 | 27 | HN096 | HN118 |  | 731 | 22.0523 | 20 | HN030 | HN034 |
| 732 | 25.2574 | 30 | HN096 | HN130 |  | 732 | 21.5343 | 26 | HN030 | HN050 |
| 733 | 21.4469 | 28 | HN096 | HN131 |  | 733 | 22.3051 | 28 | HN030 | HN055 |
| 734 | 22.3753 | 26 | HN096 | HN143 |  | 734 | 21.3236 | 23 | HN030 | HN075 |
| 735 | 25.2475 | 23 | HN097 | HN116 |  | 735 | 20.2008 | 19 | HN030 | HN075 |
| 736 | 21.2995 | 22 | HN097 | HN131 |  | 736 | 23.7766 | 25 | HN030 | HN096 |
| 737 | 22.0185 | 20 | HN097 | HN145 |  | 737 | 22.889 | 23 | HN030 | HN113 |
| 738 | 17.9348 | 23 | HN098 | HN105 |  | 738 | 23.4099 | 27 | HN030 | HN115 |
| 739 | 18.9765 | 24 | HN098 | HN111 |  | 739 | 24.9757 | 24 | HN030 | HN116 |
| 740 | 20.6208 | 22 | HN098 | HN150 |  | 740 | 18.6469 | 19 | HN030 | HN126 |
| 741 | 21.8849 | 25 | HN101 | HN127 |  | 741 | 19.0681 | 20 | HN030 | HN129 |
| 742 | 18.0421 | 20 | HN101 | HN149 |  | 742 | 20.0331 | 19 | HN030 | HN132 |
| 743 | 18.9216 | 25 | HN102 | HN105 |  | 743 | 20.5057 | 20 | HN030 | HN140 |
| 744 | 21.9855 | 26 | HN102 | HN105 |  | 744 | 20.5681 | 25 | HN031 | HN039 |
| 745 | 21.6963 | 23 | HN103 | HN104 |  | 745 | 19.5511 | 21 | HN031 | HN051 |
| 746 | 21.0712 | 25 | HN103 | HN109 |  | 746 | 18.5581 | 19 | HN031 | HN061 |
| 747 | 21.0424 | 24 | HN103 | HN111 |  | 747 | 18.932 | 18 | HN031 | HN076 |
| 748 | 25.7537 | 32 | HN103 | HN130 |  | 748 | 22.1852 | 22 | HN031 | HN082 |
| 749 | 23.9755 | 24 | HN103 | HN144 |  | 749 | 19.4262 | 20 | HN031 | HN108 |
| 750 | 18.8358 | 28 | HN103 | HN147 |  | 750 | 20.4118 | 16 | HN031 | HN116 |
| 751 | 22.0956 | 24 | HN105 | HN109 |  | 751 | 19.5965 | 21 | HN031 | HN123 |
| 752 | 24.534 | 29 | HN105 | HN128 |  | 752 | 21.6675 | 22 | HN032 | HN041 |
| 753 | 19.9888 | 28 | HN105 | HN134 |  | 753 | 21.7039 | 24 | HN032 | HN051 |
| 754 | 25.9759 | 25 | HN106 | HN116 |  | 754 | 20.8233 | 24 | HN032 | HN059 |
| 755 | 25.163 | 23 | HN106 | HN129 |  | 755 | 19.8125 | 22 | HN032 | HN059 |
| 756 | 21.5521 | 19 | HN106 | HN132 |  | 756 | 22.361 | 24 | HN032 | HN069 |
| 757 | 25.4631 | 26 | HN106 | HN138 |  | 757 | 21.9485 | 21 | HN032 | HN070 |
| 758 | 23.0954 | 25 | HN106 | HN147 |  | 758 | 23.0117 | 23 | HN032 | HN072 |
| 759 | 22.4499 | 22 | HN108 | HN116 |  | 759 | 21.3302 | 23 | HN032 | HN079 |
| 760 | 24.0206 | 26 | HN109 | HN113 |  | 760 | 22.3747 | 24 | HN032 | HN085 |
| 761 | 18.7064 | 23 | HN109 | HN140 |  | 761 | 21.1918 | 22 | HN032 | HN085 |
| 762 | 20.3463 | 25 | HN110 | HN130 |  | 762 | 25.0936 | 25 | HN032 | HN090 |
| 763 | 19.8163 | 24 | HN110 | HN134 |  | 763 | 25.2349 | 26 | HN032 | HN094 |
| 764 | 19.9632 | 20 | HN110 | HN137 |  | 764 | 25.3126 | 26 | HN032 | HN094 |
| 765 | 21.4038 | 22 | HN111 | HN126 |  | 765 | 23.4257 | 25 | HN032 | HN098 |
| 766 | 18.1176 | 18 | HN111 | HN139 |  | 766 | 22.411 | 23 | HN032 | HN105 |
| 767 | 19.8405 | 26 | HN111 | HN147 |  | 767 | 21.5975 | 23 | HN032 | HN111 |
| 768 | 27.0611 | 26 | HN112 | HN116 |  | 768 | 22.6798 | 23 | HN032 | HN113 |
| 769 | 24.2788 | 25 | HN112 | HN116 |  | 769 | 20.5702 | 23 | HN032 | HN117 |
| 770 | 20.7866 | 26 | HN112 | HN126 |  | 770 | 20.9126 | 22 | HN032 | HN123 |
| 771 | 20.3255 | 24 | HN112 | HN150 |  | 771 | 21.0523 | 22 | HN032 | HN123 |
| 772 | 22.9585 | 24 | HN113 | HN117 |  | 772 | 24.9166 | 29 | HN032 | HN130 |
| 773 | 17.2148 | 23 | HN113 | HN120 |  | 773 | 24.3474 | 27 | HN032 | HN130 |
| 774 | 24.5734 | 27 | HN113 | HN127 |  | 774 | 22.4556 | 25 | HN032 | HN137 |
| 775 | 21.4877 | 22 | HN113 | HN135 |  | 775 | 18.515 | 18 | HN032 | HN142 |
| 776 | 19.2571 | 18 | HN114 | HN117 |  | 776 | 24.4192 | 25 | HN032 | HN143 |
| 777 | 21.413 | 28 | HN114 | HN129 |  | 777 | 21.2551 | 24 | HN033 | HN039 |
| 778 | 17.8478 | 19 | HN114 | HN134 |  | 778 | 18.5845 | 23 | HN033 | HN057 |
| 779 | 21.7445 | 24 | HN115 | HN132 |  | 779 | 20.8997 | 24 | HN033 | HN064 |
| 780 | 23.1247 | 25 | HN116 | HN123 |  | 780 | 18.0251 | 23 | HN033 | HN067 |
| 781 | 25.8225 | 29 | HN116 | HN125 |  | 781 | 20.4308 | 24 | HN033 | HN067 |
| 782 | 21.5712 | 24 | HN116 | HN126 |  | 782 | 19.9183 | 24 | HN033 | HN075 |
| 783 | 27.0243 | 30 | HN116 | HN131 |  | 783 | 24.6996 | 27 | HN033 | HN082 |
| 784 | 24.1952 | 26 | HN116 | HN132 |  | 784 | 24.3518 | 28 | HN033 | HN090 |
| 785 | 18.0142 | 22 | HN116 | HN141 |  | 785 | 18.8293 | 22 | HN033 | HN091 |
| 786 | 21.5411 | 22 | HN116 | HN150 |  | 786 | 21.7769 | 25 | HN033 | HN112 |
| 787 | 21.3999 | 23 | HN117 | HN132 |  | 787 | 21.352 | 24 | HN033 | HN113 |
| 788 | 22.5228 | 23 | HN117 | HN132 |  | 788 | 20.8914 | 26 | HN033 | HN125 |
| 789 | 21.8212 | 21 | HN117 | HN138 |  | 789 | 23.5596 | 29 | HN033 | HN129 |
| 790 | 21.2786 | 23 | HN118 | HN134 |  | 790 | 24.2352 | 25 | HN033 | HN129 |
| 791 | 22.2811 | 26 | HN118 | HN136 |  | 791 | 21.4398 | 24 | HN034 | HN036 |
| 792 | 22.1877 | 24 | HN118 | HN146 |  | 792 | 22.5248 | 25 | HN034 | HN038 |
| 793 | 19.6492 | 25 | HN120 | HN126 |  | 793 | 25.4908 | 28 | HN034 | HN040 |
| 794 | 20.3929 | 23 | HN120 | HN128 |  | 794 | 22.7301 | 28 | HN034 | HN046 |
| 795 | 17.4634 | 21 | HN121 | HN123 |  | 795 | 22.0521 | 29 | HN034 | HN046 |
| 796 | 23.2209 | 28 | HN123 | HN131 |  | 796 | 25.3757 | 27 | HN034 | HN051 |
| 797 | 18.9978 | 24 | HN124 | HN137 |  | 797 | 24.9499 | 25 | HN034 | HN052 |
| 798 | 19.7852 | 24 | HN124 | HN143 |  | 798 | 25.5519 | 28 | HN034 | HN052 |
| 799 | 21.172 | 25 | HN125 | HN132 |  | 799 | 23.1827 | 27 | HN034 | HN055 |
| 800 | 20.0825 | 26 | HN126 | HN127 |  | 800 | 25.2817 | 29 | HN034 | HN057 |
| 801 | 24.7617 | 30 | HN127 | HN131 |  | 801 | 23.6439 | 27 | HN034 | HN057 |
| 802 | 22.2215 | 27 | HN127 | HN137 |  | 802 | 24.886 | 27 | HN034 | HN060 |
| 803 | 20.0328 | 30 | HN127 | HN137 |  | 803 | 25.8553 | 28 | HN034 | HN061 |
| 804 | 23.4076 | 30 | HN128 | HN130 |  | 804 | 25.4124 | 27 | HN034 | HN065 |
| 805 | 17.3894 | 20 | HN128 | HN134 |  | 805 | 24.3976 | 28 | HN034 | HN067 |
| 806 | 20.8798 | 22 | HN128 | HN144 |  | 806 | 24.3666 | 25 | HN034 | HN069 |
| 807 | 22.0427 | 26 | HN129 | HN132 |  | 807 | 26.9435 | 26 | HN034 | HN070 |
| 808 | 25.5835 | 29 | HN129 | HN133 |  | 808 | 24.1786 | 27 | HN034 | HN076 |
| 809 | 25.7834 | 28 | HN129 | HN137 |  | 809 | 22.552 | 22 | HN034 | HN080 |
| 810 | 22.8946 | 28 | HN129 | HN147 |  | 810 | 24.6238 | 26 | HN034 | HN082 |
| 811 | 21.3196 | 23 | HN130 | HN148 |  | 811 | 22.1087 | 24 | HN034 | HN084 |
| 812 | 21.966 | 26 | HN131 | HN141 |  | 812 | 25.768 | 27 | HN034 | HN085 |
| 813 | 23.6452 | 28 | HN131 | HN146 |  | 813 | 25.4938 | 26 | HN034 | HN087 |
| 814 | 22.8018 | 25 | HN132 | HN135 |  | 814 | 24.9513 | 28 | HN034 | HN090 |
| 815 | 23.8482 | 25 | HN132 | HN137 |  | 815 | 23.8249 | 26 | HN034 | HN091 |
| 816 | 20.6398 | 23 | HN133 | HN141 |  | 816 | 23.4656 | 26 | HN034 | HN093 |
| 817 | 19.7561 | 25 | HN133 | HN147 |  | 817 | 26.0936 | 30 | HN034 | HN094 |
| 818 | 17.2783 | 22 | HN134 | HN141 |  | 818 | 22.5085 | 27 | HN034 | HN098 |
| 819 | 17.4521 | 25 | HN139 | HN147 |  | 819 | 24.9477 | 26 | HN034 | HN101 |
| 820 | 17.4521 | 25 | HN139 | HN147 |  | 820 | 26.8812 | 29 | HN034 | HN103 |
| 821 | 20.0732 | 21 | HN140 | HN142 |  | 821 | 26.5427 | 28 | HN034 | HN104 |
| 822 | 18.3095 | 20 | HN140 | HN149 |  | 822 | 21.6322 | 22 | HN034 | HN110 |
| 823 | 22.4612 | 24 | HN143 | HN148 |  | 823 | 27.4173 | 29 | HN034 | HN111 |
| 824 | 16.0541 | 20 | HN144 | HN146 |  | 824 | 24.6612 | 25 | HN034 | HN114 |
|  |  |  |  |  |  | 825 | 22.6917 | 26 | HN034 | HN115 |
|  |  |  |  |  |  | 826 | 26.2021 | 28 | HN034 | HN116 |
|  |  |  |  |  |  | 827 | 24.2017 | 26 | HN034 | HN118 |
|  |  |  |  |  |  | 828 | 25.2816 | 26 | HN034 | HN120 |
|  |  |  |  |  |  | 829 | 24.3542 | 27 | HN034 | HN124 |
|  |  |  |  |  |  | 830 | 23.8694 | 26 | HN034 | HN125 |
|  |  |  |  |  |  | 831 | 26.4376 | 30 | HN034 | HN127 |
|  |  |  |  |  |  | 832 | 26.0646 | 30 | HN034 | HN129 |
|  |  |  |  |  |  | 833 | 24.0364 | 26 | HN034 | HN132 |
|  |  |  |  |  |  | 834 | 22.1386 | 24 | HN034 | HN132 |
|  |  |  |  |  |  | 835 | 24.9157 | 28 | HN034 | HN136 |
|  |  |  |  |  |  | 836 | 25.762 | 28 | HN034 | HN137 |
|  |  |  |  |  |  | 837 | 25.879 | 27 | HN034 | HN139 |
|  |  |  |  |  |  | 838 | 26.2668 | 29 | HN034 | HN140 |
|  |  |  |  |  |  | 839 | 24.5749 | 23 | HN034 | HN142 |
|  |  |  |  |  |  | 840 | 25.2999 | 25 | HN034 | HN142 |
|  |  |  |  |  |  | 841 | 24.5679 | 27 | HN034 | HN143 |
|  |  |  |  |  |  | 842 | 21.8621 | 24 | HN034 | HN145 |
|  |  |  |  |  |  | 843 | 22.9659 | 21 | HN034 | HN148 |
|  |  |  |  |  |  | 844 | 21.456 | 22 | HN034 | HN149 |
|  |  |  |  |  |  | 845 | 21.3093 | 24 | HN034 | HN149 |
|  |  |  |  |  |  | 846 | 23.1006 | 25 | HN035 | HN037 |
|  |  |  |  |  |  | 847 | 20.2759 | 19 | HN035 | HN048 |
|  |  |  |  |  |  | 848 | 26.6003 | 28 | HN035 | HN050 |
|  |  |  |  |  |  | 849 | 21.1942 | 23 | HN035 | HN057 |
|  |  |  |  |  |  | 850 | 22.5111 | 24 | HN035 | HN058 |
|  |  |  |  |  |  | 851 | 21.0267 | 22 | HN035 | HN060 |
|  |  |  |  |  |  | 852 | 22.4105 | 22 | HN035 | HN060 |
|  |  |  |  |  |  | 853 | 22.0104 | 21 | HN035 | HN061 |
|  |  |  |  |  |  | 854 | 21.7387 | 23 | HN035 | HN082 |
|  |  |  |  |  |  | 855 | 19.9227 | 22 | HN035 | HN087 |
|  |  |  |  |  |  | 856 | 21.465 | 23 | HN035 | HN107 |
|  |  |  |  |  |  | 857 | 19.1341 | 21 | HN035 | HN109 |
|  |  |  |  |  |  | 858 | 19.1262 | 25 | HN035 | HN109 |
|  |  |  |  |  |  | 859 | 24.6104 | 24 | HN035 | HN111 |
|  |  |  |  |  |  | 860 | 20.7602 | 22 | HN035 | HN111 |
|  |  |  |  |  |  | 861 | 21.0308 | 22 | HN035 | HN112 |
|  |  |  |  |  |  | 862 | 25.1391 | 23 | HN035 | HN113 |
|  |  |  |  |  |  | 863 | 28.1745 | 28 | HN035 | HN116 |
|  |  |  |  |  |  | 864 | 24.1063 | 23 | HN035 | HN117 |
|  |  |  |  |  |  | 865 | 24.8426 | 27 | HN035 | HN123 |
|  |  |  |  |  |  | 866 | 22.8382 | 24 | HN035 | HN125 |
|  |  |  |  |  |  | 867 | 19.4482 | 22 | HN035 | HN132 |
|  |  |  |  |  |  | 868 | 24.9905 | 28 | HN035 | HN133 |
|  |  |  |  |  |  | 869 | 22.83 | 22 | HN035 | HN137 |
|  |  |  |  |  |  | 870 | 21.4048 | 21 | HN035 | HN137 |
|  |  |  |  |  |  | 871 | 20.6377 | 22 | HN035 | HN146 |
|  |  |  |  |  |  | 872 | 20.9865 | 24 | HN035 | HN147 |
|  |  |  |  |  |  | 873 | 14.9986 | 19 | HN036 | HN059 |
|  |  |  |  |  |  | 874 | 21.7922 | 24 | HN036 | HN061 |
|  |  |  |  |  |  | 875 | 19.5622 | 22 | HN036 | HN070 |
|  |  |  |  |  |  | 876 | 22.6455 | 30 | HN036 | HN083 |
|  |  |  |  |  |  | 877 | 20.6765 | 25 | HN036 | HN105 |
|  |  |  |  |  |  | 878 | 19.1638 | 21 | HN036 | HN127 |
|  |  |  |  |  |  | 879 | 22.8815 | 25 | HN036 | HN131 |
|  |  |  |  |  |  | 880 | 19.6574 | 20 | HN036 | HN140 |
|  |  |  |  |  |  | 881 | 19.7064 | 19 | HN036 | HN148 |
|  |  |  |  |  |  | 882 | 20.0331 | 25 | HN037 | HN044 |
|  |  |  |  |  |  | 883 | 21.4851 | 28 | HN037 | HN047 |
|  |  |  |  |  |  | 884 | 23.3642 | 30 | HN037 | HN055 |
|  |  |  |  |  |  | 885 | 21.2464 | 25 | HN037 | HN060 |
|  |  |  |  |  |  | 886 | 19.7249 | 26 | HN037 | HN065 |
|  |  |  |  |  |  | 887 | 21.8912 | 23 | HN037 | HN070 |
|  |  |  |  |  |  | 888 | 24.1788 | 29 | HN037 | HN083 |
|  |  |  |  |  |  | 889 | 20.4434 | 22 | HN037 | HN088 |
|  |  |  |  |  |  | 890 | 21.608 | 27 | HN037 | HN093 |
|  |  |  |  |  |  | 891 | 25.1399 | 29 | HN037 | HN094 |
|  |  |  |  |  |  | 892 | 25.6468 | 30 | HN037 | HN096 |
|  |  |  |  |  |  | 893 | 22.0625 | 22 | HN037 | HN097 |
|  |  |  |  |  |  | 894 | 26.9832 | 31 | HN037 | HN105 |
|  |  |  |  |  |  | 895 | 26.2575 | 29 | HN037 | HN105 |
|  |  |  |  |  |  | 896 | 25.4172 | 30 | HN037 | HN105 |
|  |  |  |  |  |  | 897 | 21.7889 | 28 | HN037 | HN112 |
|  |  |  |  |  |  | 898 | 22.952 | 29 | HN037 | HN113 |
|  |  |  |  |  |  | 899 | 26.0398 | 27 | HN037 | HN113 |
|  |  |  |  |  |  | 900 | 20.2953 | 26 | HN037 | HN119 |
|  |  |  |  |  |  | 901 | 22.3341 | 28 | HN037 | HN125 |
|  |  |  |  |  |  | 902 | 24.6045 | 27 | HN037 | HN129 |
|  |  |  |  |  |  | 903 | 20.2014 | 24 | HN037 | HN141 |
|  |  |  |  |  |  | 904 | 19.2044 | 24 | HN037 | HN149 |
|  |  |  |  |  |  | 905 | 23.6871 | 25 | HN038 | HN064 |
|  |  |  |  |  |  | 906 | 19.3163 | 21 | HN038 | HN077 |
|  |  |  |  |  |  | 907 | 22.7456 | 28 | HN038 | HN083 |
|  |  |  |  |  |  | 908 | 19.4901 | 23 | HN038 | HN114 |
|  |  |  |  |  |  | 909 | 22.3423 | 26 | HN038 | HN115 |
|  |  |  |  |  |  | 910 | 18.7464 | 23 | HN038 | HN136 |
|  |  |  |  |  |  | 911 | 17.4345 | 20 | HN038 | HN141 |
|  |  |  |  |  |  | 912 | 20.7974 | 22 | HN038 | HN148 |
|  |  |  |  |  |  | 913 | 21.0256 | 24 | HN038 | HN149 |
|  |  |  |  |  |  | 914 | 24.9171 | 30 | HN039 | HN061 |
|  |  |  |  |  |  | 915 | 24.6127 | 28 | HN039 | HN065 |
|  |  |  |  |  |  | 916 | 20.4708 | 24 | HN039 | HN069 |
|  |  |  |  |  |  | 917 | 16.068 | 21 | HN039 | HN072 |
|  |  |  |  |  |  | 918 | 22.0648 | 24 | HN039 | HN077 |
|  |  |  |  |  |  | 919 | 24.8238 | 28 | HN039 | HN094 |
|  |  |  |  |  |  | 920 | 24.2059 | 26 | HN039 | HN094 |
|  |  |  |  |  |  | 921 | 19.8751 | 28 | HN039 | HN096 |
|  |  |  |  |  |  | 922 | 19.4173 | 26 | HN039 | HN102 |
|  |  |  |  |  |  | 923 | 25.0073 | 27 | HN039 | HN103 |
|  |  |  |  |  |  | 924 | 21.6485 | 28 | HN039 | HN131 |
|  |  |  |  |  |  | 925 | 21.1802 | 20 | HN040 | HN043 |
|  |  |  |  |  |  | 926 | 20.1929 | 23 | HN040 | HN043 |
|  |  |  |  |  |  | 927 | 18.1922 | 23 | HN040 | HN051 |
|  |  |  |  |  |  | 928 | 16.9044 | 22 | HN040 | HN077 |
|  |  |  |  |  |  | 929 | 17.8595 | 22 | HN040 | HN079 |
|  |  |  |  |  |  | 930 | 18.9521 | 22 | HN040 | HN080 |
|  |  |  |  |  |  | 931 | 22.8175 | 25 | HN040 | HN082 |
|  |  |  |  |  |  | 932 | 24.0184 | 29 | HN040 | HN083 |
|  |  |  |  |  |  | 933 | 21.45 | 27 | HN040 | HN105 |
|  |  |  |  |  |  | 934 | 17.0047 | 20 | HN040 | HN110 |
|  |  |  |  |  |  | 935 | 22.2912 | 27 | HN040 | HN115 |
|  |  |  |  |  |  | 936 | 20.5389 | 24 | HN040 | HN115 |
|  |  |  |  |  |  | 937 | 17.0514 | 20 | HN040 | HN126 |
|  |  |  |  |  |  | 938 | 19.519 | 23 | HN041 | HN050 |
|  |  |  |  |  |  | 939 | 21.4679 | 24 | HN041 | HN060 |
|  |  |  |  |  |  | 940 | 23.1192 | 27 | HN041 | HN069 |
|  |  |  |  |  |  | 941 | 27.4518 | 29 | HN041 | HN073 |
|  |  |  |  |  |  | 942 | 21.0899 | 24 | HN041 | HN080 |
|  |  |  |  |  |  | 943 | 23.8963 | 23 | HN041 | HN097 |
|  |  |  |  |  |  | 944 | 24.644 | 24 | HN041 | HN111 |
|  |  |  |  |  |  | 945 | 22.8042 | 25 | HN041 | HN111 |
|  |  |  |  |  |  | 946 | 24.9272 | 24 | HN041 | HN116 |
|  |  |  |  |  |  | 947 | 20.368 | 24 | HN041 | HN120 |
|  |  |  |  |  |  | 948 | 18.8488 | 22 | HN041 | HN122 |
|  |  |  |  |  |  | 949 | 23.2355 | 28 | HN041 | HN125 |
|  |  |  |  |  |  | 950 | 16.9727 | 21 | HN041 | HN134 |
|  |  |  |  |  |  | 951 | 18.8879 | 24 | HN041 | HN136 |
|  |  |  |  |  |  | 952 | 24.4174 | 28 | HN041 | HN138 |
|  |  |  |  |  |  | 953 | 19.8201 | 25 | HN041 | HN145 |
|  |  |  |  |  |  | 954 | 20.0708 | 21 | HN042 | HN043 |
|  |  |  |  |  |  | 955 | 23.1656 | 23 | HN042 | HN043 |
|  |  |  |  |  |  | 956 | 17.1531 | 23 | HN042 | HN060 |
|  |  |  |  |  |  | 957 | 20.1846 | 21 | HN042 | HN061 |
|  |  |  |  |  |  | 958 | 22.3521 | 25 | HN042 | HN068 |
|  |  |  |  |  |  | 959 | 21.9263 | 24 | HN042 | HN117 |
|  |  |  |  |  |  | 960 | 23.1482 | 26 | HN042 | HN118 |
|  |  |  |  |  |  | 961 | 20.2766 | 24 | HN042 | HN129 |
|  |  |  |  |  |  | 962 | 20.514 | 26 | HN042 | HN129 |
|  |  |  |  |  |  | 963 | 23.2193 | 29 | HN042 | HN130 |
|  |  |  |  |  |  | 964 | 20.3192 | 23 | HN042 | HN148 |
|  |  |  |  |  |  | 965 | 16.9418 | 22 | HN043 | HN044 |
|  |  |  |  |  |  | 966 | 22.4077 | 21 | HN043 | HN045 |
|  |  |  |  |  |  | 967 | 23.8203 | 25 | HN043 | HN047 |
|  |  |  |  |  |  | 968 | 24.6965 | 30 | HN043 | HN050 |
|  |  |  |  |  |  | 969 | 20.9572 | 21 | HN043 | HN051 |
|  |  |  |  |  |  | 970 | 20.8298 | 20 | HN043 | HN054 |
|  |  |  |  |  |  | 971 | 26.056 | 31 | HN043 | HN055 |
|  |  |  |  |  |  | 972 | 23.5505 | 25 | HN043 | HN059 |
|  |  |  |  |  |  | 973 | 22.3794 | 21 | HN043 | HN069 |
|  |  |  |  |  |  | 974 | 23.6628 | 23 | HN043 | HN069 |
|  |  |  |  |  |  | 975 | 21.3385 | 21 | HN043 | HN070 |
|  |  |  |  |  |  | 976 | 26.1085 | 26 | HN043 | HN073 |
|  |  |  |  |  |  | 977 | 25.1429 | 26 | HN043 | HN073 |
|  |  |  |  |  |  | 978 | 23.2588 | 23 | HN043 | HN074 |
|  |  |  |  |  |  | 979 | 20.2676 | 17 | HN043 | HN076 |
|  |  |  |  |  |  | 980 | 23.2509 | 24 | HN043 | HN080 |
|  |  |  |  |  |  | 981 | 26.9087 | 27 | HN043 | HN082 |
|  |  |  |  |  |  | 982 | 25.4022 | 29 | HN043 | HN083 |
|  |  |  |  |  |  | 983 | 24.4142 | 26 | HN043 | HN083 |
|  |  |  |  |  |  | 984 | 21.7094 | 22 | HN043 | HN084 |
|  |  |  |  |  |  | 985 | 22.4915 | 23 | HN043 | HN085 |
|  |  |  |  |  |  | 986 | 24.4971 | 26 | HN043 | HN087 |
|  |  |  |  |  |  | 987 | 27.5032 | 28 | HN043 | HN090 |
|  |  |  |  |  |  | 988 | 22.5224 | 21 | HN043 | HN093 |
|  |  |  |  |  |  | 989 | 24.4261 | 23 | HN043 | HN094 |
|  |  |  |  |  |  | 990 | 24.1715 | 26 | HN043 | HN096 |
|  |  |  |  |  |  | 991 | 26.4456 | 24 | HN043 | HN105 |
|  |  |  |  |  |  | 992 | 25.9793 | 27 | HN043 | HN105 |
|  |  |  |  |  |  | 993 | 22.1988 | 22 | HN043 | HN111 |
|  |  |  |  |  |  | 994 | 25.3304 | 23 | HN043 | HN113 |
|  |  |  |  |  |  | 995 | 25.4969 | 31 | HN043 | HN115 |
|  |  |  |  |  |  | 996 | 25.5283 | 24 | HN043 | HN118 |
|  |  |  |  |  |  | 997 | 20.1595 | 19 | HN043 | HN122 |
|  |  |  |  |  |  | 998 | 24.931 | 29 | HN043 | HN129 |
|  |  |  |  |  |  | 999 | 20.3971 | 20 | HN043 | HN133 |
|  |  |  |  |  |  | 1000 | 22.3244 | 22 | HN043 | HN136 |
|  |  |  |  |  |  | 1001 | 22.2052 | 21 | HN043 | HN138 |
|  |  |  |  |  |  | 1002 | 22.5672 | 24 | HN043 | HN140 |
|  |  |  |  |  |  | 1003 | 19.5372 | 24 | HN043 | HN141 |
|  |  |  |  |  |  | 1004 | 21.1459 | 22 | HN043 | HN141 |
|  |  |  |  |  |  | 1005 | 24.0946 | 29 | HN043 | HN147 |
|  |  |  |  |  |  | 1006 | 24.9377 | 28 | HN043 | HN147 |
|  |  |  |  |  |  | 1007 | 18.7784 | 25 | HN044 | HN055 |
|  |  |  |  |  |  | 1008 | 21.8556 | 26 | HN044 | HN064 |
|  |  |  |  |  |  | 1009 | 20.4645 | 24 | HN044 | HN065 |
|  |  |  |  |  |  | 1010 | 18.1825 | 23 | HN044 | HN072 |
|  |  |  |  |  |  | 1011 | 19.6644 | 23 | HN044 | HN107 |
|  |  |  |  |  |  | 1012 | 24.2164 | 25 | HN044 | HN116 |
|  |  |  |  |  |  | 1013 | 24.2164 | 25 | HN044 | HN116 |
|  |  |  |  |  |  | 1014 | 19.9637 | 22 | HN044 | HN140 |
|  |  |  |  |  |  | 1015 | 21.7909 | 27 | HN045 | HN049 |
|  |  |  |  |  |  | 1016 | 24.6132 | 31 | HN045 | HN050 |
|  |  |  |  |  |  | 1017 | 19.4249 | 26 | HN045 | HN055 |
|  |  |  |  |  |  | 1018 | 18.755 | 28 | HN045 | HN055 |
|  |  |  |  |  |  | 1019 | 22.5946 | 27 | HN045 | HN073 |
|  |  |  |  |  |  | 1020 | 20.4717 | 23 | HN045 | HN081 |
|  |  |  |  |  |  | 1021 | 25.5159 | 30 | HN045 | HN083 |
|  |  |  |  |  |  | 1022 | 21.2713 | 25 | HN045 | HN091 |
|  |  |  |  |  |  | 1023 | 22.9129 | 27 | HN045 | HN096 |
|  |  |  |  |  |  | 1024 | 23.9144 | 26 | HN045 | HN097 |
|  |  |  |  |  |  | 1025 | 21.1098 | 26 | HN045 | HN103 |
|  |  |  |  |  |  | 1026 | 21.5668 | 23 | HN045 | HN107 |
|  |  |  |  |  |  | 1027 | 17.4127 | 21 | HN045 | HN139 |
|  |  |  |  |  |  | 1028 | 15.6137 | 25 | HN045 | HN144 |
|  |  |  |  |  |  | 1029 | 18.5447 | 22 | HN045 | HN145 |
|  |  |  |  |  |  | 1030 | 19.0906 | 21 | HN045 | HN148 |
|  |  |  |  |  |  | 1031 | 21.2317 | 26 | HN046 | HN074 |
|  |  |  |  |  |  | 1032 | 15.7569 | 26 | HN046 | HN112 |
|  |  |  |  |  |  | 1033 | 17.9498 | 25 | HN046 | HN114 |
|  |  |  |  |  |  | 1034 | 17.6319 | 25 | HN046 | HN127 |
|  |  |  |  |  |  | 1035 | 19.416 | 25 | HN046 | HN132 |
|  |  |  |  |  |  | 1036 | 21.2116 | 24 | HN046 | HN136 |
|  |  |  |  |  |  | 1037 | 16.633 | 22 | HN046 | HN144 |
|  |  |  |  |  |  | 1038 | 18.1191 | 23 | HN047 | HN049 |
|  |  |  |  |  |  | 1039 | 19.9431 | 24 | HN047 | HN051 |
|  |  |  |  |  |  | 1040 | 19.0007 | 24 | HN047 | HN055 |
|  |  |  |  |  |  | 1041 | 19.4594 | 24 | HN047 | HN057 |
|  |  |  |  |  |  | 1042 | 24.0132 | 25 | HN047 | HN061 |
|  |  |  |  |  |  | 1043 | 27.2733 | 28 | HN047 | HN073 |
|  |  |  |  |  |  | 1044 | 18.7218 | 22 | HN047 | HN091 |
|  |  |  |  |  |  | 1045 | 13.9866 | 19 | HN047 | HN100 |
|  |  |  |  |  |  | 1046 | 18.1776 | 21 | HN047 | HN104 |
|  |  |  |  |  |  | 1047 | 23.0534 | 29 | HN047 | HN130 |
|  |  |  |  |  |  | 1048 | 18.4439 | 24 | HN047 | HN132 |
|  |  |  |  |  |  | 1049 | 22.8867 | 26 | HN047 | HN140 |
|  |  |  |  |  |  | 1050 | 20.7645 | 24 | HN047 | HN144 |
|  |  |  |  |  |  | 1051 | 20.7645 | 24 | HN047 | HN144 |
|  |  |  |  |  |  | 1052 | 23.2188 | 27 | HN048 | HN050 |
|  |  |  |  |  |  | 1053 | 22.0997 | 26 | HN048 | HN051 |
|  |  |  |  |  |  | 1054 | 19.4305 | 20 | HN048 | HN061 |
|  |  |  |  |  |  | 1055 | 19.9043 | 21 | HN048 | HN066 |
|  |  |  |  |  |  | 1056 | 16.9415 | 21 | HN048 | HN071 |
|  |  |  |  |  |  | 1057 | 20.564 | 21 | HN048 | HN075 |
|  |  |  |  |  |  | 1058 | 24.1426 | 25 | HN048 | HN082 |
|  |  |  |  |  |  | 1059 | 17.5721 | 17 | HN048 | HN086 |
|  |  |  |  |  |  | 1060 | 23.8689 | 27 | HN048 | HN103 |
|  |  |  |  |  |  | 1061 | 22.3853 | 27 | HN048 | HN109 |
|  |  |  |  |  |  | 1062 | 20.7944 | 22 | HN048 | HN115 |
|  |  |  |  |  |  | 1063 | 19.7049 | 17 | HN048 | HN118 |
|  |  |  |  |  |  | 1064 | 23.6346 | 23 | HN048 | HN118 |
|  |  |  |  |  |  | 1065 | 25.6706 | 29 | HN048 | HN129 |
|  |  |  |  |  |  | 1066 | 20.2993 | 20 | HN048 | HN132 |
|  |  |  |  |  |  | 1067 | 18.1366 | 22 | HN049 | HN054 |
|  |  |  |  |  |  | 1068 | 19.423 | 19 | HN049 | HN057 |
|  |  |  |  |  |  | 1069 | 20.0925 | 21 | HN049 | HN066 |
|  |  |  |  |  |  | 1070 | 21.7442 | 23 | HN049 | HN067 |
|  |  |  |  |  |  | 1071 | 20.7979 | 23 | HN049 | HN080 |
|  |  |  |  |  |  | 1072 | 25.9517 | 27 | HN049 | HN082 |
|  |  |  |  |  |  | 1073 | 24.0382 | 25 | HN049 | HN090 |
|  |  |  |  |  |  | 1074 | 23.7195 | 21 | HN049 | HN097 |
|  |  |  |  |  |  | 1075 | 23.4365 | 23 | HN049 | HN104 |
|  |  |  |  |  |  | 1076 | 20.2554 | 20 | HN049 | HN106 |
|  |  |  |  |  |  | 1077 | 22.1217 | 27 | HN049 | HN109 |
|  |  |  |  |  |  | 1078 | 19.8756 | 21 | HN049 | HN112 |
|  |  |  |  |  |  | 1079 | 18.6625 | 22 | HN049 | HN112 |
|  |  |  |  |  |  | 1080 | 21.421 | 20 | HN049 | HN113 |
|  |  |  |  |  |  | 1081 | 20.9059 | 23 | HN049 | HN131 |
|  |  |  |  |  |  | 1082 | 18.4265 | 20 | HN049 | HN140 |
|  |  |  |  |  |  | 1083 | 24.4612 | 28 | HN050 | HN058 |
|  |  |  |  |  |  | 1084 | 24.7 | 26 | HN050 | HN061 |
|  |  |  |  |  |  | 1085 | 21.5234 | 27 | HN050 | HN065 |
|  |  |  |  |  |  | 1086 | 25.0053 | 28 | HN050 | HN066 |
|  |  |  |  |  |  | 1087 | 27.9864 | 32 | HN050 | HN068 |
|  |  |  |  |  |  | 1088 | 26.5364 | 29 | HN050 | HN073 |
|  |  |  |  |  |  | 1089 | 26.804 | 29 | HN050 | HN073 |
|  |  |  |  |  |  | 1090 | 20.6319 | 26 | HN050 | HN074 |
|  |  |  |  |  |  | 1091 | 23.1654 | 29 | HN050 | HN075 |
|  |  |  |  |  |  | 1092 | 24.2169 | 27 | HN050 | HN081 |
|  |  |  |  |  |  | 1093 | 23.5956 | 28 | HN050 | HN081 |
|  |  |  |  |  |  | 1094 | 26.0112 | 30 | HN050 | HN082 |
|  |  |  |  |  |  | 1095 | 27.0411 | 33 | HN050 | HN083 |
|  |  |  |  |  |  | 1096 | 21.3335 | 25 | HN050 | HN085 |
|  |  |  |  |  |  | 1097 | 17.1386 | 23 | HN050 | HN086 |
|  |  |  |  |  |  | 1098 | 27.0362 | 30 | HN050 | HN090 |
|  |  |  |  |  |  | 1099 | 20.4765 | 24 | HN050 | HN091 |
|  |  |  |  |  |  | 1100 | 27.2813 | 32 | HN050 | HN094 |
|  |  |  |  |  |  | 1101 | 22.5811 | 26 | HN050 | HN094 |
|  |  |  |  |  |  | 1102 | 23.8247 | 29 | HN050 | HN101 |
|  |  |  |  |  |  | 1103 | 21.272 | 24 | HN050 | HN104 |
|  |  |  |  |  |  | 1104 | 22.5169 | 27 | HN050 | HN110 |
|  |  |  |  |  |  | 1105 | 20.6185 | 24 | HN050 | HN127 |
|  |  |  |  |  |  | 1106 | 29.0619 | 33 | HN050 | HN129 |
|  |  |  |  |  |  | 1107 | 23.8568 | 31 | HN050 | HN129 |
|  |  |  |  |  |  | 1108 | 22.5888 | 26 | HN050 | HN130 |
|  |  |  |  |  |  | 1109 | 23.4917 | 29 | HN050 | HN130 |
|  |  |  |  |  |  | 1110 | 22.9166 | 28 | HN050 | HN131 |
|  |  |  |  |  |  | 1111 | 23.3419 | 24 | HN050 | HN132 |
|  |  |  |  |  |  | 1112 | 21.2975 | 25 | HN050 | HN139 |
|  |  |  |  |  |  | 1113 | 23.5108 | 27 | HN050 | HN140 |
|  |  |  |  |  |  | 1114 | 25.1161 | 30 | HN050 | HN143 |
|  |  |  |  |  |  | 1115 | 20.7394 | 26 | HN051 | HN053 |
|  |  |  |  |  |  | 1116 | 20.9896 | 26 | HN051 | HN054 |
|  |  |  |  |  |  | 1117 | 22.1209 | 27 | HN051 | HN054 |
|  |  |  |  |  |  | 1118 | 25.4426 | 28 | HN051 | HN058 |
|  |  |  |  |  |  | 1119 | 21.3923 | 28 | HN051 | HN059 |
|  |  |  |  |  |  | 1120 | 23.5596 | 28 | HN051 | HN060 |
|  |  |  |  |  |  | 1121 | 22.8568 | 28 | HN051 | HN061 |
|  |  |  |  |  |  | 1122 | 20.5445 | 26 | HN051 | HN064 |
|  |  |  |  |  |  | 1123 | 22.6045 | 26 | HN051 | HN066 |
|  |  |  |  |  |  | 1124 | 21.5995 | 24 | HN051 | HN072 |
|  |  |  |  |  |  | 1125 | 21.8232 | 26 | HN051 | HN079 |
|  |  |  |  |  |  | 1126 | 24.055 | 29 | HN051 | HN083 |
|  |  |  |  |  |  | 1127 | 22.8644 | 28 | HN051 | HN086 |
|  |  |  |  |  |  | 1128 | 19.5722 | 24 | HN051 | HN087 |
|  |  |  |  |  |  | 1129 | 23.1577 | 27 | HN051 | HN093 |
|  |  |  |  |  |  | 1130 | 26.591 | 31 | HN051 | HN096 |
|  |  |  |  |  |  | 1131 | 24.8101 | 30 | HN051 | HN105 |
|  |  |  |  |  |  | 1132 | 19.4077 | 28 | HN051 | HN112 |
|  |  |  |  |  |  | 1133 | 22.6817 | 26 | HN051 | HN112 |
|  |  |  |  |  |  | 1134 | 24.2511 | 28 | HN051 | HN118 |
|  |  |  |  |  |  | 1135 | 24.1911 | 28 | HN051 | HN127 |
|  |  |  |  |  |  | 1136 | 26.6021 | 31 | HN051 | HN129 |
|  |  |  |  |  |  | 1137 | 23.1814 | 29 | HN051 | HN137 |
|  |  |  |  |  |  | 1138 | 21.914 | 25 | HN051 | HN143 |
|  |  |  |  |  |  | 1139 | 21.0333 | 24 | HN051 | HN144 |
|  |  |  |  |  |  | 1140 | 21.9557 | 25 | HN052 | HN060 |
|  |  |  |  |  |  | 1141 | 21.3429 | 26 | HN052 | HN068 |
|  |  |  |  |  |  | 1142 | 20.2221 | 24 | HN052 | HN070 |
|  |  |  |  |  |  | 1143 | 19.5182 | 23 | HN052 | HN074 |
|  |  |  |  |  |  | 1144 | 17.2488 | 18 | HN052 | HN093 |
|  |  |  |  |  |  | 1145 | 20.7143 | 25 | HN052 | HN094 |
|  |  |  |  |  |  | 1146 | 17.3 | 22 | HN052 | HN102 |
|  |  |  |  |  |  | 1147 | 22.091 | 27 | HN052 | HN107 |
|  |  |  |  |  |  | 1148 | 24.4622 | 25 | HN052 | HN113 |
|  |  |  |  |  |  | 1149 | 22.3013 | 25 | HN052 | HN118 |
|  |  |  |  |  |  | 1150 | 20.2131 | 23 | HN052 | HN141 |
|  |  |  |  |  |  | 1151 | 18.4808 | 24 | HN052 | HN147 |
|  |  |  |  |  |  | 1152 | 19.842 | 26 | HN053 | HN057 |
|  |  |  |  |  |  | 1153 | 21.3309 | 24 | HN053 | HN058 |
|  |  |  |  |  |  | 1154 | 22.2634 | 26 | HN053 | HN067 |
|  |  |  |  |  |  | 1155 | 21.8211 | 26 | HN053 | HN068 |
|  |  |  |  |  |  | 1156 | 21.7602 | 25 | HN053 | HN069 |
|  |  |  |  |  |  | 1157 | 25.0511 | 28 | HN053 | HN080 |
|  |  |  |  |  |  | 1158 | 25.7347 | 27 | HN053 | HN097 |
|  |  |  |  |  |  | 1159 | 24.8307 | 22 | HN053 | HN106 |
|  |  |  |  |  |  | 1160 | 20.7581 | 18 | HN053 | HN106 |
|  |  |  |  |  |  | 1161 | 22.579 | 19 | HN053 | HN106 |
|  |  |  |  |  |  | 1162 | 16.6011 | 22 | HN053 | HN109 |
|  |  |  |  |  |  | 1163 | 24.1544 | 26 | HN053 | HN116 |
|  |  |  |  |  |  | 1164 | 25.0945 | 26 | HN053 | HN117 |
|  |  |  |  |  |  | 1165 | 23.27 | 27 | HN053 | HN122 |
|  |  |  |  |  |  | 1166 | 22.9934 | 24 | HN053 | HN123 |
|  |  |  |  |  |  | 1167 | 22.9197 | 24 | HN053 | HN123 |
|  |  |  |  |  |  | 1168 | 22.9934 | 24 | HN053 | HN123 |
|  |  |  |  |  |  | 1169 | 21.5181 | 24 | HN053 | HN124 |
|  |  |  |  |  |  | 1170 | 23.3067 | 29 | HN053 | HN125 |
|  |  |  |  |  |  | 1171 | 20.2662 | 27 | HN053 | HN127 |
|  |  |  |  |  |  | 1172 | 21.4056 | 29 | HN053 | HN131 |
|  |  |  |  |  |  | 1173 | 21.6341 | 24 | HN053 | HN137 |
|  |  |  |  |  |  | 1174 | 21.6264 | 22 | HN053 | HN148 |
|  |  |  |  |  |  | 1175 | 23.4586 | 27 | HN054 | HN065 |
|  |  |  |  |  |  | 1176 | 23.179 | 24 | HN054 | HN068 |
|  |  |  |  |  |  | 1177 | 20.5511 | 27 | HN054 | HN069 |
|  |  |  |  |  |  | 1178 | 20.6216 | 23 | HN054 | HN073 |
|  |  |  |  |  |  | 1179 | 20.3887 | 26 | HN054 | HN079 |
|  |  |  |  |  |  | 1180 | 19.903 | 25 | HN054 | HN081 |
|  |  |  |  |  |  | 1181 | 20.9466 | 22 | HN054 | HN085 |
|  |  |  |  |  |  | 1182 | 22.1172 | 27 | HN054 | HN103 |
|  |  |  |  |  |  | 1183 | 21.7672 | 22 | HN054 | HN104 |
|  |  |  |  |  |  | 1184 | 20.005 | 23 | HN054 | HN108 |
|  |  |  |  |  |  | 1185 | 21.9483 | 25 | HN054 | HN113 |
|  |  |  |  |  |  | 1186 | 19.8833 | 25 | HN054 | HN129 |
|  |  |  |  |  |  | 1187 | 24.7885 | 30 | HN054 | HN129 |
|  |  |  |  |  |  | 1188 | 25.8058 | 30 | HN054 | HN131 |
|  |  |  |  |  |  | 1189 | 24.8921 | 31 | HN054 | HN131 |
|  |  |  |  |  |  | 1190 | 23.9138 | 31 | HN055 | HN061 |
|  |  |  |  |  |  | 1191 | 20.2452 | 26 | HN055 | HN062 |
|  |  |  |  |  |  | 1192 | 21.1828 | 27 | HN055 | HN064 |
|  |  |  |  |  |  | 1193 | 25.2318 | 27 | HN055 | HN065 |
|  |  |  |  |  |  | 1194 | 21.4488 | 25 | HN055 | HN067 |
|  |  |  |  |  |  | 1195 | 21.9871 | 25 | HN055 | HN068 |
|  |  |  |  |  |  | 1196 | 19.7981 | 26 | HN055 | HN072 |
|  |  |  |  |  |  | 1197 | 21.2934 | 27 | HN055 | HN079 |
|  |  |  |  |  |  | 1198 | 26.2656 | 28 | HN055 | HN080 |
|  |  |  |  |  |  | 1199 | 21.5408 | 28 | HN055 | HN090 |
|  |  |  |  |  |  | 1200 | 19.6645 | 27 | HN055 | HN091 |
|  |  |  |  |  |  | 1201 | 23.0608 | 26 | HN055 | HN109 |
|  |  |  |  |  |  | 1202 | 22.7972 | 29 | HN055 | HN111 |
|  |  |  |  |  |  | 1203 | 27.5536 | 29 | HN055 | HN113 |
|  |  |  |  |  |  | 1204 | 26.1723 | 27 | HN055 | HN116 |
|  |  |  |  |  |  | 1205 | 24.3784 | 28 | HN055 | HN131 |
|  |  |  |  |  |  | 1206 | 22.0317 | 25 | HN055 | HN137 |
|  |  |  |  |  |  | 1207 | 23.7133 | 30 | HN055 | HN147 |
|  |  |  |  |  |  | 1208 | 17.6778 | 23 | HN056 | HN057 |
|  |  |  |  |  |  | 1209 | 20.2769 | 27 | HN056 | HN059 |
|  |  |  |  |  |  | 1210 | 22.0105 | 29 | HN056 | HN068 |
|  |  |  |  |  |  | 1211 | 24.2047 | 30 | HN056 | HN073 |
|  |  |  |  |  |  | 1212 | 18.5694 | 22 | HN056 | HN081 |
|  |  |  |  |  |  | 1213 | 24.9814 | 28 | HN056 | HN082 |
|  |  |  |  |  |  | 1214 | 25.4375 | 28 | HN056 | HN082 |
|  |  |  |  |  |  | 1215 | 17.8517 | 23 | HN056 | HN084 |
|  |  |  |  |  |  | 1216 | 21.5485 | 25 | HN056 | HN105 |
|  |  |  |  |  |  | 1217 | 17.8242 | 27 | HN056 | HN107 |
|  |  |  |  |  |  | 1218 | 22.3625 | 25 | HN056 | HN112 |
|  |  |  |  |  |  | 1219 | 23.5825 | 30 | HN056 | HN129 |
|  |  |  |  |  |  | 1220 | 17.6128 | 20 | HN056 | HN133 |
|  |  |  |  |  |  | 1221 | 21.6769 | 25 | HN056 | HN137 |
|  |  |  |  |  |  | 1222 | 15.1987 | 23 | HN056 | HN147 |
|  |  |  |  |  |  | 1223 | 20.1777 | 21 | HN056 | HN149 |
|  |  |  |  |  |  | 1224 | 23.9478 | 26 | HN057 | HN058 |
|  |  |  |  |  |  | 1225 | 20.8617 | 25 | HN057 | HN077 |
|  |  |  |  |  |  | 1226 | 22.1009 | 26 | HN057 | HN103 |
|  |  |  |  |  |  | 1227 | 24.3111 | 31 | HN057 | HN103 |
|  |  |  |  |  |  | 1228 | 25.744 | 24 | HN057 | HN104 |
|  |  |  |  |  |  | 1229 | 23.0748 | 27 | HN057 | HN109 |
|  |  |  |  |  |  | 1230 | 22.6333 | 25 | HN057 | HN116 |
|  |  |  |  |  |  | 1231 | 18.5125 | 21 | HN057 | HN149 |
|  |  |  |  |  |  | 1232 | 21.051 | 26 | HN058 | HN067 |
|  |  |  |  |  |  | 1233 | 22.6811 | 23 | HN058 | HN082 |
|  |  |  |  |  |  | 1234 | 24.0166 | 27 | HN058 | HN090 |
|  |  |  |  |  |  | 1235 | 22.2486 | 24 | HN058 | HN090 |
|  |  |  |  |  |  | 1236 | 25.1804 | 29 | HN058 | HN096 |
|  |  |  |  |  |  | 1237 | 26.6089 | 25 | HN058 | HN097 |
|  |  |  |  |  |  | 1238 | 23.279 | 27 | HN058 | HN102 |
|  |  |  |  |  |  | 1239 | 19.4885 | 22 | HN058 | HN107 |
|  |  |  |  |  |  | 1240 | 19.0129 | 20 | HN058 | HN108 |
|  |  |  |  |  |  | 1241 | 19.6712 | 23 | HN058 | HN109 |
|  |  |  |  |  |  | 1242 | 21.3194 | 23 | HN058 | HN111 |
|  |  |  |  |  |  | 1243 | 27.9997 | 30 | HN058 | HN116 |
|  |  |  |  |  |  | 1244 | 25.6023 | 28 | HN058 | HN127 |
|  |  |  |  |  |  | 1245 | 19.1296 | 23 | HN058 | HN131 |
|  |  |  |  |  |  | 1246 | 19.4491 | 21 | HN058 | HN147 |
|  |  |  |  |  |  | 1247 | 22.186 | 27 | HN059 | HN068 |
|  |  |  |  |  |  | 1248 | 24.3259 | 28 | HN059 | HN073 |
|  |  |  |  |  |  | 1249 | 25.1124 | 28 | HN059 | HN082 |
|  |  |  |  |  |  | 1250 | 22.6887 | 29 | HN059 | HN083 |
|  |  |  |  |  |  | 1251 | 18.4952 | 22 | HN059 | HN087 |
|  |  |  |  |  |  | 1252 | 20.6246 | 16 | HN059 | HN097 |
|  |  |  |  |  |  | 1253 | 22.427 | 28 | HN059 | HN129 |
|  |  |  |  |  |  | 1254 | 22.4041 | 27 | HN059 | HN130 |
|  |  |  |  |  |  | 1255 | 23.4795 | 28 | HN059 | HN131 |
|  |  |  |  |  |  | 1256 | 19.4757 | 24 | HN059 | HN132 |
|  |  |  |  |  |  | 1257 | 18.3017 | 23 | HN059 | HN144 |
|  |  |  |  |  |  | 1258 | 20.0994 | 22 | HN059 | HN148 |
|  |  |  |  |  |  | 1259 | 24.3092 | 27 | HN060 | HN064 |
|  |  |  |  |  |  | 1260 | 22.9546 | 26 | HN060 | HN064 |
|  |  |  |  |  |  | 1261 | 20.0872 | 24 | HN060 | HN077 |
|  |  |  |  |  |  | 1262 | 23.2104 | 26 | HN060 | HN080 |
|  |  |  |  |  |  | 1263 | 26.0491 | 27 | HN060 | HN082 |
|  |  |  |  |  |  | 1264 | 21.6847 | 26 | HN060 | HN090 |
|  |  |  |  |  |  | 1265 | 19.1769 | 23 | HN060 | HN091 |
|  |  |  |  |  |  | 1266 | 22.8877 | 28 | HN060 | HN093 |
|  |  |  |  |  |  | 1267 | 22.0932 | 25 | HN060 | HN095 |
|  |  |  |  |  |  | 1268 | 21.8478 | 27 | HN060 | HN096 |
|  |  |  |  |  |  | 1269 | 23.9391 | 29 | HN060 | HN103 |
|  |  |  |  |  |  | 1270 | 26.4751 | 32 | HN060 | HN105 |
|  |  |  |  |  |  | 1271 | 21.4606 | 19 | HN060 | HN106 |
|  |  |  |  |  |  | 1272 | 22.7514 | 26 | HN060 | HN110 |
|  |  |  |  |  |  | 1273 | 23.514 | 27 | HN060 | HN110 |
|  |  |  |  |  |  | 1274 | 25.5972 | 27 | HN060 | HN116 |
|  |  |  |  |  |  | 1275 | 18.1587 | 24 | HN060 | HN119 |
|  |  |  |  |  |  | 1276 | 25.794 | 28 | HN060 | HN123 |
|  |  |  |  |  |  | 1277 | 25.5948 | 30 | HN060 | HN125 |
|  |  |  |  |  |  | 1278 | 22.7662 | 29 | HN060 | HN130 |
|  |  |  |  |  |  | 1279 | 23.1858 | 27 | HN060 | HN130 |
|  |  |  |  |  |  | 1280 | 24.5383 | 29 | HN060 | HN131 |
|  |  |  |  |  |  | 1281 | 18.1332 | 23 | HN060 | HN135 |
|  |  |  |  |  |  | 1282 | 23.4902 | 27 | HN060 | HN137 |
|  |  |  |  |  |  | 1283 | 23.3522 | 29 | HN060 | HN138 |
|  |  |  |  |  |  | 1284 | 20.7404 | 26 | HN060 | HN140 |
|  |  |  |  |  |  | 1285 | 19.8253 | 24 | HN060 | HN141 |
|  |  |  |  |  |  | 1286 | 18.4293 | 21 | HN060 | HN141 |
|  |  |  |  |  |  | 1287 | 24.092 | 25 | HN061 | HN066 |
|  |  |  |  |  |  | 1288 | 22.0621 | 22 | HN061 | HN074 |
|  |  |  |  |  |  | 1289 | 24.6408 | 25 | HN061 | HN075 |
|  |  |  |  |  |  | 1290 | 28.6658 | 30 | HN061 | HN082 |
|  |  |  |  |  |  | 1291 | 26.8303 | 27 | HN061 | HN082 |
|  |  |  |  |  |  | 1292 | 24.0561 | 24 | HN061 | HN084 |
|  |  |  |  |  |  | 1293 | 23.1343 | 25 | HN061 | HN093 |
|  |  |  |  |  |  | 1294 | 22.9223 | 25 | HN061 | HN094 |
|  |  |  |  |  |  | 1295 | 23.4341 | 25 | HN061 | HN094 |
|  |  |  |  |  |  | 1296 | 22.9837 | 29 | HN061 | HN096 |
|  |  |  |  |  |  | 1297 | 23.7973 | 24 | HN061 | HN097 |
|  |  |  |  |  |  | 1298 | 19.9653 | 26 | HN061 | HN105 |
|  |  |  |  |  |  | 1299 | 24.3844 | 26 | HN061 | HN111 |
|  |  |  |  |  |  | 1300 | 25.3284 | 28 | HN061 | HN112 |
|  |  |  |  |  |  | 1301 | 26.7844 | 26 | HN061 | HN113 |
|  |  |  |  |  |  | 1302 | 24.8364 | 24 | HN061 | HN113 |
|  |  |  |  |  |  | 1303 | 24.2234 | 26 | HN061 | HN116 |
|  |  |  |  |  |  | 1304 | 21.046 | 24 | HN061 | HN122 |
|  |  |  |  |  |  | 1305 | 22.8257 | 24 | HN061 | HN131 |
|  |  |  |  |  |  | 1306 | 21.6155 | 24 | HN061 | HN132 |
|  |  |  |  |  |  | 1307 | 22.1751 | 22 | HN061 | HN138 |
|  |  |  |  |  |  | 1308 | 23.9232 | 24 | HN061 | HN139 |
|  |  |  |  |  |  | 1309 | 23.5494 | 23 | HN061 | HN139 |
|  |  |  |  |  |  | 1310 | 19.4099 | 23 | HN061 | HN141 |
|  |  |  |  |  |  | 1311 | 22.3113 | 25 | HN061 | HN147 |
|  |  |  |  |  |  | 1312 | 24.293 | 26 | HN061 | HN149 |
|  |  |  |  |  |  | 1313 | 20.3157 | 23 | HN062 | HN065 |
|  |  |  |  |  |  | 1314 | 21.8712 | 24 | HN062 | HN067 |
|  |  |  |  |  |  | 1315 | 19.2873 | 23 | HN062 | HN068 |
|  |  |  |  |  |  | 1316 | 22.6297 | 24 | HN062 | HN082 |
|  |  |  |  |  |  | 1317 | 25.1378 | 27 | HN062 | HN082 |
|  |  |  |  |  |  | 1318 | 17.6424 | 21 | HN062 | HN087 |
|  |  |  |  |  |  | 1319 | 22.2819 | 26 | HN062 | HN131 |
|  |  |  |  |  |  | 1320 | 20.3125 | 25 | HN062 | HN131 |
|  |  |  |  |  |  | 1321 | 21.8907 | 23 | HN062 | HN143 |
|  |  |  |  |  |  | 1322 | 21.9176 | 27 | HN063 | HN077 |
|  |  |  |  |  |  | 1323 | 23.2831 | 27 | HN063 | HN080 |
|  |  |  |  |  |  | 1324 | 16.9492 | 23 | HN063 | HN085 |
|  |  |  |  |  |  | 1325 | 16.8914 | 24 | HN063 | HN095 |
|  |  |  |  |  |  | 1326 | 20.8764 | 25 | HN063 | HN107 |
|  |  |  |  |  |  | 1327 | 23.001 | 26 | HN063 | HN118 |
|  |  |  |  |  |  | 1328 | 17.8685 | 24 | HN063 | HN136 |
|  |  |  |  |  |  | 1329 | 21.6431 | 24 | HN064 | HN066 |
|  |  |  |  |  |  | 1330 | 26.1935 | 27 | HN064 | HN068 |
|  |  |  |  |  |  | 1331 | 24.7951 | 27 | HN064 | HN068 |
|  |  |  |  |  |  | 1332 | 25.2016 | 28 | HN064 | HN069 |
|  |  |  |  |  |  | 1333 | 23.709 | 27 | HN064 | HN070 |
|  |  |  |  |  |  | 1334 | 24.1974 | 27 | HN064 | HN072 |
|  |  |  |  |  |  | 1335 | 23.8758 | 26 | HN064 | HN073 |
|  |  |  |  |  |  | 1336 | 27.2367 | 28 | HN064 | HN073 |
|  |  |  |  |  |  | 1337 | 23.5882 | 26 | HN064 | HN075 |
|  |  |  |  |  |  | 1338 | 22.5417 | 24 | HN064 | HN075 |
|  |  |  |  |  |  | 1339 | 23.5882 | 26 | HN064 | HN075 |
|  |  |  |  |  |  | 1340 | 25.9099 | 26 | HN064 | HN080 |
|  |  |  |  |  |  | 1341 | 21.011 | 24 | HN064 | HN083 |
|  |  |  |  |  |  | 1342 | 22.286 | 22 | HN064 | HN092 |
|  |  |  |  |  |  | 1343 | 23.0117 | 28 | HN064 | HN094 |
|  |  |  |  |  |  | 1344 | 22.6308 | 21 | HN064 | HN097 |
|  |  |  |  |  |  | 1345 | 21.9919 | 25 | HN064 | HN098 |
|  |  |  |  |  |  | 1346 | 18.1556 | 25 | HN064 | HN105 |
|  |  |  |  |  |  | 1347 | 17.7236 | 24 | HN064 | HN105 |
|  |  |  |  |  |  | 1348 | 21.6996 | 26 | HN064 | HN108 |
|  |  |  |  |  |  | 1349 | 22.814 | 25 | HN064 | HN124 |
|  |  |  |  |  |  | 1350 | 20.5224 | 24 | HN064 | HN138 |
|  |  |  |  |  |  | 1351 | 23.1608 | 24 | HN064 | HN139 |
|  |  |  |  |  |  | 1352 | 23.9078 | 25 | HN064 | HN140 |
|  |  |  |  |  |  | 1353 | 21.3968 | 26 | HN064 | HN143 |
|  |  |  |  |  |  | 1354 | 21.7501 | 26 | HN064 | HN146 |
|  |  |  |  |  |  | 1355 | 22.6222 | 24 | HN064 | HN147 |
|  |  |  |  |  |  | 1356 | 22.4949 | 25 | HN065 | HN066 |
|  |  |  |  |  |  | 1357 | 25.9216 | 28 | HN065 | HN066 |
|  |  |  |  |  |  | 1358 | 24.1003 | 30 | HN065 | HN083 |
|  |  |  |  |  |  | 1359 | 22.5878 | 26 | HN065 | HN088 |
|  |  |  |  |  |  | 1360 | 22.6758 | 28 | HN065 | HN090 |
|  |  |  |  |  |  | 1361 | 22.4084 | 28 | HN065 | HN090 |
|  |  |  |  |  |  | 1362 | 23.7058 | 27 | HN065 | HN098 |
|  |  |  |  |  |  | 1363 | 21.485 | 26 | HN065 | HN107 |
|  |  |  |  |  |  | 1364 | 22.0406 | 25 | HN065 | HN108 |
|  |  |  |  |  |  | 1365 | 21.7419 | 26 | HN065 | HN113 |
|  |  |  |  |  |  | 1366 | 22.8836 | 25 | HN065 | HN113 |
|  |  |  |  |  |  | 1367 | 22.3407 | 28 | HN065 | HN115 |
|  |  |  |  |  |  | 1368 | 22.3973 | 23 | HN065 | HN123 |
|  |  |  |  |  |  | 1369 | 21.7361 | 27 | HN065 | HN125 |
|  |  |  |  |  |  | 1370 | 21.7361 | 27 | HN065 | HN125 |
|  |  |  |  |  |  | 1371 | 20.3101 | 27 | HN065 | HN128 |
|  |  |  |  |  |  | 1372 | 25.0744 | 30 | HN065 | HN131 |
|  |  |  |  |  |  | 1373 | 21.5903 | 26 | HN065 | HN133 |
|  |  |  |  |  |  | 1374 | 24.2797 | 28 | HN065 | HN133 |
|  |  |  |  |  |  | 1375 | 19.7981 | 24 | HN065 | HN135 |
|  |  |  |  |  |  | 1376 | 21.4823 | 26 | HN065 | HN139 |
|  |  |  |  |  |  | 1377 | 19.4939 | 20 | HN066 | HN067 |
|  |  |  |  |  |  | 1378 | 24.2298 | 23 | HN066 | HN068 |
|  |  |  |  |  |  | 1379 | 24.1486 | 24 | HN066 | HN072 |
|  |  |  |  |  |  | 1380 | 21.9182 | 26 | HN066 | HN082 |
|  |  |  |  |  |  | 1381 | 22.3378 | 23 | HN066 | HN085 |
|  |  |  |  |  |  | 1382 | 25.4151 | 24 | HN066 | HN093 |
|  |  |  |  |  |  | 1383 | 18.4533 | 27 | HN066 | HN105 |
|  |  |  |  |  |  | 1384 | 22.383 | 23 | HN066 | HN122 |
|  |  |  |  |  |  | 1385 | 18.8662 | 21 | HN066 | HN132 |
|  |  |  |  |  |  | 1386 | 19.1112 | 21 | HN066 | HN137 |
|  |  |  |  |  |  | 1387 | 22.0766 | 23 | HN066 | HN137 |
|  |  |  |  |  |  | 1388 | 22.7276 | 26 | HN066 | HN147 |
|  |  |  |  |  |  | 1389 | 20.6121 | 25 | HN067 | HN070 |
|  |  |  |  |  |  | 1390 | 21.2276 | 22 | HN067 | HN076 |
|  |  |  |  |  |  | 1391 | 21.8746 | 26 | HN067 | HN079 |
|  |  |  |  |  |  | 1392 | 19.6968 | 21 | HN067 | HN081 |
|  |  |  |  |  |  | 1393 | 19.6968 | 21 | HN067 | HN081 |
|  |  |  |  |  |  | 1394 | 23.2388 | 28 | HN067 | HN083 |
|  |  |  |  |  |  | 1395 | 24.7032 | 27 | HN067 | HN103 |
|  |  |  |  |  |  | 1396 | 24.3219 | 20 | HN067 | HN106 |
|  |  |  |  |  |  | 1397 | 21.7551 | 25 | HN067 | HN108 |
|  |  |  |  |  |  | 1398 | 24.3944 | 28 | HN067 | HN112 |
|  |  |  |  |  |  | 1399 | 24.842 | 25 | HN067 | HN113 |
|  |  |  |  |  |  | 1400 | 22.1513 | 25 | HN067 | HN123 |
|  |  |  |  |  |  | 1401 | 27.2944 | 30 | HN067 | HN125 |
|  |  |  |  |  |  | 1402 | 24.5294 | 30 | HN067 | HN129 |
|  |  |  |  |  |  | 1403 | 19.6889 | 22 | HN067 | HN145 |
|  |  |  |  |  |  | 1404 | 17.811 | 21 | HN067 | HN150 |
|  |  |  |  |  |  | 1405 | 21.0984 | 24 | HN068 | HN074 |
|  |  |  |  |  |  | 1406 | 23.6422 | 27 | HN068 | HN079 |
|  |  |  |  |  |  | 1407 | 23.3301 | 23 | HN068 | HN081 |
|  |  |  |  |  |  | 1408 | 24.9836 | 27 | HN068 | HN082 |
|  |  |  |  |  |  | 1409 | 23.6284 | 29 | HN068 | HN084 |
|  |  |  |  |  |  | 1410 | 22.8866 | 25 | HN068 | HN088 |
|  |  |  |  |  |  | 1411 | 27.7077 | 29 | HN068 | HN090 |
|  |  |  |  |  |  | 1412 | 28.2042 | 29 | HN068 | HN090 |
|  |  |  |  |  |  | 1413 | 23.3304 | 24 | HN068 | HN096 |
|  |  |  |  |  |  | 1414 | 22.106 | 22 | HN068 | HN097 |
|  |  |  |  |  |  | 1415 | 24.0311 | 26 | HN068 | HN104 |
|  |  |  |  |  |  | 1416 | 23.6452 | 24 | HN068 | HN104 |
|  |  |  |  |  |  | 1417 | 22.7468 | 24 | HN068 | HN104 |
|  |  |  |  |  |  | 1418 | 21.1219 | 24 | HN068 | HN108 |
|  |  |  |  |  |  | 1419 | 19.3081 | 22 | HN068 | HN109 |
|  |  |  |  |  |  | 1420 | 20.7093 | 26 | HN068 | HN115 |
|  |  |  |  |  |  | 1421 | 24.1769 | 24 | HN068 | HN116 |
|  |  |  |  |  |  | 1422 | 23.4636 | 23 | HN068 | HN123 |
|  |  |  |  |  |  | 1423 | 22.5672 | 25 | HN068 | HN124 |
|  |  |  |  |  |  | 1424 | 23.6648 | 27 | HN068 | HN124 |
|  |  |  |  |  |  | 1425 | 20.5588 | 27 | HN068 | HN127 |
|  |  |  |  |  |  | 1426 | 22.7774 | 27 | HN068 | HN127 |
|  |  |  |  |  |  | 1427 | 22.1835 | 30 | HN068 | HN128 |
|  |  |  |  |  |  | 1428 | 25.8116 | 27 | HN068 | HN129 |
|  |  |  |  |  |  | 1429 | 22.3942 | 27 | HN068 | HN132 |
|  |  |  |  |  |  | 1430 | 24.7128 | 26 | HN068 | HN132 |
|  |  |  |  |  |  | 1431 | 24.0442 | 27 | HN068 | HN134 |
|  |  |  |  |  |  | 1432 | 22.2265 | 25 | HN068 | HN135 |
|  |  |  |  |  |  | 1433 | 20.1732 | 26 | HN068 | HN136 |
|  |  |  |  |  |  | 1434 | 24.9845 | 28 | HN068 | HN137 |
|  |  |  |  |  |  | 1435 | 24.0187 | 29 | HN069 | HN073 |
|  |  |  |  |  |  | 1436 | 28.0434 | 30 | HN069 | HN082 |
|  |  |  |  |  |  | 1437 | 23.9003 | 28 | HN069 | HN082 |
|  |  |  |  |  |  | 1438 | 25.0587 | 31 | HN069 | HN083 |
|  |  |  |  |  |  | 1439 | 19.8436 | 22 | HN069 | HN086 |
|  |  |  |  |  |  | 1440 | 22.3909 | 27 | HN069 | HN098 |
|  |  |  |  |  |  | 1441 | 22.4183 | 26 | HN069 | HN101 |
|  |  |  |  |  |  | 1442 | 19.6183 | 22 | HN069 | HN102 |
|  |  |  |  |  |  | 1443 | 23.3176 | 29 | HN069 | HN103 |
|  |  |  |  |  |  | 1444 | 20.5019 | 18 | HN069 | HN104 |
|  |  |  |  |  |  | 1445 | 20.5059 | 19 | HN069 | HN104 |
|  |  |  |  |  |  | 1446 | 23.5922 | 27 | HN069 | HN107 |
|  |  |  |  |  |  | 1447 | 23.9547 | 27 | HN069 | HN107 |
|  |  |  |  |  |  | 1448 | 22.559 | 25 | HN069 | HN108 |
|  |  |  |  |  |  | 1449 | 18.0411 | 22 | HN069 | HN110 |
|  |  |  |  |  |  | 1450 | 24.7284 | 24 | HN069 | HN111 |
|  |  |  |  |  |  | 1451 | 21.8335 | 28 | HN069 | HN115 |
|  |  |  |  |  |  | 1452 | 24.3907 | 27 | HN069 | HN116 |
|  |  |  |  |  |  | 1453 | 25.8901 | 28 | HN069 | HN116 |
|  |  |  |  |  |  | 1454 | 23.9992 | 26 | HN069 | HN118 |
|  |  |  |  |  |  | 1455 | 24.373 | 27 | HN069 | HN118 |
|  |  |  |  |  |  | 1456 | 20.3068 | 25 | HN069 | HN120 |
|  |  |  |  |  |  | 1457 | 26.2172 | 30 | HN069 | HN125 |
|  |  |  |  |  |  | 1458 | 23.1261 | 24 | HN069 | HN125 |
|  |  |  |  |  |  | 1459 | 18.2918 | 24 | HN069 | HN127 |
|  |  |  |  |  |  | 1460 | 22.071 | 28 | HN069 | HN128 |
|  |  |  |  |  |  | 1461 | 25.2601 | 29 | HN069 | HN129 |
|  |  |  |  |  |  | 1462 | 21.5539 | 24 | HN069 | HN132 |
|  |  |  |  |  |  | 1463 | 18.5392 | 21 | HN069 | HN132 |
|  |  |  |  |  |  | 1464 | 17.6999 | 19 | HN069 | HN135 |
|  |  |  |  |  |  | 1465 | 19.7436 | 23 | HN069 | HN147 |
|  |  |  |  |  |  | 1466 | 20.2387 | 24 | HN069 | HN149 |
|  |  |  |  |  |  | 1467 | 25.0104 | 25 | HN070 | HN073 |
|  |  |  |  |  |  | 1468 | 25.6865 | 27 | HN070 | HN073 |
|  |  |  |  |  |  | 1469 | 22.974 | 23 | HN070 | HN087 |
|  |  |  |  |  |  | 1470 | 19.3985 | 21 | HN070 | HN092 |
|  |  |  |  |  |  | 1471 | 23.5731 | 23 | HN070 | HN094 |
|  |  |  |  |  |  | 1472 | 20.8972 | 23 | HN070 | HN101 |
|  |  |  |  |  |  | 1473 | 21.5766 | 24 | HN070 | HN101 |
|  |  |  |  |  |  | 1474 | 21.7842 | 22 | HN070 | HN103 |
|  |  |  |  |  |  | 1475 | 18.4988 | 20 | HN070 | HN104 |
|  |  |  |  |  |  | 1476 | 24.1233 | 29 | HN070 | HN109 |
|  |  |  |  |  |  | 1477 | 22.338 | 26 | HN070 | HN109 |
|  |  |  |  |  |  | 1478 | 22.4069 | 27 | HN070 | HN109 |
|  |  |  |  |  |  | 1479 | 21.9811 | 21 | HN070 | HN116 |
|  |  |  |  |  |  | 1480 | 23.892 | 24 | HN070 | HN118 |
|  |  |  |  |  |  | 1481 | 25.6456 | 25 | HN070 | HN118 |
|  |  |  |  |  |  | 1482 | 19.1012 | 20 | HN070 | HN126 |
|  |  |  |  |  |  | 1483 | 21.1513 | 24 | HN070 | HN127 |
|  |  |  |  |  |  | 1484 | 24.3277 | 28 | HN070 | HN131 |
|  |  |  |  |  |  | 1485 | 18.7285 | 20 | HN070 | HN149 |
|  |  |  |  |  |  | 1486 | 26.7344 | 33 | HN071 | HN083 |
|  |  |  |  |  |  | 1487 | 21.3992 | 26 | HN071 | HN090 |
|  |  |  |  |  |  | 1488 | 22.1005 | 27 | HN071 | HN096 |
|  |  |  |  |  |  | 1489 | 21.2923 | 19 | HN071 | HN106 |
|  |  |  |  |  |  | 1490 | 22.437 | 25 | HN071 | HN112 |
|  |  |  |  |  |  | 1491 | 21.5167 | 26 | HN071 | HN112 |
|  |  |  |  |  |  | 1492 | 23.4936 | 24 | HN071 | HN123 |
|  |  |  |  |  |  | 1493 | 23.0622 | 30 | HN071 | HN129 |
|  |  |  |  |  |  | 1494 | 20.324 | 22 | HN071 | HN136 |
|  |  |  |  |  |  | 1495 | 22.5918 | 25 | HN071 | HN143 |
|  |  |  |  |  |  | 1496 | 17.8225 | 22 | HN071 | HN150 |
|  |  |  |  |  |  | 1497 | 23.2236 | 26 | HN072 | HN073 |
|  |  |  |  |  |  | 1498 | 25.8098 | 29 | HN072 | HN073 |
|  |  |  |  |  |  | 1499 | 26.699 | 28 | HN072 | HN082 |
|  |  |  |  |  |  | 1500 | 23.3773 | 27 | HN072 | HN094 |
|  |  |  |  |  |  | 1501 | 23.7322 | 18 | HN072 | HN106 |
|  |  |  |  |  |  | 1502 | 23.663 | 19 | HN072 | HN106 |
|  |  |  |  |  |  | 1503 | 22.3713 | 23 | HN072 | HN114 |
|  |  |  |  |  |  | 1504 | 22.8903 | 29 | HN072 | HN125 |
|  |  |  |  |  |  | 1505 | 26.0249 | 29 | HN072 | HN131 |
|  |  |  |  |  |  | 1506 | 21.248 | 25 | HN072 | HN132 |
|  |  |  |  |  |  | 1507 | 22.5504 | 26 | HN073 | HN075 |
|  |  |  |  |  |  | 1508 | 29.1008 | 29 | HN073 | HN082 |
|  |  |  |  |  |  | 1509 | 25.1104 | 27 | HN073 | HN091 |
|  |  |  |  |  |  | 1510 | 25.0016 | 23 | HN073 | HN097 |
|  |  |  |  |  |  | 1511 | 27.5012 | 23 | HN073 | HN097 |
|  |  |  |  |  |  | 1512 | 25.3055 | 28 | HN073 | HN098 |
|  |  |  |  |  |  | 1513 | 22.5093 | 28 | HN073 | HN098 |
|  |  |  |  |  |  | 1514 | 22.4329 | 24 | HN073 | HN101 |
|  |  |  |  |  |  | 1515 | 24.5547 | 26 | HN073 | HN101 |
|  |  |  |  |  |  | 1516 | 23.7381 | 24 | HN073 | HN101 |
|  |  |  |  |  |  | 1517 | 23.5943 | 27 | HN073 | HN102 |
|  |  |  |  |  |  | 1518 | 22.1003 | 27 | HN073 | HN103 |
|  |  |  |  |  |  | 1519 | 24.4839 | 25 | HN073 | HN104 |
|  |  |  |  |  |  | 1520 | 23.4673 | 21 | HN073 | HN106 |
|  |  |  |  |  |  | 1521 | 26.1835 | 25 | HN073 | HN106 |
|  |  |  |  |  |  | 1522 | 24.6117 | 24 | HN073 | HN108 |
|  |  |  |  |  |  | 1523 | 20.934 | 24 | HN073 | HN109 |
|  |  |  |  |  |  | 1524 | 25.7066 | 24 | HN073 | HN113 |
|  |  |  |  |  |  | 1525 | 22.4961 | 23 | HN073 | HN114 |
|  |  |  |  |  |  | 1526 | 23.7202 | 25 | HN073 | HN117 |
|  |  |  |  |  |  | 1527 | 22.9753 | 25 | HN073 | HN117 |
|  |  |  |  |  |  | 1528 | 25.066 | 28 | HN073 | HN121 |
|  |  |  |  |  |  | 1529 | 23.4959 | 25 | HN073 | HN122 |
|  |  |  |  |  |  | 1530 | 26.2422 | 29 | HN073 | HN123 |
|  |  |  |  |  |  | 1531 | 24.0939 | 26 | HN073 | HN124 |
|  |  |  |  |  |  | 1532 | 25.3178 | 25 | HN073 | HN125 |
|  |  |  |  |  |  | 1533 | 27.7701 | 28 | HN073 | HN131 |
|  |  |  |  |  |  | 1534 | 24.1232 | 26 | HN073 | HN135 |
|  |  |  |  |  |  | 1535 | 24.5055 | 27 | HN073 | HN138 |
|  |  |  |  |  |  | 1536 | 27.6411 | 26 | HN073 | HN141 |
|  |  |  |  |  |  | 1537 | 23.8329 | 26 | HN073 | HN143 |
|  |  |  |  |  |  | 1538 | 22.732 | 26 | HN073 | HN143 |
|  |  |  |  |  |  | 1539 | 16.8002 | 21 | HN074 | HN081 |
|  |  |  |  |  |  | 1540 | 21.8424 | 24 | HN074 | HN090 |
|  |  |  |  |  |  | 1541 | 21.8152 | 25 | HN074 | HN090 |
|  |  |  |  |  |  | 1542 | 20.958 | 26 | HN074 | HN094 |
|  |  |  |  |  |  | 1543 | 19.2538 | 24 | HN074 | HN098 |
|  |  |  |  |  |  | 1544 | 22.6672 | 25 | HN074 | HN108 |
|  |  |  |  |  |  | 1545 | 15.804 | 22 | HN074 | HN109 |
|  |  |  |  |  |  | 1546 | 18.2772 | 27 | HN074 | HN112 |
|  |  |  |  |  |  | 1547 | 19.8165 | 24 | HN074 | HN120 |
|  |  |  |  |  |  | 1548 | 21.9567 | 26 | HN074 | HN125 |
|  |  |  |  |  |  | 1549 | 21.3151 | 25 | HN074 | HN136 |
|  |  |  |  |  |  | 1550 | 20.8854 | 23 | HN074 | HN140 |
|  |  |  |  |  |  | 1551 | 23.3379 | 23 | HN075 | HN080 |
|  |  |  |  |  |  | 1552 | 23.2015 | 29 | HN075 | HN083 |
|  |  |  |  |  |  | 1553 | 24.7572 | 30 | HN075 | HN083 |
|  |  |  |  |  |  | 1554 | 19.2915 | 22 | HN075 | HN087 |
|  |  |  |  |  |  | 1555 | 20.1747 | 25 | HN075 | HN107 |
|  |  |  |  |  |  | 1556 | 23.0492 | 25 | HN075 | HN112 |
|  |  |  |  |  |  | 1557 | 27.0181 | 28 | HN075 | HN116 |
|  |  |  |  |  |  | 1558 | 22.7374 | 26 | HN075 | HN120 |
|  |  |  |  |  |  | 1559 | 18.8065 | 24 | HN075 | HN121 |
|  |  |  |  |  |  | 1560 | 22.6358 | 25 | HN075 | HN126 |
|  |  |  |  |  |  | 1561 | 21.9716 | 25 | HN075 | HN126 |
|  |  |  |  |  |  | 1562 | 22.4891 | 26 | HN075 | HN127 |
|  |  |  |  |  |  | 1563 | 20.8295 | 24 | HN075 | HN146 |
|  |  |  |  |  |  | 1564 | 23.9253 | 22 | HN076 | HN082 |
|  |  |  |  |  |  | 1565 | 20.1774 | 23 | HN076 | HN085 |
|  |  |  |  |  |  | 1566 | 24.2858 | 24 | HN076 | HN094 |
|  |  |  |  |  |  | 1567 | 22.4452 | 29 | HN076 | HN096 |
|  |  |  |  |  |  | 1568 | 17.6114 | 22 | HN076 | HN112 |
|  |  |  |  |  |  | 1569 | 19.2851 | 23 | HN076 | HN119 |
|  |  |  |  |  |  | 1570 | 21.5728 | 24 | HN076 | HN126 |
|  |  |  |  |  |  | 1571 | 16.8402 | 20 | HN076 | HN137 |
|  |  |  |  |  |  | 1572 | 20.7378 | 23 | HN077 | HN093 |
|  |  |  |  |  |  | 1573 | 23.5481 | 25 | HN077 | HN116 |
|  |  |  |  |  |  | 1574 | 22.5852 | 28 | HN077 | HN130 |
|  |  |  |  |  |  | 1575 | 19.366 | 24 | HN079 | HN081 |
|  |  |  |  |  |  | 1576 | 22.7985 | 27 | HN079 | HN082 |
|  |  |  |  |  |  | 1577 | 25.1783 | 26 | HN079 | HN082 |
|  |  |  |  |  |  | 1578 | 20.5055 | 25 | HN079 | HN107 |
|  |  |  |  |  |  | 1579 | 23.3166 | 23 | HN079 | HN113 |
|  |  |  |  |  |  | 1580 | 22.6783 | 26 | HN079 | HN125 |
|  |  |  |  |  |  | 1581 | 21.7966 | 25 | HN079 | HN130 |
|  |  |  |  |  |  | 1582 | 22.4383 | 28 | HN079 | HN132 |
|  |  |  |  |  |  | 1583 | 19.2049 | 24 | HN079 | HN138 |
|  |  |  |  |  |  | 1584 | 25.9913 | 25 | HN080 | HN082 |
|  |  |  |  |  |  | 1585 | 26.8706 | 26 | HN080 | HN082 |
|  |  |  |  |  |  | 1586 | 18.8787 | 21 | HN080 | HN086 |
|  |  |  |  |  |  | 1587 | 22.9644 | 25 | HN080 | HN090 |
|  |  |  |  |  |  | 1588 | 25.8667 | 28 | HN080 | HN094 |
|  |  |  |  |  |  | 1589 | 25.3494 | 30 | HN080 | HN096 |
|  |  |  |  |  |  | 1590 | 20.2588 | 25 | HN080 | HN103 |
|  |  |  |  |  |  | 1591 | 23.4418 | 24 | HN080 | HN104 |
|  |  |  |  |  |  | 1592 | 25.5445 | 28 | HN080 | HN118 |
|  |  |  |  |  |  | 1593 | 21.7424 | 26 | HN080 | HN119 |
|  |  |  |  |  |  | 1594 | 22.0789 | 27 | HN080 | HN132 |
|  |  |  |  |  |  | 1595 | 22.0789 | 27 | HN080 | HN132 |
|  |  |  |  |  |  | 1596 | 19.6004 | 21 | HN080 | HN134 |
|  |  |  |  |  |  | 1597 | 24.3926 | 26 | HN080 | HN138 |
|  |  |  |  |  |  | 1598 | 19.0815 | 20 | HN080 | HN140 |
|  |  |  |  |  |  | 1599 | 19.5649 | 21 | HN080 | HN140 |
|  |  |  |  |  |  | 1600 | 19.2383 | 21 | HN080 | HN146 |
|  |  |  |  |  |  | 1601 | 22.3806 | 24 | HN080 | HN148 |
|  |  |  |  |  |  | 1602 | 22.8103 | 24 | HN081 | HN082 |
|  |  |  |  |  |  | 1603 | 18.1243 | 17 | HN081 | HN093 |
|  |  |  |  |  |  | 1604 | 24.1403 | 28 | HN081 | HN118 |
|  |  |  |  |  |  | 1605 | 21.6312 | 23 | HN081 | HN123 |
|  |  |  |  |  |  | 1606 | 19.0412 | 23 | HN081 | HN124 |
|  |  |  |  |  |  | 1607 | 20.86 | 22 | HN081 | HN125 |
|  |  |  |  |  |  | 1608 | 21.5227 | 26 | HN081 | HN129 |
|  |  |  |  |  |  | 1609 | 21.2837 | 27 | HN081 | HN131 |
|  |  |  |  |  |  | 1610 | 17.579 | 20 | HN081 | HN144 |
|  |  |  |  |  |  | 1611 | 23.8161 | 26 | HN082 | HN083 |
|  |  |  |  |  |  | 1612 | 24.1561 | 24 | HN082 | HN086 |
|  |  |  |  |  |  | 1613 | 26.0079 | 27 | HN082 | HN090 |
|  |  |  |  |  |  | 1614 | 25.1048 | 25 | HN082 | HN094 |
|  |  |  |  |  |  | 1615 | 24.964 | 26 | HN082 | HN094 |
|  |  |  |  |  |  | 1616 | 25.574 | 28 | HN082 | HN094 |
|  |  |  |  |  |  | 1617 | 25.3057 | 28 | HN082 | HN095 |
|  |  |  |  |  |  | 1618 | 25.1434 | 28 | HN082 | HN109 |
|  |  |  |  |  |  | 1619 | 27.9249 | 31 | HN082 | HN109 |
|  |  |  |  |  |  | 1620 | 25.07 | 23 | HN082 | HN111 |
|  |  |  |  |  |  | 1621 | 23.6722 | 22 | HN082 | HN113 |
|  |  |  |  |  |  | 1622 | 28.0755 | 30 | HN082 | HN114 |
|  |  |  |  |  |  | 1623 | 28.4289 | 30 | HN082 | HN116 |
|  |  |  |  |  |  | 1624 | 24.6933 | 26 | HN082 | HN118 |
|  |  |  |  |  |  | 1625 | 27.6675 | 31 | HN082 | HN125 |
|  |  |  |  |  |  | 1626 | 27.6675 | 31 | HN082 | HN125 |
|  |  |  |  |  |  | 1627 | 24.5927 | 27 | HN082 | HN128 |
|  |  |  |  |  |  | 1628 | 26.4976 | 27 | HN082 | HN129 |
|  |  |  |  |  |  | 1629 | 22.3907 | 26 | HN082 | HN137 |
|  |  |  |  |  |  | 1630 | 25.8115 | 26 | HN082 | HN137 |
|  |  |  |  |  |  | 1631 | 21.9013 | 25 | HN082 | HN142 |
|  |  |  |  |  |  | 1632 | 26.3973 | 30 | HN082 | HN144 |
|  |  |  |  |  |  | 1633 | 24.4622 | 22 | HN082 | HN148 |
|  |  |  |  |  |  | 1634 | 20.4996 | 26 | HN083 | HN087 |
|  |  |  |  |  |  | 1635 | 23.7585 | 29 | HN083 | HN087 |
|  |  |  |  |  |  | 1636 | 21.1918 | 27 | HN083 | HN093 |
|  |  |  |  |  |  | 1637 | 25.7869 | 27 | HN083 | HN097 |
|  |  |  |  |  |  | 1638 | 21.9834 | 26 | HN083 | HN101 |
|  |  |  |  |  |  | 1639 | 24.0347 | 27 | HN083 | HN101 |
|  |  |  |  |  |  | 1640 | 25.6589 | 29 | HN083 | HN111 |
|  |  |  |  |  |  | 1641 | 26.2953 | 29 | HN083 | HN113 |
|  |  |  |  |  |  | 1642 | 27.7619 | 29 | HN083 | HN113 |
|  |  |  |  |  |  | 1643 | 23.7765 | 33 | HN083 | HN114 |
|  |  |  |  |  |  | 1644 | 21.3639 | 30 | HN083 | HN114 |
|  |  |  |  |  |  | 1645 | 24.3731 | 28 | HN083 | HN114 |
|  |  |  |  |  |  | 1646 | 29.4069 | 33 | HN083 | HN116 |
|  |  |  |  |  |  | 1647 | 22.2308 | 24 | HN083 | HN117 |
|  |  |  |  |  |  | 1648 | 24.3874 | 31 | HN083 | HN118 |
|  |  |  |  |  |  | 1649 | 25.7021 | 31 | HN083 | HN120 |
|  |  |  |  |  |  | 1650 | 24.7772 | 30 | HN083 | HN122 |
|  |  |  |  |  |  | 1651 | 25.5436 | 31 | HN083 | HN125 |
|  |  |  |  |  |  | 1652 | 20.8887 | 26 | HN083 | HN130 |
|  |  |  |  |  |  | 1653 | 25.8458 | 31 | HN083 | HN130 |
|  |  |  |  |  |  | 1654 | 23.9303 | 31 | HN083 | HN133 |
|  |  |  |  |  |  | 1655 | 23.8341 | 30 | HN083 | HN136 |
|  |  |  |  |  |  | 1656 | 23.6097 | 29 | HN083 | HN138 |
|  |  |  |  |  |  | 1657 | 24.5147 | 28 | HN083 | HN140 |
|  |  |  |  |  |  | 1658 | 22.1153 | 28 | HN083 | HN142 |
|  |  |  |  |  |  | 1659 | 23.4619 | 29 | HN083 | HN142 |
|  |  |  |  |  |  | 1660 | 19.9424 | 20 | HN084 | HN093 |
|  |  |  |  |  |  | 1661 | 18.6477 | 22 | HN084 | HN103 |
|  |  |  |  |  |  | 1662 | 18.4155 | 20 | HN084 | HN108 |
|  |  |  |  |  |  | 1663 | 21.8767 | 23 | HN084 | HN113 |
|  |  |  |  |  |  | 1664 | 19.9493 | 27 | HN084 | HN125 |
|  |  |  |  |  |  | 1665 | 19.3064 | 22 | HN084 | HN126 |
|  |  |  |  |  |  | 1666 | 23.5912 | 26 | HN085 | HN090 |
|  |  |  |  |  |  | 1667 | 24.4175 | 28 | HN085 | HN090 |
|  |  |  |  |  |  | 1668 | 20.6258 | 22 | HN085 | HN092 |
|  |  |  |  |  |  | 1669 | 24.3602 | 27 | HN085 | HN094 |
|  |  |  |  |  |  | 1670 | 27.1088 | 30 | HN085 | HN096 |
|  |  |  |  |  |  | 1671 | 19.369 | 22 | HN085 | HN111 |
|  |  |  |  |  |  | 1672 | 24.0338 | 25 | HN085 | HN113 |
|  |  |  |  |  |  | 1673 | 16.1151 | 20 | HN085 | HN114 |
|  |  |  |  |  |  | 1674 | 23.6145 | 24 | HN085 | HN118 |
|  |  |  |  |  |  | 1675 | 23.3304 | 26 | HN085 | HN130 |
|  |  |  |  |  |  | 1676 | 24.3977 | 28 | HN085 | HN130 |
|  |  |  |  |  |  | 1677 | 24.6291 | 28 | HN085 | HN131 |
|  |  |  |  |  |  | 1678 | 19.974 | 21 | HN085 | HN144 |
|  |  |  |  |  |  | 1679 | 19.3435 | 20 | HN085 | HN148 |
|  |  |  |  |  |  | 1680 | 15.1151 | 20 | HN086 | HN102 |
|  |  |  |  |  |  | 1681 | 17.8543 | 23 | HN086 | HN115 |
|  |  |  |  |  |  | 1682 | 13.9037 | 19 | HN086 | HN117 |
|  |  |  |  |  |  | 1683 | 19.1783 | 20 | HN086 | HN117 |
|  |  |  |  |  |  | 1684 | 14.6519 | 20 | HN086 | HN123 |
|  |  |  |  |  |  | 1685 | 18.7324 | 26 | HN086 | HN127 |
|  |  |  |  |  |  | 1686 | 18.4793 | 21 | HN086 | HN133 |
|  |  |  |  |  |  | 1687 | 15.4759 | 18 | HN087 | HN088 |
|  |  |  |  |  |  | 1688 | 16.8278 | 20 | HN087 | HN101 |
|  |  |  |  |  |  | 1689 | 23.244 | 27 | HN087 | HN112 |
|  |  |  |  |  |  | 1690 | 24.3734 | 24 | HN087 | HN113 |
|  |  |  |  |  |  | 1691 | 18.403 | 21 | HN087 | HN122 |
|  |  |  |  |  |  | 1692 | 24.8548 | 27 | HN087 | HN127 |
|  |  |  |  |  |  | 1693 | 22.424 | 25 | HN087 | HN138 |
|  |  |  |  |  |  | 1694 | 21.0657 | 20 | HN087 | HN142 |
|  |  |  |  |  |  | 1695 | 19.4878 | 20 | HN088 | HN101 |
|  |  |  |  |  |  | 1696 | 23.4754 | 24 | HN088 | HN116 |
|  |  |  |  |  |  | 1697 | 22.1751 | 23 | HN088 | HN125 |
|  |  |  |  |  |  | 1698 | 21.238 | 23 | HN088 | HN129 |
|  |  |  |  |  |  | 1699 | 23.6646 | 27 | HN090 | HN101 |
|  |  |  |  |  |  | 1700 | 18.6813 | 26 | HN090 | HN105 |
|  |  |  |  |  |  | 1701 | 24.8276 | 30 | HN090 | HN107 |
|  |  |  |  |  |  | 1702 | 24.1699 | 27 | HN090 | HN113 |
|  |  |  |  |  |  | 1703 | 18.7965 | 24 | HN090 | HN115 |
|  |  |  |  |  |  | 1704 | 25.0042 | 27 | HN090 | HN118 |
|  |  |  |  |  |  | 1705 | 25.6038 | 30 | HN090 | HN131 |
|  |  |  |  |  |  | 1706 | 22.0107 | 26 | HN090 | HN146 |
|  |  |  |  |  |  | 1707 | 21.8731 | 27 | HN090 | HN147 |
|  |  |  |  |  |  | 1708 | 22.6296 | 25 | HN090 | HN150 |
|  |  |  |  |  |  | 1709 | 19.8833 | 26 | HN090 | HN150 |
|  |  |  |  |  |  | 1710 | 20.926 | 25 | HN091 | HN093 |
|  |  |  |  |  |  | 1711 | 25.2869 | 29 | HN091 | HN096 |
|  |  |  |  |  |  | 1712 | 23.0859 | 21 | HN091 | HN097 |
|  |  |  |  |  |  | 1713 | 19.094 | 23 | HN091 | HN103 |
|  |  |  |  |  |  | 1714 | 20.1723 | 26 | HN091 | HN109 |
|  |  |  |  |  |  | 1715 | 20.2898 | 22 | HN091 | HN110 |
|  |  |  |  |  |  | 1716 | 20.3291 | 21 | HN091 | HN117 |
|  |  |  |  |  |  | 1717 | 18.3648 | 22 | HN091 | HN136 |
|  |  |  |  |  |  | 1718 | 17.6441 | 14 | HN092 | HN106 |
|  |  |  |  |  |  | 1719 | 18.5945 | 18 | HN092 | HN117 |
|  |  |  |  |  |  | 1720 | 19.2717 | 21 | HN092 | HN125 |
|  |  |  |  |  |  | 1721 | 21.7161 | 23 | HN092 | HN125 |
|  |  |  |  |  |  | 1722 | 19.907 | 23 | HN092 | HN131 |
|  |  |  |  |  |  | 1723 | 21.6152 | 23 | HN093 | HN095 |
|  |  |  |  |  |  | 1724 | 23.6017 | 22 | HN093 | HN097 |
|  |  |  |  |  |  | 1725 | 21.9137 | 27 | HN093 | HN098 |
|  |  |  |  |  |  | 1726 | 19.2556 | 22 | HN093 | HN101 |
|  |  |  |  |  |  | 1727 | 21.3385 | 23 | HN093 | HN101 |
|  |  |  |  |  |  | 1728 | 24.1035 | 26 | HN093 | HN105 |
|  |  |  |  |  |  | 1729 | 22.7622 | 27 | HN093 | HN105 |
|  |  |  |  |  |  | 1730 | 22.3722 | 24 | HN093 | HN113 |
|  |  |  |  |  |  | 1731 | 19.1884 | 26 | HN093 | HN128 |
|  |  |  |  |  |  | 1732 | 22.1919 | 29 | HN093 | HN130 |
|  |  |  |  |  |  | 1733 | 26.5241 | 27 | HN093 | HN131 |
|  |  |  |  |  |  | 1734 | 22.1645 | 25 | HN093 | HN134 |
|  |  |  |  |  |  | 1735 | 19.4761 | 21 | HN093 | HN140 |
|  |  |  |  |  |  | 1736 | 18.708 | 22 | HN093 | HN147 |
|  |  |  |  |  |  | 1737 | 23.1606 | 24 | HN093 | HN147 |
|  |  |  |  |  |  | 1738 | 17.6218 | 22 | HN094 | HN095 |
|  |  |  |  |  |  | 1739 | 23.9247 | 29 | HN094 | HN096 |
|  |  |  |  |  |  | 1740 | 28.7789 | 27 | HN094 | HN097 |
|  |  |  |  |  |  | 1741 | 28.4952 | 26 | HN094 | HN097 |
|  |  |  |  |  |  | 1742 | 24.0763 | 29 | HN094 | HN098 |
|  |  |  |  |  |  | 1743 | 26.8431 | 33 | HN094 | HN101 |
|  |  |  |  |  |  | 1744 | 23.9602 | 29 | HN094 | HN102 |
|  |  |  |  |  |  | 1745 | 27.1421 | 27 | HN094 | HN104 |
|  |  |  |  |  |  | 1746 | 21.293 | 26 | HN094 | HN107 |
|  |  |  |  |  |  | 1747 | 23.1319 | 28 | HN094 | HN111 |
|  |  |  |  |  |  | 1748 | 25.2718 | 28 | HN094 | HN112 |
|  |  |  |  |  |  | 1749 | 25.2038 | 30 | HN094 | HN113 |
|  |  |  |  |  |  | 1750 | 28.5876 | 29 | HN094 | HN116 |
|  |  |  |  |  |  | 1751 | 26.2365 | 30 | HN094 | HN116 |
|  |  |  |  |  |  | 1752 | 23.0117 | 28 | HN094 | HN122 |
|  |  |  |  |  |  | 1753 | 21.9193 | 26 | HN094 | HN125 |
|  |  |  |  |  |  | 1754 | 25.8465 | 29 | HN094 | HN127 |
|  |  |  |  |  |  | 1755 | 22.9995 | 28 | HN094 | HN128 |
|  |  |  |  |  |  | 1756 | 26.831 | 31 | HN094 | HN129 |
|  |  |  |  |  |  | 1757 | 24.5411 | 28 | HN094 | HN130 |
|  |  |  |  |  |  | 1758 | 24.0369 | 27 | HN094 | HN130 |
|  |  |  |  |  |  | 1759 | 20.921 | 26 | HN094 | HN143 |
|  |  |  |  |  |  | 1760 | 23.6448 | 26 | HN094 | HN145 |
|  |  |  |  |  |  | 1761 | 23.9918 | 30 | HN094 | HN147 |
|  |  |  |  |  |  | 1762 | 22.2036 | 25 | HN094 | HN149 |
|  |  |  |  |  |  | 1763 | 19.0276 | 22 | HN095 | HN112 |
|  |  |  |  |  |  | 1764 | 21.0899 | 23 | HN095 | HN118 |
|  |  |  |  |  |  | 1765 | 21.4274 | 30 | HN096 | HN105 |
|  |  |  |  |  |  | 1766 | 20.5363 | 27 | HN096 | HN107 |
|  |  |  |  |  |  | 1767 | 25.8436 | 29 | HN096 | HN111 |
|  |  |  |  |  |  | 1768 | 27.2472 | 31 | HN096 | HN114 |
|  |  |  |  |  |  | 1769 | 24.6325 | 28 | HN096 | HN116 |
|  |  |  |  |  |  | 1770 | 27.5112 | 29 | HN096 | HN116 |
|  |  |  |  |  |  | 1771 | 26.1039 | 28 | HN096 | HN116 |
|  |  |  |  |  |  | 1772 | 27.1292 | 30 | HN096 | HN117 |
|  |  |  |  |  |  | 1773 | 26.7481 | 30 | HN096 | HN125 |
|  |  |  |  |  |  | 1774 | 22.8493 | 24 | HN096 | HN132 |
|  |  |  |  |  |  | 1775 | 23.2932 | 28 | HN096 | HN133 |
|  |  |  |  |  |  | 1776 | 26.1892 | 31 | HN096 | HN134 |
|  |  |  |  |  |  | 1777 | 22.2493 | 24 | HN096 | HN135 |
|  |  |  |  |  |  | 1778 | 22.8126 | 27 | HN096 | HN136 |
|  |  |  |  |  |  | 1779 | 24.2603 | 29 | HN096 | HN137 |
|  |  |  |  |  |  | 1780 | 23.2475 | 29 | HN096 | HN138 |
|  |  |  |  |  |  | 1781 | 27.8379 | 28 | HN096 | HN140 |
|  |  |  |  |  |  | 1782 | 25.2837 | 28 | HN096 | HN148 |
|  |  |  |  |  |  | 1783 | 24.8028 | 28 | HN096 | HN148 |
|  |  |  |  |  |  | 1784 | 21.6448 | 21 | HN097 | HN102 |
|  |  |  |  |  |  | 1785 | 21.7899 | 19 | HN097 | HN102 |
|  |  |  |  |  |  | 1786 | 22.5993 | 27 | HN097 | HN105 |
|  |  |  |  |  |  | 1787 | 24.5232 | 23 | HN097 | HN107 |
|  |  |  |  |  |  | 1788 | 24.3842 | 19 | HN097 | HN113 |
|  |  |  |  |  |  | 1789 | 22.2279 | 21 | HN097 | HN113 |
|  |  |  |  |  |  | 1790 | 23.2615 | 21 | HN097 | HN116 |
|  |  |  |  |  |  | 1791 | 23.6992 | 19 | HN097 | HN118 |
|  |  |  |  |  |  | 1792 | 25.3875 | 18 | HN097 | HN118 |
|  |  |  |  |  |  | 1793 | 17.7764 | 18 | HN097 | HN126 |
|  |  |  |  |  |  | 1794 | 25.2571 | 28 | HN097 | HN127 |
|  |  |  |  |  |  | 1795 | 20.9294 | 21 | HN097 | HN127 |
|  |  |  |  |  |  | 1796 | 22.847 | 23 | HN097 | HN129 |
|  |  |  |  |  |  | 1797 | 26.0509 | 25 | HN097 | HN131 |
|  |  |  |  |  |  | 1798 | 20.3095 | 20 | HN097 | HN132 |
|  |  |  |  |  |  | 1799 | 21.5469 | 18 | HN097 | HN133 |
|  |  |  |  |  |  | 1800 | 22.4837 | 21 | HN097 | HN143 |
|  |  |  |  |  |  | 1801 | 18.4727 | 21 | HN097 | HN144 |
|  |  |  |  |  |  | 1802 | 21.9456 | 25 | HN098 | HN101 |
|  |  |  |  |  |  | 1803 | 22.3032 | 26 | HN098 | HN111 |
|  |  |  |  |  |  | 1804 | 20.4227 | 26 | HN098 | HN117 |
|  |  |  |  |  |  | 1805 | 20.3781 | 26 | HN098 | HN118 |
|  |  |  |  |  |  | 1806 | 20.4591 | 25 | HN098 | HN138 |
|  |  |  |  |  |  | 1807 | 22.6523 | 24 | HN100 | HN113 |
|  |  |  |  |  |  | 1808 | 23.2367 | 28 | HN101 | HN105 |
|  |  |  |  |  |  | 1809 | 21.7971 | 26 | HN101 | HN109 |
|  |  |  |  |  |  | 1810 | 22.846 | 24 | HN101 | HN111 |
|  |  |  |  |  |  | 1811 | 21.8443 | 28 | HN101 | HN115 |
|  |  |  |  |  |  | 1812 | 18.5285 | 22 | HN101 | HN120 |
|  |  |  |  |  |  | 1813 | 20.1473 | 24 | HN101 | HN126 |
|  |  |  |  |  |  | 1814 | 21.5076 | 26 | HN101 | HN127 |
|  |  |  |  |  |  | 1815 | 21.809 | 25 | HN101 | HN129 |
|  |  |  |  |  |  | 1816 | 22.7609 | 29 | HN101 | HN130 |
|  |  |  |  |  |  | 1817 | 25.6903 | 31 | HN101 | HN131 |
|  |  |  |  |  |  | 1818 | 19.4066 | 21 | HN101 | HN133 |
|  |  |  |  |  |  | 1819 | 19.9318 | 23 | HN101 | HN140 |
|  |  |  |  |  |  | 1820 | 16.225 | 21 | HN101 | HN142 |
|  |  |  |  |  |  | 1821 | 22.0435 | 29 | HN102 | HN109 |
|  |  |  |  |  |  | 1822 | 21.6525 | 25 | HN102 | HN111 |
|  |  |  |  |  |  | 1823 | 25.4348 | 26 | HN102 | HN116 |
|  |  |  |  |  |  | 1824 | 22.0123 | 25 | HN102 | HN123 |
|  |  |  |  |  |  | 1825 | 19.9456 | 25 | HN102 | HN138 |
|  |  |  |  |  |  | 1826 | 23.6853 | 28 | HN103 | HN105 |
|  |  |  |  |  |  | 1827 | 26.3439 | 30 | HN103 | HN105 |
|  |  |  |  |  |  | 1828 | 24.9509 | 30 | HN103 | HN107 |
|  |  |  |  |  |  | 1829 | 20.8778 | 25 | HN103 | HN112 |
|  |  |  |  |  |  | 1830 | 24.821 | 28 | HN103 | HN113 |
|  |  |  |  |  |  | 1831 | 25.1404 | 26 | HN103 | HN116 |
|  |  |  |  |  |  | 1832 | 23.4883 | 29 | HN103 | HN129 |
|  |  |  |  |  |  | 1833 | 27.1843 | 32 | HN103 | HN131 |
|  |  |  |  |  |  | 1834 | 27.1109 | 31 | HN103 | HN131 |
|  |  |  |  |  |  | 1835 | 20.5401 | 27 | HN103 | HN133 |
|  |  |  |  |  |  | 1836 | 22.5357 | 28 | HN103 | HN136 |
|  |  |  |  |  |  | 1837 | 23.2734 | 30 | HN103 | HN138 |
|  |  |  |  |  |  | 1838 | 23.1224 | 26 | HN103 | HN144 |
|  |  |  |  |  |  | 1839 | 19.3543 | 26 | HN103 | HN145 |
|  |  |  |  |  |  | 1840 | 22.1329 | 28 | HN103 | HN147 |
|  |  |  |  |  |  | 1841 | 26.8631 | 31 | HN104 | HN105 |
|  |  |  |  |  |  | 1842 | 28.399 | 32 | HN104 | HN105 |
|  |  |  |  |  |  | 1843 | 23.8661 | 23 | HN104 | HN107 |
|  |  |  |  |  |  | 1844 | 23.9918 | 24 | HN104 | HN107 |
|  |  |  |  |  |  | 1845 | 20.0234 | 18 | HN104 | HN134 |
|  |  |  |  |  |  | 1846 | 20.4048 | 20 | HN104 | HN149 |
|  |  |  |  |  |  | 1847 | 22.5729 | 31 | HN105 | HN107 |
|  |  |  |  |  |  | 1848 | 20.5204 | 28 | HN105 | HN112 |
|  |  |  |  |  |  | 1849 | 25.7318 | 28 | HN105 | HN116 |
|  |  |  |  |  |  | 1850 | 24.584 | 29 | HN105 | HN117 |
|  |  |  |  |  |  | 1851 | 23.4851 | 26 | HN105 | HN117 |
|  |  |  |  |  |  | 1852 | 25.6821 | 29 | HN105 | HN122 |
|  |  |  |  |  |  | 1853 | 20.9946 | 26 | HN105 | HN123 |
|  |  |  |  |  |  | 1854 | 20.2335 | 24 | HN105 | HN127 |
|  |  |  |  |  |  | 1855 | 24.5501 | 29 | HN105 | HN128 |
|  |  |  |  |  |  | 1856 | 23.8411 | 26 | HN105 | HN131 |
|  |  |  |  |  |  | 1857 | 21.4418 | 31 | HN105 | HN133 |
|  |  |  |  |  |  | 1858 | 22.4346 | 25 | HN105 | HN145 |
|  |  |  |  |  |  | 1859 | 21.9084 | 25 | HN105 | HN145 |
|  |  |  |  |  |  | 1860 | 23.038 | 25 | HN105 | HN149 |
|  |  |  |  |  |  | 1861 | 20.0869 | 21 | HN106 | HN109 |
|  |  |  |  |  |  | 1862 | 19.1235 | 19 | HN106 | HN116 |
|  |  |  |  |  |  | 1863 | 21.5415 | 21 | HN106 | HN125 |
|  |  |  |  |  |  | 1864 | 23.9945 | 27 | HN106 | HN129 |
|  |  |  |  |  |  | 1865 | 28.4137 | 30 | HN106 | HN129 |
|  |  |  |  |  |  | 1866 | 19.4196 | 18 | HN106 | HN133 |
|  |  |  |  |  |  | 1867 | 23.506 | 20 | HN106 | HN138 |
|  |  |  |  |  |  | 1868 | 22.5829 | 20 | HN106 | HN147 |
|  |  |  |  |  |  | 1869 | 20.1673 | 27 | HN107 | HN112 |
|  |  |  |  |  |  | 1870 | 23.4251 | 25 | HN107 | HN116 |
|  |  |  |  |  |  | 1871 | 21.8774 | 24 | HN107 | HN124 |
|  |  |  |  |  |  | 1872 | 21.5999 | 25 | HN107 | HN130 |
|  |  |  |  |  |  | 1873 | 23.3414 | 29 | HN107 | HN131 |
|  |  |  |  |  |  | 1874 | 22.064 | 24 | HN107 | HN141 |
|  |  |  |  |  |  | 1875 | 25.0835 | 26 | HN108 | HN111 |
|  |  |  |  |  |  | 1876 | 20.7629 | 26 | HN108 | HN126 |
|  |  |  |  |  |  | 1877 | 20.7629 | 26 | HN108 | HN126 |
|  |  |  |  |  |  | 1878 | 21.9013 | 27 | HN108 | HN130 |
|  |  |  |  |  |  | 1879 | 18.8264 | 22 | HN108 | HN144 |
|  |  |  |  |  |  | 1880 | 22.4178 | 26 | HN109 | HN113 |
|  |  |  |  |  |  | 1881 | 22.779 | 27 | HN109 | HN115 |
|  |  |  |  |  |  | 1882 | 17.4765 | 23 | HN109 | HN119 |
|  |  |  |  |  |  | 1883 | 21.5608 | 25 | HN109 | HN122 |
|  |  |  |  |  |  | 1884 | 21.1121 | 26 | HN109 | HN130 |
|  |  |  |  |  |  | 1885 | 21.4469 | 28 | HN109 | HN131 |
|  |  |  |  |  |  | 1886 | 21.7839 | 26 | HN109 | HN131 |
|  |  |  |  |  |  | 1887 | 21.0799 | 25 | HN109 | HN133 |
|  |  |  |  |  |  | 1888 | 19.5426 | 24 | HN109 | HN139 |
|  |  |  |  |  |  | 1889 | 24.9976 | 25 | HN110 | HN116 |
|  |  |  |  |  |  | 1890 | 21.6353 | 22 | HN110 | HN118 |
|  |  |  |  |  |  | 1891 | 24.171 | 30 | HN110 | HN129 |
|  |  |  |  |  |  | 1892 | 17.8384 | 22 | HN110 | HN150 |
|  |  |  |  |  |  | 1893 | 22.7277 | 21 | HN111 | HN115 |
|  |  |  |  |  |  | 1894 | 25.6952 | 26 | HN111 | HN118 |
|  |  |  |  |  |  | 1895 | 21.6757 | 23 | HN111 | HN122 |
|  |  |  |  |  |  | 1896 | 22.778 | 27 | HN111 | HN123 |
|  |  |  |  |  |  | 1897 | 26.1206 | 28 | HN111 | HN127 |
|  |  |  |  |  |  | 1898 | 22.4534 | 23 | HN111 | HN128 |
|  |  |  |  |  |  | 1899 | 25.0931 | 26 | HN111 | HN129 |
|  |  |  |  |  |  | 1900 | 27.1076 | 28 | HN111 | HN130 |
|  |  |  |  |  |  | 1901 | 22.118 | 28 | HN111 | HN131 |
|  |  |  |  |  |  | 1902 | 22.7102 | 24 | HN111 | HN132 |
|  |  |  |  |  |  | 1903 | 25.2525 | 27 | HN111 | HN132 |
|  |  |  |  |  |  | 1904 | 24.7535 | 22 | HN111 | HN135 |
|  |  |  |  |  |  | 1905 | 22.7899 | 22 | HN111 | HN140 |
|  |  |  |  |  |  | 1906 | 24.0097 | 27 | HN111 | HN143 |
|  |  |  |  |  |  | 1907 | 20.7963 | 24 | HN111 | HN145 |
|  |  |  |  |  |  | 1908 | 21.0864 | 24 | HN111 | HN147 |
|  |  |  |  |  |  | 1909 | 21.4509 | 23 | HN111 | HN149 |
|  |  |  |  |  |  | 1910 | 24.3962 | 25 | HN112 | HN118 |
|  |  |  |  |  |  | 1911 | 20.1707 | 25 | HN112 | HN122 |
|  |  |  |  |  |  | 1912 | 20.6152 | 25 | HN112 | HN123 |
|  |  |  |  |  |  | 1913 | 25.3383 | 32 | HN112 | HN129 |
|  |  |  |  |  |  | 1914 | 18.2339 | 25 | HN112 | HN132 |
|  |  |  |  |  |  | 1915 | 23.4913 | 27 | HN112 | HN138 |
|  |  |  |  |  |  | 1916 | 23.0794 | 29 | HN112 | HN140 |
|  |  |  |  |  |  | 1917 | 20.8296 | 25 | HN113 | HN115 |
|  |  |  |  |  |  | 1918 | 20.3595 | 27 | HN113 | HN127 |
|  |  |  |  |  |  | 1919 | 23.0459 | 26 | HN113 | HN127 |
|  |  |  |  |  |  | 1920 | 28.8903 | 29 | HN113 | HN129 |
|  |  |  |  |  |  | 1921 | 23.3132 | 26 | HN113 | HN131 |
|  |  |  |  |  |  | 1922 | 25.5609 | 26 | HN113 | HN138 |
|  |  |  |  |  |  | 1923 | 21.5484 | 26 | HN113 | HN138 |
|  |  |  |  |  |  | 1924 | 22.6533 | 23 | HN113 | HN141 |
|  |  |  |  |  |  | 1925 | 24.011 | 25 | HN113 | HN150 |
|  |  |  |  |  |  | 1926 | 24.4573 | 25 | HN114 | HN118 |
|  |  |  |  |  |  | 1927 | 23.7712 | 24 | HN114 | HN118 |
|  |  |  |  |  |  | 1928 | 25.7463 | 31 | HN114 | HN129 |
|  |  |  |  |  |  | 1929 | 22.3593 | 29 | HN114 | HN131 |
|  |  |  |  |  |  | 1930 | 21.4489 | 29 | HN114 | HN131 |
|  |  |  |  |  |  | 1931 | 25.4738 | 30 | HN114 | HN131 |
|  |  |  |  |  |  | 1932 | 20.1758 | 20 | HN114 | HN137 |
|  |  |  |  |  |  | 1933 | 19.7441 | 20 | HN114 | HN140 |
|  |  |  |  |  |  | 1934 | 20.9685 | 23 | HN114 | HN141 |
|  |  |  |  |  |  | 1935 | 22.6385 | 25 | HN114 | HN148 |
|  |  |  |  |  |  | 1936 | 22.2647 | 24 | HN114 | HN148 |
|  |  |  |  |  |  | 1937 | 24.1355 | 25 | HN115 | HN116 |
|  |  |  |  |  |  | 1938 | 19.4514 | 23 | HN115 | HN122 |
|  |  |  |  |  |  | 1939 | 21.2914 | 24 | HN115 | HN129 |
|  |  |  |  |  |  | 1940 | 22.6448 | 28 | HN115 | HN130 |
|  |  |  |  |  |  | 1941 | 20.2014 | 19 | HN115 | HN148 |
|  |  |  |  |  |  | 1942 | 24.4768 | 27 | HN115 | HN149 |
|  |  |  |  |  |  | 1943 | 25.5264 | 28 | HN116 | HN120 |
|  |  |  |  |  |  | 1944 | 20.2673 | 23 | HN116 | HN124 |
|  |  |  |  |  |  | 1945 | 24.4372 | 26 | HN116 | HN125 |
|  |  |  |  |  |  | 1946 | 25.6526 | 28 | HN116 | HN126 |
|  |  |  |  |  |  | 1947 | 23.5432 | 26 | HN116 | HN126 |
|  |  |  |  |  |  | 1948 | 30.7485 | 31 | HN116 | HN129 |
|  |  |  |  |  |  | 1949 | 31.3646 | 34 | HN116 | HN129 |
|  |  |  |  |  |  | 1950 | 28.6269 | 31 | HN116 | HN129 |
|  |  |  |  |  |  | 1951 | 26.9156 | 30 | HN116 | HN129 |
|  |  |  |  |  |  | 1952 | 30.6029 | 32 | HN116 | HN131 |
|  |  |  |  |  |  | 1953 | 24.3913 | 23 | HN116 | HN135 |
|  |  |  |  |  |  | 1954 | 25.5989 | 27 | HN116 | HN140 |
|  |  |  |  |  |  | 1955 | 23.2393 | 24 | HN116 | HN141 |
|  |  |  |  |  |  | 1956 | 24.6288 | 25 | HN116 | HN144 |
|  |  |  |  |  |  | 1957 | 22.2775 | 24 | HN116 | HN146 |
|  |  |  |  |  |  | 1958 | 25.8496 | 28 | HN116 | HN149 |
|  |  |  |  |  |  | 1959 | 26.0838 | 26 | HN117 | HN118 |
|  |  |  |  |  |  | 1960 | 22.9151 | 26 | HN117 | HN120 |
|  |  |  |  |  |  | 1961 | 20.1201 | 21 | HN117 | HN135 |
|  |  |  |  |  |  | 1962 | 20.0317 | 22 | HN117 | HN139 |
|  |  |  |  |  |  | 1963 | 22.2066 | 20 | HN117 | HN140 |
|  |  |  |  |  |  | 1964 | 19.1609 | 21 | HN117 | HN142 |
|  |  |  |  |  |  | 1965 | 16.9299 | 16 | HN117 | HN142 |
|  |  |  |  |  |  | 1966 | 18.697 | 22 | HN117 | HN149 |
|  |  |  |  |  |  | 1967 | 19.1687 | 22 | HN118 | HN121 |
|  |  |  |  |  |  | 1968 | 22.4521 | 23 | HN118 | HN123 |
|  |  |  |  |  |  | 1969 | 22.5008 | 26 | HN118 | HN125 |
|  |  |  |  |  |  | 1970 | 25.3276 | 29 | HN118 | HN127 |
|  |  |  |  |  |  | 1971 | 24.9918 | 28 | HN118 | HN129 |
|  |  |  |  |  |  | 1972 | 22.9497 | 28 | HN118 | HN130 |
|  |  |  |  |  |  | 1973 | 23.8669 | 25 | HN118 | HN131 |
|  |  |  |  |  |  | 1974 | 19.1539 | 24 | HN118 | HN136 |
|  |  |  |  |  |  | 1975 | 23.5692 | 27 | HN118 | HN136 |
|  |  |  |  |  |  | 1976 | 24.1803 | 28 | HN118 | HN137 |
|  |  |  |  |  |  | 1977 | 23.2642 | 26 | HN118 | HN139 |
|  |  |  |  |  |  | 1978 | 23.7236 | 25 | HN118 | HN143 |
|  |  |  |  |  |  | 1979 | 22.1705 | 24 | HN118 | HN144 |
|  |  |  |  |  |  | 1980 | 14.9054 | 22 | HN119 | HN128 |
|  |  |  |  |  |  | 1981 | 16.2651 | 22 | HN119 | HN145 |
|  |  |  |  |  |  | 1982 | 16.4824 | 21 | HN119 | HN150 |
|  |  |  |  |  |  | 1983 | 18.7758 | 24 | HN120 | HN138 |
|  |  |  |  |  |  | 1984 | 22.3534 | 26 | HN120 | HN143 |
|  |  |  |  |  |  | 1985 | 23.7627 | 25 | HN120 | HN143 |
|  |  |  |  |  |  | 1986 | 23.1568 | 28 | HN121 | HN129 |
|  |  |  |  |  |  | 1987 | 17.1864 | 22 | HN121 | HN145 |
|  |  |  |  |  |  | 1988 | 23.0866 | 27 | HN122 | HN127 |
|  |  |  |  |  |  | 1989 | 18.8817 | 23 | HN122 | HN130 |
|  |  |  |  |  |  | 1990 | 21.9099 | 27 | HN122 | HN130 |
|  |  |  |  |  |  | 1991 | 21.9713 | 26 | HN122 | HN138 |
|  |  |  |  |  |  | 1992 | 27.0985 | 28 | HN123 | HN129 |
|  |  |  |  |  |  | 1993 | 24.9407 | 28 | HN123 | HN131 |
|  |  |  |  |  |  | 1994 | 21.9163 | 23 | HN123 | HN140 |
|  |  |  |  |  |  | 1995 | 18.3432 | 22 | HN123 | HN144 |
|  |  |  |  |  |  | 1996 | 23.4977 | 24 | HN124 | HN129 |
|  |  |  |  |  |  | 1997 | 25.215 | 28 | HN124 | HN129 |
|  |  |  |  |  |  | 1998 | 25.382 | 29 | HN124 | HN131 |
|  |  |  |  |  |  | 1999 | 21.7137 | 25 | HN124 | HN132 |
|  |  |  |  |  |  | 2000 | 20.54 | 24 | HN124 | HN136 |
|  |  |  |  |  |  | 2001 | 21.4689 | 23 | HN124 | HN140 |
|  |  |  |  |  |  | 2002 | 20.3531 | 22 | HN124 | HN144 |
|  |  |  |  |  |  | 2003 | 18.4733 | 23 | HN124 | HN147 |
|  |  |  |  |  |  | 2004 | 19.565 | 23 | HN124 | HN149 |
|  |  |  |  |  |  | 2005 | 20.6488 | 25 | HN125 | HN126 |
|  |  |  |  |  |  | 2006 | 19.763 | 23 | HN125 | HN126 |
|  |  |  |  |  |  | 2007 | 23.2036 | 29 | HN125 | HN132 |
|  |  |  |  |  |  | 2008 | 21.9724 | 26 | HN125 | HN136 |
|  |  |  |  |  |  | 2009 | 25.2428 | 27 | HN125 | HN138 |
|  |  |  |  |  |  | 2010 | 20.8581 | 24 | HN125 | HN139 |
|  |  |  |  |  |  | 2011 | 24.2047 | 27 | HN125 | HN144 |
|  |  |  |  |  |  | 2012 | 20.1466 | 20 | HN125 | HN149 |
|  |  |  |  |  |  | 2013 | 21.3363 | 27 | HN126 | HN129 |
|  |  |  |  |  |  | 2014 | 21.1303 | 24 | HN126 | HN144 |
|  |  |  |  |  |  | 2015 | 18.6405 | 23 | HN126 | HN145 |
|  |  |  |  |  |  | 2016 | 20.8571 | 30 | HN127 | HN128 |
|  |  |  |  |  |  | 2017 | 23.193 | 29 | HN127 | HN131 |
|  |  |  |  |  |  | 2018 | 21.518 | 24 | HN127 | HN133 |
|  |  |  |  |  |  | 2019 | 15.7353 | 21 | HN127 | HN139 |
|  |  |  |  |  |  | 2020 | 22.0944 | 24 | HN127 | HN150 |
|  |  |  |  |  |  | 2021 | 19.5288 | 23 | HN127 | HN150 |
|  |  |  |  |  |  | 2022 | 21.6068 | 27 | HN128 | HN131 |
|  |  |  |  |  |  | 2023 | 19.046 | 23 | HN128 | HN148 |
|  |  |  |  |  |  | 2024 | 23.5325 | 28 | HN129 | HN131 |
|  |  |  |  |  |  | 2025 | 21.9402 | 25 | HN129 | HN132 |
|  |  |  |  |  |  | 2026 | 24.4494 | 28 | HN129 | HN133 |
|  |  |  |  |  |  | 2027 | 24.778 | 25 | HN129 | HN134 |
|  |  |  |  |  |  | 2028 | 23.1511 | 27 | HN129 | HN135 |
|  |  |  |  |  |  | 2029 | 23.6994 | 30 | HN129 | HN136 |
|  |  |  |  |  |  | 2030 | 20.3863 | 25 | HN129 | HN141 |
|  |  |  |  |  |  | 2031 | 21.238 | 23 | HN129 | HN142 |
|  |  |  |  |  |  | 2032 | 24.7403 | 24 | HN129 | HN148 |
|  |  |  |  |  |  | 2033 | 22.0543 | 27 | HN130 | HN131 |
|  |  |  |  |  |  | 2034 | 21.2049 | 27 | HN130 | HN143 |
|  |  |  |  |  |  | 2035 | 22.0168 | 28 | HN130 | HN143 |
|  |  |  |  |  |  | 2036 | 24.9269 | 27 | HN131 | HN132 |
|  |  |  |  |  |  | 2037 | 21.1674 | 24 | HN131 | HN132 |
|  |  |  |  |  |  | 2038 | 26.0383 | 31 | HN131 | HN134 |
|  |  |  |  |  |  | 2039 | 25.0293 | 28 | HN131 | HN137 |
|  |  |  |  |  |  | 2040 | 23.1893 | 28 | HN131 | HN139 |
|  |  |  |  |  |  | 2041 | 27.6998 | 28 | HN131 | HN140 |
|  |  |  |  |  |  | 2042 | 21.3661 | 25 | HN132 | HN138 |
|  |  |  |  |  |  | 2043 | 21.746 | 23 | HN132 | HN140 |
|  |  |  |  |  |  | 2044 | 22.0141 | 23 | HN132 | HN147 |
|  |  |  |  |  |  | 2045 | 20.1613 | 21 | HN134 | HN135 |
|  |  |  |  |  |  | 2046 | 21.3714 | 23 | HN134 | HN138 |
|  |  |  |  |  |  | 2047 | 14.6674 | 18 | HN134 | HN145 |
|  |  |  |  |  |  | 2048 | 23.3492 | 26 | HN135 | HN143 |
|  |  |  |  |  |  | 2049 | 21.4061 | 26 | HN136 | HN137 |
|  |  |  |  |  |  | 2050 | 16.568 | 19 | HN136 | HN150 |
|  |  |  |  |  |  | 2051 | 19.6734 | 23 | HN137 | HN142 |
|  |  |  |  |  |  | 2052 | 20.3217 | 25 | HN138 | HN148 |
|  |  |  |  |  |  | 2053 | 19.0352 | 22 | HN139 | HN145 |
|  |  |  |  |  |  | 2054 | 18.635 | 24 | HN140 | HN143 |
|  |  |  |  |  |  | 2055 | 15.8223 | 20 | HN140 | HN143 |
|  |  |  |  |  |  | 2056 | 22.1386 | 24 | HN141 | HN143 |
|  |  |  |  |  |  | 2057 | 18.9715 | 25 | HN141 | HN147 |
|  |  |  |  |  |  | 2058 | 18.1944 | 22 | HN141 | HN149 |
|  |  |  |  |  |  | 2059 | 19.9389 | 22 | HN147 | HN149 |

**Supplementary Table 9.** Simulated breeding results for an F_2_ population size of 500.

| Method-Ped | | | | |  | Method-Bulk | | | | |
| --- | --- | --- | --- | --- | --- | --- | --- | --- | --- | --- |
| Individual | GenoValue | NSA | SCP1 | SCP2 |  | Individual | GenoValue | NSA | SCP1 | SCP2 |
| 1 | 17.581 | 25 | HN002 | HN019 |  | 1 | 23.8125 | 24 | HN002 | HN004 |
| 2 | 13.1217 | 15 | HN002 | HN039 |  | 2 | 16.0324 | 24 | HN002 | HN011 |
| 3 | 21.3615 | 24 | HN002 | HN051 |  | 3 | 24.5655 | 28 | HN002 | HN016 |
| 4 | 24.9236 | 30 | HN002 | HN094 |  | 4 | 20.3508 | 27 | HN002 | HN024 |
| 5 | 19.092 | 23 | HN002 | HN115 |  | 5 | 18.8967 | 21 | HN002 | HN032 |
| 6 | 16.6825 | 18 | HN002 | HN117 |  | 6 | 15.0718 | 19 | HN002 | HN041 |
| 7 | 19.7607 | 20 | HN002 | HN124 |  | 7 | 18.1462 | 23 | HN002 | HN046 |
| 8 | 17.9989 | 20 | HN002 | HN133 |  | 8 | 23.4857 | 25 | HN002 | HN051 |
| 9 | 12.0575 | 18 | HN002 | HN135 |  | 9 | 19.765 | 24 | HN002 | HN090 |
| 10 | 18.3997 | 21 | HN002 | HN136 |  | 10 | 20.8592 | 21 | HN002 | HN090 |
| 11 | 21.8588 | 24 | HN003 | HN016 |  | 11 | 24.4428 | 22 | HN002 | HN113 |
| 12 | 22.1233 | 21 | HN003 | HN016 |  | 12 | 20.8062 | 21 | HN002 | HN116 |
| 13 | 20.0304 | 26 | HN003 | HN039 |  | 13 | 23.2383 | 28 | HN002 | HN125 |
| 14 | 18.2333 | 20 | HN003 | HN062 |  | 14 | 19.5141 | 22 | HN002 | HN143 |
| 15 | 21.6297 | 24 | HN003 | HN064 |  | 15 | 21.5162 | 21 | HN003 | HN007 |
| 16 | 19.8445 | 25 | HN003 | HN065 |  | 16 | 22.6053 | 20 | HN003 | HN018 |
| 17 | 21.0819 | 20 | HN003 | HN067 |  | 17 | 25.1563 | 23 | HN003 | HN022 |
| 18 | 22.1939 | 22 | HN003 | HN070 |  | 18 | 23.1881 | 22 | HN003 | HN024 |
| 19 | 20.9016 | 24 | HN003 | HN073 |  | 19 | 20.1825 | 22 | HN003 | HN024 |
| 20 | 20.8116 | 19 | HN003 | HN082 |  | 20 | 19.6111 | 21 | HN003 | HN025 |
| 21 | 18.9434 | 24 | HN003 | HN103 |  | 21 | 19.4412 | 18 | HN003 | HN032 |
| 22 | 25.8302 | 26 | HN003 | HN116 |  | 22 | 22.7269 | 22 | HN003 | HN051 |
| 23 | 23.0235 | 24 | HN003 | HN118 |  | 23 | 19.7948 | 22 | HN003 | HN059 |
| 24 | 23.3294 | 24 | HN003 | HN127 |  | 24 | 21.8866 | 22 | HN003 | HN061 |
| 25 | 19.8055 | 21 | HN004 | HN009 |  | 25 | 13.1589 | 16 | HN003 | HN063 |
| 26 | 20.7564 | 18 | HN004 | HN010 |  | 26 | 22.1198 | 23 | HN003 | HN065 |
| 27 | 17.0616 | 21 | HN004 | HN019 |  | 27 | 22.227 | 21 | HN003 | HN068 |
| 28 | 22.1392 | 22 | HN004 | HN022 |  | 28 | 19.3411 | 21 | HN003 | HN079 |
| 29 | 27.4439 | 23 | HN004 | HN022 |  | 29 | 20.8783 | 24 | HN003 | HN083 |
| 30 | 21.8855 | 24 | HN004 | HN024 |  | 30 | 22.0344 | 22 | HN003 | HN087 |
| 31 | 27.7718 | 26 | HN004 | HN026 |  | 31 | 18.0271 | 18 | HN003 | HN100 |
| 32 | 23.4955 | 23 | HN004 | HN027 |  | 32 | 16.6091 | 18 | HN003 | HN100 |
| 33 | 23.6935 | 20 | HN004 | HN027 |  | 33 | 23.8262 | 25 | HN003 | HN103 |
| 34 | 22.7596 | 23 | HN004 | HN034 |  | 34 | 22.8154 | 28 | HN003 | HN129 |
| 35 | 25.8849 | 25 | HN004 | HN034 |  | 35 | 20.3476 | 20 | HN003 | HN135 |
| 36 | 24.3574 | 21 | HN004 | HN035 |  | 36 | 20.552 | 19 | HN003 | HN140 |
| 37 | 21.2176 | 22 | HN004 | HN037 |  | 37 | 18.5336 | 17 | HN003 | HN148 |
| 38 | 26.6877 | 26 | HN004 | HN039 |  | 38 | 19.3163 | 18 | HN003 | HN149 |
| 39 | 22.1749 | 22 | HN004 | HN041 |  | 39 | 19.8341 | 22 | HN004 | HN006 |
| 40 | 21.0753 | 21 | HN004 | HN042 |  | 40 | 22.1927 | 21 | HN004 | HN007 |
| 41 | 20.872 | 19 | HN004 | HN043 |  | 41 | 27.0129 | 28 | HN004 | HN010 |
| 42 | 21.1889 | 20 | HN004 | HN043 |  | 42 | 23.2794 | 27 | HN004 | HN011 |
| 43 | 22.3207 | 21 | HN004 | HN043 |  | 43 | 21.3036 | 19 | HN004 | HN014 |
| 44 | 19.2431 | 20 | HN004 | HN044 |  | 44 | 26.5401 | 25 | HN004 | HN016 |
| 45 | 20.1624 | 19 | HN004 | HN049 |  | 45 | 24.099 | 23 | HN004 | HN018 |
| 46 | 23.1771 | 24 | HN004 | HN053 |  | 46 | 17.254 | 19 | HN004 | HN019 |
| 47 | 22.0794 | 22 | HN004 | HN059 |  | 47 | 23 | 21 | HN004 | HN021 |
| 48 | 24.8839 | 26 | HN004 | HN061 |  | 48 | 27.9966 | 26 | HN004 | HN026 |
| 49 | 18.4773 | 20 | HN004 | HN066 |  | 49 | 22.4811 | 20 | HN004 | HN027 |
| 50 | 22.9955 | 23 | HN004 | HN067 |  | 50 | 21.2327 | 18 | HN004 | HN030 |
| 51 | 23.8012 | 22 | HN004 | HN073 |  | 51 | 26.5505 | 24 | HN004 | HN034 |
| 52 | 27.5847 | 25 | HN004 | HN073 |  | 52 | 26.3932 | 27 | HN004 | HN034 |
| 53 | 21.8522 | 21 | HN004 | HN076 |  | 53 | 16.1742 | 15 | HN004 | HN036 |
| 54 | 22.8831 | 23 | HN004 | HN077 |  | 54 | 20.8478 | 22 | HN004 | HN037 |
| 55 | 24.517 | 26 | HN004 | HN083 |  | 55 | 18.7163 | 21 | HN004 | HN037 |
| 56 | 23.9527 | 25 | HN004 | HN090 |  | 56 | 24.0418 | 24 | HN004 | HN045 |
| 57 | 24.6371 | 26 | HN004 | HN096 |  | 57 | 22.9917 | 23 | HN004 | HN045 |
| 58 | 24.1466 | 27 | HN004 | HN096 |  | 58 | 20.4969 | 17 | HN004 | HN046 |
| 59 | 22.519 | 23 | HN004 | HN098 |  | 59 | 20.6611 | 20 | HN004 | HN048 |
| 60 | 23.7796 | 22 | HN004 | HN113 |  | 60 | 19.275 | 25 | HN004 | HN055 |
| 61 | 24.4228 | 25 | HN004 | HN116 |  | 61 | 22.6227 | 22 | HN004 | HN060 |
| 62 | 23.9267 | 23 | HN004 | HN116 |  | 62 | 23.6435 | 25 | HN004 | HN060 |
| 63 | 22.8831 | 22 | HN004 | HN116 |  | 63 | 24.3448 | 23 | HN004 | HN062 |
| 64 | 25.4491 | 27 | HN004 | HN118 |  | 64 | 20.8569 | 21 | HN004 | HN063 |
| 65 | 24.988 | 27 | HN004 | HN126 |  | 65 | 23.6169 | 23 | HN004 | HN066 |
| 66 | 22.473 | 23 | HN004 | HN130 |  | 66 | 20.1796 | 21 | HN004 | HN074 |
| 67 | 24.5828 | 24 | HN004 | HN131 |  | 67 | 24.9815 | 26 | HN004 | HN075 |
| 68 | 20.0297 | 21 | HN004 | HN133 |  | 68 | 21.6995 | 21 | HN004 | HN076 |
| 69 | 20.8577 | 23 | HN004 | HN133 |  | 69 | 20.1594 | 19 | HN004 | HN076 |
| 70 | 22.9578 | 25 | HN004 | HN136 |  | 70 | 24.2307 | 24 | HN004 | HN079 |
| 71 | 22.7678 | 21 | HN004 | HN139 |  | 71 | 25.5954 | 23 | HN004 | HN082 |
| 72 | 20.3401 | 22 | HN004 | HN141 |  | 72 | 26.5121 | 26 | HN004 | HN082 |
| 73 | 23.2769 | 26 | HN004 | HN143 |  | 73 | 24.8232 | 23 | HN004 | HN085 |
| 74 | 25.4088 | 29 | HN004 | HN143 |  | 74 | 20.0935 | 19 | HN004 | HN088 |
| 75 | 22.135 | 19 | HN004 | HN144 |  | 75 | 22.0701 | 27 | HN004 | HN090 |
| 76 | 23.6265 | 24 | HN004 | HN147 |  | 76 | 26.4128 | 25 | HN004 | HN094 |
| 77 | 22.2054 | 22 | HN005 | HN007 |  | 77 | 25.1305 | 26 | HN004 | HN105 |
| 78 | 19.9897 | 22 | HN005 | HN093 |  | 78 | 21.6937 | 17 | HN004 | HN106 |
| 79 | 22.2398 | 27 | HN005 | HN130 |  | 79 | 23.4586 | 23 | HN004 | HN107 |
| 80 | 22.0629 | 27 | HN005 | HN131 |  | 80 | 22.513 | 23 | HN004 | HN114 |
| 81 | 18.8254 | 23 | HN006 | HN011 |  | 81 | 20.4811 | 26 | HN004 | HN119 |
| 82 | 16.4529 | 19 | HN006 | HN015 |  | 82 | 19.6492 | 21 | HN004 | HN125 |
| 83 | 20.3407 | 23 | HN006 | HN016 |  | 83 | 24.1455 | 28 | HN004 | HN127 |
| 84 | 19.9963 | 24 | HN006 | HN017 |  | 84 | 27.5991 | 29 | HN004 | HN129 |
| 85 | 20.3375 | 24 | HN006 | HN018 |  | 85 | 24.8112 | 29 | HN004 | HN130 |
| 86 | 25.4865 | 24 | HN006 | HN022 |  | 86 | 22.5492 | 28 | HN004 | HN130 |
| 87 | 24.0247 | 24 | HN006 | HN022 |  | 87 | 23.592 | 25 | HN004 | HN136 |
| 88 | 24.2571 | 26 | HN006 | HN026 |  | 88 | 24.6371 | 24 | HN004 | HN138 |
| 89 | 19.1538 | 23 | HN006 | HN060 |  | 89 | 24.6978 | 25 | HN004 | HN138 |
| 90 | 17.0986 | 21 | HN006 | HN077 |  | 90 | 24.4804 | 25 | HN004 | HN144 |
| 91 | 19.0582 | 22 | HN006 | HN085 |  | 91 | 20.7082 | 21 | HN004 | HN148 |
| 92 | 21.2733 | 22 | HN006 | HN097 |  | 92 | 18.4817 | 17 | HN004 | HN150 |
| 93 | 19.6173 | 24 | HN006 | HN098 |  | 93 | 23.5243 | 22 | HN005 | HN007 |
| 94 | 16.0547 | 19 | HN006 | HN100 |  | 94 | 20.0775 | 25 | HN005 | HN020 |
| 95 | 16.2198 | 18 | HN006 | HN104 |  | 95 | 18.9096 | 23 | HN005 | HN024 |
| 96 | 17.5271 | 23 | HN006 | HN140 |  | 96 | 17.3393 | 18 | HN005 | HN036 |
| 97 | 17.9865 | 20 | HN006 | HN148 |  | 97 | 18.0482 | 22 | HN005 | HN064 |
| 98 | 20.0072 | 23 | HN006 | HN148 |  | 98 | 18.4371 | 21 | HN005 | HN111 |
| 99 | 20.5718 | 22 | HN007 | HN009 |  | 99 | 17.9452 | 21 | HN005 | HN128 |
| 100 | 27.4696 | 28 | HN007 | HN012 |  | 100 | 21.7247 | 29 | HN005 | HN129 |
| 101 | 21.9943 | 22 | HN007 | HN015 |  | 101 | 15.3425 | 17 | HN005 | HN134 |
| 102 | 18.8679 | 24 | HN007 | HN019 |  | 102 | 16.9293 | 24 | HN005 | HN143 |
| 103 | 21.9073 | 24 | HN007 | HN020 |  | 103 | 17.5469 | 21 | HN006 | HN029 |
| 104 | 26.9225 | 25 | HN007 | HN022 |  | 104 | 18.1905 | 20 | HN006 | HN032 |
| 105 | 23.9038 | 24 | HN007 | HN037 |  | 105 | 19.8565 | 23 | HN006 | HN057 |
| 106 | 25.7503 | 26 | HN007 | HN041 |  | 106 | 19.0945 | 19 | HN006 | HN070 |
| 107 | 24.856 | 22 | HN007 | HN043 |  | 107 | 18.0603 | 23 | HN006 | HN072 |
| 108 | 20.9787 | 23 | HN007 | HN045 |  | 108 | 23.1038 | 30 | HN006 | HN083 |
| 109 | 26.3227 | 26 | HN007 | HN047 |  | 109 | 21.2521 | 27 | HN006 | HN103 |
| 110 | 21.2564 | 20 | HN007 | HN049 |  | 110 | 21.1585 | 24 | HN006 | HN129 |
| 111 | 25.61 | 26 | HN007 | HN051 |  | 111 | 23.0824 | 25 | HN006 | HN131 |
| 112 | 24.4418 | 26 | HN007 | HN053 |  | 112 | 23.4646 | 21 | HN007 | HN014 |
| 113 | 23.917 | 24 | HN007 | HN054 |  | 113 | 25.4368 | 24 | HN007 | HN021 |
| 114 | 19.5211 | 22 | HN007 | HN054 |  | 114 | 25.324 | 24 | HN007 | HN022 |
| 115 | 21.0778 | 20 | HN007 | HN058 |  | 115 | 26.4096 | 26 | HN007 | HN034 |
| 116 | 22.2818 | 25 | HN007 | HN061 |  | 116 | 20.6053 | 22 | HN007 | HN036 |
| 117 | 21.8519 | 22 | HN007 | HN062 |  | 117 | 23.895 | 27 | HN007 | HN045 |
| 118 | 24.4813 | 28 | HN007 | HN065 |  | 118 | 24.7644 | 25 | HN007 | HN046 |
| 119 | 24.1848 | 23 | HN007 | HN069 |  | 119 | 22.6015 | 25 | HN007 | HN051 |
| 120 | 21.9537 | 23 | HN007 | HN069 |  | 120 | 23.9892 | 25 | HN007 | HN052 |
| 121 | 20.6053 | 22 | HN007 | HN071 |  | 121 | 21.6518 | 24 | HN007 | HN053 |
| 122 | 22.2894 | 23 | HN007 | HN075 |  | 122 | 24.4111 | 25 | HN007 | HN056 |
| 123 | 23.3251 | 24 | HN007 | HN077 |  | 123 | 22.5669 | 24 | HN007 | HN058 |
| 124 | 20.5581 | 21 | HN007 | HN080 |  | 124 | 26.5996 | 28 | HN007 | HN060 |
| 125 | 23.5937 | 24 | HN007 | HN087 |  | 125 | 23.3628 | 24 | HN007 | HN061 |
| 126 | 22.1735 | 21 | HN007 | HN095 |  | 126 | 23.2649 | 23 | HN007 | HN066 |
| 127 | 21.5931 | 22 | HN007 | HN097 |  | 127 | 22.8716 | 23 | HN007 | HN069 |
| 128 | 24.2885 | 25 | HN007 | HN103 |  | 128 | 24.0062 | 24 | HN007 | HN069 |
| 129 | 21.2441 | 23 | HN007 | HN103 |  | 129 | 23.337 | 23 | HN007 | HN077 |
| 130 | 21.0982 | 18 | HN007 | HN106 |  | 130 | 28.0525 | 28 | HN007 | HN080 |
| 131 | 16.3593 | 13 | HN007 | HN106 |  | 131 | 22.6752 | 21 | HN007 | HN082 |
| 132 | 25.0816 | 25 | HN007 | HN111 |  | 132 | 20.5694 | 22 | HN007 | HN085 |
| 133 | 20.1457 | 22 | HN007 | HN114 |  | 133 | 22.7762 | 23 | HN007 | HN090 |
| 134 | 24.2057 | 23 | HN007 | HN114 |  | 134 | 24.403 | 25 | HN007 | HN093 |
| 135 | 21.3406 | 24 | HN007 | HN118 |  | 135 | 21.4672 | 19 | HN007 | HN097 |
| 136 | 20.7072 | 23 | HN007 | HN123 |  | 136 | 23.4176 | 24 | HN007 | HN107 |
| 137 | 19.3461 | 24 | HN007 | HN123 |  | 137 | 21.0021 | 22 | HN007 | HN107 |
| 138 | 23.4269 | 25 | HN007 | HN125 |  | 138 | 23.6241 | 25 | HN007 | HN111 |
| 139 | 27.6485 | 29 | HN007 | HN129 |  | 139 | 24.8639 | 23 | HN007 | HN114 |
| 140 | 24.5743 | 27 | HN007 | HN131 |  | 140 | 22.8829 | 23 | HN007 | HN116 |
| 141 | 20.0852 | 24 | HN007 | HN131 |  | 141 | 22.6682 | 23 | HN007 | HN117 |
| 142 | 22.9822 | 21 | HN007 | HN137 |  | 142 | 23.2975 | 24 | HN007 | HN122 |
| 143 | 23.1283 | 20 | HN007 | HN139 |  | 143 | 23.5105 | 23 | HN007 | HN122 |
| 144 | 16.5925 | 22 | HN007 | HN144 |  | 144 | 24.4571 | 24 | HN007 | HN123 |
| 145 | 18.2038 | 20 | HN007 | HN149 |  | 145 | 19.9497 | 21 | HN007 | HN126 |
| 146 | 20.1776 | 20 | HN007 | HN150 |  | 146 | 24.2969 | 27 | HN007 | HN127 |
| 147 | 20.2731 | 26 | HN008 | HN009 |  | 147 | 26.7186 | 28 | HN007 | HN130 |
| 148 | 23.237 | 26 | HN008 | HN016 |  | 148 | 23.3197 | 24 | HN007 | HN133 |
| 149 | 19.9116 | 23 | HN008 | HN022 |  | 149 | 25.7156 | 25 | HN007 | HN137 |
| 150 | 21.9741 | 27 | HN008 | HN051 |  | 150 | 21.6518 | 24 | HN007 | HN138 |
| 151 | 21.4914 | 26 | HN008 | HN051 |  | 151 | 22.5535 | 24 | HN007 | HN138 |
| 152 | 20.7585 | 25 | HN008 | HN062 |  | 152 | 24.8484 | 25 | HN007 | HN140 |
| 153 | 18.837 | 24 | HN008 | HN064 |  | 153 | 21.0263 | 25 | HN007 | HN147 |
| 154 | 22.9469 | 28 | HN008 | HN064 |  | 154 | 22.0746 | 25 | HN008 | HN012 |
| 155 | 22.3732 | 27 | HN008 | HN065 |  | 155 | 14.9523 | 20 | HN008 | HN019 |
| 156 | 23.048 | 30 | HN008 | HN072 |  | 156 | 22.8205 | 27 | HN008 | HN022 |
| 157 | 19.0254 | 25 | HN008 | HN075 |  | 157 | 23.7865 | 22 | HN008 | HN022 |
| 158 | 25.2581 | 27 | HN008 | HN113 |  | 158 | 25.2441 | 27 | HN008 | HN022 |
| 159 | 20.3243 | 21 | HN008 | HN116 |  | 159 | 23.2964 | 26 | HN008 | HN034 |
| 160 | 18.0271 | 23 | HN008 | HN116 |  | 160 | 15.0998 | 21 | HN008 | HN036 |
| 161 | 22.8236 | 25 | HN008 | HN138 |  | 161 | 21.7794 | 24 | HN008 | HN049 |
| 162 | 21.4294 | 26 | HN008 | HN143 |  | 162 | 19.5762 | 24 | HN008 | HN056 |
| 163 | 21.4294 | 25 | HN008 | HN143 |  | 163 | 19.7572 | 24 | HN008 | HN067 |
| 164 | 24.0223 | 29 | HN009 | HN012 |  | 164 | 19.8282 | 26 | HN008 | HN103 |
| 165 | 22.7045 | 27 | HN009 | HN020 |  | 165 | 21.2574 | 25 | HN008 | HN108 |
| 166 | 19.3656 | 24 | HN009 | HN023 |  | 166 | 17.2531 | 22 | HN008 | HN117 |
| 167 | 23.6462 | 27 | HN009 | HN034 |  | 167 | 23.4092 | 26 | HN008 | HN125 |
| 168 | 22.3244 | 25 | HN009 | HN041 |  | 168 | 20.7515 | 24 | HN008 | HN132 |
| 169 | 20.8643 | 24 | HN009 | HN046 |  | 169 | 22.9321 | 25 | HN008 | HN138 |
| 170 | 20.7883 | 23 | HN009 | HN048 |  | 170 | 17.9122 | 23 | HN008 | HN141 |
| 171 | 24.2071 | 29 | HN009 | HN050 |  | 171 | 19.9446 | 20 | HN008 | HN145 |
| 172 | 21.1206 | 26 | HN009 | HN051 |  | 172 | 21.9732 | 26 | HN008 | HN147 |
| 173 | 21.221 | 26 | HN009 | HN051 |  | 173 | 16.7551 | 23 | HN008 | HN150 |
| 174 | 24.0889 | 28 | HN009 | HN060 |  | 174 | 20.6635 | 25 | HN009 | HN010 |
| 175 | 22.733 | 30 | HN009 | HN065 |  | 175 | 18.2189 | 21 | HN009 | HN015 |
| 176 | 23.7047 | 28 | HN009 | HN071 |  | 176 | 21.1399 | 27 | HN009 | HN017 |
| 177 | 22.4861 | 26 | HN009 | HN086 |  | 177 | 24.417 | 29 | HN009 | HN018 |
| 178 | 20.2935 | 24 | HN009 | HN087 |  | 178 | 24.4493 | 28 | HN009 | HN021 |
| 179 | 22.8831 | 28 | HN009 | HN090 |  | 179 | 25.2231 | 28 | HN009 | HN022 |
| 180 | 21.5098 | 23 | HN009 | HN092 |  | 180 | 21.7682 | 25 | HN009 | HN035 |
| 181 | 22.0392 | 26 | HN009 | HN098 |  | 181 | 21.3722 | 26 | HN009 | HN043 |
| 182 | 22.8795 | 26 | HN009 | HN105 |  | 182 | 23.1807 | 25 | HN009 | HN046 |
| 183 | 19.967 | 26 | HN009 | HN109 |  | 183 | 23.1092 | 26 | HN009 | HN053 |
| 184 | 20.3489 | 26 | HN009 | HN109 |  | 184 | 18.7838 | 27 | HN009 | HN053 |
| 185 | 25.0552 | 26 | HN009 | HN111 |  | 185 | 22.7159 | 27 | HN009 | HN056 |
| 186 | 22.032 | 27 | HN009 | HN130 |  | 186 | 21.4558 | 27 | HN009 | HN056 |
| 187 | 20.4917 | 24 | HN009 | HN132 |  | 187 | 22.7532 | 27 | HN009 | HN060 |
| 188 | 20.6666 | 26 | HN009 | HN143 |  | 188 | 23.3907 | 28 | HN009 | HN062 |
| 189 | 20.0585 | 24 | HN009 | HN146 |  | 189 | 22.0099 | 26 | HN009 | HN064 |
| 190 | 26.385 | 26 | HN010 | HN014 |  | 190 | 20.9047 | 23 | HN009 | HN066 |
| 191 | 19.4181 | 23 | HN010 | HN015 |  | 191 | 20.8953 | 26 | HN009 | HN067 |
| 192 | 21.2661 | 26 | HN010 | HN016 |  | 192 | 24.8307 | 28 | HN009 | HN068 |
| 193 | 23.0385 | 26 | HN010 | HN020 |  | 193 | 20.4268 | 23 | HN009 | HN075 |
| 194 | 25.308 | 25 | HN010 | HN022 |  | 194 | 22.4582 | 28 | HN009 | HN094 |
| 195 | 25.0265 | 26 | HN010 | HN022 |  | 195 | 20.9796 | 25 | HN009 | HN095 |
| 196 | 21.4907 | 28 | HN010 | HN024 |  | 196 | 24.485 | 30 | HN009 | HN096 |
| 197 | 22.5171 | 26 | HN010 | HN041 |  | 197 | 24.3458 | 31 | HN009 | HN101 |
| 198 | 25.9351 | 29 | HN010 | HN043 |  | 198 | 24.6503 | 29 | HN009 | HN103 |
| 199 | 17.8508 | 25 | HN010 | HN045 |  | 199 | 25.9224 | 28 | HN009 | HN116 |
| 200 | 22.6565 | 26 | HN010 | HN060 |  | 200 | 24.9969 | 29 | HN009 | HN116 |
| 201 | 21.8904 | 26 | HN010 | HN066 |  | 201 | 24.9969 | 29 | HN009 | HN116 |
| 202 | 22.8319 | 28 | HN010 | HN068 |  | 202 | 24.7771 | 24 | HN009 | HN116 |
| 203 | 26.5689 | 29 | HN010 | HN068 |  | 203 | 20.3232 | 25 | HN009 | HN121 |
| 204 | 19.2137 | 25 | HN010 | HN072 |  | 204 | 20.3232 | 25 | HN009 | HN121 |
| 205 | 23.6084 | 29 | HN010 | HN077 |  | 205 | 23.8336 | 29 | HN009 | HN122 |
| 206 | 17.328 | 22 | HN010 | HN079 |  | 206 | 20.7204 | 27 | HN009 | HN133 |
| 207 | 23.3873 | 25 | HN010 | HN082 |  | 207 | 18.5053 | 25 | HN009 | HN141 |
| 208 | 20.519 | 28 | HN010 | HN090 |  | 208 | 21.849 | 26 | HN010 | HN011 |
| 209 | 21.0475 | 21 | HN010 | HN093 |  | 209 | 22.2845 | 24 | HN010 | HN019 |
| 210 | 23.0082 | 28 | HN010 | HN098 |  | 210 | 24.6465 | 28 | HN010 | HN022 |
| 211 | 22.9209 | 28 | HN010 | HN105 |  | 211 | 21.3791 | 22 | HN010 | HN026 |
| 212 | 22.5444 | 28 | HN010 | HN105 |  | 212 | 24.5282 | 26 | HN010 | HN034 |
| 213 | 22.9112 | 19 | HN010 | HN106 |  | 213 | 18.6618 | 25 | HN010 | HN036 |
| 214 | 20.4358 | 25 | HN010 | HN110 |  | 214 | 22.4271 | 26 | HN010 | HN036 |
| 215 | 25.3081 | 28 | HN010 | HN112 |  | 215 | 20.4754 | 24 | HN010 | HN037 |
| 216 | 21.4488 | 25 | HN010 | HN115 |  | 216 | 21.4162 | 28 | HN010 | HN040 |
| 217 | 27.4774 | 29 | HN010 | HN125 |  | 217 | 20.2977 | 24 | HN010 | HN041 |
| 218 | 19.8601 | 22 | HN010 | HN136 |  | 218 | 22.1183 | 27 | HN010 | HN043 |
| 219 | 18.9661 | 22 | HN010 | HN144 |  | 219 | 24.1469 | 27 | HN010 | HN050 |
| 220 | 26.2501 | 27 | HN011 | HN022 |  | 220 | 21.4602 | 27 | HN010 | HN052 |
| 221 | 20.7887 | 24 | HN011 | HN026 |  | 221 | 24.2282 | 27 | HN010 | HN060 |
| 222 | 25.2849 | 26 | HN011 | HN034 |  | 222 | 25.196 | 30 | HN010 | HN065 |
| 223 | 19.9692 | 24 | HN011 | HN051 |  | 223 | 22.4697 | 28 | HN010 | HN068 |
| 224 | 20.8665 | 26 | HN011 | HN067 |  | 224 | 22.7885 | 28 | HN010 | HN072 |
| 225 | 23.7457 | 25 | HN011 | HN073 |  | 225 | 19.6992 | 26 | HN010 | HN074 |
| 226 | 21.4493 | 26 | HN011 | HN091 |  | 226 | 25.3058 | 32 | HN010 | HN083 |
| 227 | 22.4038 | 29 | HN011 | HN094 |  | 227 | 25.5758 | 28 | HN010 | HN084 |
| 228 | 17.5386 | 22 | HN011 | HN104 |  | 228 | 18.0736 | 23 | HN010 | HN088 |
| 229 | 14.9315 | 22 | HN011 | HN106 |  | 229 | 22.5383 | 25 | HN010 | HN091 |
| 230 | 22.0973 | 26 | HN011 | HN107 |  | 230 | 21.3753 | 26 | HN010 | HN096 |
| 231 | 22.4806 | 27 | HN011 | HN107 |  | 231 | 25.9135 | 27 | HN010 | HN097 |
| 232 | 19.5013 | 26 | HN011 | HN113 |  | 232 | 18.3231 | 22 | HN010 | HN104 |
| 233 | 25.5335 | 28 | HN011 | HN116 |  | 233 | 21.7633 | 26 | HN010 | HN109 |
| 234 | 22.1178 | 24 | HN012 | HN014 |  | 234 | 22.981 | 26 | HN010 | HN116 |
| 235 | 26.0688 | 30 | HN012 | HN016 |  | 235 | 16.6595 | 20 | HN010 | HN117 |
| 236 | 23.2992 | 29 | HN012 | HN017 |  | 236 | 26.253 | 29 | HN010 | HN125 |
| 237 | 25.6144 | 27 | HN012 | HN021 |  | 237 | 26.115 | 30 | HN010 | HN137 |
| 238 | 27.2046 | 31 | HN012 | HN021 |  | 238 | 20.1548 | 21 | HN010 | HN140 |
| 239 | 24.189 | 21 | HN012 | HN022 |  | 239 | 20.5462 | 24 | HN010 | HN142 |
| 240 | 17.9189 | 22 | HN012 | HN026 |  | 240 | 18.198 | 20 | HN011 | HN015 |
| 241 | 20.0849 | 22 | HN012 | HN030 |  | 241 | 20.5585 | 27 | HN011 | HN024 |
| 242 | 20.0275 | 26 | HN012 | HN033 |  | 242 | 22.1461 | 27 | HN011 | HN034 |
| 243 | 20.4267 | 24 | HN012 | HN033 |  | 243 | 23.8879 | 26 | HN011 | HN043 |
| 244 | 29.2061 | 32 | HN012 | HN034 |  | 244 | 21.018 | 25 | HN011 | HN051 |
| 245 | 19.7142 | 24 | HN012 | HN036 |  | 245 | 23.7863 | 28 | HN011 | HN064 |
| 246 | 24.7858 | 30 | HN012 | HN037 |  | 246 | 21.3765 | 24 | HN011 | HN064 |
| 247 | 21.3706 | 26 | HN012 | HN054 |  | 247 | 20.5853 | 26 | HN011 | HN072 |
| 248 | 27.0965 | 30 | HN012 | HN058 |  | 248 | 27.3049 | 33 | HN011 | HN083 |
| 249 | 20.1505 | 25 | HN012 | HN060 |  | 249 | 23.7834 | 27 | HN011 | HN117 |
| 250 | 21.9557 | 27 | HN012 | HN060 |  | 250 | 18.822 | 23 | HN011 | HN124 |
| 251 | 24.9081 | 28 | HN012 | HN060 |  | 251 | 21.9228 | 26 | HN011 | HN125 |
| 252 | 23.5149 | 25 | HN012 | HN061 |  | 252 | 26.2248 | 29 | HN012 | HN016 |
| 253 | 22.6099 | 28 | HN012 | HN072 |  | 253 | 25.9706 | 30 | HN012 | HN020 |
| 254 | 24.9993 | 27 | HN012 | HN090 |  | 254 | 23.1293 | 28 | HN012 | HN022 |
| 255 | 24.4995 | 29 | HN012 | HN092 |  | 255 | 26.5911 | 30 | HN012 | HN034 |
| 256 | 28.8698 | 32 | HN012 | HN096 |  | 256 | 24.5304 | 28 | HN012 | HN037 |
| 257 | 23.9415 | 25 | HN012 | HN108 |  | 257 | 20.8456 | 24 | HN012 | HN040 |
| 258 | 26.8016 | 28 | HN012 | HN113 |  | 258 | 25.5918 | 27 | HN012 | HN043 |
| 259 | 21.5303 | 24 | HN012 | HN118 |  | 259 | 20.1277 | 22 | HN012 | HN045 |
| 260 | 22.3757 | 27 | HN012 | HN121 |  | 260 | 22.6057 | 26 | HN012 | HN046 |
| 261 | 23.023 | 28 | HN012 | HN123 |  | 261 | 24.0729 | 28 | HN012 | HN055 |
| 262 | 21.1339 | 26 | HN012 | HN124 |  | 262 | 26.4801 | 29 | HN012 | HN068 |
| 263 | 22.7054 | 31 | HN012 | HN129 |  | 263 | 21.6136 | 27 | HN012 | HN069 |
| 264 | 26.6138 | 30 | HN012 | HN131 |  | 264 | 23.3092 | 26 | HN012 | HN070 |
| 265 | 25.981 | 31 | HN012 | HN131 |  | 265 | 23.6963 | 29 | HN012 | HN072 |
| 266 | 26.8888 | 29 | HN012 | HN131 |  | 266 | 22.9918 | 29 | HN012 | HN072 |
| 267 | 17.767 | 21 | HN012 | HN134 |  | 267 | 25.5315 | 30 | HN012 | HN073 |
| 268 | 20.6851 | 28 | HN012 | HN136 |  | 268 | 19.2011 | 24 | HN012 | HN074 |
| 269 | 22.6366 | 23 | HN014 | HN017 |  | 269 | 27.7815 | 29 | HN012 | HN082 |
| 270 | 25.0165 | 24 | HN014 | HN018 |  | 270 | 27.4903 | 28 | HN012 | HN082 |
| 271 | 25.6792 | 24 | HN014 | HN020 |  | 271 | 28.0435 | 35 | HN012 | HN083 |
| 272 | 24.5579 | 25 | HN014 | HN020 |  | 272 | 16.0204 | 20 | HN012 | HN084 |
| 273 | 26.2057 | 25 | HN014 | HN020 |  | 273 | 24.366 | 29 | HN012 | HN096 |
| 274 | 20.4047 | 21 | HN014 | HN021 |  | 274 | 25.5246 | 26 | HN012 | HN104 |
| 275 | 19.6686 | 19 | HN014 | HN023 |  | 275 | 23.734 | 29 | HN012 | HN108 |
| 276 | 19.8488 | 20 | HN014 | HN027 |  | 276 | 22.8541 | 26 | HN012 | HN110 |
| 277 | 25.0435 | 22 | HN014 | HN035 |  | 277 | 21.611 | 24 | HN012 | HN110 |
| 278 | 22.052 | 23 | HN014 | HN037 |  | 278 | 20.8004 | 24 | HN012 | HN113 |
| 279 | 22.9756 | 24 | HN014 | HN039 |  | 279 | 24.6612 | 27 | HN012 | HN113 |
| 280 | 24.278 | 26 | HN014 | HN039 |  | 280 | 24.7243 | 27 | HN012 | HN116 |
| 281 | 25.1118 | 26 | HN014 | HN039 |  | 281 | 26.5113 | 28 | HN012 | HN116 |
| 282 | 22.2555 | 25 | HN014 | HN041 |  | 282 | 22.6197 | 25 | HN012 | HN117 |
| 283 | 23.2959 | 22 | HN014 | HN048 |  | 283 | 23.8887 | 26 | HN012 | HN118 |
| 284 | 22.8672 | 26 | HN014 | HN050 |  | 284 | 19.8389 | 24 | HN012 | HN119 |
| 285 | 22.0762 | 24 | HN014 | HN057 |  | 285 | 22.7858 | 29 | HN012 | HN124 |
| 286 | 22.6351 | 22 | HN014 | HN061 |  | 286 | 22.6812 | 27 | HN012 | HN125 |
| 287 | 22.094 | 20 | HN014 | HN064 |  | 287 | 20.6912 | 26 | HN012 | HN126 |
| 288 | 19.6161 | 22 | HN014 | HN065 |  | 288 | 25.1951 | 29 | HN012 | HN130 |
| 289 | 21.8619 | 24 | HN014 | HN065 |  | 289 | 22.0198 | 29 | HN012 | HN130 |
| 290 | 21.193 | 20 | HN014 | HN069 |  | 290 | 21.581 | 24 | HN012 | HN136 |
| 291 | 25.8916 | 23 | HN014 | HN073 |  | 291 | 23.5825 | 27 | HN012 | HN145 |
| 292 | 25.6259 | 22 | HN014 | HN073 |  | 292 | 22.6738 | 27 | HN012 | HN147 |
| 293 | 23.5272 | 22 | HN014 | HN076 |  | 293 | 23.7724 | 23 | HN014 | HN016 |
| 294 | 23.2518 | 24 | HN014 | HN077 |  | 294 | 23.9661 | 24 | HN014 | HN017 |
| 295 | 22.2123 | 22 | HN014 | HN082 |  | 295 | 26.2735 | 23 | HN014 | HN020 |
| 296 | 23.7621 | 22 | HN014 | HN082 |  | 296 | 21.5485 | 20 | HN014 | HN022 |
| 297 | 22.6316 | 21 | HN014 | HN082 |  | 297 | 25.1667 | 23 | HN014 | HN022 |
| 298 | 22.0774 | 22 | HN014 | HN082 |  | 298 | 25.1847 | 24 | HN014 | HN026 |
| 299 | 27.0588 | 29 | HN014 | HN096 |  | 299 | 23.9983 | 25 | HN014 | HN033 |
| 300 | 21.4505 | 20 | HN014 | HN103 |  | 300 | 26.6548 | 24 | HN014 | HN034 |
| 301 | 21.8377 | 24 | HN014 | HN103 |  | 301 | 23.5874 | 21 | HN014 | HN035 |
| 302 | 24.4379 | 27 | HN014 | HN103 |  | 302 | 21.6294 | 22 | HN014 | HN035 |
| 303 | 20.5565 | 21 | HN014 | HN103 |  | 303 | 20.1819 | 20 | HN014 | HN040 |
| 304 | 19.9426 | 19 | HN014 | HN104 |  | 304 | 24.8968 | 26 | HN014 | HN047 |
| 305 | 21.7908 | 21 | HN014 | HN108 |  | 305 | 18.9324 | 20 | HN014 | HN049 |
| 306 | 20.3461 | 20 | HN014 | HN109 |  | 306 | 23.5593 | 23 | HN014 | HN050 |
| 307 | 23.9332 | 23 | HN014 | HN110 |  | 307 | 22.1178 | 24 | HN014 | HN050 |
| 308 | 18.3006 | 20 | HN014 | HN111 |  | 308 | 24.0251 | 26 | HN014 | HN051 |
| 309 | 21.479 | 21 | HN014 | HN112 |  | 309 | 19.5115 | 20 | HN014 | HN053 |
| 310 | 23.6266 | 21 | HN014 | HN113 |  | 310 | 26.9558 | 30 | HN014 | HN055 |
| 311 | 23.6399 | 23 | HN014 | HN113 |  | 311 | 21.1149 | 21 | HN014 | HN057 |
| 312 | 23.154 | 22 | HN014 | HN117 |  | 312 | 22.1357 | 22 | HN014 | HN061 |
| 313 | 25.4008 | 24 | HN014 | HN129 |  | 313 | 23.2655 | 24 | HN014 | HN068 |
| 314 | 23.3487 | 22 | HN014 | HN138 |  | 314 | 26.8514 | 29 | HN014 | HN083 |
| 315 | 22.1037 | 21 | HN014 | HN141 |  | 315 | 27.2102 | 27 | HN014 | HN083 |
| 316 | 24.2903 | 21 | HN014 | HN143 |  | 316 | 22.7224 | 27 | HN014 | HN094 |
| 317 | 18.1728 | 18 | HN014 | HN148 |  | 317 | 23.4753 | 27 | HN014 | HN096 |
| 318 | 22.0775 | 25 | HN015 | HN039 |  | 318 | 23.1375 | 23 | HN014 | HN098 |
| 319 | 19.2331 | 20 | HN015 | HN041 |  | 319 | 24.9185 | 25 | HN014 | HN101 |
| 320 | 22.0378 | 20 | HN015 | HN043 |  | 320 | 22.4976 | 23 | HN014 | HN107 |
| 321 | 19.0173 | 20 | HN015 | HN049 |  | 321 | 21.1207 | 20 | HN014 | HN111 |
| 322 | 21.4525 | 25 | HN015 | HN053 |  | 322 | 21.2336 | 20 | HN014 | HN113 |
| 323 | 17.4051 | 21 | HN015 | HN069 |  | 323 | 24.9719 | 28 | HN014 | HN118 |
| 324 | 25.6232 | 30 | HN015 | HN083 |  | 324 | 24.0117 | 25 | HN014 | HN123 |
| 325 | 24.187 | 29 | HN015 | HN083 |  | 325 | 20.4742 | 22 | HN014 | HN124 |
| 326 | 22.4685 | 26 | HN015 | HN083 |  | 326 | 20.6296 | 23 | HN014 | HN125 |
| 327 | 25.0724 | 31 | HN015 | HN083 |  | 327 | 24.5994 | 29 | HN014 | HN127 |
| 328 | 23.4345 | 27 | HN015 | HN083 |  | 328 | 23.1756 | 24 | HN014 | HN128 |
| 329 | 20.8468 | 23 | HN015 | HN096 |  | 329 | 26.0384 | 24 | HN014 | HN129 |
| 330 | 20.2651 | 26 | HN015 | HN109 |  | 330 | 27.974 | 28 | HN014 | HN129 |
| 331 | 20.5793 | 25 | HN015 | HN130 |  | 331 | 25.8025 | 26 | HN014 | HN129 |
| 332 | 21.6407 | 26 | HN015 | HN131 |  | 332 | 24.518 | 26 | HN014 | HN131 |
| 333 | 20.4046 | 23 | HN015 | HN138 |  | 333 | 23.9245 | 23 | HN014 | HN133 |
| 334 | 17.8154 | 19 | HN015 | HN139 |  | 334 | 24.7456 | 26 | HN014 | HN134 |
| 335 | 20.255 | 24 | HN016 | HN018 |  | 335 | 24.2598 | 22 | HN014 | HN144 |
| 336 | 23.2758 | 27 | HN016 | HN020 |  | 336 | 21.9259 | 19 | HN014 | HN149 |
| 337 | 24.427 | 27 | HN016 | HN020 |  | 337 | 19.4097 | 23 | HN015 | HN035 |
| 338 | 20.7264 | 23 | HN016 | HN021 |  | 338 | 19.0326 | 22 | HN015 | HN051 |
| 339 | 21.5147 | 26 | HN016 | HN023 |  | 339 | 20.0627 | 25 | HN015 | HN051 |
| 340 | 17.1012 | 20 | HN016 | HN023 |  | 340 | 20.6395 | 27 | HN015 | HN055 |
| 341 | 18.7557 | 19 | HN016 | HN031 |  | 341 | 23.1484 | 26 | HN015 | HN064 |
| 342 | 23.5467 | 22 | HN016 | HN032 |  | 342 | 21.5171 | 21 | HN015 | HN067 |
| 343 | 24.639 | 25 | HN016 | HN032 |  | 343 | 25.8501 | 31 | HN015 | HN083 |
| 344 | 24.0935 | 27 | HN016 | HN037 |  | 344 | 23.423 | 21 | HN015 | HN097 |
| 345 | 24.898 | 28 | HN016 | HN037 |  | 345 | 20.7811 | 23 | HN015 | HN098 |
| 346 | 26.964 | 29 | HN016 | HN043 |  | 346 | 18.0921 | 20 | HN015 | HN101 |
| 347 | 19.8578 | 24 | HN016 | HN046 |  | 347 | 15.7385 | 19 | HN015 | HN102 |
| 348 | 25.1141 | 26 | HN016 | HN047 |  | 348 | 19.4498 | 20 | HN015 | HN104 |
| 349 | 24.6211 | 29 | HN016 | HN053 |  | 349 | 22.0558 | 23 | HN015 | HN106 |
| 350 | 22.41 | 25 | HN016 | HN058 |  | 350 | 22.9692 | 24 | HN015 | HN116 |
| 351 | 23.1703 | 27 | HN016 | HN060 |  | 351 | 17.7089 | 21 | HN015 | HN122 |
| 352 | 25.5461 | 28 | HN016 | HN069 |  | 352 | 19.5098 | 22 | HN015 | HN126 |
| 353 | 24.8542 | 27 | HN016 | HN069 |  | 353 | 17.9489 | 23 | HN015 | HN128 |
| 354 | 25.0264 | 26 | HN016 | HN069 |  | 354 | 24.2599 | 27 | HN015 | HN131 |
| 355 | 20.4002 | 24 | HN016 | HN075 |  | 355 | 22.783 | 25 | HN016 | HN022 |
| 356 | 21.5636 | 23 | HN016 | HN076 |  | 356 | 22.289 | 26 | HN016 | HN024 |
| 357 | 24.1253 | 24 | HN016 | HN082 |  | 357 | 25.1774 | 28 | HN016 | HN024 |
| 358 | 17.2708 | 20 | HN016 | HN085 |  | 358 | 21.0777 | 21 | HN016 | HN030 |
| 359 | 21.6103 | 25 | HN016 | HN090 |  | 359 | 21.8421 | 25 | HN016 | HN033 |
| 360 | 22.2937 | 20 | HN016 | HN097 |  | 360 | 20.9482 | 25 | HN016 | HN040 |
| 361 | 26.4664 | 24 | HN016 | HN097 |  | 361 | 22.7637 | 24 | HN016 | HN041 |
| 362 | 21.888 | 25 | HN016 | HN102 |  | 362 | 24.1994 | 26 | HN016 | HN049 |
| 363 | 21.0103 | 26 | HN016 | HN103 |  | 363 | 24.898 | 28 | HN016 | HN054 |
| 364 | 22.7363 | 26 | HN016 | HN105 |  | 364 | 18.8778 | 23 | HN016 | HN054 |
| 365 | 20.2039 | 23 | HN016 | HN107 |  | 365 | 25.9705 | 32 | HN016 | HN065 |
| 366 | 24.781 | 27 | HN016 | HN114 |  | 366 | 24.7261 | 26 | HN016 | HN072 |
| 367 | 24.9937 | 27 | HN016 | HN114 |  | 367 | 21.0015 | 26 | HN016 | HN079 |
| 368 | 27.6881 | 28 | HN016 | HN116 |  | 368 | 23.3102 | 25 | HN016 | HN082 |
| 369 | 23.0864 | 24 | HN016 | HN125 |  | 369 | 23.8082 | 25 | HN016 | HN087 |
| 370 | 23.4205 | 28 | HN016 | HN127 |  | 370 | 21.7743 | 24 | HN016 | HN091 |
| 371 | 23.8751 | 29 | HN016 | HN130 |  | 371 | 24.9711 | 23 | HN016 | HN093 |
| 372 | 25.1716 | 29 | HN016 | HN130 |  | 372 | 21.8588 | 24 | HN016 | HN095 |
| 373 | 25.0844 | 28 | HN016 | HN130 |  | 373 | 22.6026 | 29 | HN016 | HN096 |
| 374 | 25.9713 | 31 | HN016 | HN131 |  | 374 | 23.3904 | 25 | HN016 | HN101 |
| 375 | 18.1917 | 22 | HN016 | HN137 |  | 375 | 25.9616 | 30 | HN016 | HN103 |
| 376 | 23.6197 | 24 | HN016 | HN140 |  | 376 | 25.392 | 25 | HN016 | HN104 |
| 377 | 17.68 | 23 | HN016 | HN145 |  | 377 | 22.871 | 30 | HN016 | HN105 |
| 378 | 21.8016 | 25 | HN017 | HN020 |  | 378 | 22.2641 | 27 | HN016 | HN120 |
| 379 | 19.4798 | 27 | HN017 | HN024 |  | 379 | 23.0552 | 24 | HN016 | HN122 |
| 380 | 23.4955 | 28 | HN017 | HN026 |  | 380 | 22.0781 | 25 | HN016 | HN123 |
| 381 | 22.1107 | 24 | HN017 | HN035 |  | 381 | 21.9386 | 23 | HN016 | HN123 |
| 382 | 20.6353 | 24 | HN017 | HN036 |  | 382 | 26.0395 | 26 | HN016 | HN125 |
| 383 | 21.7803 | 28 | HN017 | HN037 |  | 383 | 20.0722 | 25 | HN016 | HN126 |
| 384 | 16.1501 | 25 | HN017 | HN042 |  | 384 | 21.2968 | 25 | HN016 | HN128 |
| 385 | 21.8888 | 27 | HN017 | HN045 |  | 385 | 23.8368 | 25 | HN016 | HN129 |
| 386 | 21.8232 | 26 | HN017 | HN051 |  | 386 | 24.5555 | 27 | HN016 | HN130 |
| 387 | 18.7143 | 20 | HN017 | HN058 |  | 387 | 24.3635 | 29 | HN016 | HN137 |
| 388 | 21.0433 | 26 | HN017 | HN060 |  | 388 | 22.0618 | 24 | HN016 | HN141 |
| 389 | 20.1856 | 22 | HN017 | HN066 |  | 389 | 22.4213 | 27 | HN016 | HN147 |
| 390 | 22.6838 | 26 | HN017 | HN066 |  | 390 | 22.4454 | 25 | HN016 | HN148 |
| 391 | 21.1191 | 27 | HN017 | HN067 |  | 391 | 23.2534 | 24 | HN016 | HN149 |
| 392 | 22.9155 | 26 | HN017 | HN068 |  | 392 | 25.0499 | 25 | HN017 | HN021 |
| 393 | 24.2211 | 29 | HN017 | HN072 |  | 393 | 23.4864 | 28 | HN017 | HN024 |
| 394 | 21.2244 | 27 | HN017 | HN075 |  | 394 | 21.5316 | 23 | HN017 | HN025 |
| 395 | 21.3746 | 25 | HN017 | HN075 |  | 395 | 22.2099 | 25 | HN017 | HN026 |
| 396 | 18.835 | 22 | HN017 | HN088 |  | 396 | 24.5958 | 29 | HN017 | HN034 |
| 397 | 24.0024 | 29 | HN017 | HN090 |  | 397 | 23.656 | 28 | HN017 | HN043 |
| 398 | 25.095 | 29 | HN017 | HN090 |  | 398 | 18.0866 | 24 | HN017 | HN044 |
| 399 | 17.1265 | 24 | HN017 | HN091 |  | 399 | 22.6037 | 27 | HN017 | HN045 |
| 400 | 22.459 | 26 | HN017 | HN093 |  | 400 | 22.5354 | 28 | HN017 | HN051 |
| 401 | 20.7868 | 28 | HN017 | HN109 |  | 401 | 23.5242 | 28 | HN017 | HN060 |
| 402 | 22.7534 | 28 | HN017 | HN110 |  | 402 | 24.962 | 29 | HN017 | HN068 |
| 403 | 23.7733 | 27 | HN017 | HN112 |  | 403 | 22.9299 | 26 | HN017 | HN068 |
| 404 | 22.4087 | 25 | HN017 | HN113 |  | 404 | 20.2297 | 27 | HN017 | HN075 |
| 405 | 17.8128 | 25 | HN017 | HN117 |  | 405 | 19.1361 | 25 | HN017 | HN076 |
| 406 | 16.7751 | 23 | HN017 | HN124 |  | 406 | 23.9765 | 26 | HN017 | HN080 |
| 407 | 25.1555 | 28 | HN017 | HN129 |  | 407 | 20.7334 | 26 | HN017 | HN082 |
| 408 | 23.0691 | 29 | HN017 | HN133 |  | 408 | 23.2175 | 29 | HN017 | HN083 |
| 409 | 21.9628 | 27 | HN017 | HN137 |  | 409 | 23.4318 | 30 | HN017 | HN094 |
| 410 | 20.5026 | 26 | HN017 | HN138 |  | 410 | 24.8728 | 22 | HN017 | HN097 |
| 411 | 23.0913 | 30 | HN017 | HN149 |  | 411 | 26.066 | 31 | HN017 | HN103 |
| 412 | 25.9433 | 25 | HN018 | HN026 |  | 412 | 22.4097 | 26 | HN017 | HN124 |
| 413 | 22.1521 | 23 | HN018 | HN032 |  | 413 | 25.5453 | 29 | HN017 | HN125 |
| 414 | 26.8198 | 28 | HN018 | HN034 |  | 414 | 20.7968 | 23 | HN017 | HN139 |
| 415 | 23.0735 | 22 | HN018 | HN035 |  | 415 | 20.5966 | 25 | HN017 | HN144 |
| 416 | 16.7547 | 19 | HN018 | HN036 |  | 416 | 25.6332 | 27 | HN018 | HN022 |
| 417 | 19.8086 | 23 | HN018 | HN054 |  | 417 | 24.0612 | 24 | HN018 | HN022 |
| 418 | 23.4311 | 26 | HN018 | HN061 |  | 418 | 25.5895 | 25 | HN018 | HN026 |
| 419 | 20.0434 | 24 | HN018 | HN065 |  | 419 | 20.7388 | 21 | HN018 | HN038 |
| 420 | 19.9366 | 21 | HN018 | HN076 |  | 420 | 21.6661 | 24 | HN018 | HN040 |
| 421 | 20.0007 | 22 | HN018 | HN082 |  | 421 | 22.7216 | 28 | HN018 | HN045 |
| 422 | 28.1928 | 28 | HN018 | HN082 |  | 422 | 22.5541 | 25 | HN018 | HN068 |
| 423 | 17.7027 | 20 | HN018 | HN088 |  | 423 | 25.0368 | 29 | HN018 | HN083 |
| 424 | 23.4119 | 29 | HN018 | HN094 |  | 424 | 27.8965 | 31 | HN018 | HN083 |
| 425 | 24.6752 | 25 | HN018 | HN094 |  | 425 | 23.4172 | 20 | HN018 | HN097 |
| 426 | 25.5233 | 28 | HN018 | HN094 |  | 426 | 22.7725 | 26 | HN018 | HN098 |
| 427 | 28.2556 | 31 | HN018 | HN096 |  | 427 | 23.3035 | 23 | HN018 | HN104 |
| 428 | 22.2536 | 24 | HN018 | HN105 |  | 428 | 25.3139 | 29 | HN018 | HN105 |
| 429 | 26.4227 | 25 | HN018 | HN113 |  | 429 | 25.3139 | 29 | HN018 | HN105 |
| 430 | 20.1641 | 22 | HN018 | HN123 |  | 430 | 23.7913 | 24 | HN018 | HN107 |
| 431 | 25.9586 | 26 | HN018 | HN129 |  | 431 | 21.5966 | 22 | HN018 | HN111 |
| 432 | 20.7853 | 23 | HN018 | HN136 |  | 432 | 21.4108 | 23 | HN018 | HN111 |
| 433 | 21.0177 | 24 | HN018 | HN136 |  | 433 | 27.6725 | 28 | HN018 | HN113 |
| 434 | 19.6766 | 22 | HN018 | HN143 |  | 434 | 27.6725 | 28 | HN018 | HN113 |
| 435 | 22.2237 | 22 | HN018 | HN144 |  | 435 | 19.5119 | 19 | HN018 | HN120 |
| 436 | 20.6865 | 23 | HN019 | HN020 |  | 436 | 19.5999 | 20 | HN018 | HN121 |
| 437 | 17.8439 | 22 | HN019 | HN070 |  | 437 | 19.8923 | 20 | HN018 | HN123 |
| 438 | 25.4848 | 27 | HN019 | HN082 |  | 438 | 24.4632 | 27 | HN018 | HN129 |
| 439 | 17.7024 | 19 | HN019 | HN088 |  | 439 | 26.6388 | 26 | HN018 | HN131 |
| 440 | 22.1544 | 26 | HN019 | HN143 |  | 440 | 22.2725 | 26 | HN018 | HN145 |
| 441 | 18.7664 | 18 | HN019 | HN148 |  | 441 | 20.5956 | 24 | HN019 | HN024 |
| 442 | 26.1928 | 26 | HN020 | HN021 |  | 442 | 20.1336 | 22 | HN019 | HN025 |
| 443 | 25.3128 | 24 | HN020 | HN022 |  | 443 | 23.795 | 22 | HN019 | HN026 |
| 444 | 25.0651 | 22 | HN020 | HN022 |  | 444 | 25.1337 | 28 | HN019 | HN043 |
| 445 | 25.1808 | 25 | HN020 | HN022 |  | 445 | 21.5218 | 25 | HN019 | HN060 |
| 446 | 23.6836 | 23 | HN020 | HN025 |  | 446 | 21.3743 | 24 | HN019 | HN068 |
| 447 | 22.3671 | 21 | HN020 | HN027 |  | 447 | 24.9818 | 26 | HN019 | HN073 |
| 448 | 23.7642 | 24 | HN020 | HN027 |  | 448 | 21.6553 | 27 | HN019 | HN074 |
| 449 | 24.7454 | 24 | HN020 | HN035 |  | 449 | 20.4102 | 29 | HN019 | HN083 |
| 450 | 24.7572 | 28 | HN020 | HN047 |  | 450 | 20.3943 | 21 | HN019 | HN087 |
| 451 | 23.5298 | 26 | HN020 | HN051 |  | 451 | 20.087 | 26 | HN019 | HN094 |
| 452 | 16.5774 | 22 | HN020 | HN053 |  | 452 | 19.3921 | 25 | HN019 | HN109 |
| 453 | 22.1405 | 25 | HN020 | HN054 |  | 453 | 24.4007 | 29 | HN019 | HN131 |
| 454 | 20.9177 | 23 | HN020 | HN061 |  | 454 | 20.1689 | 21 | HN019 | HN132 |
| 455 | 22.3422 | 24 | HN020 | HN064 |  | 455 | 22.2028 | 27 | HN019 | HN138 |
| 456 | 26.681 | 28 | HN020 | HN065 |  | 456 | 20.4366 | 23 | HN019 | HN140 |
| 457 | 24.2789 | 26 | HN020 | HN072 |  | 457 | 25.7686 | 27 | HN020 | HN021 |
| 458 | 25.3683 | 29 | HN020 | HN072 |  | 458 | 26.0489 | 25 | HN020 | HN021 |
| 459 | 27.1331 | 30 | HN020 | HN073 |  | 459 | 25.5761 | 26 | HN020 | HN022 |
| 460 | 24.7136 | 28 | HN020 | HN082 |  | 460 | 23.7495 | 23 | HN020 | HN027 |
| 461 | 15.4372 | 21 | HN020 | HN087 |  | 461 | 22.0349 | 25 | HN020 | HN033 |
| 462 | 21.3147 | 25 | HN020 | HN090 |  | 462 | 24.1351 | 27 | HN020 | HN035 |
| 463 | 24.5618 | 27 | HN020 | HN093 |  | 463 | 22.6106 | 26 | HN020 | HN035 |
| 464 | 25.8077 | 28 | HN020 | HN096 |  | 464 | 22.0848 | 26 | HN020 | HN037 |
| 465 | 27.2573 | 23 | HN020 | HN097 |  | 465 | 22.2988 | 26 | HN020 | HN046 |
| 466 | 20.357 | 23 | HN020 | HN100 |  | 466 | 24.4126 | 26 | HN020 | HN051 |
| 467 | 22.5257 | 22 | HN020 | HN100 |  | 467 | 22.0528 | 25 | HN020 | HN054 |
| 468 | 18.1543 | 20 | HN020 | HN100 |  | 468 | 22.0421 | 25 | HN020 | HN063 |
| 469 | 19.5218 | 23 | HN020 | HN102 |  | 469 | 21.3887 | 19 | HN020 | HN066 |
| 470 | 25.6459 | 26 | HN020 | HN104 |  | 470 | 24.6419 | 25 | HN020 | HN066 |
| 471 | 22.4425 | 27 | HN020 | HN105 |  | 471 | 25.3139 | 26 | HN020 | HN071 |
| 472 | 21.5359 | 25 | HN020 | HN108 |  | 472 | 20.3423 | 25 | HN020 | HN075 |
| 473 | 21.1678 | 24 | HN020 | HN110 |  | 473 | 21.8589 | 24 | HN020 | HN085 |
| 474 | 24.9742 | 26 | HN020 | HN111 |  | 474 | 25.209 | 28 | HN020 | HN093 |
| 475 | 25.0975 | 26 | HN020 | HN113 |  | 475 | 20.9299 | 25 | HN020 | HN093 |
| 476 | 25.7278 | 26 | HN020 | HN116 |  | 476 | 23.2357 | 24 | HN020 | HN097 |
| 477 | 25.8632 | 27 | HN020 | HN116 |  | 477 | 22.7159 | 21 | HN020 | HN097 |
| 478 | 26.2236 | 27 | HN020 | HN116 |  | 478 | 25.7858 | 27 | HN020 | HN098 |
| 479 | 24.5864 | 25 | HN020 | HN118 |  | 479 | 24.2735 | 28 | HN020 | HN098 |
| 480 | 16.3273 | 20 | HN020 | HN123 |  | 480 | 24.1152 | 27 | HN020 | HN102 |
| 481 | 20.9927 | 26 | HN020 | HN123 |  | 481 | 28.2899 | 32 | HN020 | HN103 |
| 482 | 22.7446 | 26 | HN020 | HN127 |  | 482 | 26.665 | 30 | HN020 | HN103 |
| 483 | 21.2291 | 24 | HN020 | HN129 |  | 483 | 26.665 | 30 | HN020 | HN103 |
| 484 | 25.047 | 30 | HN020 | HN129 |  | 484 | 24.5335 | 24 | HN020 | HN104 |
| 485 | 22.7354 | 25 | HN020 | HN133 |  | 485 | 21.5735 | 22 | HN020 | HN110 |
| 486 | 23.3905 | 24 | HN020 | HN133 |  | 486 | 27.0228 | 25 | HN020 | HN113 |
| 487 | 23.8835 | 25 | HN020 | HN137 |  | 487 | 24.9825 | 26 | HN020 | HN114 |
| 488 | 22.7258 | 26 | HN020 | HN140 |  | 488 | 22.1773 | 25 | HN020 | HN114 |
| 489 | 22.2979 | 24 | HN020 | HN141 |  | 489 | 27.6568 | 28 | HN020 | HN116 |
| 490 | 24.7609 | 22 | HN020 | HN141 |  | 490 | 25.6457 | 25 | HN020 | HN116 |
| 491 | 21.3642 | 19 | HN020 | HN142 |  | 491 | 22.2957 | 22 | HN020 | HN117 |
| 492 | 17.6221 | 22 | HN020 | HN146 |  | 492 | 22.8169 | 24 | HN020 | HN118 |
| 493 | 21.7232 | 23 | HN020 | HN148 |  | 493 | 21.7015 | 25 | HN020 | HN120 |
| 494 | 19.706 | 23 | HN020 | HN150 |  | 494 | 23.4068 | 23 | HN020 | HN120 |
| 495 | 24.6021 | 24 | HN021 | HN022 |  | 495 | 24.3871 | 27 | HN020 | HN123 |
| 496 | 19.4713 | 20 | HN021 | HN025 |  | 496 | 23.6181 | 26 | HN020 | HN124 |
| 497 | 20.8666 | 18 | HN021 | HN026 |  | 497 | 23.3462 | 24 | HN020 | HN126 |
| 498 | 25.8033 | 28 | HN021 | HN034 |  | 498 | 21.1574 | 22 | HN020 | HN129 |
| 499 | 22.5906 | 25 | HN021 | HN050 |  | 499 | 22.6126 | 26 | HN020 | HN134 |
| 500 | 22.405 | 27 | HN021 | HN050 |  | 500 | 22.2182 | 22 | HN020 | HN142 |
| 501 | 25.0851 | 25 | HN021 | HN050 |  | 501 | 23.6586 | 27 | HN020 | HN146 |
| 502 | 24.2172 | 23 | HN021 | HN050 |  | 502 | 24.901 | 26 | HN020 | HN149 |
| 503 | 21.8317 | 24 | HN021 | HN050 |  | 503 | 25.1651 | 24 | HN021 | HN026 |
| 504 | 19.8615 | 23 | HN021 | HN057 |  | 504 | 24.7635 | 26 | HN021 | HN034 |
| 505 | 18.9519 | 26 | HN021 | HN063 |  | 505 | 21.5947 | 22 | HN021 | HN035 |
| 506 | 28.2252 | 28 | HN021 | HN064 |  | 506 | 21.2746 | 23 | HN021 | HN037 |
| 507 | 20.9155 | 23 | HN021 | HN065 |  | 507 | 20.9803 | 25 | HN021 | HN040 |
| 508 | 27.9614 | 25 | HN021 | HN066 |  | 508 | 19.4767 | 24 | HN021 | HN040 |
| 509 | 22.7514 | 25 | HN021 | HN069 |  | 509 | 22.7785 | 26 | HN021 | HN045 |
| 510 | 25.4375 | 26 | HN021 | HN073 |  | 510 | 22.7785 | 26 | HN021 | HN045 |
| 511 | 20.16 | 21 | HN021 | HN080 |  | 511 | 24.787 | 24 | HN021 | HN048 |
| 512 | 26.5049 | 24 | HN021 | HN082 |  | 512 | 15.1795 | 20 | HN021 | HN055 |
| 513 | 21.9083 | 27 | HN021 | HN083 |  | 513 | 24.8997 | 27 | HN021 | HN057 |
| 514 | 24.255 | 31 | HN021 | HN083 |  | 514 | 26.0339 | 27 | HN021 | HN058 |
| 515 | 25.4618 | 30 | HN021 | HN096 |  | 515 | 18.6812 | 22 | HN021 | HN059 |
| 516 | 24.0643 | 30 | HN021 | HN096 |  | 516 | 21.6166 | 27 | HN021 | HN060 |
| 517 | 23.7342 | 24 | HN021 | HN098 |  | 517 | 23.4531 | 27 | HN021 | HN060 |
| 518 | 22.8656 | 30 | HN021 | HN103 |  | 518 | 24.0544 | 25 | HN021 | HN072 |
| 519 | 19.7474 | 20 | HN021 | HN105 |  | 519 | 26.9239 | 30 | HN021 | HN073 |
| 520 | 25.9785 | 25 | HN021 | HN107 |  | 520 | 24.2997 | 25 | HN021 | HN075 |
| 521 | 22.2029 | 24 | HN021 | HN110 |  | 521 | 22.1443 | 26 | HN021 | HN079 |
| 522 | 22.2021 | 25 | HN021 | HN111 |  | 522 | 21.2575 | 21 | HN021 | HN080 |
| 523 | 18.75 | 22 | HN021 | HN111 |  | 523 | 22.9094 | 26 | HN021 | HN082 |
| 524 | 24.2157 | 24 | HN021 | HN113 |  | 524 | 25.0719 | 25 | HN021 | HN083 |
| 525 | 22.0255 | 20 | HN021 | HN113 |  | 525 | 24.4429 | 25 | HN021 | HN083 |
| 526 | 25.6869 | 25 | HN021 | HN118 |  | 526 | 23.4782 | 24 | HN021 | HN090 |
| 527 | 22.133 | 26 | HN021 | HN122 |  | 527 | 27.0121 | 24 | HN021 | HN090 |
| 528 | 22.551 | 24 | HN021 | HN124 |  | 528 | 23.4806 | 27 | HN021 | HN103 |
| 529 | 24.15 | 27 | HN021 | HN131 |  | 529 | 26.1107 | 27 | HN021 | HN107 |
| 530 | 25.8311 | 27 | HN022 | HN024 |  | 530 | 19.3823 | 20 | HN021 | HN114 |
| 531 | 25.9402 | 27 | HN022 | HN024 |  | 531 | 24.1302 | 24 | HN021 | HN118 |
| 532 | 25.0566 | 26 | HN022 | HN026 |  | 532 | 20.752 | 20 | HN021 | HN121 |
| 533 | 24.0611 | 26 | HN022 | HN026 |  | 533 | 23.9866 | 26 | HN021 | HN123 |
| 534 | 24.5293 | 22 | HN022 | HN026 |  | 534 | 23.2304 | 28 | HN021 | HN125 |
| 535 | 25.2947 | 24 | HN022 | HN030 |  | 535 | 18.2624 | 20 | HN021 | HN128 |
| 536 | 26.6952 | 26 | HN022 | HN032 |  | 536 | 22.5285 | 25 | HN021 | HN131 |
| 537 | 27.5576 | 30 | HN022 | HN034 |  | 537 | 27.3307 | 28 | HN021 | HN143 |
| 538 | 25.7155 | 24 | HN022 | HN040 |  | 538 | 26.072 | 26 | HN021 | HN148 |
| 539 | 28.3483 | 26 | HN022 | HN043 |  | 539 | 24.4008 | 21 | HN022 | HN025 |
| 540 | 24.2314 | 25 | HN022 | HN044 |  | 540 | 24.3372 | 25 | HN022 | HN026 |
| 541 | 22.6106 | 25 | HN022 | HN046 |  | 541 | 24.842 | 25 | HN022 | HN031 |
| 542 | 21.4137 | 24 | HN022 | HN050 |  | 542 | 25.2107 | 24 | HN022 | HN038 |
| 543 | 25.3917 | 29 | HN022 | HN051 |  | 543 | 22.4739 | 21 | HN022 | HN040 |
| 544 | 26.8614 | 27 | HN022 | HN054 |  | 544 | 27.1904 | 23 | HN022 | HN043 |
| 545 | 25.2422 | 29 | HN022 | HN055 |  | 545 | 24.5256 | 24 | HN022 | HN043 |
| 546 | 24.9695 | 28 | HN022 | HN057 |  | 546 | 28.1211 | 29 | HN022 | HN046 |
| 547 | 21.4071 | 21 | HN022 | HN059 |  | 547 | 27.7158 | 28 | HN022 | HN046 |
| 548 | 27.4132 | 30 | HN022 | HN060 |  | 548 | 23.5856 | 23 | HN022 | HN048 |
| 549 | 26.4512 | 28 | HN022 | HN060 |  | 549 | 23.582 | 24 | HN022 | HN049 |
| 550 | 24.535 | 29 | HN022 | HN060 |  | 550 | 26.8633 | 31 | HN022 | HN050 |
| 551 | 23.6523 | 23 | HN022 | HN061 |  | 551 | 25.076 | 27 | HN022 | HN052 |
| 552 | 24.6745 | 23 | HN022 | HN062 |  | 552 | 24.7958 | 25 | HN022 | HN052 |
| 553 | 25.0216 | 27 | HN022 | HN063 |  | 553 | 24.4723 | 27 | HN022 | HN059 |
| 554 | 27.0346 | 27 | HN022 | HN065 |  | 554 | 28.7497 | 27 | HN022 | HN065 |
| 555 | 23.8095 | 23 | HN022 | HN067 |  | 555 | 25.5562 | 27 | HN022 | HN067 |
| 556 | 25.3329 | 29 | HN022 | HN068 |  | 556 | 26.9898 | 27 | HN022 | HN071 |
| 557 | 24.2018 | 24 | HN022 | HN071 |  | 557 | 25.5974 | 24 | HN022 | HN074 |
| 558 | 25.0077 | 23 | HN022 | HN073 |  | 558 | 26.1667 | 26 | HN022 | HN074 |
| 559 | 23.0199 | 24 | HN022 | HN074 |  | 559 | 23.8481 | 26 | HN022 | HN075 |
| 560 | 21.881 | 25 | HN022 | HN075 |  | 560 | 24.0211 | 23 | HN022 | HN076 |
| 561 | 24.7995 | 24 | HN022 | HN076 |  | 561 | 25.5793 | 24 | HN022 | HN076 |
| 562 | 24.6616 | 22 | HN022 | HN077 |  | 562 | 27.1116 | 34 | HN022 | HN083 |
| 563 | 24.6227 | 23 | HN022 | HN077 |  | 563 | 22.5877 | 23 | HN022 | HN085 |
| 564 | 25.4731 | 30 | HN022 | HN079 |  | 564 | 28.5838 | 30 | HN022 | HN090 |
| 565 | 22.7983 | 20 | HN022 | HN080 |  | 565 | 26.0164 | 27 | HN022 | HN091 |
| 566 | 26.8014 | 27 | HN022 | HN082 |  | 566 | 26.2551 | 26 | HN022 | HN094 |
| 567 | 26.1127 | 24 | HN022 | HN083 |  | 567 | 26.3935 | 27 | HN022 | HN094 |
| 568 | 29.4517 | 34 | HN022 | HN083 |  | 568 | 24.7323 | 23 | HN022 | HN095 |
| 569 | 23.122 | 22 | HN022 | HN085 |  | 569 | 25.7001 | 23 | HN022 | HN096 |
| 570 | 24.8225 | 26 | HN022 | HN085 |  | 570 | 28.3967 | 29 | HN022 | HN098 |
| 571 | 24.5755 | 26 | HN022 | HN091 |  | 571 | 24.5697 | 25 | HN022 | HN102 |
| 572 | 21.2103 | 22 | HN022 | HN092 |  | 572 | 27.4363 | 31 | HN022 | HN103 |
| 573 | 26.4833 | 26 | HN022 | HN093 |  | 573 | 27.6865 | 23 | HN022 | HN104 |
| 574 | 23.9473 | 28 | HN022 | HN094 |  | 574 | 27.8197 | 29 | HN022 | HN105 |
| 575 | 25.5065 | 28 | HN022 | HN096 |  | 575 | 22.7988 | 20 | HN022 | HN106 |
| 576 | 25.7427 | 27 | HN022 | HN096 |  | 576 | 24.0227 | 28 | HN022 | HN109 |
| 577 | 25.5949 | 30 | HN022 | HN098 |  | 577 | 27.3853 | 30 | HN022 | HN109 |
| 578 | 24.9721 | 23 | HN022 | HN100 |  | 578 | 28.2738 | 29 | HN022 | HN112 |
| 579 | 23.1986 | 22 | HN022 | HN100 |  | 579 | 26.5001 | 27 | HN022 | HN113 |
| 580 | 27.357 | 26 | HN022 | HN103 |  | 580 | 25.0571 | 27 | HN022 | HN113 |
| 581 | 25.9334 | 27 | HN022 | HN109 |  | 581 | 29.7542 | 28 | HN022 | HN116 |
| 582 | 21.0165 | 20 | HN022 | HN110 |  | 582 | 23.9592 | 23 | HN022 | HN118 |
| 583 | 26.4518 | 26 | HN022 | HN111 |  | 583 | 24.9444 | 25 | HN022 | HN120 |
| 584 | 22.5845 | 25 | HN022 | HN116 |  | 584 | 21.3308 | 25 | HN022 | HN122 |
| 585 | 26.8683 | 26 | HN022 | HN117 |  | 585 | 20.973 | 23 | HN022 | HN122 |
| 586 | 26.9918 | 23 | HN022 | HN117 |  | 586 | 27.6695 | 28 | HN022 | HN125 |
| 587 | 26.2473 | 24 | HN022 | HN118 |  | 587 | 28.161 | 29 | HN022 | HN125 |
| 588 | 22.8375 | 23 | HN022 | HN118 |  | 588 | 27.4818 | 30 | HN022 | HN129 |
| 589 | 24.3532 | 26 | HN022 | HN122 |  | 589 | 26.5944 | 26 | HN022 | HN131 |
| 590 | 24.7586 | 22 | HN022 | HN122 |  | 590 | 26.5944 | 26 | HN022 | HN131 |
| 591 | 22.4983 | 23 | HN022 | HN123 |  | 591 | 25.0203 | 24 | HN022 | HN137 |
| 592 | 23.7695 | 24 | HN022 | HN126 |  | 592 | 21.5758 | 21 | HN022 | HN137 |
| 593 | 26.8558 | 28 | HN022 | HN127 |  | 593 | 27.0417 | 27 | HN022 | HN137 |
| 594 | 26.2858 | 29 | HN022 | HN127 |  | 594 | 18.7393 | 25 | HN022 | HN139 |
| 595 | 26.0871 | 26 | HN022 | HN128 |  | 595 | 23.6908 | 22 | HN022 | HN141 |
| 596 | 26.4365 | 26 | HN022 | HN131 |  | 596 | 18.7305 | 22 | HN023 | HN025 |
| 597 | 23.9476 | 25 | HN022 | HN133 |  | 597 | 18.2865 | 21 | HN023 | HN030 |
| 598 | 21.1691 | 24 | HN022 | HN135 |  | 598 | 21.1816 | 25 | HN023 | HN034 |
| 599 | 23.7521 | 24 | HN022 | HN135 |  | 599 | 21.8478 | 23 | HN023 | HN043 |
| 600 | 27.2332 | 29 | HN022 | HN137 |  | 600 | 18.3177 | 24 | HN023 | HN045 |
| 601 | 18.8438 | 22 | HN022 | HN137 |  | 601 | 19.1606 | 24 | HN023 | HN050 |
| 602 | 23.0072 | 24 | HN022 | HN138 |  | 602 | 18.7127 | 23 | HN023 | HN062 |
| 603 | 20.9587 | 24 | HN022 | HN144 |  | 603 | 24.2481 | 27 | HN023 | HN073 |
| 604 | 23.6776 | 23 | HN022 | HN148 |  | 604 | 21.299 | 27 | HN023 | HN083 |
| 605 | 23.2134 | 28 | HN023 | HN094 |  | 605 | 18.4763 | 23 | HN023 | HN085 |
| 606 | 17.5166 | 23 | HN023 | HN102 |  | 606 | 15.6354 | 20 | HN023 | HN085 |
| 607 | 20.663 | 22 | HN023 | HN104 |  | 607 | 23.0252 | 28 | HN023 | HN094 |
| 608 | 17.2124 | 23 | HN023 | HN107 |  | 608 | 20.0348 | 25 | HN023 | HN105 |
| 609 | 17.7894 | 22 | HN023 | HN111 |  | 609 | 20.948 | 22 | HN023 | HN113 |
| 610 | 19.9002 | 21 | HN023 | HN113 |  | 610 | 20.7307 | 25 | HN023 | HN115 |
| 611 | 20.9032 | 24 | HN023 | HN118 |  | 611 | 22.4205 | 26 | HN023 | HN116 |
| 612 | 21.7801 | 27 | HN023 | HN130 |  | 612 | 17.802 | 25 | HN023 | HN119 |
| 613 | 20.914 | 24 | HN023 | HN138 |  | 613 | 23.0104 | 24 | HN023 | HN129 |
| 614 | 21.3158 | 27 | HN024 | HN029 |  | 614 | 16.7025 | 21 | HN023 | HN135 |
| 615 | 19.777 | 25 | HN024 | HN033 |  | 615 | 17.877 | 20 | HN023 | HN140 |
| 616 | 24.7488 | 29 | HN024 | HN034 |  | 616 | 19.0392 | 24 | HN024 | HN041 |
| 617 | 22.749 | 29 | HN024 | HN043 |  | 617 | 19.7889 | 25 | HN024 | HN041 |
| 618 | 21.131 | 26 | HN024 | HN060 |  | 618 | 20.9564 | 27 | HN024 | HN041 |
| 619 | 25.4606 | 28 | HN024 | HN073 |  | 619 | 23.4683 | 27 | HN024 | HN057 |
| 620 | 20.9651 | 25 | HN024 | HN076 |  | 620 | 20.5085 | 26 | HN024 | HN070 |
| 621 | 24.8876 | 30 | HN024 | HN083 |  | 621 | 20.7146 | 29 | HN024 | HN072 |
| 622 | 22.0544 | 26 | HN024 | HN087 |  | 622 | 22.9377 | 26 | HN024 | HN075 |
| 623 | 18.1469 | 22 | HN024 | HN088 |  | 623 | 17.985 | 22 | HN024 | HN076 |
| 624 | 20.6231 | 26 | HN024 | HN102 |  | 624 | 27.7255 | 29 | HN024 | HN082 |
| 625 | 21.5904 | 25 | HN024 | HN104 |  | 625 | 23.9374 | 26 | HN024 | HN082 |
| 626 | 24.3825 | 26 | HN024 | HN113 |  | 626 | 23.0985 | 28 | HN024 | HN118 |
| 627 | 21.5468 | 27 | HN024 | HN125 |  | 627 | 23.3879 | 29 | HN024 | HN118 |
| 628 | 19.6367 | 26 | HN024 | HN129 |  | 628 | 19.7066 | 26 | HN024 | HN124 |
| 629 | 25.1603 | 28 | HN024 | HN129 |  | 629 | 19.4691 | 26 | HN024 | HN126 |
| 630 | 25.1603 | 28 | HN024 | HN129 |  | 630 | 19.7081 | 27 | HN024 | HN126 |
| 631 | 21.2602 | 26 | HN024 | HN134 |  | 631 | 25.725 | 30 | HN024 | HN131 |
| 632 | 20.3591 | 28 | HN024 | HN138 |  | 632 | 23.4892 | 26 | HN024 | HN132 |
| 633 | 19.9782 | 26 | HN024 | HN147 |  | 633 | 21.0949 | 25 | HN024 | HN137 |
| 634 | 21.1622 | 27 | HN024 | HN147 |  | 634 | 23.8376 | 26 | HN024 | HN138 |
| 635 | 20.2664 | 25 | HN024 | HN148 |  | 635 | 18.7714 | 23 | HN024 | HN147 |
| 636 | 18.3424 | 20 | HN025 | HN049 |  | 636 | 21.305 | 28 | HN024 | HN147 |
| 637 | 23.3706 | 26 | HN025 | HN050 |  | 637 | 20.3322 | 25 | HN024 | HN148 |
| 638 | 19.3911 | 21 | HN025 | HN054 |  | 638 | 20.6774 | 23 | HN025 | HN026 |
| 639 | 19.9093 | 23 | HN025 | HN060 |  | 639 | 22.578 | 24 | HN025 | HN034 |
| 640 | 23.8605 | 24 | HN025 | HN067 |  | 640 | 19.2225 | 25 | HN025 | HN035 |
| 641 | 24.3581 | 27 | HN025 | HN083 |  | 641 | 17.8893 | 20 | HN025 | HN036 |
| 642 | 22.9403 | 27 | HN025 | HN094 |  | 642 | 22.2957 | 21 | HN025 | HN043 |
| 643 | 20.1913 | 24 | HN025 | HN096 |  | 643 | 25.0587 | 26 | HN025 | HN082 |
| 644 | 22.8408 | 21 | HN025 | HN097 |  | 644 | 23.1507 | 25 | HN025 | HN094 |
| 645 | 18.543 | 21 | HN025 | HN111 |  | 645 | 20.9178 | 28 | HN025 | HN103 |
| 646 | 22.4212 | 26 | HN025 | HN130 |  | 646 | 23.2202 | 30 | HN025 | HN105 |
| 647 | 22.6607 | 28 | HN025 | HN131 |  | 647 | 22.5225 | 22 | HN025 | HN113 |
| 648 | 19.3622 | 19 | HN025 | HN148 |  | 648 | 23.7017 | 24 | HN025 | HN118 |
| 649 | 25.7637 | 27 | HN026 | HN037 |  | 649 | 23.4146 | 27 | HN025 | HN127 |
| 650 | 26.415 | 30 | HN026 | HN037 |  | 650 | 18.1644 | 19 | HN025 | HN128 |
| 651 | 26.415 | 30 | HN026 | HN037 |  | 651 | 21.0582 | 22 | HN025 | HN140 |
| 652 | 24.6515 | 23 | HN026 | HN039 |  | 652 | 21.6289 | 24 | HN025 | HN143 |
| 653 | 19.1626 | 23 | HN026 | HN040 |  | 653 | 21.2506 | 19 | HN026 | HN029 |
| 654 | 22.9617 | 25 | HN026 | HN041 |  | 654 | 23.276 | 22 | HN026 | HN030 |
| 655 | 25.7281 | 25 | HN026 | HN043 |  | 655 | 21.3001 | 22 | HN026 | HN030 |
| 656 | 20.9588 | 26 | HN026 | HN046 |  | 656 | 22.5246 | 23 | HN026 | HN033 |
| 657 | 25.0753 | 29 | HN026 | HN055 |  | 657 | 26.096 | 28 | HN026 | HN034 |
| 658 | 20.3601 | 22 | HN026 | HN056 |  | 658 | 25.7995 | 25 | HN026 | HN043 |
| 659 | 20.8674 | 22 | HN026 | HN057 |  | 659 | 24.6249 | 24 | HN026 | HN044 |
| 660 | 22.5633 | 25 | HN026 | HN057 |  | 660 | 18.9338 | 25 | HN026 | HN044 |
| 661 | 23.7805 | 27 | HN026 | HN058 |  | 661 | 24.7738 | 26 | HN026 | HN045 |
| 662 | 24.8499 | 27 | HN026 | HN059 |  | 662 | 23.8406 | 25 | HN026 | HN047 |
| 663 | 23.5399 | 24 | HN026 | HN060 |  | 663 | 25.6903 | 28 | HN026 | HN050 |
| 664 | 24.6354 | 28 | HN026 | HN063 |  | 664 | 23.2003 | 23 | HN026 | HN050 |
| 665 | 22.5671 | 24 | HN026 | HN064 |  | 665 | 24.8313 | 29 | HN026 | HN051 |
| 666 | 18.3647 | 19 | HN026 | HN066 |  | 666 | 23.8293 | 27 | HN026 | HN051 |
| 667 | 27.2318 | 28 | HN026 | HN068 |  | 667 | 25.1411 | 27 | HN026 | HN051 |
| 668 | 23.7788 | 27 | HN026 | HN072 |  | 668 | 25.2385 | 27 | HN026 | HN052 |
| 669 | 26.0905 | 27 | HN026 | HN072 |  | 669 | 23.2444 | 25 | HN026 | HN052 |
| 670 | 28.3493 | 30 | HN026 | HN073 |  | 670 | 21.8794 | 25 | HN026 | HN059 |
| 671 | 27.5498 | 30 | HN026 | HN082 |  | 671 | 24.7653 | 26 | HN026 | HN061 |
| 672 | 25.5413 | 31 | HN026 | HN083 |  | 672 | 23.4825 | 24 | HN026 | HN062 |
| 673 | 18.4204 | 17 | HN026 | HN092 |  | 673 | 21.9781 | 25 | HN026 | HN062 |
| 674 | 20.7079 | 20 | HN026 | HN093 |  | 674 | 23.7383 | 24 | HN026 | HN064 |
| 675 | 23.9322 | 25 | HN026 | HN094 |  | 675 | 27.8396 | 29 | HN026 | HN065 |
| 676 | 25.8432 | 29 | HN026 | HN094 |  | 676 | 22.4293 | 24 | HN026 | HN070 |
| 677 | 24.5923 | 19 | HN026 | HN097 |  | 677 | 23.8717 | 24 | HN026 | HN071 |
| 678 | 24.8552 | 26 | HN026 | HN102 |  | 678 | 25.0529 | 27 | HN026 | HN073 |
| 679 | 26.7431 | 27 | HN026 | HN103 |  | 679 | 21.3713 | 25 | HN026 | HN079 |
| 680 | 23.7749 | 20 | HN026 | HN106 |  | 680 | 25.6796 | 28 | HN026 | HN082 |
| 681 | 22.0904 | 19 | HN026 | HN106 |  | 681 | 25.7138 | 24 | HN026 | HN084 |
| 682 | 26.959 | 26 | HN026 | HN111 |  | 682 | 25.4897 | 26 | HN026 | HN087 |
| 683 | 23.8692 | 24 | HN026 | HN113 |  | 683 | 21.4487 | 23 | HN026 | HN088 |
| 684 | 26.8232 | 26 | HN026 | HN113 |  | 684 | 26.2223 | 28 | HN026 | HN090 |
| 685 | 24.2264 | 25 | HN026 | HN113 |  | 685 | 20.2119 | 22 | HN026 | HN091 |
| 686 | 20.255 | 24 | HN026 | HN115 |  | 686 | 25.9241 | 29 | HN026 | HN094 |
| 687 | 26.728 | 26 | HN026 | HN116 |  | 687 | 25.464 | 21 | HN026 | HN097 |
| 688 | 29.5722 | 28 | HN026 | HN116 |  | 688 | 27.2001 | 22 | HN026 | HN104 |
| 689 | 22.8262 | 24 | HN026 | HN116 |  | 689 | 25.0732 | 22 | HN026 | HN104 |
| 690 | 23.991 | 25 | HN026 | HN118 |  | 690 | 23.6574 | 24 | HN026 | HN107 |
| 691 | 25.8785 | 30 | HN026 | HN130 |  | 691 | 25.1275 | 27 | HN026 | HN108 |
| 692 | 25.9066 | 29 | HN026 | HN130 |  | 692 | 25.082 | 25 | HN026 | HN109 |
| 693 | 22.8508 | 24 | HN026 | HN132 |  | 693 | 24.6483 | 25 | HN026 | HN116 |
| 694 | 20.4613 | 22 | HN026 | HN134 |  | 694 | 26.152 | 27 | HN026 | HN118 |
| 695 | 24.586 | 25 | HN026 | HN140 |  | 695 | 23.8742 | 25 | HN026 | HN119 |
| 696 | 23.4328 | 24 | HN026 | HN142 |  | 696 | 20.4437 | 24 | HN026 | HN121 |
| 697 | 23.6574 | 24 | HN026 | HN143 |  | 697 | 22.5773 | 23 | HN026 | HN122 |
| 698 | 19.028 | 22 | HN026 | HN144 |  | 698 | 21.9617 | 24 | HN026 | HN123 |
| 699 | 24.7896 | 24 | HN026 | HN144 |  | 699 | 23.4573 | 24 | HN026 | HN123 |
| 700 | 23.4901 | 23 | HN026 | HN148 |  | 700 | 26.6181 | 27 | HN026 | HN125 |
| 701 | 23.3711 | 22 | HN026 | HN148 |  | 701 | 26.3617 | 28 | HN026 | HN127 |
| 702 | 25.0514 | 26 | HN026 | HN149 |  | 702 | 25.0329 | 27 | HN026 | HN130 |
| 703 | 22.2131 | 21 | HN026 | HN150 |  | 703 | 24.5302 | 25 | HN026 | HN132 |
| 704 | 22.8881 | 24 | HN027 | HN034 |  | 704 | 21.8514 | 23 | HN026 | HN133 |
| 705 | 22.4629 | 25 | HN027 | HN037 |  | 705 | 21.455 | 22 | HN026 | HN140 |
| 706 | 23.438 | 24 | HN027 | HN037 |  | 706 | 22.7852 | 22 | HN026 | HN140 |
| 707 | 19.5322 | 21 | HN027 | HN047 |  | 707 | 25.7654 | 26 | HN026 | HN141 |
| 708 | 19.8338 | 21 | HN027 | HN061 |  | 708 | 24.1354 | 26 | HN026 | HN146 |
| 709 | 25.3422 | 26 | HN027 | HN068 |  | 709 | 23.74 | 26 | HN026 | HN147 |
| 710 | 24.918 | 26 | HN027 | HN068 |  | 710 | 20.2096 | 22 | HN027 | HN033 |
| 711 | 23.3158 | 25 | HN027 | HN070 |  | 711 | 19.4014 | 18 | HN027 | HN035 |
| 712 | 22.4153 | 22 | HN027 | HN090 |  | 712 | 20.5932 | 20 | HN027 | HN036 |
| 713 | 24.3659 | 24 | HN027 | HN103 |  | 713 | 19.7733 | 25 | HN027 | HN054 |
| 714 | 25.2106 | 26 | HN027 | HN103 |  | 714 | 22.3379 | 21 | HN027 | HN058 |
| 715 | 22.0496 | 26 | HN027 | HN105 |  | 715 | 20.1781 | 23 | HN027 | HN063 |
| 716 | 27.2325 | 25 | HN027 | HN111 |  | 716 | 23.6475 | 28 | HN027 | HN065 |
| 717 | 23.6821 | 25 | HN027 | HN111 |  | 717 | 22.9426 | 22 | HN027 | HN082 |
| 718 | 22.2789 | 21 | HN027 | HN122 |  | 718 | 22.2945 | 26 | HN027 | HN083 |
| 719 | 19.811 | 23 | HN027 | HN125 |  | 719 | 21.2125 | 18 | HN027 | HN084 |
| 720 | 22.6762 | 25 | HN027 | HN130 |  | 720 | 22.3391 | 26 | HN027 | HN094 |
| 721 | 22.5532 | 25 | HN027 | HN131 |  | 721 | 18.3642 | 22 | HN027 | HN095 |
| 722 | 24.9149 | 27 | HN027 | HN131 |  | 722 | 22.2119 | 22 | HN027 | HN101 |
| 723 | 24.4499 | 28 | HN027 | HN137 |  | 723 | 21.9696 | 21 | HN027 | HN109 |
| 724 | 22.8198 | 21 | HN027 | HN149 |  | 724 | 20.1841 | 22 | HN027 | HN109 |
| 725 | 17.5373 | 20 | HN029 | HN036 |  | 725 | 25.6849 | 26 | HN027 | HN116 |
| 726 | 17.1541 | 18 | HN029 | HN040 |  | 726 | 20.177 | 21 | HN027 | HN123 |
| 727 | 20.1488 | 20 | HN029 | HN060 |  | 727 | 20.9548 | 23 | HN027 | HN124 |
| 728 | 18.4602 | 22 | HN029 | HN060 |  | 728 | 19.7142 | 19 | HN027 | HN135 |
| 729 | 21.5851 | 22 | HN029 | HN061 |  | 729 | 19.9108 | 21 | HN027 | HN141 |
| 730 | 25.028 | 26 | HN029 | HN082 |  | 730 | 20.0671 | 23 | HN027 | HN145 |
| 731 | 20.8433 | 20 | HN029 | HN113 |  | 731 | 20.7159 | 24 | HN029 | HN034 |
| 732 | 22.933 | 23 | HN029 | HN116 |  | 732 | 19.6787 | 22 | HN029 | HN051 |
| 733 | 18.5472 | 20 | HN030 | HN054 |  | 733 | 19.2648 | 25 | HN029 | HN065 |
| 734 | 19.9464 | 19 | HN030 | HN058 |  | 734 | 21.0215 | 19 | HN029 | HN087 |
| 735 | 20.8482 | 22 | HN030 | HN075 |  | 735 | 25.0058 | 20 | HN029 | HN097 |
| 736 | 16.4754 | 16 | HN030 | HN077 |  | 736 | 23.985 | 21 | HN029 | HN113 |
| 737 | 17.7383 | 18 | HN030 | HN079 |  | 737 | 24.8266 | 22 | HN029 | HN113 |
| 738 | 20.2288 | 23 | HN030 | HN094 |  | 738 | 19.79 | 23 | HN029 | HN127 |
| 739 | 23.9405 | 24 | HN030 | HN113 |  | 739 | 26.8007 | 26 | HN030 | HN034 |
| 740 | 19.5497 | 19 | HN030 | HN123 |  | 740 | 21.5017 | 21 | HN030 | HN035 |
| 741 | 18.3396 | 21 | HN030 | HN137 |  | 741 | 22.9326 | 22 | HN030 | HN053 |
| 742 | 20.8156 | 21 | HN031 | HN058 |  | 742 | 25.1392 | 28 | HN030 | HN055 |
| 743 | 18.1855 | 20 | HN031 | HN069 |  | 743 | 18.817 | 21 | HN030 | HN057 |
| 744 | 17.8236 | 17 | HN031 | HN075 |  | 744 | 23.03 | 25 | HN030 | HN064 |
| 745 | 13.7147 | 21 | HN031 | HN115 |  | 745 | 22.1455 | 23 | HN030 | HN067 |
| 746 | 21.139 | 21 | HN032 | HN043 |  | 746 | 23.4327 | 24 | HN030 | HN073 |
| 747 | 23.7167 | 24 | HN032 | HN045 |  | 747 | 18.1711 | 20 | HN030 | HN074 |
| 748 | 20.3135 | 23 | HN032 | HN053 |  | 748 | 25.3415 | 26 | HN030 | HN090 |
| 749 | 21.0723 | 24 | HN032 | HN057 |  | 749 | 19.4594 | 18 | HN030 | HN098 |
| 750 | 21.0723 | 24 | HN032 | HN057 |  | 750 | 22.2314 | 25 | HN030 | HN130 |
| 751 | 24.1329 | 23 | HN032 | HN061 |  | 751 | 18.0145 | 19 | HN030 | HN135 |
| 752 | 22.6254 | 26 | HN032 | HN063 |  | 752 | 20.8367 | 21 | HN030 | HN141 |
| 753 | 22.2371 | 22 | HN032 | HN076 |  | 753 | 21.9385 | 25 | HN031 | HN064 |
| 754 | 18.7698 | 20 | HN032 | HN081 |  | 754 | 21.7075 | 25 | HN031 | HN064 |
| 755 | 24.6628 | 24 | HN032 | HN082 |  | 755 | 20.7585 | 24 | HN031 | HN096 |
| 756 | 23.7701 | 24 | HN032 | HN082 |  | 756 | 19.8685 | 24 | HN031 | HN103 |
| 757 | 22.171 | 22 | HN032 | HN083 |  | 757 | 20.4756 | 23 | HN031 | HN107 |
| 758 | 20.3519 | 23 | HN032 | HN107 |  | 758 | 18.4386 | 19 | HN031 | HN109 |
| 759 | 25.5479 | 29 | HN032 | HN112 |  | 759 | 17.9584 | 22 | HN031 | HN114 |
| 760 | 24.378 | 26 | HN032 | HN113 |  | 760 | 19.0743 | 23 | HN031 | HN127 |
| 761 | 24.72 | 27 | HN032 | HN118 |  | 761 | 17.5853 | 18 | HN031 | HN148 |
| 762 | 17.748 | 20 | HN032 | HN118 |  | 762 | 24.4547 | 28 | HN032 | HN050 |
| 763 | 22.3304 | 23 | HN032 | HN128 |  | 763 | 18.5273 | 20 | HN032 | HN052 |
| 764 | 27.1955 | 28 | HN032 | HN129 |  | 764 | 19.5573 | 25 | HN032 | HN054 |
| 765 | 23.1007 | 23 | HN032 | HN132 |  | 765 | 20.1124 | 25 | HN032 | HN055 |
| 766 | 20.3473 | 21 | HN032 | HN136 |  | 766 | 23.1036 | 24 | HN032 | HN060 |
| 767 | 20.1577 | 20 | HN032 | HN140 |  | 767 | 24.4815 | 25 | HN032 | HN061 |
| 768 | 20.1281 | 23 | HN032 | HN143 |  | 768 | 21.5433 | 21 | HN032 | HN074 |
| 769 | 19.6305 | 25 | HN032 | HN145 |  | 769 | 19.6149 | 20 | HN032 | HN076 |
| 770 | 24.7136 | 27 | HN033 | HN034 |  | 770 | 26.6146 | 31 | HN032 | HN083 |
| 771 | 24.7136 | 27 | HN033 | HN034 |  | 771 | 23.1105 | 22 | HN032 | HN093 |
| 772 | 19.6488 | 24 | HN033 | HN039 |  | 772 | 22.397 | 28 | HN032 | HN103 |
| 773 | 23.178 | 25 | HN033 | HN065 |  | 773 | 20.133 | 23 | HN032 | HN109 |
| 774 | 19.2382 | 22 | HN033 | HN066 |  | 774 | 22.1196 | 24 | HN032 | HN111 |
| 775 | 21.5155 | 29 | HN033 | HN103 |  | 775 | 23.7144 | 27 | HN032 | HN112 |
| 776 | 21.9224 | 23 | HN033 | HN113 |  | 776 | 23.4362 | 24 | HN032 | HN113 |
| 777 | 22.3144 | 27 | HN033 | HN127 |  | 777 | 23.4362 | 24 | HN032 | HN113 |
| 778 | 18.9677 | 18 | HN033 | HN139 |  | 778 | 23.7575 | 24 | HN032 | HN118 |
| 779 | 16.6983 | 16 | HN033 | HN140 |  | 779 | 18.9252 | 21 | HN032 | HN119 |
| 780 | 22.5113 | 22 | HN034 | HN035 |  | 780 | 24.4789 | 25 | HN032 | HN123 |
| 781 | 24.8338 | 29 | HN034 | HN037 |  | 781 | 22.2954 | 24 | HN032 | HN125 |
| 782 | 24.8993 | 28 | HN034 | HN039 |  | 782 | 25.719 | 27 | HN032 | HN127 |
| 783 | 21.477 | 23 | HN034 | HN041 |  | 783 | 25.0279 | 28 | HN032 | HN129 |
| 784 | 22.6854 | 25 | HN034 | HN041 |  | 784 | 24.6048 | 25 | HN032 | HN131 |
| 785 | 24.4424 | 26 | HN034 | HN043 |  | 785 | 23.5856 | 25 | HN032 | HN138 |
| 786 | 23.8181 | 23 | HN034 | HN043 |  | 786 | 22.0332 | 22 | HN032 | HN140 |
| 787 | 25.4128 | 27 | HN034 | HN045 |  | 787 | 20.8627 | 25 | HN032 | HN147 |
| 788 | 22.3004 | 23 | HN034 | HN048 |  | 788 | 19.6742 | 25 | HN033 | HN034 |
| 789 | 25.147 | 25 | HN034 | HN048 |  | 789 | 21.1326 | 24 | HN033 | HN039 |
| 790 | 24.6711 | 28 | HN034 | HN051 |  | 790 | 19.1389 | 23 | HN033 | HN039 |
| 791 | 24.76 | 27 | HN034 | HN051 |  | 791 | 20.8367 | 25 | HN033 | HN048 |
| 792 | 22.7328 | 27 | HN034 | HN051 |  | 792 | 22.0779 | 25 | HN033 | HN053 |
| 793 | 24.8984 | 28 | HN034 | HN051 |  | 793 | 20.3779 | 24 | HN033 | HN057 |
| 794 | 25.8247 | 29 | HN034 | HN051 |  | 794 | 19.7653 | 23 | HN033 | HN070 |
| 795 | 22.0912 | 26 | HN034 | HN057 |  | 795 | 21.1883 | 23 | HN033 | HN075 |
| 796 | 23.5158 | 28 | HN034 | HN060 |  | 796 | 21.3181 | 24 | HN033 | HN093 |
| 797 | 25.5878 | 28 | HN034 | HN060 |  | 797 | 21.3181 | 24 | HN033 | HN093 |
| 798 | 24.0741 | 27 | HN034 | HN064 |  | 798 | 21.2289 | 25 | HN033 | HN096 |
| 799 | 24.5596 | 28 | HN034 | HN064 |  | 799 | 23.5292 | 27 | HN033 | HN103 |
| 800 | 25.0612 | 26 | HN034 | HN066 |  | 800 | 21.8983 | 20 | HN033 | HN104 |
| 801 | 26.5738 | 29 | HN034 | HN067 |  | 801 | 20.0483 | 23 | HN033 | HN107 |
| 802 | 27.248 | 30 | HN034 | HN068 |  | 802 | 18.1589 | 21 | HN033 | HN108 |
| 803 | 18.7474 | 23 | HN034 | HN069 |  | 803 | 22.6429 | 22 | HN033 | HN116 |
| 804 | 25.8256 | 27 | HN034 | HN070 |  | 804 | 19.0568 | 21 | HN033 | HN117 |
| 805 | 26.0436 | 27 | HN034 | HN073 |  | 805 | 21.2071 | 27 | HN033 | HN120 |
| 806 | 23.4858 | 22 | HN034 | HN076 |  | 806 | 20.0975 | 25 | HN033 | HN143 |
| 807 | 23.9419 | 25 | HN034 | HN077 |  | 807 | 22.6909 | 26 | HN034 | HN036 |
| 808 | 23.8123 | 26 | HN034 | HN082 |  | 808 | 24.2183 | 25 | HN034 | HN042 |
| 809 | 24.6145 | 31 | HN034 | HN083 |  | 809 | 27.047 | 25 | HN034 | HN043 |
| 810 | 23.1919 | 30 | HN034 | HN083 |  | 810 | 20.8532 | 25 | HN034 | HN046 |
| 811 | 23.6073 | 26 | HN034 | HN083 |  | 811 | 24.1196 | 29 | HN034 | HN046 |
| 812 | 23.6057 | 29 | HN034 | HN083 |  | 812 | 23.868 | 27 | HN034 | HN047 |
| 813 | 22.4426 | 21 | HN034 | HN084 |  | 813 | 25.4223 | 27 | HN034 | HN047 |
| 814 | 23.868 | 25 | HN034 | HN085 |  | 814 | 24.3263 | 24 | HN034 | HN048 |
| 815 | 21.5549 | 25 | HN034 | HN085 |  | 815 | 27.1196 | 30 | HN034 | HN050 |
| 816 | 23.8599 | 25 | HN034 | HN087 |  | 816 | 27.52 | 30 | HN034 | HN050 |
| 817 | 27.2965 | 31 | HN034 | HN090 |  | 817 | 26.1353 | 27 | HN034 | HN060 |
| 818 | 26.8224 | 29 | HN034 | HN091 |  | 818 | 27.4193 | 30 | HN034 | HN060 |
| 819 | 26.6655 | 29 | HN034 | HN093 |  | 819 | 25.6336 | 29 | HN034 | HN061 |
| 820 | 25.067 | 26 | HN034 | HN093 |  | 820 | 23.4202 | 26 | HN034 | HN063 |
| 821 | 23.348 | 26 | HN034 | HN094 |  | 821 | 24.5679 | 27 | HN034 | HN064 |
| 822 | 23.1741 | 27 | HN034 | HN094 |  | 822 | 24.2934 | 28 | HN034 | HN064 |
| 823 | 24.9584 | 29 | HN034 | HN094 |  | 823 | 26.1236 | 25 | HN034 | HN070 |
| 824 | 24.7594 | 26 | HN034 | HN095 |  | 824 | 26.8908 | 30 | HN034 | HN073 |
| 825 | 20.3746 | 25 | HN034 | HN098 |  | 825 | 24.1531 | 24 | HN034 | HN076 |
| 826 | 28.6386 | 33 | HN034 | HN103 |  | 826 | 24.7835 | 26 | HN034 | HN077 |
| 827 | 28.2648 | 32 | HN034 | HN103 |  | 827 | 23.6096 | 27 | HN034 | HN080 |
| 828 | 24.8274 | 29 | HN034 | HN103 |  | 828 | 25.2817 | 29 | HN034 | HN086 |
| 829 | 26.5235 | 27 | HN034 | HN103 |  | 829 | 22.902 | 27 | HN034 | HN091 |
| 830 | 22.1278 | 29 | HN034 | HN105 |  | 830 | 24.3453 | 27 | HN034 | HN093 |
| 831 | 25.1433 | 28 | HN034 | HN107 |  | 831 | 20.7182 | 24 | HN034 | HN095 |
| 832 | 24.3017 | 27 | HN034 | HN107 |  | 832 | 24.0412 | 27 | HN034 | HN096 |
| 833 | 24.7063 | 28 | HN034 | HN108 |  | 833 | 25.6459 | 30 | HN034 | HN096 |
| 834 | 21.4428 | 27 | HN034 | HN112 |  | 834 | 25.5126 | 27 | HN034 | HN097 |
| 835 | 27.2389 | 27 | HN034 | HN113 |  | 835 | 26.5543 | 30 | HN034 | HN102 |
| 836 | 25.7701 | 28 | HN034 | HN113 |  | 836 | 27.9998 | 28 | HN034 | HN104 |
| 837 | 22.065 | 26 | HN034 | HN115 |  | 837 | 23.5281 | 26 | HN034 | HN104 |
| 838 | 28.2955 | 28 | HN034 | HN116 |  | 838 | 24.4184 | 19 | HN034 | HN106 |
| 839 | 28.1885 | 28 | HN034 | HN116 |  | 839 | 23.0743 | 24 | HN034 | HN106 |
| 840 | 23.6182 | 25 | HN034 | HN116 |  | 840 | 23.9297 | 26 | HN034 | HN107 |
| 841 | 22.1165 | 24 | HN034 | HN117 |  | 841 | 23.7262 | 26 | HN034 | HN107 |
| 842 | 27.0641 | 27 | HN034 | HN117 |  | 842 | 23.7262 | 26 | HN034 | HN107 |
| 843 | 25.1389 | 28 | HN034 | HN118 |  | 843 | 26.166 | 29 | HN034 | HN112 |
| 844 | 23.6421 | 26 | HN034 | HN118 |  | 844 | 26.8302 | 29 | HN034 | HN112 |
| 845 | 25.223 | 26 | HN034 | HN118 |  | 845 | 29.6379 | 29 | HN034 | HN116 |
| 846 | 24.5042 | 30 | HN034 | HN119 |  | 846 | 29.9619 | 29 | HN034 | HN116 |
| 847 | 24.5079 | 28 | HN034 | HN123 |  | 847 | 24.917 | 28 | HN034 | HN118 |
| 848 | 24.3184 | 27 | HN034 | HN126 |  | 848 | 25.3311 | 28 | HN034 | HN118 |
| 849 | 25.6555 | 30 | HN034 | HN126 |  | 849 | 23.1273 | 25 | HN034 | HN122 |
| 850 | 26.4736 | 31 | HN034 | HN127 |  | 850 | 24.9414 | 26 | HN034 | HN125 |
| 851 | 25.5251 | 30 | HN034 | HN127 |  | 851 | 23.1369 | 26 | HN034 | HN126 |
| 852 | 25.9819 | 28 | HN034 | HN130 |  | 852 | 23.3266 | 28 | HN034 | HN130 |
| 853 | 25.6903 | 29 | HN034 | HN131 |  | 853 | 23.1313 | 27 | HN034 | HN133 |
| 854 | 24.6091 | 27 | HN034 | HN131 |  | 854 | 24.7748 | 25 | HN034 | HN135 |
| 855 | 23.2673 | 30 | HN034 | HN131 |  | 855 | 22.6754 | 26 | HN034 | HN138 |
| 856 | 25.1685 | 27 | HN034 | HN131 |  | 856 | 26.9848 | 28 | HN034 | HN140 |
| 857 | 22.7347 | 26 | HN034 | HN132 |  | 857 | 26.4686 | 26 | HN034 | HN141 |
| 858 | 24.0117 | 27 | HN034 | HN132 |  | 858 | 21.1005 | 26 | HN034 | HN147 |
| 859 | 22.0908 | 26 | HN034 | HN132 |  | 859 | 24.8898 | 30 | HN034 | HN147 |
| 860 | 22.4197 | 25 | HN034 | HN134 |  | 860 | 23.1068 | 25 | HN034 | HN150 |
| 861 | 23.6151 | 25 | HN034 | HN134 |  | 861 | 23.4202 | 23 | HN035 | HN040 |
| 862 | 24.0847 | 26 | HN034 | HN134 |  | 862 | 19.7074 | 20 | HN035 | HN048 |
| 863 | 24.1596 | 25 | HN034 | HN134 |  | 863 | 23.2791 | 23 | HN035 | HN049 |
| 864 | 21.8102 | 26 | HN034 | HN135 |  | 864 | 22.208 | 22 | HN035 | HN049 |
| 865 | 23.6156 | 26 | HN034 | HN136 |  | 865 | 22.1307 | 23 | HN035 | HN060 |
| 866 | 24.6374 | 28 | HN034 | HN137 |  | 866 | 24.2394 | 26 | HN035 | HN061 |
| 867 | 25.0612 | 26 | HN034 | HN144 |  | 867 | 23.6833 | 27 | HN035 | HN065 |
| 868 | 23.1827 | 26 | HN034 | HN144 |  | 868 | 23.8979 | 27 | HN035 | HN067 |
| 869 | 20.5363 | 26 | HN034 | HN145 |  | 869 | 21.9496 | 25 | HN035 | HN069 |
| 870 | 20.7452 | 24 | HN034 | HN145 |  | 870 | 18.0098 | 21 | HN035 | HN079 |
| 871 | 23.2999 | 28 | HN034 | HN145 |  | 871 | 25.1391 | 23 | HN035 | HN087 |
| 872 | 24.5738 | 27 | HN034 | HN147 |  | 872 | 22.6795 | 25 | HN035 | HN090 |
| 873 | 23.252 | 23 | HN035 | HN040 |  | 873 | 24.8477 | 25 | HN035 | HN091 |
| 874 | 20.5034 | 18 | HN035 | HN041 |  | 874 | 22.2453 | 29 | HN035 | HN096 |
| 875 | 23.1844 | 24 | HN035 | HN045 |  | 875 | 25.8223 | 24 | HN035 | HN097 |
| 876 | 24.6401 | 27 | HN035 | HN061 |  | 876 | 25.7813 | 23 | HN035 | HN097 |
| 877 | 19.747 | 23 | HN035 | HN067 |  | 877 | 23.0556 | 19 | HN035 | HN106 |
| 878 | 19.1112 | 22 | HN035 | HN071 |  | 878 | 22.1843 | 22 | HN035 | HN111 |
| 879 | 25.0817 | 25 | HN035 | HN082 |  | 879 | 22.6927 | 23 | HN035 | HN113 |
| 880 | 20.2632 | 21 | HN035 | HN084 |  | 880 | 26.2861 | 29 | HN035 | HN120 |
| 881 | 21.4247 | 23 | HN035 | HN094 |  | 881 | 26.3528 | 30 | HN035 | HN120 |
| 882 | 21.3967 | 22 | HN035 | HN094 |  | 882 | 22.9518 | 23 | HN035 | HN123 |
| 883 | 25.9151 | 30 | HN035 | HN105 |  | 883 | 19.5352 | 23 | HN035 | HN124 |
| 884 | 22.1654 | 23 | HN035 | HN124 |  | 884 | 23.5047 | 27 | HN035 | HN146 |
| 885 | 19.6259 | 21 | HN035 | HN130 |  | 885 | 20.4623 | 25 | HN035 | HN147 |
| 886 | 23.1851 | 23 | HN035 | HN133 |  | 886 | 19.4228 | 25 | HN036 | HN054 |
| 887 | 22.682 | 24 | HN035 | HN137 |  | 887 | 15.982 | 18 | HN036 | HN058 |
| 888 | 23.0872 | 24 | HN035 | HN146 |  | 888 | 18.5108 | 20 | HN036 | HN058 |
| 889 | 22.6567 | 23 | HN036 | HN082 |  | 889 | 18.5593 | 20 | HN036 | HN113 |
| 890 | 16.394 | 21 | HN036 | HN112 |  | 890 | 20.6702 | 23 | HN036 | HN116 |
| 891 | 19.582 | 25 | HN036 | HN112 |  | 891 | 20.4839 | 21 | HN036 | HN118 |
| 892 | 18.7828 | 20 | HN036 | HN118 |  | 892 | 21.0369 | 26 | HN037 | HN049 |
| 893 | 20.398 | 19 | HN036 | HN144 |  | 893 | 22.071 | 28 | HN037 | HN049 |
| 894 | 20.5153 | 25 | HN037 | HN043 |  | 894 | 22.5737 | 25 | HN037 | HN057 |
| 895 | 19.1382 | 24 | HN037 | HN052 |  | 895 | 20.6569 | 28 | HN037 | HN066 |
| 896 | 22.786 | 28 | HN037 | HN055 |  | 896 | 23.1402 | 24 | HN037 | HN067 |
| 897 | 22.2031 | 26 | HN037 | HN056 |  | 897 | 20.1354 | 24 | HN037 | HN069 |
| 898 | 22.8161 | 25 | HN037 | HN058 |  | 898 | 19.7545 | 26 | HN037 | HN072 |
| 899 | 23.1438 | 28 | HN037 | HN063 |  | 899 | 25.0033 | 28 | HN037 | HN073 |
| 900 | 20.6038 | 25 | HN037 | HN067 |  | 900 | 25.0033 | 28 | HN037 | HN073 |
| 901 | 19.0887 | 26 | HN037 | HN074 |  | 901 | 21.6092 | 24 | HN037 | HN082 |
| 902 | 19.2473 | 25 | HN037 | HN075 |  | 902 | 26.3202 | 30 | HN037 | HN090 |
| 903 | 22.1469 | 27 | HN037 | HN079 |  | 903 | 21.0901 | 26 | HN037 | HN091 |
| 904 | 26.6934 | 28 | HN037 | HN082 |  | 904 | 18.8904 | 21 | HN037 | HN092 |
| 905 | 28.3382 | 31 | HN037 | HN082 |  | 905 | 24.8432 | 30 | HN037 | HN094 |
| 906 | 26.3237 | 30 | HN037 | HN083 |  | 906 | 24.4045 | 25 | HN037 | HN097 |
| 907 | 26.8932 | 31 | HN037 | HN083 |  | 907 | 18.8263 | 23 | HN037 | HN104 |
| 908 | 20.94 | 27 | HN037 | HN103 |  | 908 | 23.6511 | 26 | HN037 | HN107 |
| 909 | 23.9926 | 24 | HN037 | HN113 |  | 909 | 23 | 22 | HN037 | HN113 |
| 910 | 23.2491 | 24 | HN037 | HN113 |  | 910 | 23.3983 | 25 | HN037 | HN113 |
| 911 | 22.2435 | 26 | HN037 | HN125 |  | 911 | 22.9312 | 24 | HN037 | HN113 |
| 912 | 22.9484 | 29 | HN037 | HN127 |  | 912 | 23.7964 | 26 | HN037 | HN118 |
| 913 | 21.3066 | 26 | HN037 | HN129 |  | 913 | 21.7349 | 24 | HN037 | HN141 |
| 914 | 25.7102 | 28 | HN037 | HN129 |  | 914 | 20.3501 | 23 | HN037 | HN143 |
| 915 | 21.1958 | 27 | HN037 | HN133 |  | 915 | 21.3906 | 24 | HN038 | HN043 |
| 916 | 18.4603 | 24 | HN037 | HN134 |  | 916 | 21.4026 | 24 | HN038 | HN050 |
| 917 | 20.2823 | 28 | HN037 | HN137 |  | 917 | 17.1333 | 24 | HN038 | HN056 |
| 918 | 21.2057 | 25 | HN037 | HN138 |  | 918 | 20.1732 | 22 | HN038 | HN066 |
| 919 | 20.297 | 25 | HN037 | HN147 |  | 919 | 26.0907 | 24 | HN038 | HN082 |
| 920 | 19.4363 | 26 | HN038 | HN055 |  | 920 | 26.0765 | 24 | HN038 | HN082 |
| 921 | 16.4402 | 21 | HN038 | HN055 |  | 921 | 21.0237 | 18 | HN038 | HN097 |
| 922 | 18.6815 | 24 | HN038 | HN059 |  | 922 | 20.589 | 21 | HN038 | HN111 |
| 923 | 22.3036 | 23 | HN038 | HN064 |  | 923 | 20.9197 | 24 | HN038 | HN122 |
| 924 | 21.6354 | 26 | HN038 | HN068 |  | 924 | 22.1797 | 28 | HN038 | HN131 |
| 925 | 15.5961 | 17 | HN038 | HN076 |  | 925 | 22.4535 | 25 | HN039 | HN043 |
| 926 | 20.415 | 23 | HN038 | HN080 |  | 926 | 22.6846 | 25 | HN039 | HN045 |
| 927 | 22.5568 | 25 | HN038 | HN087 |  | 927 | 20.9789 | 23 | HN039 | HN048 |
| 928 | 19.2256 | 22 | HN038 | HN102 |  | 928 | 19.5289 | 24 | HN039 | HN055 |
| 929 | 22.2832 | 22 | HN038 | HN113 |  | 929 | 18.6466 | 23 | HN039 | HN058 |
| 930 | 17.9349 | 21 | HN038 | HN123 |  | 930 | 23.2307 | 26 | HN039 | HN060 |
| 931 | 20.9279 | 26 | HN038 | HN125 |  | 931 | 20.3269 | 26 | HN039 | HN065 |
| 932 | 22.3466 | 26 | HN038 | HN127 |  | 932 | 23.0065 | 29 | HN039 | HN067 |
| 933 | 21.2255 | 23 | HN039 | HN048 |  | 933 | 26.2087 | 26 | HN039 | HN073 |
| 934 | 23.6842 | 27 | HN039 | HN051 |  | 934 | 17.8137 | 24 | HN039 | HN079 |
| 935 | 22.8245 | 24 | HN039 | HN058 |  | 935 | 18.7922 | 26 | HN039 | HN080 |
| 936 | 22.3548 | 28 | HN039 | HN069 |  | 936 | 23.8294 | 29 | HN039 | HN083 |
| 937 | 22.6935 | 28 | HN039 | HN073 |  | 937 | 23.7578 | 25 | HN039 | HN087 |
| 938 | 24.2028 | 26 | HN039 | HN080 |  | 938 | 23.2048 | 26 | HN039 | HN110 |
| 939 | 20.8879 | 23 | HN039 | HN090 |  | 939 | 23.3626 | 25 | HN039 | HN116 |
| 940 | 10.9768 | 17 | HN039 | HN095 |  | 940 | 22.7136 | 26 | HN039 | HN118 |
| 941 | 20.8577 | 25 | HN039 | HN101 |  | 941 | 23.5899 | 25 | HN039 | HN124 |
| 942 | 19.2129 | 23 | HN039 | HN102 |  | 942 | 23.8256 | 28 | HN039 | HN129 |
| 943 | 24.5168 | 25 | HN039 | HN116 |  | 943 | 21.6051 | 24 | HN039 | HN132 |
| 944 | 18.3711 | 23 | HN039 | HN116 |  | 944 | 18.5925 | 22 | HN039 | HN144 |
| 945 | 20.7465 | 26 | HN039 | HN126 |  | 945 | 20.5682 | 26 | HN039 | HN149 |
| 946 | 22.5737 | 28 | HN039 | HN129 |  | 946 | 16.568 | 18 | HN040 | HN041 |
| 947 | 21.6754 | 24 | HN039 | HN132 |  | 947 | 20.7838 | 28 | HN040 | HN050 |
| 948 | 16.1995 | 21 | HN040 | HN042 |  | 948 | 22.4534 | 26 | HN040 | HN082 |
| 949 | 17.923 | 22 | HN040 | HN042 |  | 949 | 22.5622 | 27 | HN040 | HN083 |
| 950 | 21.8 | 26 | HN040 | HN051 |  | 950 | 20.7777 | 25 | HN040 | HN083 |
| 951 | 23.8025 | 26 | HN040 | HN073 |  | 951 | 21.678 | 25 | HN040 | HN094 |
| 952 | 19.8796 | 27 | HN040 | HN103 |  | 952 | 23.4425 | 27 | HN040 | HN118 |
| 953 | 18.2033 | 14 | HN040 | HN106 |  | 953 | 22.4907 | 29 | HN040 | HN131 |
| 954 | 19.9456 | 26 | HN040 | HN107 |  | 954 | 21.5703 | 27 | HN040 | HN131 |
| 955 | 22.6791 | 26 | HN040 | HN116 |  | 955 | 16.8861 | 22 | HN040 | HN133 |
| 956 | 23.3416 | 25 | HN040 | HN116 |  | 956 | 16.3532 | 20 | HN040 | HN134 |
| 957 | 19.0457 | 22 | HN040 | HN132 |  | 957 | 20.8668 | 25 | HN040 | HN144 |
| 958 | 21.9603 | 23 | HN041 | HN068 |  | 958 | 22.3917 | 28 | HN041 | HN045 |
| 959 | 22.899 | 28 | HN041 | HN069 |  | 959 | 20.363 | 22 | HN041 | HN048 |
| 960 | 24.2602 | 28 | HN041 | HN073 |  | 960 | 20.0985 | 25 | HN041 | HN052 |
| 961 | 19.7202 | 24 | HN041 | HN079 |  | 961 | 23.5208 | 26 | HN041 | HN064 |
| 962 | 16.4565 | 20 | HN041 | HN084 |  | 962 | 21.5389 | 23 | HN041 | HN075 |
| 963 | 23.6444 | 23 | HN041 | HN097 |  | 963 | 23.3953 | 29 | HN041 | HN083 |
| 964 | 20.8399 | 24 | HN041 | HN124 |  | 964 | 22.2013 | 26 | HN041 | HN094 |
| 965 | 20.8399 | 24 | HN041 | HN124 |  | 965 | 22.4068 | 27 | HN041 | HN103 |
| 966 | 20.8029 | 23 | HN041 | HN130 |  | 966 | 19.9286 | 24 | HN041 | HN107 |
| 967 | 15.8962 | 18 | HN041 | HN135 |  | 967 | 20.0474 | 25 | HN041 | HN115 |
| 968 | 20.3934 | 20 | HN041 | HN139 |  | 968 | 25.7452 | 28 | HN041 | HN116 |
| 969 | 21.2409 | 26 | HN041 | HN147 |  | 969 | 20.9921 | 23 | HN041 | HN118 |
| 970 | 24.6486 | 29 | HN042 | HN051 |  | 970 | 17.5803 | 20 | HN041 | HN122 |
| 971 | 22.3193 | 25 | HN042 | HN060 |  | 971 | 20.5804 | 25 | HN041 | HN136 |
| 972 | 20.7911 | 23 | HN042 | HN060 |  | 972 | 20.5804 | 25 | HN041 | HN136 |
| 973 | 22.2256 | 25 | HN042 | HN064 |  | 973 | 21.0068 | 26 | HN041 | HN136 |
| 974 | 22.0094 | 25 | HN042 | HN065 |  | 974 | 23.7543 | 26 | HN041 | HN138 |
| 975 | 21.6779 | 24 | HN042 | HN090 |  | 975 | 23.0702 | 26 | HN041 | HN144 |
| 976 | 23.4242 | 25 | HN042 | HN116 |  | 976 | 21.3953 | 22 | HN042 | HN043 |
| 977 | 17.029 | 22 | HN042 | HN118 |  | 977 | 18.2048 | 25 | HN042 | HN063 |
| 978 | 20.8367 | 26 | HN042 | HN129 |  | 978 | 21.9231 | 24 | HN042 | HN064 |
| 979 | 19.6192 | 22 | HN042 | HN132 |  | 979 | 19.7453 | 22 | HN042 | HN067 |
| 980 | 22.4589 | 25 | HN042 | HN140 |  | 980 | 23.5148 | 24 | HN042 | HN068 |
| 981 | 19.4621 | 17 | HN043 | HN049 |  | 981 | 23.8021 | 25 | HN042 | HN082 |
| 982 | 22.1791 | 26 | HN043 | HN050 |  | 982 | 18.454 | 21 | HN042 | HN085 |
| 983 | 22.7118 | 24 | HN043 | HN052 |  | 983 | 19.53 | 26 | HN042 | HN085 |
| 984 | 21.9712 | 26 | HN043 | HN055 |  | 984 | 24.5968 | 26 | HN042 | HN090 |
| 985 | 23.4184 | 24 | HN043 | HN055 |  | 985 | 20.0802 | 27 | HN042 | HN094 |
| 986 | 23.3213 | 24 | HN043 | HN067 |  | 986 | 22.8753 | 29 | HN042 | HN096 |
| 987 | 20.951 | 22 | HN043 | HN067 |  | 987 | 24.118 | 21 | HN042 | HN106 |
| 988 | 20.9578 | 24 | HN043 | HN072 |  | 988 | 19.3452 | 25 | HN042 | HN115 |
| 989 | 29.0766 | 30 | HN043 | HN073 |  | 989 | 19.9917 | 25 | HN042 | HN126 |
| 990 | 23.5417 | 23 | HN043 | HN076 |  | 990 | 20.3804 | 26 | HN042 | HN133 |
| 991 | 19.7693 | 19 | HN043 | HN080 |  | 991 | 17.7708 | 25 | HN042 | HN140 |
| 992 | 24.6136 | 26 | HN043 | HN082 |  | 992 | 19.7065 | 23 | HN043 | HN046 |
| 993 | 25.3586 | 24 | HN043 | HN083 |  | 993 | 19.4257 | 19 | HN043 | HN049 |
| 994 | 26.9587 | 32 | HN043 | HN083 |  | 994 | 23.5305 | 28 | HN043 | HN050 |
| 995 | 26.0748 | 27 | HN043 | HN083 |  | 995 | 25.8401 | 27 | HN043 | HN057 |
| 996 | 23.7633 | 27 | HN043 | HN083 |  | 996 | 22.6421 | 23 | HN043 | HN058 |
| 997 | 19.7645 | 18 | HN043 | HN088 |  | 997 | 24.0988 | 23 | HN043 | HN061 |
| 998 | 21.5612 | 26 | HN043 | HN090 |  | 998 | 24.5325 | 26 | HN043 | HN063 |
| 999 | 23.7034 | 26 | HN043 | HN094 |  | 999 | 22.2128 | 25 | HN043 | HN064 |
| 1000 | 23.5534 | 22 | HN043 | HN096 |  | 1000 | 22.6578 | 20 | HN043 | HN066 |
| 1001 | 23.7333 | 19 | HN043 | HN097 |  | 1001 | 24.5891 | 29 | HN043 | HN068 |
| 1002 | 21.1117 | 22 | HN043 | HN098 |  | 1002 | 22.5224 | 21 | HN043 | HN072 |
| 1003 | 20.8586 | 19 | HN043 | HN100 |  | 1003 | 23.2215 | 21 | HN043 | HN073 |
| 1004 | 21.267 | 22 | HN043 | HN102 |  | 1004 | 25.4742 | 26 | HN043 | HN074 |
| 1005 | 19.4366 | 18 | HN043 | HN104 |  | 1005 | 25.8696 | 27 | HN043 | HN082 |
| 1006 | 21.2781 | 24 | HN043 | HN107 |  | 1006 | 28.2153 | 31 | HN043 | HN083 |
| 1007 | 19.9425 | 22 | HN043 | HN110 |  | 1007 | 21.7008 | 26 | HN043 | HN083 |
| 1008 | 23.859 | 27 | HN043 | HN116 |  | 1008 | 27.2565 | 26 | HN043 | HN090 |
| 1009 | 18.9281 | 21 | HN043 | HN124 |  | 1009 | 21.1503 | 22 | HN043 | HN092 |
| 1010 | 24.3635 | 29 | HN043 | HN127 |  | 1010 | 27.7892 | 28 | HN043 | HN094 |
| 1011 | 23.9876 | 30 | HN043 | HN127 |  | 1011 | 28.1849 | 31 | HN043 | HN094 |
| 1012 | 27.4718 | 30 | HN043 | HN129 |  | 1012 | 25.6834 | 27 | HN043 | HN096 |
| 1013 | 28.7771 | 30 | HN043 | HN129 |  | 1013 | 25.3228 | 25 | HN043 | HN098 |
| 1014 | 28.3512 | 31 | HN043 | HN129 |  | 1014 | 24.547 | 27 | HN043 | HN103 |
| 1015 | 26.1968 | 28 | HN043 | HN130 |  | 1015 | 24.1574 | 21 | HN043 | HN106 |
| 1016 | 25.0437 | 25 | HN043 | HN138 |  | 1016 | 24.151 | 26 | HN043 | HN107 |
| 1017 | 25.0043 | 24 | HN043 | HN143 |  | 1017 | 21.4928 | 21 | HN043 | HN110 |
| 1018 | 20.0743 | 23 | HN043 | HN145 |  | 1018 | 23.0868 | 23 | HN043 | HN114 |
| 1019 | 17.5152 | 22 | HN044 | HN052 |  | 1019 | 21.4119 | 23 | HN043 | HN114 |
| 1020 | 22.9291 | 25 | HN044 | HN082 |  | 1020 | 21.5697 | 22 | HN043 | HN116 |
| 1021 | 21.4583 | 25 | HN044 | HN090 |  | 1021 | 21.2629 | 23 | HN043 | HN121 |
| 1022 | 17.6734 | 23 | HN044 | HN117 |  | 1022 | 25.8354 | 28 | HN043 | HN125 |
| 1023 | 16.9136 | 20 | HN044 | HN126 |  | 1023 | 28.2761 | 26 | HN043 | HN129 |
| 1024 | 18.3339 | 24 | HN044 | HN129 |  | 1024 | 29.7069 | 30 | HN043 | HN129 |
| 1025 | 22.0142 | 27 | HN045 | HN051 |  | 1025 | 27.9335 | 29 | HN043 | HN129 |
| 1026 | 24.3372 | 28 | HN045 | HN064 |  | 1026 | 21.8102 | 22 | HN043 | HN142 |
| 1027 | 18.6103 | 20 | HN045 | HN066 |  | 1027 | 17.38 | 23 | HN044 | HN059 |
| 1028 | 22.4699 | 28 | HN045 | HN067 |  | 1028 | 22.6894 | 26 | HN044 | HN064 |
| 1029 | 16.5667 | 21 | HN045 | HN070 |  | 1029 | 23.4757 | 27 | HN044 | HN065 |
| 1030 | 24.515 | 27 | HN045 | HN073 |  | 1030 | 25.0208 | 25 | HN044 | HN068 |
| 1031 | 25.6204 | 29 | HN045 | HN073 |  | 1031 | 23.9733 | 24 | HN044 | HN082 |
| 1032 | 23.4688 | 30 | HN045 | HN083 |  | 1032 | 24.9136 | 31 | HN044 | HN083 |
| 1033 | 22.6682 | 20 | HN045 | HN106 |  | 1033 | 24.1175 | 31 | HN044 | HN083 |
| 1034 | 22.1813 | 23 | HN045 | HN111 |  | 1034 | 21.7306 | 24 | HN044 | HN113 |
| 1035 | 24.5101 | 25 | HN045 | HN113 |  | 1035 | 18.2224 | 22 | HN044 | HN116 |
| 1036 | 21.6507 | 28 | HN045 | HN116 |  | 1036 | 20.0719 | 24 | HN044 | HN118 |
| 1037 | 22.7839 | 28 | HN045 | HN127 |  | 1037 | 20.2724 | 24 | HN044 | HN125 |
| 1038 | 25.3802 | 30 | HN045 | HN129 |  | 1038 | 16.9078 | 20 | HN044 | HN141 |
| 1039 | 25.3445 | 29 | HN045 | HN129 |  | 1039 | 19.3837 | 25 | HN044 | HN143 |
| 1040 | 19.3854 | 24 | HN045 | HN135 |  | 1040 | 20.2335 | 24 | HN045 | HN047 |
| 1041 | 21.3988 | 27 | HN045 | HN137 |  | 1041 | 25.3759 | 32 | HN045 | HN060 |
| 1042 | 25.3376 | 28 | HN046 | HN073 |  | 1042 | 22.3493 | 25 | HN045 | HN061 |
| 1043 | 20.413 | 24 | HN046 | HN077 |  | 1043 | 22.7968 | 27 | HN045 | HN068 |
| 1044 | 20.8209 | 26 | HN046 | HN125 |  | 1044 | 21.6755 | 27 | HN045 | HN074 |
| 1045 | 21.0751 | 26 | HN046 | HN131 |  | 1045 | 21.1228 | 28 | HN045 | HN083 |
| 1046 | 18.7363 | 25 | HN046 | HN137 |  | 1046 | 24.2494 | 28 | HN045 | HN090 |
| 1047 | 20.1889 | 25 | HN046 | HN143 |  | 1047 | 23.6364 | 28 | HN045 | HN094 |
| 1048 | 13.7319 | 17 | HN047 | HN048 |  | 1048 | 21.3539 | 24 | HN045 | HN104 |
| 1049 | 26.069 | 31 | HN047 | HN058 |  | 1049 | 24.6016 | 28 | HN045 | HN105 |
| 1050 | 18.529 | 26 | HN047 | HN060 |  | 1050 | 16.8935 | 23 | HN045 | HN112 |
| 1051 | 25.1113 | 28 | HN047 | HN064 |  | 1051 | 23.9672 | 27 | HN045 | HN116 |
| 1052 | 16.9332 | 21 | HN047 | HN069 |  | 1052 | 21.9092 | 22 | HN045 | HN118 |
| 1053 | 25.7362 | 24 | HN047 | HN082 |  | 1053 | 22.8116 | 26 | HN045 | HN118 |
| 1054 | 26.4375 | 31 | HN047 | HN094 |  | 1054 | 19.1545 | 24 | HN045 | HN119 |
| 1055 | 22.7935 | 28 | HN047 | HN096 |  | 1055 | 23.0079 | 26 | HN045 | HN125 |
| 1056 | 21.5695 | 25 | HN047 | HN110 |  | 1056 | 19.0479 | 24 | HN045 | HN134 |
| 1057 | 20.5885 | 29 | HN047 | HN127 |  | 1057 | 21.3133 | 25 | HN045 | HN135 |
| 1058 | 18.4896 | 22 | HN047 | HN128 |  | 1058 | 22.8884 | 24 | HN045 | HN144 |
| 1059 | 22.1043 | 27 | HN047 | HN131 |  | 1059 | 19.648 | 26 | HN046 | HN055 |
| 1060 | 20.2621 | 25 | HN047 | HN132 |  | 1060 | 24.3827 | 25 | HN046 | HN082 |
| 1061 | 24.3195 | 27 | HN048 | HN053 |  | 1061 | 22.359 | 29 | HN046 | HN083 |
| 1062 | 22.4636 | 29 | HN048 | HN083 |  | 1062 | 23.4327 | 27 | HN046 | HN107 |
| 1063 | 18.2151 | 19 | HN048 | HN092 |  | 1063 | 21.1921 | 21 | HN046 | HN111 |
| 1064 | 20.1603 | 20 | HN048 | HN093 |  | 1064 | 22.2467 | 28 | HN046 | HN131 |
| 1065 | 22.1626 | 25 | HN048 | HN098 |  | 1065 | 19.2383 | 24 | HN047 | HN055 |
| 1066 | 25.7663 | 25 | HN048 | HN113 |  | 1066 | 22.0645 | 29 | HN047 | HN058 |
| 1067 | 18.4609 | 23 | HN048 | HN128 |  | 1067 | 18.0161 | 23 | HN047 | HN059 |
| 1068 | 17.7471 | 21 | HN048 | HN128 |  | 1068 | 19.1067 | 26 | HN047 | HN067 |
| 1069 | 22.031 | 26 | HN048 | HN129 |  | 1069 | 21.4177 | 27 | HN047 | HN067 |
| 1070 | 17.1982 | 16 | HN048 | HN135 |  | 1070 | 18.7175 | 25 | HN047 | HN072 |
| 1071 | 19.0182 | 23 | HN049 | HN051 |  | 1071 | 21.8565 | 26 | HN047 | HN073 |
| 1072 | 23.4999 | 27 | HN049 | HN051 |  | 1072 | 20.845 | 28 | HN047 | HN090 |
| 1073 | 18.7957 | 22 | HN049 | HN052 |  | 1073 | 27.3372 | 33 | HN047 | HN094 |
| 1074 | 23.887 | 27 | HN049 | HN064 |  | 1074 | 21.5703 | 27 | HN047 | HN105 |
| 1075 | 19.4051 | 19 | HN049 | HN067 |  | 1075 | 20.5797 | 20 | HN047 | HN106 |
| 1076 | 18.1057 | 21 | HN049 | HN069 |  | 1076 | 25.7527 | 26 | HN047 | HN116 |
| 1077 | 18.9723 | 22 | HN049 | HN069 |  | 1077 | 24.5831 | 28 | HN047 | HN118 |
| 1078 | 25.3756 | 26 | HN049 | HN073 |  | 1078 | 18.3023 | 25 | HN047 | HN133 |
| 1079 | 21.6904 | 24 | HN049 | HN081 |  | 1079 | 17.3863 | 24 | HN047 | HN136 |
| 1080 | 24.2408 | 28 | HN049 | HN094 |  | 1080 | 21.0886 | 22 | HN048 | HN050 |
| 1081 | 21.2434 | 18 | HN049 | HN097 |  | 1081 | 20.2787 | 22 | HN048 | HN054 |
| 1082 | 19.0955 | 22 | HN049 | HN109 |  | 1082 | 20.9405 | 23 | HN048 | HN063 |
| 1083 | 18.5601 | 19 | HN049 | HN117 |  | 1083 | 20.8358 | 21 | HN048 | HN072 |
| 1084 | 19.264 | 21 | HN049 | HN122 |  | 1084 | 23.2451 | 28 | HN048 | HN083 |
| 1085 | 19.2188 | 21 | HN049 | HN122 |  | 1085 | 18.3451 | 18 | HN048 | HN084 |
| 1086 | 23.7961 | 29 | HN049 | HN127 |  | 1086 | 22.2045 | 24 | HN048 | HN098 |
| 1087 | 20.8961 | 23 | HN049 | HN137 |  | 1087 | 21.3218 | 24 | HN048 | HN101 |
| 1088 | 23.7001 | 28 | HN050 | HN051 |  | 1088 | 21.28 | 22 | HN048 | HN108 |
| 1089 | 22.745 | 26 | HN050 | HN053 |  | 1089 | 19.5301 | 20 | HN048 | HN110 |
| 1090 | 20.3177 | 25 | HN050 | HN054 |  | 1090 | 21.5282 | 23 | HN048 | HN118 |
| 1091 | 22.7023 | 27 | HN050 | HN058 |  | 1091 | 21.9346 | 23 | HN048 | HN118 |
| 1092 | 19.8764 | 25 | HN050 | HN060 |  | 1092 | 25.2045 | 29 | HN048 | HN125 |
| 1093 | 25.4039 | 29 | HN050 | HN061 |  | 1093 | 21.5235 | 24 | HN048 | HN137 |
| 1094 | 24.0761 | 28 | HN050 | HN073 |  | 1094 | 21.5203 | 21 | HN049 | HN061 |
| 1095 | 27.0556 | 28 | HN050 | HN073 |  | 1095 | 17.7084 | 22 | HN049 | HN063 |
| 1096 | 21.3888 | 22 | HN050 | HN076 |  | 1096 | 22.8956 | 25 | HN049 | HN064 |
| 1097 | 28.6045 | 32 | HN050 | HN082 |  | 1097 | 22.5416 | 26 | HN049 | HN067 |
| 1098 | 24.9537 | 26 | HN050 | HN082 |  | 1098 | 20.915 | 24 | HN049 | HN079 |
| 1099 | 18.8747 | 23 | HN050 | HN091 |  | 1099 | 25.9134 | 26 | HN049 | HN082 |
| 1100 | 23.3169 | 26 | HN050 | HN093 |  | 1100 | 24.7795 | 28 | HN049 | HN094 |
| 1101 | 18.5777 | 24 | HN050 | HN098 |  | 1101 | 20.3826 | 27 | HN049 | HN096 |
| 1102 | 21.4092 | 26 | HN050 | HN101 |  | 1102 | 26.4283 | 29 | HN049 | HN096 |
| 1103 | 21.9583 | 27 | HN050 | HN101 |  | 1103 | 26.2952 | 25 | HN049 | HN113 |
| 1104 | 19.5065 | 27 | HN050 | HN102 |  | 1104 | 19.7181 | 23 | HN049 | HN147 |
| 1105 | 26.1858 | 31 | HN050 | HN103 |  | 1105 | 25.1406 | 26 | HN050 | HN061 |
| 1106 | 20.5529 | 27 | HN050 | HN105 |  | 1106 | 24.7045 | 29 | HN050 | HN064 |
| 1107 | 24.8156 | 29 | HN050 | HN105 |  | 1107 | 25.214 | 28 | HN050 | HN066 |
| 1108 | 21.4209 | 17 | HN050 | HN106 |  | 1108 | 25.2334 | 30 | HN050 | HN068 |
| 1109 | 22.7005 | 25 | HN050 | HN111 |  | 1109 | 24.5752 | 29 | HN050 | HN071 |
| 1110 | 23.8448 | 26 | HN050 | HN111 |  | 1110 | 24.2942 | 27 | HN050 | HN072 |
| 1111 | 22.7567 | 27 | HN050 | HN115 |  | 1111 | 24.6776 | 28 | HN050 | HN072 |
| 1112 | 24.7673 | 29 | HN050 | HN116 |  | 1112 | 24.4955 | 28 | HN050 | HN075 |
| 1113 | 25.2351 | 29 | HN050 | HN116 |  | 1113 | 18.4287 | 23 | HN050 | HN077 |
| 1114 | 20.3727 | 23 | HN050 | HN118 |  | 1114 | 18.3227 | 23 | HN050 | HN081 |
| 1115 | 21.512 | 28 | HN050 | HN126 |  | 1115 | 22.9434 | 25 | HN050 | HN087 |
| 1116 | 27.3624 | 29 | HN050 | HN129 |  | 1116 | 20.9475 | 25 | HN050 | HN114 |
| 1117 | 27.9379 | 30 | HN050 | HN129 |  | 1117 | 22.9505 | 27 | HN050 | HN115 |
| 1118 | 22.79 | 30 | HN050 | HN129 |  | 1118 | 21.2845 | 24 | HN050 | HN117 |
| 1119 | 25.9629 | 30 | HN050 | HN129 |  | 1119 | 23.3036 | 26 | HN050 | HN118 |
| 1120 | 23.5645 | 29 | HN050 | HN130 |  | 1120 | 21.4184 | 26 | HN050 | HN123 |
| 1121 | 25.3379 | 30 | HN050 | HN130 |  | 1121 | 21.8801 | 27 | HN050 | HN124 |
| 1122 | 21.2798 | 26 | HN050 | HN131 |  | 1122 | 25.5509 | 29 | HN050 | HN125 |
| 1123 | 22.6985 | 29 | HN050 | HN131 |  | 1123 | 24.4562 | 28 | HN050 | HN127 |
| 1124 | 17.0123 | 22 | HN050 | HN139 |  | 1124 | 22.1742 | 28 | HN050 | HN128 |
| 1125 | 20.5128 | 24 | HN050 | HN147 |  | 1125 | 21.4518 | 26 | HN050 | HN128 |
| 1126 | 22.3179 | 27 | HN051 | HN054 |  | 1126 | 27.3761 | 31 | HN050 | HN131 |
| 1127 | 24.1371 | 29 | HN051 | HN055 |  | 1127 | 22.9798 | 26 | HN050 | HN137 |
| 1128 | 22.5315 | 28 | HN051 | HN057 |  | 1128 | 20.5203 | 26 | HN050 | HN138 |
| 1129 | 21.049 | 26 | HN051 | HN068 |  | 1129 | 22.8102 | 28 | HN050 | HN138 |
| 1130 | 25.1928 | 30 | HN051 | HN068 |  | 1130 | 21.8959 | 29 | HN050 | HN145 |
| 1131 | 19.578 | 22 | HN051 | HN069 |  | 1131 | 24.3746 | 30 | HN051 | HN055 |
| 1132 | 21.4612 | 24 | HN051 | HN074 |  | 1132 | 20.89 | 27 | HN051 | HN061 |
| 1133 | 21.8276 | 26 | HN051 | HN079 |  | 1133 | 24.0895 | 29 | HN051 | HN068 |
| 1134 | 27.4709 | 29 | HN051 | HN082 |  | 1134 | 22.7171 | 27 | HN051 | HN070 |
| 1135 | 24.1211 | 30 | HN051 | HN083 |  | 1135 | 24.0743 | 25 | HN051 | HN073 |
| 1136 | 24.3276 | 29 | HN051 | HN094 |  | 1136 | 21.98 | 26 | HN051 | HN080 |
| 1137 | 22.5215 | 28 | HN051 | HN098 |  | 1137 | 21.7099 | 24 | HN051 | HN084 |
| 1138 | 20.7363 | 22 | HN051 | HN106 |  | 1138 | 23.6424 | 27 | HN051 | HN096 |
| 1139 | 25.5188 | 28 | HN051 | HN111 |  | 1139 | 26.4436 | 28 | HN051 | HN097 |
| 1140 | 21.9291 | 26 | HN051 | HN115 |  | 1140 | 22.1443 | 26 | HN051 | HN098 |
| 1141 | 26.8214 | 28 | HN051 | HN116 |  | 1141 | 23.3501 | 29 | HN051 | HN102 |
| 1142 | 21.9184 | 22 | HN051 | HN117 |  | 1142 | 21.658 | 27 | HN051 | HN103 |
| 1143 | 20.6834 | 24 | HN051 | HN123 |  | 1143 | 22.0586 | 26 | HN051 | HN109 |
| 1144 | 23.5768 | 27 | HN051 | HN125 |  | 1144 | 22.7183 | 29 | HN051 | HN112 |
| 1145 | 21.2811 | 27 | HN051 | HN127 |  | 1145 | 25.6434 | 28 | HN051 | HN113 |
| 1146 | 23.9381 | 28 | HN051 | HN131 |  | 1146 | 18.8402 | 24 | HN051 | HN115 |
| 1147 | 21.0786 | 26 | HN051 | HN132 |  | 1147 | 26.935 | 30 | HN051 | HN116 |
| 1148 | 21.767 | 27 | HN051 | HN132 |  | 1148 | 24.5989 | 25 | HN051 | HN123 |
| 1149 | 22.5803 | 28 | HN051 | HN143 |  | 1149 | 20.1826 | 28 | HN051 | HN123 |
| 1150 | 23.6551 | 28 | HN051 | HN143 |  | 1150 | 22.9643 | 27 | HN051 | HN137 |
| 1151 | 18.8589 | 21 | HN051 | HN145 |  | 1151 | 20.2047 | 23 | HN051 | HN139 |
| 1152 | 21.4441 | 25 | HN051 | HN148 |  | 1152 | 21.1672 | 28 | HN051 | HN147 |
| 1153 | 21.053 | 26 | HN051 | HN150 |  | 1153 | 16.8832 | 22 | HN052 | HN055 |
| 1154 | 22.3988 | 27 | HN052 | HN060 |  | 1154 | 22.8307 | 27 | HN052 | HN065 |
| 1155 | 23.1987 | 27 | HN052 | HN065 |  | 1155 | 22.3908 | 25 | HN052 | HN093 |
| 1156 | 18.0727 | 22 | HN052 | HN070 |  | 1156 | 19.8504 | 24 | HN052 | HN094 |
| 1157 | 21.3302 | 23 | HN052 | HN074 |  | 1157 | 18.0428 | 23 | HN052 | HN107 |
| 1158 | 22.4615 | 27 | HN052 | HN075 |  | 1158 | 22.5197 | 24 | HN052 | HN116 |
| 1159 | 22.2291 | 26 | HN052 | HN075 |  | 1159 | 24.6269 | 26 | HN052 | HN116 |
| 1160 | 19.0331 | 25 | HN052 | HN077 |  | 1160 | 23.7793 | 27 | HN052 | HN116 |
| 1161 | 20.3705 | 25 | HN052 | HN111 |  | 1161 | 22.3617 | 26 | HN052 | HN123 |
| 1162 | 17.8335 | 24 | HN052 | HN112 |  | 1162 | 19.5291 | 23 | HN052 | HN123 |
| 1163 | 24.3541 | 26 | HN052 | HN116 |  | 1163 | 22.2889 | 26 | HN052 | HN125 |
| 1164 | 16.4947 | 22 | HN052 | HN128 |  | 1164 | 19.8866 | 24 | HN052 | HN129 |
| 1165 | 19.9185 | 25 | HN052 | HN138 |  | 1165 | 21.3593 | 25 | HN053 | HN055 |
| 1166 | 23.1044 | 26 | HN052 | HN140 |  | 1166 | 15.8324 | 20 | HN053 | HN055 |
| 1167 | 20.6308 | 24 | HN053 | HN059 |  | 1167 | 19.8686 | 25 | HN053 | HN059 |
| 1168 | 23.8371 | 26 | HN053 | HN064 |  | 1168 | 23.3557 | 28 | HN053 | HN065 |
| 1169 | 20.4023 | 26 | HN053 | HN065 |  | 1169 | 24.1161 | 28 | HN053 | HN068 |
| 1170 | 23.1982 | 25 | HN053 | HN070 |  | 1170 | 24.6933 | 28 | HN053 | HN072 |
| 1171 | 19.4181 | 26 | HN053 | HN072 |  | 1171 | 22.8167 | 24 | HN053 | HN073 |
| 1172 | 19.5051 | 23 | HN053 | HN107 |  | 1172 | 24.2063 | 25 | HN053 | HN082 |
| 1173 | 25.8023 | 26 | HN053 | HN113 |  | 1173 | 17.5425 | 22 | HN053 | HN088 |
| 1174 | 23.8866 | 24 | HN053 | HN116 |  | 1174 | 24.6048 | 30 | HN053 | HN090 |
| 1175 | 19.4566 | 25 | HN053 | HN129 |  | 1175 | 19.1789 | 19 | HN053 | HN097 |
| 1176 | 22.7716 | 28 | HN053 | HN131 |  | 1176 | 17.422 | 24 | HN053 | HN098 |
| 1177 | 19.1212 | 23 | HN053 | HN134 |  | 1177 | 22.173 | 24 | HN053 | HN101 |
| 1178 | 20.6176 | 25 | HN053 | HN143 |  | 1178 | 25.6114 | 30 | HN053 | HN103 |
| 1179 | 20.9009 | 20 | HN053 | HN148 |  | 1179 | 24.1878 | 27 | HN053 | HN116 |
| 1180 | 21.925 | 27 | HN054 | HN055 |  | 1180 | 22.8228 | 22 | HN053 | HN117 |
| 1181 | 20.1047 | 25 | HN054 | HN060 |  | 1181 | 18.3703 | 24 | HN053 | HN121 |
| 1182 | 23.1036 | 26 | HN054 | HN064 |  | 1182 | 20.0034 | 25 | HN053 | HN122 |
| 1183 | 22.0259 | 26 | HN054 | HN067 |  | 1183 | 19.3503 | 24 | HN053 | HN125 |
| 1184 | 23.1804 | 26 | HN054 | HN073 |  | 1184 | 18.9588 | 26 | HN053 | HN126 |
| 1185 | 26.5351 | 30 | HN054 | HN082 |  | 1185 | 22.8445 | 26 | HN053 | HN134 |
| 1186 | 21.3103 | 23 | HN054 | HN087 |  | 1186 | 21.8627 | 22 | HN053 | HN137 |
| 1187 | 24.2565 | 29 | HN054 | HN096 |  | 1187 | 21.6217 | 24 | HN053 | HN143 |
| 1188 | 25.1919 | 28 | HN054 | HN096 |  | 1188 | 18.4263 | 22 | HN053 | HN145 |
| 1189 | 22.4482 | 22 | HN054 | HN097 |  | 1189 | 20.5565 | 28 | HN054 | HN072 |
| 1190 | 21.4301 | 27 | HN054 | HN103 |  | 1190 | 24.8779 | 28 | HN054 | HN082 |
| 1191 | 21.3843 | 25 | HN054 | HN108 |  | 1191 | 22.6507 | 26 | HN054 | HN090 |
| 1192 | 20.4207 | 24 | HN054 | HN115 |  | 1192 | 23.8884 | 28 | HN054 | HN090 |
| 1193 | 18.4568 | 24 | HN054 | HN117 |  | 1193 | 26.2592 | 31 | HN054 | HN096 |
| 1194 | 21.7024 | 25 | HN054 | HN117 |  | 1194 | 25.3897 | 25 | HN054 | HN097 |
| 1195 | 23.6494 | 25 | HN054 | HN118 |  | 1195 | 23.9723 | 23 | HN054 | HN111 |
| 1196 | 19.2005 | 23 | HN054 | HN124 |  | 1196 | 22.446 | 24 | HN054 | HN116 |
| 1197 | 24.9424 | 31 | HN054 | HN129 |  | 1197 | 19.7565 | 24 | HN054 | HN126 |
| 1198 | 27.2101 | 31 | HN054 | HN129 |  | 1198 | 24.1565 | 29 | HN054 | HN131 |
| 1199 | 19.3095 | 20 | HN054 | HN140 |  | 1199 | 21.1009 | 26 | HN054 | HN132 |
| 1200 | 20.2757 | 22 | HN055 | HN061 |  | 1200 | 21.8176 | 26 | HN054 | HN148 |
| 1201 | 24.3151 | 27 | HN055 | HN061 |  | 1201 | 23.2509 | 29 | HN055 | HN056 |
| 1202 | 26.7384 | 29 | HN055 | HN068 |  | 1202 | 25.0096 | 30 | HN055 | HN061 |
| 1203 | 22.4156 | 25 | HN055 | HN069 |  | 1203 | 26.1317 | 29 | HN055 | HN065 |
| 1204 | 21.9758 | 29 | HN055 | HN072 |  | 1204 | 26.3678 | 32 | HN055 | HN069 |
| 1205 | 19.4028 | 20 | HN055 | HN077 |  | 1205 | 20.0098 | 23 | HN055 | HN069 |
| 1206 | 23.2992 | 26 | HN055 | HN080 |  | 1206 | 24.5021 | 27 | HN055 | HN072 |
| 1207 | 16.8717 | 25 | HN055 | HN088 |  | 1207 | 25.767 | 29 | HN055 | HN073 |
| 1208 | 18.2812 | 23 | HN055 | HN091 |  | 1208 | 23.0315 | 30 | HN055 | HN083 |
| 1209 | 22.7817 | 27 | HN055 | HN094 |  | 1209 | 21.639 | 21 | HN055 | HN097 |
| 1210 | 24.045 | 31 | HN055 | HN094 |  | 1210 | 22.6685 | 25 | HN055 | HN097 |
| 1211 | 22.8475 | 29 | HN055 | HN096 |  | 1211 | 24.6047 | 27 | HN055 | HN113 |
| 1212 | 22.9996 | 28 | HN055 | HN103 |  | 1212 | 25.4591 | 27 | HN055 | HN116 |
| 1213 | 19.7181 | 27 | HN055 | HN109 |  | 1213 | 24.1259 | 30 | HN055 | HN117 |
| 1214 | 26.3576 | 26 | HN055 | HN111 |  | 1214 | 19.179 | 23 | HN055 | HN135 |
| 1215 | 21.5471 | 26 | HN055 | HN114 |  | 1215 | 24.1856 | 25 | HN055 | HN137 |
| 1216 | 27.2208 | 31 | HN055 | HN116 |  | 1216 | 21.3538 | 20 | HN055 | HN142 |
| 1217 | 23.1513 | 29 | HN055 | HN116 |  | 1217 | 19.4982 | 26 | HN056 | HN061 |
| 1218 | 27.3596 | 32 | HN055 | HN116 |  | 1218 | 18.0709 | 24 | HN056 | HN062 |
| 1219 | 23.0146 | 27 | HN055 | HN116 |  | 1219 | 26.3087 | 29 | HN056 | HN073 |
| 1220 | 20.2136 | 24 | HN055 | HN124 |  | 1220 | 25.1369 | 28 | HN056 | HN082 |
| 1221 | 22.5199 | 28 | HN055 | HN128 |  | 1221 | 25.5462 | 29 | HN056 | HN090 |
| 1222 | 18.1046 | 28 | HN055 | HN131 |  | 1222 | 22.831 | 24 | HN056 | HN093 |
| 1223 | 21.7239 | 26 | HN055 | HN132 |  | 1223 | 20.2338 | 14 | HN056 | HN106 |
| 1224 | 22.3121 | 27 | HN055 | HN136 |  | 1224 | 20.5481 | 22 | HN056 | HN114 |
| 1225 | 21.9239 | 22 | HN056 | HN061 |  | 1225 | 20.935 | 25 | HN056 | HN118 |
| 1226 | 21.4339 | 26 | HN056 | HN064 |  | 1226 | 20.6583 | 27 | HN056 | HN127 |
| 1227 | 24.7125 | 26 | HN056 | HN082 |  | 1227 | 21.0846 | 25 | HN056 | HN127 |
| 1228 | 23.7123 | 29 | HN056 | HN083 |  | 1228 | 22.0282 | 25 | HN057 | HN062 |
| 1229 | 25.92 | 29 | HN056 | HN090 |  | 1229 | 22.9105 | 26 | HN057 | HN073 |
| 1230 | 23.7725 | 26 | HN056 | HN090 |  | 1230 | 22.4348 | 29 | HN057 | HN083 |
| 1231 | 24.0105 | 29 | HN056 | HN105 |  | 1231 | 21.8884 | 28 | HN057 | HN083 |
| 1232 | 26.0895 | 28 | HN056 | HN113 |  | 1232 | 21.6451 | 24 | HN057 | HN085 |
| 1233 | 23.4741 | 25 | HN056 | HN113 |  | 1233 | 20.9361 | 21 | HN057 | HN092 |
| 1234 | 16.6559 | 24 | HN056 | HN124 |  | 1234 | 23.4862 | 27 | HN057 | HN096 |
| 1235 | 21.0409 | 26 | HN056 | HN125 |  | 1235 | 24.3793 | 26 | HN057 | HN116 |
| 1236 | 20.4513 | 26 | HN056 | HN129 |  | 1236 | 23.0827 | 28 | HN057 | HN125 |
| 1237 | 22.7607 | 28 | HN056 | HN129 |  | 1237 | 18.1689 | 24 | HN057 | HN136 |
| 1238 | 21.6563 | 24 | HN056 | HN137 |  | 1238 | 19.8464 | 26 | HN057 | HN138 |
| 1239 | 18.119 | 24 | HN056 | HN138 |  | 1239 | 20.7953 | 25 | HN057 | HN144 |
| 1240 | 20.9272 | 27 | HN057 | HN065 |  | 1240 | 18.2194 | 26 | HN057 | HN147 |
| 1241 | 25.5376 | 23 | HN057 | HN068 |  | 1241 | 20.2141 | 23 | HN058 | HN059 |
| 1242 | 20.5676 | 22 | HN057 | HN072 |  | 1242 | 21.9138 | 25 | HN058 | HN065 |
| 1243 | 20.3423 | 23 | HN057 | HN077 |  | 1243 | 23.4601 | 27 | HN058 | HN065 |
| 1244 | 19.9537 | 24 | HN057 | HN079 |  | 1244 | 20.279 | 24 | HN058 | HN071 |
| 1245 | 23.1841 | 23 | HN057 | HN082 |  | 1245 | 20.1068 | 24 | HN058 | HN074 |
| 1246 | 22.0137 | 24 | HN057 | HN085 |  | 1246 | 22.2922 | 22 | HN058 | HN075 |
| 1247 | 24.101 | 25 | HN057 | HN093 |  | 1247 | 23.7132 | 24 | HN058 | HN082 |
| 1248 | 25.7345 | 30 | HN057 | HN096 |  | 1248 | 27.3177 | 27 | HN058 | HN082 |
| 1249 | 22.7992 | 26 | HN057 | HN109 |  | 1249 | 22.2174 | 27 | HN058 | HN083 |
| 1250 | 23.7236 | 25 | HN057 | HN118 |  | 1250 | 21.5329 | 26 | HN058 | HN084 |
| 1251 | 17.0171 | 22 | HN057 | HN119 |  | 1251 | 21.8244 | 23 | HN058 | HN093 |
| 1252 | 21.7459 | 25 | HN057 | HN124 |  | 1252 | 21.0933 | 22 | HN058 | HN093 |
| 1253 | 24.0746 | 28 | HN057 | HN131 |  | 1253 | 21.4666 | 25 | HN058 | HN102 |
| 1254 | 20.5592 | 24 | HN057 | HN134 |  | 1254 | 23.8505 | 26 | HN058 | HN103 |
| 1255 | 17.5718 | 24 | HN057 | HN147 |  | 1255 | 22.0147 | 25 | HN058 | HN103 |
| 1256 | 22.3954 | 26 | HN058 | HN064 |  | 1256 | 21.6942 | 21 | HN058 | HN104 |
| 1257 | 19.5857 | 23 | HN058 | HN065 |  | 1257 | 20.4299 | 25 | HN058 | HN109 |
| 1258 | 23.9246 | 25 | HN058 | HN070 |  | 1258 | 23.6426 | 24 | HN058 | HN111 |
| 1259 | 17.8984 | 19 | HN058 | HN076 |  | 1259 | 24.2227 | 23 | HN058 | HN116 |
| 1260 | 20.7628 | 28 | HN058 | HN083 |  | 1260 | 22.9808 | 27 | HN058 | HN118 |
| 1261 | 20.2738 | 21 | HN058 | HN084 |  | 1261 | 20.2572 | 25 | HN058 | HN123 |
| 1262 | 24.3915 | 25 | HN058 | HN084 |  | 1262 | 24.3732 | 27 | HN058 | HN125 |
| 1263 | 23.474 | 23 | HN058 | HN113 |  | 1263 | 22.0793 | 28 | HN058 | HN133 |
| 1264 | 26.6634 | 27 | HN058 | HN116 |  | 1264 | 21.4199 | 22 | HN058 | HN135 |
| 1265 | 21.0654 | 23 | HN058 | HN120 |  | 1265 | 21.3937 | 22 | HN058 | HN135 |
| 1266 | 23.3947 | 23 | HN058 | HN122 |  | 1266 | 21.5296 | 22 | HN058 | HN149 |
| 1267 | 22.2929 | 23 | HN058 | HN122 |  | 1267 | 21.0553 | 21 | HN058 | HN150 |
| 1268 | 19.2729 | 24 | HN058 | HN123 |  | 1268 | 21.8678 | 27 | HN059 | HN064 |
| 1269 | 21.7444 | 25 | HN058 | HN125 |  | 1269 | 24.2699 | 25 | HN059 | HN082 |
| 1270 | 24.1323 | 29 | HN058 | HN127 |  | 1270 | 17.7919 | 20 | HN059 | HN084 |
| 1271 | 22.2359 | 22 | HN058 | HN130 |  | 1271 | 16.8058 | 21 | HN059 | HN091 |
| 1272 | 23.031 | 25 | HN058 | HN137 |  | 1272 | 25.2965 | 30 | HN059 | HN096 |
| 1273 | 23.471 | 26 | HN058 | HN138 |  | 1273 | 19.2287 | 23 | HN059 | HN145 |
| 1274 | 24.3557 | 26 | HN058 | HN138 |  | 1274 | 25.3877 | 26 | HN060 | HN066 |
| 1275 | 18.2209 | 28 | HN059 | HN072 |  | 1275 | 22.5825 | 26 | HN060 | HN068 |
| 1276 | 15.3881 | 20 | HN059 | HN074 |  | 1276 | 22.7186 | 26 | HN060 | HN076 |
| 1277 | 21.89 | 24 | HN059 | HN075 |  | 1277 | 25.6424 | 31 | HN060 | HN079 |
| 1278 | 16.9241 | 23 | HN059 | HN075 |  | 1278 | 28.538 | 29 | HN060 | HN082 |
| 1279 | 24.1222 | 29 | HN059 | HN090 |  | 1279 | 26.6165 | 33 | HN060 | HN083 |
| 1280 | 20.5258 | 25 | HN059 | HN107 |  | 1280 | 25.7595 | 32 | HN060 | HN083 |
| 1281 | 20.6452 | 23 | HN059 | HN116 |  | 1281 | 24.0234 | 29 | HN060 | HN112 |
| 1282 | 18.6049 | 22 | HN059 | HN123 |  | 1282 | 20.1364 | 28 | HN060 | HN126 |
| 1283 | 16.7428 | 22 | HN059 | HN133 |  | 1283 | 22.5634 | 26 | HN060 | HN127 |
| 1284 | 17.6607 | 22 | HN059 | HN133 |  | 1284 | 22.3787 | 28 | HN060 | HN130 |
| 1285 | 24.7634 | 28 | HN060 | HN061 |  | 1285 | 23.5776 | 28 | HN060 | HN136 |
| 1286 | 24.6183 | 27 | HN060 | HN064 |  | 1286 | 23.615 | 28 | HN060 | HN137 |
| 1287 | 22.4915 | 27 | HN060 | HN067 |  | 1287 | 21.8132 | 27 | HN060 | HN138 |
| 1288 | 20.7975 | 28 | HN060 | HN072 |  | 1288 | 23.7641 | 29 | HN060 | HN147 |
| 1289 | 22.986 | 24 | HN060 | HN080 |  | 1289 | 22.1743 | 24 | HN060 | HN149 |
| 1290 | 20.6381 | 24 | HN060 | HN081 |  | 1290 | 25.7105 | 28 | HN061 | HN062 |
| 1291 | 23.0663 | 27 | HN060 | HN102 |  | 1291 | 24.046 | 29 | HN061 | HN063 |
| 1292 | 25.1603 | 30 | HN060 | HN103 |  | 1292 | 21.4784 | 24 | HN061 | HN067 |
| 1293 | 25.636 | 26 | HN060 | HN104 |  | 1293 | 24.0441 | 24 | HN061 | HN070 |
| 1294 | 25.6399 | 27 | HN060 | HN104 |  | 1294 | 22.0091 | 23 | HN061 | HN077 |
| 1295 | 22.4131 | 23 | HN060 | HN106 |  | 1295 | 22.2959 | 24 | HN061 | HN080 |
| 1296 | 20.7279 | 26 | HN060 | HN114 |  | 1296 | 26.5005 | 29 | HN061 | HN083 |
| 1297 | 22.2022 | 26 | HN060 | HN127 |  | 1297 | 24.1791 | 26 | HN061 | HN085 |
| 1298 | 26.3024 | 32 | HN060 | HN129 |  | 1298 | 22.3133 | 22 | HN061 | HN091 |
| 1299 | 24.2884 | 30 | HN060 | HN130 |  | 1299 | 19.8611 | 20 | HN061 | HN092 |
| 1300 | 26.3499 | 29 | HN060 | HN131 |  | 1300 | 22.7823 | 24 | HN061 | HN093 |
| 1301 | 26.0709 | 30 | HN060 | HN131 |  | 1301 | 24.6725 | 28 | HN061 | HN094 |
| 1302 | 23.4206 | 27 | HN060 | HN132 |  | 1302 | 23.7807 | 22 | HN061 | HN097 |
| 1303 | 22.835 | 27 | HN060 | HN134 |  | 1303 | 25.5049 | 23 | HN061 | HN097 |
| 1304 | 14.5591 | 21 | HN060 | HN139 |  | 1304 | 24.9358 | 31 | HN061 | HN103 |
| 1305 | 19.9619 | 23 | HN060 | HN144 |  | 1305 | 24.5084 | 23 | HN061 | HN104 |
| 1306 | 20.2341 | 27 | HN060 | HN145 |  | 1306 | 22.7754 | 24 | HN061 | HN110 |
| 1307 | 22.7053 | 24 | HN061 | HN070 |  | 1307 | 21.0406 | 25 | HN061 | HN112 |
| 1308 | 23.5164 | 26 | HN061 | HN070 |  | 1308 | 21.6218 | 22 | HN061 | HN117 |
| 1309 | 23.8543 | 27 | HN061 | HN074 |  | 1309 | 22.8289 | 25 | HN061 | HN118 |
| 1310 | 23.3186 | 24 | HN061 | HN075 |  | 1310 | 26.8785 | 31 | HN061 | HN129 |
| 1311 | 21.9052 | 24 | HN061 | HN076 |  | 1311 | 26.2741 | 32 | HN061 | HN129 |
| 1312 | 22.6233 | 27 | HN061 | HN083 |  | 1312 | 22.4985 | 26 | HN061 | HN137 |
| 1313 | 18.9876 | 20 | HN061 | HN084 |  | 1313 | 21.3617 | 22 | HN061 | HN145 |
| 1314 | 23.8599 | 27 | HN061 | HN085 |  | 1314 | 21.8881 | 22 | HN061 | HN148 |
| 1315 | 23.8599 | 27 | HN061 | HN085 |  | 1315 | 21.7138 | 26 | HN062 | HN065 |
| 1316 | 25.0364 | 30 | HN061 | HN094 |  | 1316 | 22.2677 | 24 | HN062 | HN066 |
| 1317 | 19.767 | 24 | HN061 | HN094 |  | 1317 | 21.861 | 26 | HN062 | HN068 |
| 1318 | 23.9287 | 23 | HN061 | HN103 |  | 1318 | 22.1381 | 25 | HN062 | HN080 |
| 1319 | 26.2155 | 28 | HN061 | HN103 |  | 1319 | 25.6046 | 29 | HN062 | HN082 |
| 1320 | 23.5095 | 21 | HN061 | HN104 |  | 1320 | 15.1488 | 20 | HN062 | HN092 |
| 1321 | 21.012 | 21 | HN061 | HN104 |  | 1321 | 19.6132 | 22 | HN062 | HN103 |
| 1322 | 24.2166 | 20 | HN061 | HN106 |  | 1322 | 22.2713 | 27 | HN062 | HN105 |
| 1323 | 28.6447 | 29 | HN061 | HN111 |  | 1323 | 23.8821 | 28 | HN062 | HN105 |
| 1324 | 24.0688 | 24 | HN061 | HN113 |  | 1324 | 21.4385 | 25 | HN062 | HN109 |
| 1325 | 24.6485 | 26 | HN061 | HN113 |  | 1325 | 19.7216 | 24 | HN062 | HN116 |
| 1326 | 23.7102 | 24 | HN061 | HN114 |  | 1326 | 25.3886 | 27 | HN062 | HN118 |
| 1327 | 23.5888 | 27 | HN061 | HN115 |  | 1327 | 18.4917 | 21 | HN062 | HN119 |
| 1328 | 20.1906 | 26 | HN061 | HN119 |  | 1328 | 22.4859 | 25 | HN062 | HN132 |
| 1329 | 17.5664 | 22 | HN061 | HN122 |  | 1329 | 19.8398 | 23 | HN062 | HN135 |
| 1330 | 26.5543 | 29 | HN061 | HN125 |  | 1330 | 22.7617 | 24 | HN062 | HN148 |
| 1331 | 21.2574 | 27 | HN061 | HN127 |  | 1331 | 20.1482 | 26 | HN063 | HN065 |
| 1332 | 21.6393 | 23 | HN061 | HN128 |  | 1332 | 20.3561 | 25 | HN063 | HN072 |
| 1333 | 23.1298 | 28 | HN061 | HN129 |  | 1333 | 23.2645 | 31 | HN063 | HN083 |
| 1334 | 21.9632 | 28 | HN061 | HN129 |  | 1334 | 22.6019 | 27 | HN063 | HN108 |
| 1335 | 22.7095 | 28 | HN061 | HN130 |  | 1335 | 25.8559 | 28 | HN063 | HN116 |
| 1336 | 24.2397 | 27 | HN061 | HN138 |  | 1336 | 25.1299 | 30 | HN063 | HN118 |
| 1337 | 21.2662 | 24 | HN062 | HN069 |  | 1337 | 19.2521 | 22 | HN063 | HN124 |
| 1338 | 25.2866 | 28 | HN062 | HN073 |  | 1338 | 17.6443 | 22 | HN063 | HN135 |
| 1339 | 16.311 | 22 | HN062 | HN081 |  | 1339 | 19.3561 | 23 | HN063 | HN141 |
| 1340 | 24.703 | 28 | HN062 | HN094 |  | 1340 | 20.5062 | 25 | HN063 | HN146 |
| 1341 | 20.0407 | 24 | HN062 | HN094 |  | 1341 | 22.9348 | 28 | HN063 | HN149 |
| 1342 | 22.6106 | 24 | HN062 | HN123 |  | 1342 | 21.1613 | 22 | HN064 | HN066 |
| 1343 | 20.0302 | 22 | HN062 | HN124 |  | 1343 | 21.4129 | 25 | HN064 | HN072 |
| 1344 | 20.5016 | 25 | HN062 | HN125 |  | 1344 | 26.2925 | 30 | HN064 | HN073 |
| 1345 | 22.315 | 26 | HN063 | HN073 |  | 1345 | 20.1257 | 24 | HN064 | HN074 |
| 1346 | 23.0597 | 24 | HN063 | HN082 |  | 1346 | 19.5474 | 24 | HN064 | HN081 |
| 1347 | 19.995 | 27 | HN063 | HN085 |  | 1347 | 23.605 | 24 | HN064 | HN093 |
| 1348 | 23.818 | 24 | HN063 | HN093 |  | 1348 | 22.6274 | 23 | HN064 | HN093 |
| 1349 | 18.3913 | 25 | HN063 | HN094 |  | 1349 | 20.3486 | 22 | HN064 | HN095 |
| 1350 | 20.3891 | 24 | HN063 | HN117 |  | 1350 | 23.5252 | 28 | HN064 | HN096 |
| 1351 | 21.0029 | 25 | HN063 | HN117 |  | 1351 | 21.1048 | 27 | HN064 | HN098 |
| 1352 | 19.4003 | 21 | HN063 | HN148 |  | 1352 | 26.796 | 29 | HN064 | HN103 |
| 1353 | 21.3443 | 23 | HN064 | HN067 |  | 1353 | 25.2001 | 21 | HN064 | HN104 |
| 1354 | 24.6392 | 26 | HN064 | HN073 |  | 1354 | 22.0246 | 26 | HN064 | HN105 |
| 1355 | 25.291 | 27 | HN064 | HN073 |  | 1355 | 26.3129 | 27 | HN064 | HN111 |
| 1356 | 24.5723 | 25 | HN064 | HN073 |  | 1356 | 25.4008 | 27 | HN064 | HN118 |
| 1357 | 22.4136 | 26 | HN064 | HN080 |  | 1357 | 23.8516 | 26 | HN064 | HN122 |
| 1358 | 22.1019 | 27 | HN064 | HN083 |  | 1358 | 23.3146 | 28 | HN064 | HN126 |
| 1359 | 22.6181 | 21 | HN064 | HN084 |  | 1359 | 23.3265 | 27 | HN064 | HN137 |
| 1360 | 23.2147 | 27 | HN064 | HN085 |  | 1360 | 22.874 | 26 | HN064 | HN138 |
| 1361 | 23.0834 | 26 | HN064 | HN085 |  | 1361 | 23.4634 | 25 | HN064 | HN140 |
| 1362 | 23.3234 | 25 | HN064 | HN085 |  | 1362 | 20.1654 | 26 | HN065 | HN072 |
| 1363 | 18.6966 | 21 | HN064 | HN088 |  | 1363 | 22.8196 | 25 | HN065 | HN085 |
| 1364 | 22.5564 | 26 | HN064 | HN090 |  | 1364 | 20.6377 | 22 | HN065 | HN097 |
| 1365 | 22.8681 | 27 | HN064 | HN090 |  | 1365 | 18.3286 | 24 | HN065 | HN100 |
| 1366 | 22.136 | 25 | HN064 | HN095 |  | 1366 | 20.7683 | 24 | HN065 | HN121 |
| 1367 | 22.9776 | 26 | HN064 | HN095 |  | 1367 | 21.8498 | 29 | HN065 | HN130 |
| 1368 | 24.4742 | 28 | HN064 | HN096 |  | 1368 | 25.9065 | 29 | HN065 | HN131 |
| 1369 | 20.5949 | 24 | HN064 | HN115 |  | 1369 | 20.731 | 27 | HN065 | HN134 |
| 1370 | 24.8691 | 26 | HN064 | HN116 |  | 1370 | 20.0065 | 24 | HN066 | HN067 |
| 1371 | 23.99 | 27 | HN064 | HN124 |  | 1371 | 25.6865 | 26 | HN066 | HN073 |
| 1372 | 23.3084 | 27 | HN064 | HN128 |  | 1372 | 22.6763 | 18 | HN066 | HN076 |
| 1373 | 20.336 | 23 | HN064 | HN145 |  | 1373 | 21.5117 | 22 | HN066 | HN082 |
| 1374 | 22.0071 | 25 | HN064 | HN146 |  | 1374 | 18.6788 | 20 | HN066 | HN085 |
| 1375 | 23.8255 | 30 | HN065 | HN068 |  | 1375 | 25.1016 | 24 | HN066 | HN087 |
| 1376 | 25.1109 | 30 | HN065 | HN073 |  | 1376 | 19.2416 | 22 | HN066 | HN101 |
| 1377 | 19.5735 | 23 | HN065 | HN082 |  | 1377 | 24.151 | 22 | HN066 | HN104 |
| 1378 | 17.6935 | 20 | HN065 | HN082 |  | 1378 | 22.0422 | 26 | HN066 | HN109 |
| 1379 | 20.5232 | 26 | HN065 | HN083 |  | 1379 | 25.2813 | 23 | HN066 | HN113 |
| 1380 | 21.7268 | 25 | HN065 | HN085 |  | 1380 | 25.8903 | 25 | HN066 | HN116 |
| 1381 | 21.2429 | 27 | HN065 | HN094 |  | 1381 | 23.5963 | 21 | HN066 | HN117 |
| 1382 | 25.5746 | 28 | HN065 | HN094 |  | 1382 | 18.6376 | 23 | HN066 | HN126 |
| 1383 | 25.916 | 31 | HN065 | HN096 |  | 1383 | 22.8457 | 22 | HN066 | HN128 |
| 1384 | 22.7548 | 27 | HN065 | HN101 |  | 1384 | 24.099 | 24 | HN066 | HN129 |
| 1385 | 19.4519 | 23 | HN065 | HN102 |  | 1385 | 25.9517 | 25 | HN066 | HN129 |
| 1386 | 21.513 | 24 | HN065 | HN104 |  | 1386 | 16.1609 | 19 | HN066 | HN135 |
| 1387 | 22.7384 | 23 | HN065 | HN104 |  | 1387 | 23.2783 | 24 | HN066 | HN140 |
| 1388 | 21.1608 | 23 | HN065 | HN106 |  | 1388 | 20.8974 | 24 | HN066 | HN143 |
| 1389 | 23.1182 | 28 | HN065 | HN107 |  | 1389 | 19.6488 | 23 | HN066 | HN147 |
| 1390 | 21.9221 | 25 | HN065 | HN116 |  | 1390 | 17.192 | 21 | HN066 | HN149 |
| 1391 | 14.2513 | 23 | HN065 | HN120 |  | 1391 | 22.3526 | 23 | HN066 | HN149 |
| 1392 | 20.7539 | 27 | HN065 | HN130 |  | 1392 | 22.2659 | 23 | HN067 | HN069 |
| 1393 | 24.5175 | 29 | HN065 | HN131 |  | 1393 | 16.4526 | 21 | HN067 | HN072 |
| 1394 | 21.7709 | 28 | HN065 | HN132 |  | 1394 | 23.3613 | 27 | HN067 | HN083 |
| 1395 | 26.2268 | 30 | HN065 | HN138 |  | 1395 | 20.5251 | 22 | HN067 | HN094 |
| 1396 | 24.4534 | 29 | HN065 | HN138 |  | 1396 | 24.6263 | 29 | HN067 | HN094 |
| 1397 | 21.4016 | 27 | HN065 | HN138 |  | 1397 | 24.6263 | 29 | HN067 | HN094 |
| 1398 | 24.5595 | 28 | HN065 | HN143 |  | 1398 | 23.9154 | 27 | HN067 | HN096 |
| 1399 | 22.7861 | 27 | HN065 | HN143 |  | 1399 | 25.4286 | 29 | HN067 | HN103 |
| 1400 | 20.5824 | 23 | HN065 | HN145 |  | 1400 | 21.051 | 26 | HN067 | HN107 |
| 1401 | 15.2634 | 24 | HN065 | HN147 |  | 1401 | 22.0265 | 28 | HN067 | HN109 |
| 1402 | 21.805 | 24 | HN066 | HN069 |  | 1402 | 21.9216 | 24 | HN067 | HN114 |
| 1403 | 19.395 | 25 | HN066 | HN105 |  | 1403 | 22.5776 | 25 | HN067 | HN116 |
| 1404 | 21.3955 | 22 | HN066 | HN111 |  | 1404 | 24.239 | 31 | HN067 | HN131 |
| 1405 | 21.5264 | 22 | HN066 | HN122 |  | 1405 | 22.356 | 26 | HN067 | HN138 |
| 1406 | 24.2013 | 28 | HN066 | HN129 |  | 1406 | 21.7534 | 24 | HN067 | HN138 |
| 1407 | 24.0533 | 27 | HN066 | HN129 |  | 1407 | 24.1703 | 26 | HN067 | HN140 |
| 1408 | 24.1922 | 26 | HN067 | HN068 |  | 1408 | 21.5819 | 28 | HN067 | HN143 |
| 1409 | 24.8453 | 27 | HN067 | HN073 |  | 1409 | 22.2708 | 28 | HN067 | HN147 |
| 1410 | 22.3327 | 25 | HN067 | HN075 |  | 1410 | 21.1016 | 20 | HN067 | HN149 |
| 1411 | 24.0092 | 28 | HN067 | HN080 |  | 1411 | 15.4875 | 20 | HN067 | HN150 |
| 1412 | 21.6884 | 24 | HN067 | HN081 |  | 1412 | 23.6568 | 29 | HN068 | HN069 |
| 1413 | 25.648 | 29 | HN067 | HN082 |  | 1413 | 25.2713 | 28 | HN068 | HN069 |
| 1414 | 23.3832 | 28 | HN067 | HN083 |  | 1414 | 25.2807 | 27 | HN068 | HN070 |
| 1415 | 21.1667 | 23 | HN067 | HN092 |  | 1415 | 26.3555 | 25 | HN068 | HN075 |
| 1416 | 20.9601 | 25 | HN067 | HN094 |  | 1416 | 27.9662 | 29 | HN068 | HN083 |
| 1417 | 25.6388 | 26 | HN067 | HN103 |  | 1417 | 21.2014 | 25 | HN068 | HN084 |
| 1418 | 19.3553 | 25 | HN067 | HN103 |  | 1418 | 25.2833 | 29 | HN068 | HN103 |
| 1419 | 21.7811 | 20 | HN067 | HN106 |  | 1419 | 23.5665 | 27 | HN068 | HN103 |
| 1420 | 22.7522 | 27 | HN067 | HN118 |  | 1420 | 23.7036 | 27 | HN068 | HN109 |
| 1421 | 22.6157 | 27 | HN067 | HN125 |  | 1421 | 23.2948 | 27 | HN068 | HN110 |
| 1422 | 24.3535 | 28 | HN067 | HN127 |  | 1422 | 26.1712 | 26 | HN068 | HN111 |
| 1423 | 21.8146 | 24 | HN067 | HN129 |  | 1423 | 24.6195 | 26 | HN068 | HN118 |
| 1424 | 22.6717 | 25 | HN067 | HN131 |  | 1424 | 27.5485 | 26 | HN068 | HN123 |
| 1425 | 19.1071 | 21 | HN067 | HN149 |  | 1425 | 23.0135 | 25 | HN068 | HN128 |
| 1426 | 25.3938 | 28 | HN068 | HN073 |  | 1426 | 23.0113 | 28 | HN068 | HN130 |
| 1427 | 26.4508 | 27 | HN068 | HN073 |  | 1427 | 20.8901 | 24 | HN068 | HN134 |
| 1428 | 23.7575 | 27 | HN068 | HN075 |  | 1428 | 26.8704 | 25 | HN068 | HN139 |
| 1429 | 22.5427 | 24 | HN068 | HN075 |  | 1429 | 22.3348 | 24 | HN068 | HN140 |
| 1430 | 24.6766 | 27 | HN068 | HN077 |  | 1430 | 24.4331 | 27 | HN068 | HN143 |
| 1431 | 24.601 | 29 | HN068 | HN079 |  | 1431 | 21.4396 | 23 | HN069 | HN072 |
| 1432 | 21.7057 | 21 | HN068 | HN080 |  | 1432 | 23.5477 | 29 | HN069 | HN083 |
| 1433 | 24.0082 | 25 | HN068 | HN082 |  | 1433 | 25.4892 | 30 | HN069 | HN083 |
| 1434 | 26.8428 | 32 | HN068 | HN083 |  | 1434 | 17.644 | 20 | HN069 | HN091 |
| 1435 | 29.279 | 33 | HN068 | HN083 |  | 1435 | 23.4697 | 26 | HN069 | HN107 |
| 1436 | 21.7719 | 25 | HN068 | HN083 |  | 1436 | 21.1046 | 23 | HN069 | HN109 |
| 1437 | 18.2112 | 22 | HN068 | HN094 |  | 1437 | 22.7043 | 26 | HN069 | HN118 |
| 1438 | 24.9216 | 26 | HN068 | HN094 |  | 1438 | 20.41 | 24 | HN069 | HN121 |
| 1439 | 27.6081 | 28 | HN068 | HN096 |  | 1439 | 21.1136 | 23 | HN069 | HN123 |
| 1440 | 22.0878 | 27 | HN068 | HN101 |  | 1440 | 23.4013 | 26 | HN069 | HN125 |
| 1441 | 24.1825 | 27 | HN068 | HN103 |  | 1441 | 27.9199 | 33 | HN069 | HN131 |
| 1442 | 22.7713 | 23 | HN068 | HN104 |  | 1442 | 24.2805 | 26 | HN069 | HN138 |
| 1443 | 21.8624 | 20 | HN068 | HN106 |  | 1443 | 19.4513 | 24 | HN069 | HN142 |
| 1444 | 22.9209 | 28 | HN068 | HN109 |  | 1444 | 21.4853 | 24 | HN069 | HN148 |
| 1445 | 21.9924 | 25 | HN068 | HN116 |  | 1445 | 23.0646 | 26 | HN070 | HN075 |
| 1446 | 24.0136 | 28 | HN068 | HN116 |  | 1446 | 25.421 | 29 | HN070 | HN083 |
| 1447 | 23.1534 | 26 | HN068 | HN118 |  | 1447 | 25.3638 | 31 | HN070 | HN096 |
| 1448 | 22.8121 | 23 | HN068 | HN118 |  | 1448 | 20.5651 | 20 | HN070 | HN097 |
| 1449 | 25.2517 | 30 | HN068 | HN118 |  | 1449 | 21.6956 | 24 | HN070 | HN101 |
| 1450 | 20.2683 | 24 | HN068 | HN120 |  | 1450 | 19.6546 | 21 | HN070 | HN104 |
| 1451 | 24.4824 | 28 | HN068 | HN121 |  | 1451 | 21.8306 | 29 | HN070 | HN105 |
| 1452 | 25.2971 | 27 | HN068 | HN124 |  | 1452 | 20.1666 | 19 | HN070 | HN113 |
| 1453 | 23.2696 | 27 | HN068 | HN125 |  | 1453 | 21.1743 | 20 | HN070 | HN113 |
| 1454 | 26.246 | 31 | HN068 | HN127 |  | 1454 | 21.7193 | 21 | HN070 | HN116 |
| 1455 | 24.0904 | 27 | HN068 | HN137 |  | 1455 | 16.6816 | 21 | HN070 | HN119 |
| 1456 | 27.2052 | 29 | HN068 | HN139 |  | 1456 | 25.8027 | 30 | HN070 | HN129 |
| 1457 | 21.1277 | 26 | HN068 | HN144 |  | 1457 | 26.896 | 31 | HN070 | HN131 |
| 1458 | 24.2574 | 26 | HN068 | HN146 |  | 1458 | 28.058 | 31 | HN070 | HN131 |
| 1459 | 21.4682 | 22 | HN068 | HN149 |  | 1459 | 27.0528 | 29 | HN070 | HN131 |
| 1460 | 18.8831 | 20 | HN069 | HN070 |  | 1460 | 16.7827 | 17 | HN070 | HN134 |
| 1461 | 25.8144 | 29 | HN069 | HN073 |  | 1461 | 17.3383 | 22 | HN071 | HN075 |
| 1462 | 24.3216 | 26 | HN069 | HN075 |  | 1462 | 22.2129 | 23 | HN071 | HN080 |
| 1463 | 21.0303 | 24 | HN069 | HN080 |  | 1463 | 23.956 | 29 | HN071 | HN083 |
| 1464 | 23.8866 | 28 | HN069 | HN082 |  | 1464 | 19.9448 | 23 | HN071 | HN122 |
| 1465 | 23.6487 | 27 | HN069 | HN083 |  | 1465 | 18.9986 | 27 | HN071 | HN125 |
| 1466 | 23.738 | 26 | HN069 | HN083 |  | 1466 | 19.6982 | 23 | HN071 | HN135 |
| 1467 | 18.8835 | 22 | HN069 | HN087 |  | 1467 | 22.3827 | 26 | HN071 | HN143 |
| 1468 | 24.059 | 31 | HN069 | HN096 |  | 1468 | 26.2707 | 27 | HN072 | HN073 |
| 1469 | 23.5038 | 25 | HN069 | HN104 |  | 1469 | 24.2854 | 28 | HN072 | HN079 |
| 1470 | 22.7958 | 28 | HN069 | HN109 |  | 1470 | 21.0942 | 26 | HN072 | HN093 |
| 1471 | 23.0481 | 28 | HN069 | HN112 |  | 1471 | 24.0436 | 29 | HN072 | HN107 |
| 1472 | 21.9328 | 22 | HN069 | HN113 |  | 1472 | 20.9337 | 23 | HN072 | HN110 |
| 1473 | 25.4582 | 31 | HN069 | HN130 |  | 1473 | 22.1682 | 28 | HN072 | HN111 |
| 1474 | 21.3509 | 24 | HN069 | HN137 |  | 1474 | 24.6522 | 26 | HN072 | HN113 |
| 1475 | 22.32 | 27 | HN069 | HN140 |  | 1475 | 21.945 | 26 | HN072 | HN115 |
| 1476 | 21.9326 | 23 | HN069 | HN141 |  | 1476 | 23.368 | 26 | HN072 | HN116 |
| 1477 | 22.8044 | 26 | HN069 | HN149 |  | 1477 | 20.759 | 23 | HN072 | HN117 |
| 1478 | 19.9584 | 19 | HN070 | HN085 |  | 1478 | 21.6798 | 21 | HN072 | HN118 |
| 1479 | 27.7356 | 29 | HN070 | HN096 |  | 1479 | 24.2954 | 29 | HN072 | HN130 |
| 1480 | 19.8622 | 25 | HN070 | HN103 |  | 1480 | 21.2838 | 26 | HN072 | HN130 |
| 1481 | 19.6492 | 26 | HN070 | HN103 |  | 1481 | 20.8057 | 26 | HN072 | HN137 |
| 1482 | 20.6786 | 24 | HN070 | HN109 |  | 1482 | 20.8694 | 24 | HN072 | HN140 |
| 1483 | 26.9951 | 28 | HN070 | HN131 |  | 1483 | 23.3508 | 24 | HN073 | HN074 |
| 1484 | 23.4111 | 25 | HN070 | HN143 |  | 1484 | 21.2351 | 24 | HN073 | HN076 |
| 1485 | 16.2173 | 20 | HN070 | HN144 |  | 1485 | 24.0663 | 29 | HN073 | HN077 |
| 1486 | 20.7341 | 27 | HN071 | HN083 |  | 1486 | 19.2115 | 23 | HN073 | HN079 |
| 1487 | 23.1096 | 30 | HN071 | HN083 |  | 1487 | 23.8248 | 25 | HN073 | HN080 |
| 1488 | 24.8965 | 27 | HN071 | HN118 |  | 1488 | 21.1166 | 25 | HN073 | HN081 |
| 1489 | 18.8989 | 23 | HN071 | HN122 |  | 1489 | 24.6465 | 24 | HN073 | HN081 |
| 1490 | 21.1274 | 24 | HN071 | HN135 |  | 1490 | 26.4639 | 27 | HN073 | HN082 |
| 1491 | 22.7326 | 28 | HN071 | HN137 |  | 1491 | 26.7341 | 31 | HN073 | HN083 |
| 1492 | 23.1154 | 29 | HN072 | HN075 |  | 1492 | 24.7485 | 28 | HN073 | HN090 |
| 1493 | 22.977 | 28 | HN072 | HN075 |  | 1493 | 23.9393 | 26 | HN073 | HN091 |
| 1494 | 18.9857 | 20 | HN072 | HN077 |  | 1494 | 24.5533 | 25 | HN073 | HN093 |
| 1495 | 24.1058 | 26 | HN072 | HN079 |  | 1495 | 21.9463 | 23 | HN073 | HN093 |
| 1496 | 21.0141 | 25 | HN072 | HN080 |  | 1496 | 23.7616 | 24 | HN073 | HN093 |
| 1497 | 19.2792 | 23 | HN072 | HN081 |  | 1497 | 22.2911 | 28 | HN073 | HN096 |
| 1498 | 26.0089 | 26 | HN072 | HN082 |  | 1498 | 21.4876 | 25 | HN073 | HN098 |
| 1499 | 24.488 | 26 | HN072 | HN082 |  | 1499 | 24.2868 | 26 | HN073 | HN098 |
| 1500 | 25.1146 | 29 | HN072 | HN083 |  | 1500 | 26.8657 | 28 | HN073 | HN103 |
| 1501 | 21.4052 | 27 | HN072 | HN090 |  | 1501 | 26.4176 | 28 | HN073 | HN104 |
| 1502 | 19.6582 | 24 | HN072 | HN093 |  | 1502 | 24.6801 | 25 | HN073 | HN104 |
| 1503 | 24.0573 | 27 | HN072 | HN094 |  | 1503 | 28.0666 | 32 | HN073 | HN105 |
| 1504 | 24.5918 | 29 | HN072 | HN096 |  | 1504 | 27.0521 | 32 | HN073 | HN105 |
| 1505 | 20.1866 | 21 | HN072 | HN097 |  | 1505 | 23.4566 | 19 | HN073 | HN106 |
| 1506 | 23.4268 | 29 | HN072 | HN103 |  | 1506 | 23.8752 | 25 | HN073 | HN107 |
| 1507 | 16.9165 | 26 | HN072 | HN105 |  | 1507 | 23.6224 | 28 | HN073 | HN108 |
| 1508 | 20.7569 | 25 | HN072 | HN124 |  | 1508 | 22.6995 | 23 | HN073 | HN108 |
| 1509 | 25.8042 | 28 | HN072 | HN129 |  | 1509 | 23.2473 | 25 | HN073 | HN109 |
| 1510 | 21.9189 | 28 | HN072 | HN138 |  | 1510 | 28.9296 | 30 | HN073 | HN113 |
| 1511 | 18.8344 | 21 | HN072 | HN140 |  | 1511 | 28.5143 | 29 | HN073 | HN113 |
| 1512 | 20.4979 | 23 | HN072 | HN141 |  | 1512 | 28.3903 | 26 | HN073 | HN113 |
| 1513 | 23.4234 | 26 | HN072 | HN144 |  | 1513 | 25.1519 | 30 | HN073 | HN114 |
| 1514 | 24.1492 | 28 | HN072 | HN144 |  | 1514 | 22.7657 | 26 | HN073 | HN115 |
| 1515 | 20.1686 | 21 | HN072 | HN148 |  | 1515 | 28.341 | 32 | HN073 | HN116 |
| 1516 | 22.998 | 27 | HN073 | HN074 |  | 1516 | 23.1713 | 22 | HN073 | HN117 |
| 1517 | 21.513 | 23 | HN073 | HN074 |  | 1517 | 25.3688 | 28 | HN073 | HN120 |
| 1518 | 24.4097 | 27 | HN073 | HN079 |  | 1518 | 25.4157 | 26 | HN073 | HN123 |
| 1519 | 23.3845 | 23 | HN073 | HN080 |  | 1519 | 24.5867 | 27 | HN073 | HN127 |
| 1520 | 22.2153 | 23 | HN073 | HN081 |  | 1520 | 21.0727 | 24 | HN073 | HN128 |
| 1521 | 24.7265 | 26 | HN073 | HN082 |  | 1521 | 25.9473 | 29 | HN073 | HN129 |
| 1522 | 27.3198 | 27 | HN073 | HN082 |  | 1522 | 27.506 | 31 | HN073 | HN129 |
| 1523 | 22.6617 | 29 | HN073 | HN083 |  | 1523 | 27.0754 | 28 | HN073 | HN129 |
| 1524 | 25.9574 | 31 | HN073 | HN083 |  | 1524 | 24.5439 | 26 | HN073 | HN130 |
| 1525 | 26.4375 | 29 | HN073 | HN085 |  | 1525 | 24.3069 | 24 | HN073 | HN132 |
| 1526 | 27.3312 | 30 | HN073 | HN085 |  | 1526 | 24.8606 | 24 | HN073 | HN133 |
| 1527 | 23.2955 | 25 | HN073 | HN085 |  | 1527 | 26.6829 | 29 | HN073 | HN134 |
| 1528 | 25.3603 | 28 | HN073 | HN086 |  | 1528 | 22.5864 | 21 | HN073 | HN135 |
| 1529 | 23.0035 | 23 | HN073 | HN092 |  | 1529 | 24.8814 | 28 | HN073 | HN135 |
| 1530 | 24.3531 | 27 | HN073 | HN093 |  | 1530 | 25.2306 | 27 | HN073 | HN138 |
| 1531 | 27.0529 | 30 | HN073 | HN094 |  | 1531 | 26.3415 | 29 | HN073 | HN141 |
| 1532 | 26.1835 | 29 | HN073 | HN094 |  | 1532 | 24.3477 | 25 | HN073 | HN144 |
| 1533 | 19.9257 | 22 | HN073 | HN095 |  | 1533 | 21.8294 | 27 | HN073 | HN147 |
| 1534 | 23.6641 | 25 | HN073 | HN095 |  | 1534 | 18.61 | 23 | HN074 | HN090 |
| 1535 | 23.0207 | 28 | HN073 | HN096 |  | 1535 | 17.2744 | 21 | HN074 | HN093 |
| 1536 | 23.559 | 25 | HN073 | HN097 |  | 1536 | 19.5778 | 25 | HN074 | HN096 |
| 1537 | 27.0403 | 26 | HN073 | HN097 |  | 1537 | 21.4678 | 23 | HN074 | HN101 |
| 1538 | 25.7229 | 24 | HN073 | HN097 |  | 1538 | 24.6751 | 26 | HN074 | HN113 |
| 1539 | 24.4191 | 27 | HN073 | HN101 |  | 1539 | 22.5359 | 26 | HN074 | HN127 |
| 1540 | 23.7351 | 24 | HN073 | HN102 |  | 1540 | 25.1438 | 28 | HN074 | HN127 |
| 1541 | 25.656 | 22 | HN073 | HN104 |  | 1541 | 23.7609 | 26 | HN075 | HN090 |
| 1542 | 27.5491 | 25 | HN073 | HN104 |  | 1542 | 22.3763 | 17 | HN075 | HN097 |
| 1543 | 24.8913 | 25 | HN073 | HN105 |  | 1543 | 21.5307 | 23 | HN075 | HN102 |
| 1544 | 27.5388 | 31 | HN073 | HN112 |  | 1544 | 24.4149 | 29 | HN075 | HN103 |
| 1545 | 26.4023 | 29 | HN073 | HN113 |  | 1545 | 22.0463 | 25 | HN075 | HN109 |
| 1546 | 27.4024 | 28 | HN073 | HN113 |  | 1546 | 24.9372 | 27 | HN075 | HN116 |
| 1547 | 22.6274 | 29 | HN073 | HN115 |  | 1547 | 23.374 | 24 | HN075 | HN123 |
| 1548 | 28.647 | 29 | HN073 | HN116 |  | 1548 | 23.1651 | 26 | HN075 | HN127 |
| 1549 | 25.7019 | 27 | HN073 | HN116 |  | 1549 | 22.9666 | 27 | HN075 | HN130 |
| 1550 | 23.9045 | 26 | HN073 | HN117 |  | 1550 | 20.955 | 26 | HN075 | HN137 |
| 1551 | 27.3675 | 28 | HN073 | HN120 |  | 1551 | 23.2437 | 24 | HN075 | HN144 |
| 1552 | 25.2456 | 26 | HN073 | HN120 |  | 1552 | 22.8193 | 28 | HN075 | HN146 |
| 1553 | 24.7246 | 26 | HN073 | HN120 |  | 1553 | 19.7465 | 24 | HN075 | HN148 |
| 1554 | 24.4075 | 25 | HN073 | HN120 |  | 1554 | 20.2262 | 20 | HN076 | HN080 |
| 1555 | 25.4091 | 27 | HN073 | HN125 |  | 1555 | 23.4982 | 27 | HN076 | HN096 |
| 1556 | 25.1124 | 28 | HN073 | HN127 |  | 1556 | 18.7271 | 22 | HN076 | HN101 |
| 1557 | 24.7924 | 28 | HN073 | HN127 |  | 1557 | 22.5437 | 22 | HN076 | HN113 |
| 1558 | 25.3812 | 28 | HN073 | HN129 |  | 1558 | 23.5955 | 27 | HN076 | HN115 |
| 1559 | 26.7972 | 32 | HN073 | HN130 |  | 1559 | 17.4391 | 21 | HN076 | HN124 |
| 1560 | 22.6334 | 26 | HN073 | HN131 |  | 1560 | 18.2257 | 19 | HN076 | HN141 |
| 1561 | 25.45 | 27 | HN073 | HN132 |  | 1561 | 21.63 | 24 | HN076 | HN143 |
| 1562 | 23.4418 | 26 | HN073 | HN136 |  | 1562 | 12.9441 | 18 | HN076 | HN146 |
| 1563 | 22.3374 | 22 | HN073 | HN138 |  | 1563 | 21.8884 | 28 | HN077 | HN083 |
| 1564 | 24.6305 | 27 | HN073 | HN138 |  | 1564 | 18.4243 | 23 | HN077 | HN086 |
| 1565 | 25.64 | 30 | HN073 | HN143 |  | 1565 | 14.812 | 19 | HN077 | HN120 |
| 1566 | 21.9491 | 23 | HN073 | HN145 |  | 1566 | 21.4659 | 25 | HN077 | HN123 |
| 1567 | 26.8167 | 27 | HN073 | HN147 |  | 1567 | 18.2742 | 19 | HN079 | HN092 |
| 1568 | 23.9183 | 24 | HN074 | HN082 |  | 1568 | 14.3433 | 20 | HN079 | HN095 |
| 1569 | 21.8393 | 25 | HN074 | HN094 |  | 1569 | 21.8261 | 28 | HN079 | HN103 |
| 1570 | 20.6183 | 24 | HN074 | HN102 |  | 1570 | 23.5414 | 29 | HN079 | HN105 |
| 1571 | 22.5736 | 24 | HN074 | HN111 |  | 1571 | 21.7416 | 24 | HN079 | HN106 |
| 1572 | 24.4781 | 25 | HN074 | HN113 |  | 1572 | 20.9664 | 24 | HN079 | HN113 |
| 1573 | 23.9273 | 25 | HN074 | HN118 |  | 1573 | 20.5427 | 23 | HN079 | HN115 |
| 1574 | 21.6738 | 23 | HN074 | HN122 |  | 1574 | 20.0003 | 22 | HN079 | HN117 |
| 1575 | 23.3841 | 28 | HN074 | HN130 |  | 1575 | 18.7085 | 23 | HN079 | HN120 |
| 1576 | 20.9087 | 25 | HN074 | HN130 |  | 1576 | 23.7902 | 27 | HN079 | HN125 |
| 1577 | 19.275 | 24 | HN074 | HN133 |  | 1577 | 21.2991 | 24 | HN079 | HN129 |
| 1578 | 17.9138 | 21 | HN074 | HN142 |  | 1578 | 20.664 | 27 | HN079 | HN132 |
| 1579 | 20.9008 | 24 | HN075 | HN080 |  | 1579 | 21.6081 | 27 | HN079 | HN137 |
| 1580 | 22.0274 | 24 | HN075 | HN080 |  | 1580 | 22.2039 | 23 | HN079 | HN148 |
| 1581 | 20.5651 | 26 | HN075 | HN083 |  | 1581 | 27.4083 | 26 | HN080 | HN082 |
| 1582 | 22.8492 | 24 | HN075 | HN090 |  | 1582 | 21.0769 | 22 | HN080 | HN090 |
| 1583 | 24.317 | 28 | HN075 | HN094 |  | 1583 | 19.9455 | 21 | HN080 | HN093 |
| 1584 | 22.3976 | 24 | HN075 | HN125 |  | 1584 | 17.9476 | 20 | HN080 | HN098 |
| 1585 | 18.2661 | 26 | HN075 | HN132 |  | 1585 | 23.4656 | 27 | HN080 | HN103 |
| 1586 | 20.5984 | 21 | HN076 | HN082 |  | 1586 | 22.4119 | 22 | HN080 | HN105 |
| 1587 | 25.3562 | 30 | HN076 | HN083 |  | 1587 | 22.3346 | 26 | HN080 | HN109 |
| 1588 | 25.882 | 29 | HN076 | HN083 |  | 1588 | 19.6468 | 21 | HN080 | HN117 |
| 1589 | 16.9046 | 16 | HN076 | HN106 |  | 1589 | 25.3194 | 26 | HN080 | HN129 |
| 1590 | 17.167 | 20 | HN076 | HN108 |  | 1590 | 23.3424 | 29 | HN080 | HN131 |
| 1591 | 20.6242 | 18 | HN076 | HN148 |  | 1591 | 23.5931 | 25 | HN080 | HN138 |
| 1592 | 18.8546 | 20 | HN076 | HN148 |  | 1592 | 24.4013 | 26 | HN080 | HN139 |
| 1593 | 18.1655 | 21 | HN076 | HN150 |  | 1593 | 20.5041 | 22 | HN080 | HN139 |
| 1594 | 22.6831 | 21 | HN077 | HN082 |  | 1594 | 21.6847 | 23 | HN080 | HN140 |
| 1595 | 24.1508 | 28 | HN077 | HN090 |  | 1595 | 24.4254 | 27 | HN081 | HN082 |
| 1596 | 25.4871 | 23 | HN077 | HN097 |  | 1596 | 22.4178 | 29 | HN081 | HN083 |
| 1597 | 20.8655 | 21 | HN077 | HN105 |  | 1597 | 23.1486 | 24 | HN081 | HN090 |
| 1598 | 21.9494 | 28 | HN077 | HN105 |  | 1598 | 25.077 | 27 | HN081 | HN090 |
| 1599 | 20.7314 | 21 | HN077 | HN113 |  | 1599 | 22.7381 | 25 | HN081 | HN094 |
| 1600 | 16.2671 | 23 | HN077 | HN114 |  | 1600 | 24.6346 | 29 | HN081 | HN103 |
| 1601 | 22.2484 | 25 | HN077 | HN138 |  | 1601 | 27.6588 | 30 | HN081 | HN116 |
| 1602 | 23.5637 | 26 | HN079 | HN080 |  | 1602 | 20.3277 | 25 | HN081 | HN130 |
| 1603 | 22.4645 | 28 | HN079 | HN083 |  | 1603 | 20.6627 | 25 | HN081 | HN136 |
| 1604 | 21.9393 | 28 | HN079 | HN087 |  | 1604 | 16.5014 | 19 | HN081 | HN141 |
| 1605 | 23.8327 | 27 | HN079 | HN087 |  | 1605 | 19.2668 | 22 | HN081 | HN148 |
| 1606 | 22.0041 | 23 | HN079 | HN087 |  | 1606 | 24.8337 | 28 | HN082 | HN083 |
| 1607 | 23.1416 | 25 | HN079 | HN093 |  | 1607 | 24.5556 | 26 | HN082 | HN092 |
| 1608 | 20.0071 | 25 | HN079 | HN109 |  | 1608 | 21.9422 | 20 | HN082 | HN092 |
| 1609 | 18.6346 | 22 | HN079 | HN111 |  | 1609 | 24.8281 | 28 | HN082 | HN096 |
| 1610 | 23.2187 | 27 | HN079 | HN111 |  | 1610 | 22.929 | 25 | HN082 | HN096 |
| 1611 | 21.5538 | 26 | HN079 | HN114 |  | 1611 | 23.4196 | 22 | HN082 | HN100 |
| 1612 | 22.3727 | 25 | HN079 | HN116 |  | 1612 | 25.3141 | 26 | HN082 | HN100 |
| 1613 | 20.9573 | 26 | HN079 | HN133 |  | 1613 | 26.8125 | 29 | HN082 | HN103 |
| 1614 | 21.0205 | 26 | HN079 | HN136 |  | 1614 | 19.9606 | 19 | HN082 | HN106 |
| 1615 | 19.9188 | 26 | HN079 | HN138 |  | 1615 | 25.7222 | 30 | HN082 | HN108 |
| 1616 | 17.6513 | 23 | HN079 | HN146 |  | 1616 | 22.471 | 25 | HN082 | HN109 |
| 1617 | 23.0163 | 25 | HN080 | HN094 |  | 1617 | 23.6858 | 26 | HN082 | HN109 |
| 1618 | 23.5546 | 26 | HN080 | HN096 |  | 1618 | 25.6702 | 27 | HN082 | HN110 |
| 1619 | 23.9599 | 26 | HN080 | HN103 |  | 1619 | 25.1746 | 27 | HN082 | HN111 |
| 1620 | 25.9158 | 28 | HN080 | HN105 |  | 1620 | 24.0675 | 26 | HN082 | HN112 |
| 1621 | 17.7315 | 17 | HN080 | HN106 |  | 1621 | 25.6724 | 30 | HN082 | HN112 |
| 1622 | 22.5519 | 24 | HN080 | HN114 |  | 1622 | 26.7008 | 27 | HN082 | HN113 |
| 1623 | 23.3919 | 26 | HN080 | HN118 |  | 1623 | 26.3122 | 27 | HN082 | HN116 |
| 1624 | 19.5479 | 22 | HN080 | HN122 |  | 1624 | 27.0351 | 26 | HN082 | HN116 |
| 1625 | 21.2461 | 24 | HN080 | HN124 |  | 1625 | 22.8229 | 26 | HN082 | HN119 |
| 1626 | 22.8053 | 26 | HN080 | HN127 |  | 1626 | 20.0051 | 23 | HN082 | HN121 |
| 1627 | 21.2975 | 26 | HN080 | HN136 |  | 1627 | 25.8483 | 28 | HN082 | HN122 |
| 1628 | 22.7491 | 25 | HN080 | HN140 |  | 1628 | 23.778 | 23 | HN082 | HN122 |
| 1629 | 21.5431 | 22 | HN080 | HN141 |  | 1629 | 27.682 | 29 | HN082 | HN125 |
| 1630 | 17.1727 | 23 | HN080 | HN147 |  | 1630 | 27.3596 | 30 | HN082 | HN125 |
| 1631 | 20.0192 | 22 | HN080 | HN149 |  | 1631 | 25.9279 | 25 | HN082 | HN128 |
| 1632 | 17.525 | 22 | HN081 | HN093 |  | 1632 | 22.2512 | 27 | HN082 | HN129 |
| 1633 | 22.8223 | 26 | HN081 | HN094 |  | 1633 | 24.9941 | 27 | HN082 | HN130 |
| 1634 | 21.9084 | 24 | HN081 | HN105 |  | 1634 | 25.6379 | 27 | HN082 | HN130 |
| 1635 | 20.4178 | 25 | HN081 | HN112 |  | 1635 | 28.0314 | 31 | HN082 | HN130 |
| 1636 | 26.9459 | 28 | HN081 | HN116 |  | 1636 | 29.6063 | 30 | HN082 | HN140 |
| 1637 | 23.2077 | 28 | HN081 | HN125 |  | 1637 | 22.0099 | 25 | HN082 | HN144 |
| 1638 | 18.5659 | 25 | HN081 | HN127 |  | 1638 | 24.8715 | 28 | HN082 | HN146 |
| 1639 | 23.0243 | 29 | HN081 | HN130 |  | 1639 | 26.3818 | 29 | HN082 | HN147 |
| 1640 | 20.3631 | 25 | HN081 | HN138 |  | 1640 | 24.0146 | 28 | HN083 | HN084 |
| 1641 | 24.5311 | 24 | HN082 | HN085 |  | 1641 | 24.7972 | 29 | HN083 | HN087 |
| 1642 | 22.3807 | 25 | HN082 | HN090 |  | 1642 | 24.1458 | 30 | HN083 | HN091 |
| 1643 | 22.8104 | 25 | HN082 | HN090 |  | 1643 | 24.4614 | 29 | HN083 | HN091 |
| 1644 | 26.0543 | 28 | HN082 | HN091 |  | 1644 | 24.5898 | 28 | HN083 | HN093 |
| 1645 | 27.0055 | 25 | HN082 | HN093 |  | 1645 | 23.0291 | 31 | HN083 | HN094 |
| 1646 | 23.5185 | 24 | HN082 | HN094 |  | 1646 | 21.0697 | 25 | HN083 | HN098 |
| 1647 | 25.6795 | 27 | HN082 | HN095 |  | 1647 | 23.7984 | 21 | HN083 | HN106 |
| 1648 | 25.2745 | 27 | HN082 | HN096 |  | 1648 | 23.5509 | 29 | HN083 | HN107 |
| 1649 | 23.3541 | 21 | HN082 | HN097 |  | 1649 | 27.747 | 30 | HN083 | HN111 |
| 1650 | 22.3252 | 23 | HN082 | HN105 |  | 1650 | 26.6832 | 28 | HN083 | HN113 |
| 1651 | 23.8497 | 26 | HN082 | HN110 |  | 1651 | 25.8415 | 27 | HN083 | HN113 |
| 1652 | 26.6357 | 26 | HN082 | HN111 |  | 1652 | 28.4552 | 32 | HN083 | HN113 |
| 1653 | 25.0905 | 27 | HN082 | HN111 |  | 1653 | 26.5329 | 31 | HN083 | HN114 |
| 1654 | 27.7752 | 29 | HN082 | HN112 |  | 1654 | 26.7424 | 32 | HN083 | HN114 |
| 1655 | 25.8526 | 26 | HN082 | HN112 |  | 1655 | 23.279 | 26 | HN083 | HN116 |
| 1656 | 21.1676 | 25 | HN082 | HN114 |  | 1656 | 29.2467 | 32 | HN083 | HN116 |
| 1657 | 26.6028 | 25 | HN082 | HN114 |  | 1657 | 25.4457 | 28 | HN083 | HN122 |
| 1658 | 23.1155 | 24 | HN082 | HN116 |  | 1658 | 23.0402 | 27 | HN083 | HN123 |
| 1659 | 26.2933 | 29 | HN082 | HN118 |  | 1659 | 23.8767 | 28 | HN083 | HN125 |
| 1660 | 26.6093 | 25 | HN082 | HN120 |  | 1660 | 27.1001 | 31 | HN083 | HN125 |
| 1661 | 22.8345 | 25 | HN082 | HN123 |  | 1661 | 23.547 | 33 | HN083 | HN127 |
| 1662 | 23.3995 | 27 | HN082 | HN126 |  | 1662 | 23.6041 | 28 | HN083 | HN128 |
| 1663 | 23.5046 | 25 | HN082 | HN126 |  | 1663 | 25.2884 | 31 | HN083 | HN129 |
| 1664 | 27.1225 | 28 | HN082 | HN129 |  | 1664 | 22.0234 | 26 | HN083 | HN132 |
| 1665 | 23.5049 | 26 | HN082 | HN129 |  | 1665 | 22.7703 | 28 | HN083 | HN133 |
| 1666 | 25.3812 | 28 | HN082 | HN129 |  | 1666 | 23.7109 | 29 | HN083 | HN134 |
| 1667 | 21.49 | 25 | HN082 | HN130 |  | 1667 | 24.3757 | 32 | HN083 | HN136 |
| 1668 | 26.5665 | 29 | HN082 | HN131 |  | 1668 | 25.4656 | 30 | HN083 | HN137 |
| 1669 | 22.4779 | 26 | HN082 | HN132 |  | 1669 | 24.2968 | 28 | HN083 | HN139 |
| 1670 | 24.0002 | 24 | HN082 | HN139 |  | 1670 | 27.5098 | 29 | HN083 | HN140 |
| 1671 | 20.4812 | 19 | HN082 | HN142 |  | 1671 | 26.1322 | 26 | HN083 | HN140 |
| 1672 | 22.7334 | 21 | HN082 | HN142 |  | 1672 | 22.7651 | 30 | HN083 | HN146 |
| 1673 | 22.8615 | 27 | HN082 | HN143 |  | 1673 | 20.4951 | 25 | HN083 | HN150 |
| 1674 | 23.6892 | 24 | HN082 | HN143 |  | 1674 | 18.4002 | 19 | HN084 | HN091 |
| 1675 | 25.0213 | 31 | HN082 | HN144 |  | 1675 | 23.7905 | 26 | HN084 | HN096 |
| 1676 | 23.017 | 20 | HN082 | HN144 |  | 1676 | 23.381 | 29 | HN084 | HN096 |
| 1677 | 21.9686 | 22 | HN082 | HN146 |  | 1677 | 24.1917 | 25 | HN084 | HN125 |
| 1678 | 24.2677 | 24 | HN082 | HN148 |  | 1678 | 19.7802 | 21 | HN084 | HN133 |
| 1679 | 25.7061 | 23 | HN082 | HN148 |  | 1679 | 20.489 | 23 | HN084 | HN137 |
| 1680 | 23.6877 | 28 | HN083 | HN091 |  | 1680 | 20.1332 | 23 | HN084 | HN143 |
| 1681 | 23.2842 | 29 | HN083 | HN094 |  | 1681 | 22.9967 | 22 | HN085 | HN113 |
| 1682 | 24.422 | 29 | HN083 | HN100 |  | 1682 | 23.8651 | 25 | HN085 | HN116 |
| 1683 | 24.4705 | 31 | HN083 | HN101 |  | 1683 | 24.1544 | 26 | HN085 | HN116 |
| 1684 | 23.0945 | 26 | HN083 | HN101 |  | 1684 | 20.7723 | 24 | HN085 | HN120 |
| 1685 | 25.4861 | 31 | HN083 | HN106 |  | 1685 | 17.4717 | 22 | HN085 | HN128 |
| 1686 | 22.243 | 27 | HN083 | HN109 |  | 1686 | 20.4128 | 23 | HN086 | HN107 |
| 1687 | 27.2022 | 29 | HN083 | HN111 |  | 1687 | 19.3616 | 20 | HN086 | HN114 |
| 1688 | 26.7866 | 28 | HN083 | HN111 |  | 1688 | 15.8901 | 19 | HN086 | HN117 |
| 1689 | 25.0463 | 30 | HN083 | HN116 |  | 1689 | 21.7203 | 25 | HN086 | HN118 |
| 1690 | 22.2688 | 26 | HN083 | HN123 |  | 1690 | 20.7306 | 21 | HN086 | HN125 |
| 1691 | 25.7472 | 31 | HN083 | HN130 |  | 1691 | 16.5961 | 22 | HN086 | HN139 |
| 1692 | 25.0395 | 31 | HN083 | HN130 |  | 1692 | 25.6652 | 27 | HN087 | HN090 |
| 1693 | 21.5117 | 28 | HN083 | HN131 |  | 1693 | 21.4643 | 24 | HN087 | HN093 |
| 1694 | 18.4522 | 23 | HN083 | HN135 |  | 1694 | 26.3546 | 29 | HN087 | HN094 |
| 1695 | 25.3923 | 30 | HN083 | HN137 |  | 1695 | 21.7706 | 20 | HN087 | HN097 |
| 1696 | 21.7642 | 24 | HN083 | HN145 |  | 1696 | 22.6345 | 21 | HN087 | HN106 |
| 1697 | 21.5883 | 28 | HN083 | HN146 |  | 1697 | 24.1597 | 27 | HN087 | HN107 |
| 1698 | 22.4383 | 30 | HN083 | HN146 |  | 1698 | 18.2199 | 20 | HN087 | HN120 |
| 1699 | 20.5109 | 27 | HN083 | HN147 |  | 1699 | 18.2519 | 22 | HN087 | HN124 |
| 1700 | 20.886 | 24 | HN083 | HN149 |  | 1700 | 21.4841 | 25 | HN087 | HN126 |
| 1701 | 21.0447 | 24 | HN083 | HN150 |  | 1701 | 19.2454 | 23 | HN087 | HN128 |
| 1702 | 22.9556 | 27 | HN084 | HN094 |  | 1702 | 27.0488 | 28 | HN087 | HN129 |
| 1703 | 17.5862 | 21 | HN084 | HN094 |  | 1703 | 21.3779 | 17 | HN088 | HN097 |
| 1704 | 23.3334 | 22 | HN084 | HN118 |  | 1704 | 15.9351 | 17 | HN088 | HN105 |
| 1705 | 22.6534 | 26 | HN084 | HN129 |  | 1705 | 17.6575 | 21 | HN090 | HN092 |
| 1706 | 23.1922 | 26 | HN084 | HN130 |  | 1706 | 24.9535 | 28 | HN090 | HN094 |
| 1707 | 21.6373 | 21 | HN084 | HN143 |  | 1707 | 25.3652 | 30 | HN090 | HN094 |
| 1708 | 22.0853 | 24 | HN085 | HN094 |  | 1708 | 20.476 | 21 | HN090 | HN095 |
| 1709 | 21.2605 | 24 | HN085 | HN096 |  | 1709 | 24.3644 | 28 | HN090 | HN096 |
| 1710 | 22.3248 | 21 | HN085 | HN097 |  | 1710 | 23.887 | 31 | HN090 | HN103 |
| 1711 | 21.0206 | 22 | HN085 | HN106 |  | 1711 | 24.1816 | 28 | HN090 | HN108 |
| 1712 | 22.7002 | 23 | HN085 | HN116 |  | 1712 | 22.3388 | 26 | HN090 | HN109 |
| 1713 | 20.717 | 20 | HN085 | HN117 |  | 1713 | 24.5113 | 29 | HN090 | HN113 |
| 1714 | 23.0905 | 26 | HN085 | HN131 |  | 1714 | 25.8129 | 27 | HN090 | HN113 |
| 1715 | 21.3257 | 21 | HN085 | HN135 |  | 1715 | 21.5041 | 27 | HN090 | HN114 |
| 1716 | 21.1088 | 24 | HN085 | HN140 |  | 1716 | 24.5671 | 25 | HN090 | HN117 |
| 1717 | 20.1228 | 23 | HN085 | HN143 |  | 1717 | 23.4411 | 28 | HN090 | HN128 |
| 1718 | 21.7664 | 24 | HN085 | HN149 |  | 1718 | 23.2936 | 27 | HN090 | HN129 |
| 1719 | 17.487 | 21 | HN086 | HN087 |  | 1719 | 20.8594 | 28 | HN090 | HN138 |
| 1720 | 14.4689 | 20 | HN086 | HN096 |  | 1720 | 18.7112 | 22 | HN090 | HN149 |
| 1721 | 20.2319 | 26 | HN086 | HN096 |  | 1721 | 24.077 | 27 | HN091 | HN094 |
| 1722 | 19.5187 | 30 | HN086 | HN105 |  | 1722 | 21.2188 | 24 | HN091 | HN103 |
| 1723 | 22.0575 | 21 | HN086 | HN113 |  | 1723 | 22.573 | 28 | HN091 | HN105 |
| 1724 | 21.137 | 25 | HN086 | HN115 |  | 1724 | 20.9263 | 18 | HN091 | HN106 |
| 1725 | 23.0612 | 27 | HN086 | HN129 |  | 1725 | 21.6088 | 20 | HN091 | HN111 |
| 1726 | 21.5099 | 26 | HN087 | HN090 |  | 1726 | 16.5887 | 22 | HN091 | HN114 |
| 1727 | 20.9673 | 22 | HN087 | HN093 |  | 1727 | 20.8622 | 24 | HN091 | HN124 |
| 1728 | 24.3127 | 28 | HN087 | HN096 |  | 1728 | 20.8622 | 24 | HN091 | HN124 |
| 1729 | 21.2763 | 21 | HN087 | HN111 |  | 1729 | 17.2267 | 18 | HN091 | HN135 |
| 1730 | 24.7741 | 26 | HN087 | HN113 |  | 1730 | 18.5236 | 23 | HN091 | HN143 |
| 1731 | 26.8304 | 30 | HN087 | HN129 |  | 1731 | 17.4568 | 23 | HN092 | HN125 |
| 1732 | 19.9163 | 24 | HN087 | HN131 |  | 1732 | 20.9855 | 26 | HN092 | HN130 |
| 1733 | 19.5715 | 24 | HN087 | HN140 |  | 1733 | 22.7132 | 28 | HN092 | HN131 |
| 1734 | 23.3538 | 24 | HN087 | HN143 |  | 1734 | 19.6235 | 19 | HN092 | HN140 |
| 1735 | 20.1895 | 24 | HN088 | HN094 |  | 1735 | 24.3361 | 27 | HN093 | HN098 |
| 1736 | 19.0015 | 16 | HN088 | HN097 |  | 1736 | 20.563 | 25 | HN093 | HN098 |
| 1737 | 14.4661 | 13 | HN088 | HN108 |  | 1737 | 26.1534 | 30 | HN093 | HN105 |
| 1738 | 18.4928 | 21 | HN088 | HN125 |  | 1738 | 22.2696 | 24 | HN093 | HN108 |
| 1739 | 17.7446 | 20 | HN088 | HN144 |  | 1739 | 21.8958 | 23 | HN093 | HN108 |
| 1740 | 24.1129 | 26 | HN090 | HN091 |  | 1740 | 25.7261 | 26 | HN093 | HN113 |
| 1741 | 17.5089 | 22 | HN090 | HN093 |  | 1741 | 25.7261 | 26 | HN093 | HN113 |
| 1742 | 22.3371 | 24 | HN090 | HN102 |  | 1742 | 23.4862 | 27 | HN093 | HN125 |
| 1743 | 21.3766 | 25 | HN090 | HN109 |  | 1743 | 23.5893 | 29 | HN093 | HN129 |
| 1744 | 21.9023 | 28 | HN090 | HN112 |  | 1744 | 19.978 | 24 | HN093 | HN134 |
| 1745 | 27.6607 | 31 | HN090 | HN116 |  | 1745 | 23.0555 | 23 | HN093 | HN141 |
| 1746 | 23.1894 | 25 | HN090 | HN117 |  | 1746 | 21.1357 | 23 | HN093 | HN149 |
| 1747 | 26.7345 | 31 | HN090 | HN118 |  | 1747 | 23.8796 | 25 | HN094 | HN097 |
| 1748 | 24.2443 | 27 | HN090 | HN118 |  | 1748 | 23.8692 | 27 | HN094 | HN098 |
| 1749 | 25.3304 | 29 | HN090 | HN122 |  | 1749 | 22.7188 | 28 | HN094 | HN107 |
| 1750 | 24.9566 | 28 | HN090 | HN122 |  | 1750 | 24.8697 | 29 | HN094 | HN112 |
| 1751 | 21.6482 | 26 | HN090 | HN122 |  | 1751 | 26.4686 | 26 | HN094 | HN116 |
| 1752 | 21.6868 | 28 | HN090 | HN125 |  | 1752 | 24.5793 | 25 | HN094 | HN116 |
| 1753 | 22.1336 | 26 | HN090 | HN126 |  | 1753 | 22.4559 | 29 | HN094 | HN119 |
| 1754 | 19.4527 | 25 | HN090 | HN127 |  | 1754 | 25.2435 | 29 | HN094 | HN122 |
| 1755 | 18.2549 | 25 | HN090 | HN128 |  | 1755 | 22.7668 | 28 | HN094 | HN124 |
| 1756 | 22.9294 | 27 | HN090 | HN129 |  | 1756 | 26.5067 | 31 | HN094 | HN125 |
| 1757 | 21.8577 | 24 | HN090 | HN129 |  | 1757 | 24.2035 | 31 | HN094 | HN129 |
| 1758 | 22.5349 | 27 | HN090 | HN130 |  | 1758 | 21.6231 | 27 | HN094 | HN134 |
| 1759 | 21.7521 | 28 | HN090 | HN131 |  | 1759 | 23.6755 | 25 | HN094 | HN137 |
| 1760 | 19.7578 | 25 | HN090 | HN131 |  | 1760 | 26.6128 | 30 | HN094 | HN137 |
| 1761 | 24.2943 | 27 | HN090 | HN134 |  | 1761 | 19.792 | 23 | HN094 | HN142 |
| 1762 | 19.4068 | 24 | HN090 | HN137 |  | 1762 | 24.1446 | 24 | HN095 | HN113 |
| 1763 | 23.2995 | 27 | HN090 | HN146 |  | 1763 | 16.3015 | 19 | HN095 | HN128 |
| 1764 | 22.2647 | 25 | HN090 | HN150 |  | 1764 | 28.9173 | 30 | HN096 | HN097 |
| 1765 | 19.4464 | 20 | HN091 | HN111 |  | 1765 | 21.2174 | 26 | HN096 | HN100 |
| 1766 | 17.5968 | 19 | HN091 | HN117 |  | 1766 | 22.6836 | 25 | HN096 | HN101 |
| 1767 | 21.2626 | 24 | HN091 | HN118 |  | 1767 | 25.6257 | 31 | HN096 | HN102 |
| 1768 | 22.4016 | 26 | HN091 | HN131 |  | 1768 | 24.4328 | 31 | HN096 | HN103 |
| 1769 | 16.606 | 21 | HN091 | HN133 |  | 1769 | 23.9839 | 28 | HN096 | HN105 |
| 1770 | 18.567 | 21 | HN091 | HN141 |  | 1770 | 23.9839 | 27 | HN096 | HN105 |
| 1771 | 19.1622 | 22 | HN091 | HN143 |  | 1771 | 25.3645 | 24 | HN096 | HN106 |
| 1772 | 21.0557 | 25 | HN092 | HN126 |  | 1772 | 24.9485 | 30 | HN096 | HN109 |
| 1773 | 20.9037 | 23 | HN092 | HN127 |  | 1773 | 24.8028 | 27 | HN096 | HN111 |
| 1774 | 18.8008 | 21 | HN092 | HN138 |  | 1774 | 25.5707 | 29 | HN096 | HN111 |
| 1775 | 24.066 | 28 | HN093 | HN094 |  | 1775 | 25.4361 | 28 | HN096 | HN114 |
| 1776 | 18.497 | 23 | HN093 | HN095 |  | 1776 | 26.057 | 26 | HN096 | HN116 |
| 1777 | 27.0351 | 29 | HN093 | HN096 |  | 1777 | 26.5721 | 30 | HN096 | HN118 |
| 1778 | 20.2616 | 22 | HN093 | HN105 |  | 1778 | 23.716 | 29 | HN096 | HN120 |
| 1779 | 21.5225 | 24 | HN093 | HN107 |  | 1779 | 22.2797 | 26 | HN096 | HN122 |
| 1780 | 26.9077 | 26 | HN093 | HN116 |  | 1780 | 26.7481 | 30 | HN096 | HN125 |
| 1781 | 21.4687 | 21 | HN093 | HN117 |  | 1781 | 24.3784 | 29 | HN096 | HN125 |
| 1782 | 23.0495 | 22 | HN093 | HN123 |  | 1782 | 23.4042 | 31 | HN096 | HN129 |
| 1783 | 25.3627 | 28 | HN093 | HN129 |  | 1783 | 22.9347 | 29 | HN096 | HN130 |
| 1784 | 23.0271 | 25 | HN093 | HN129 |  | 1784 | 21.4469 | 28 | HN096 | HN131 |
| 1785 | 18.8419 | 21 | HN093 | HN139 |  | 1785 | 24.888 | 29 | HN096 | HN132 |
| 1786 | 21.2231 | 24 | HN094 | HN100 |  | 1786 | 22.6942 | 30 | HN096 | HN133 |
| 1787 | 18.9726 | 26 | HN094 | HN108 |  | 1787 | 28.3768 | 32 | HN096 | HN140 |
| 1788 | 22.045 | 27 | HN094 | HN109 |  | 1788 | 28.3768 | 32 | HN096 | HN140 |
| 1789 | 23.0032 | 28 | HN094 | HN110 |  | 1789 | 25.3778 | 30 | HN096 | HN144 |
| 1790 | 22.8689 | 27 | HN094 | HN110 |  | 1790 | 23.0846 | 28 | HN096 | HN146 |
| 1791 | 23.7523 | 24 | HN094 | HN111 |  | 1791 | 20.6018 | 22 | HN097 | HN102 |
| 1792 | 28.9767 | 29 | HN094 | HN111 |  | 1792 | 22.1353 | 23 | HN097 | HN110 |
| 1793 | 24.428 | 28 | HN094 | HN114 |  | 1793 | 22.4195 | 26 | HN097 | HN112 |
| 1794 | 25.0778 | 29 | HN094 | HN114 |  | 1794 | 24.3567 | 22 | HN097 | HN116 |
| 1795 | 28.8871 | 30 | HN094 | HN116 |  | 1795 | 22.073 | 23 | HN097 | HN116 |
| 1796 | 28.8871 | 30 | HN094 | HN116 |  | 1796 | 23.2156 | 22 | HN097 | HN128 |
| 1797 | 26.1037 | 29 | HN094 | HN118 |  | 1797 | 21.5265 | 23 | HN097 | HN128 |
| 1798 | 22.0417 | 27 | HN094 | HN120 |  | 1798 | 24.2019 | 25 | HN097 | HN130 |
| 1799 | 23.7025 | 29 | HN094 | HN123 |  | 1799 | 25.2191 | 22 | HN097 | HN131 |
| 1800 | 20.239 | 26 | HN094 | HN126 |  | 1800 | 24.5054 | 20 | HN097 | HN136 |
| 1801 | 24.4001 | 28 | HN094 | HN128 |  | 1801 | 25.436 | 21 | HN097 | HN138 |
| 1802 | 23.7705 | 31 | HN094 | HN129 |  | 1802 | 21.6187 | 19 | HN097 | HN146 |
| 1803 | 25.4617 | 30 | HN094 | HN130 |  | 1803 | 20.8312 | 18 | HN097 | HN147 |
| 1804 | 24.3492 | 27 | HN094 | HN130 |  | 1804 | 19.0809 | 16 | HN098 | HN106 |
| 1805 | 25.3375 | 29 | HN094 | HN131 |  | 1805 | 21.296 | 26 | HN098 | HN109 |
| 1806 | 24.5852 | 27 | HN094 | HN131 |  | 1806 | 25.1972 | 24 | HN098 | HN116 |
| 1807 | 24.9086 | 31 | HN094 | HN131 |  | 1807 | 19.4913 | 24 | HN098 | HN117 |
| 1808 | 21.6025 | 25 | HN094 | HN132 |  | 1808 | 21.3953 | 26 | HN098 | HN126 |
| 1809 | 22.6899 | 27 | HN094 | HN133 |  | 1809 | 21.6195 | 27 | HN098 | HN128 |
| 1810 | 26.583 | 30 | HN094 | HN137 |  | 1810 | 23.5022 | 29 | HN098 | HN128 |
| 1811 | 22.7454 | 29 | HN094 | HN139 |  | 1811 | 19.2436 | 29 | HN098 | HN129 |
| 1812 | 20.9166 | 26 | HN094 | HN143 |  | 1812 | 18.4062 | 23 | HN098 | HN135 |
| 1813 | 18.6478 | 24 | HN094 | HN144 |  | 1813 | 14.4973 | 21 | HN098 | HN139 |
| 1814 | 22.6054 | 25 | HN094 | HN145 |  | 1814 | 19.8317 | 24 | HN098 | HN143 |
| 1815 | 24.5403 | 29 | HN094 | HN147 |  | 1815 | 20.5001 | 23 | HN100 | HN107 |
| 1816 | 21.426 | 24 | HN094 | HN148 |  | 1816 | 20.5768 | 24 | HN100 | HN116 |
| 1817 | 25.9197 | 31 | HN094 | HN149 |  | 1817 | 22.0598 | 24 | HN100 | HN130 |
| 1818 | 21.844 | 19 | HN095 | HN106 |  | 1818 | 22.1702 | 26 | HN100 | HN130 |
| 1819 | 24.8252 | 23 | HN096 | HN097 |  | 1819 | 20.0183 | 23 | HN100 | HN131 |
| 1820 | 21.4469 | 27 | HN096 | HN098 |  | 1820 | 21.7638 | 26 | HN101 | HN103 |
| 1821 | 24.3398 | 29 | HN096 | HN101 |  | 1821 | 25.1234 | 28 | HN101 | HN105 |
| 1822 | 23.705 | 28 | HN096 | HN103 |  | 1822 | 25.1234 | 28 | HN101 | HN105 |
| 1823 | 27.0374 | 31 | HN096 | HN103 |  | 1823 | 23.2102 | 23 | HN101 | HN113 |
| 1824 | 20.7521 | 28 | HN096 | HN103 |  | 1824 | 19.4683 | 22 | HN101 | HN132 |
| 1825 | 23.9832 | 29 | HN096 | HN107 |  | 1825 | 20.13 | 23 | HN101 | HN132 |
| 1826 | 24.1389 | 26 | HN096 | HN110 |  | 1826 | 19.239 | 19 | HN102 | HN104 |
| 1827 | 26.9425 | 31 | HN096 | HN116 |  | 1827 | 19.7187 | 24 | HN102 | HN108 |
| 1828 | 29.0617 | 30 | HN096 | HN116 |  | 1828 | 25.0158 | 29 | HN102 | HN118 |
| 1829 | 23.2436 | 27 | HN096 | HN117 |  | 1829 | 19.902 | 24 | HN102 | HN122 |
| 1830 | 19.432 | 25 | HN096 | HN120 |  | 1830 | 17.9277 | 21 | HN102 | HN128 |
| 1831 | 25.8538 | 29 | HN096 | HN123 |  | 1831 | 18.9877 | 24 | HN102 | HN140 |
| 1832 | 24.6651 | 28 | HN096 | HN124 |  | 1832 | 19.332 | 22 | HN102 | HN145 |
| 1833 | 23.8166 | 29 | HN096 | HN125 |  | 1833 | 18.233 | 24 | HN102 | HN147 |
| 1834 | 22.739 | 28 | HN096 | HN129 |  | 1834 | 21.175 | 26 | HN103 | HN104 |
| 1835 | 22.625 | 29 | HN096 | HN130 |  | 1835 | 25.6282 | 28 | HN103 | HN105 |
| 1836 | 23.1051 | 27 | HN096 | HN135 |  | 1836 | 28.8802 | 34 | HN103 | HN105 |
| 1837 | 25.6252 | 29 | HN096 | HN137 |  | 1837 | 26.4025 | 32 | HN103 | HN105 |
| 1838 | 26.4921 | 29 | HN096 | HN140 |  | 1838 | 20.6222 | 26 | HN103 | HN107 |
| 1839 | 22.5336 | 26 | HN096 | HN143 |  | 1839 | 24.3607 | 28 | HN103 | HN116 |
| 1840 | 21.5186 | 27 | HN096 | HN149 |  | 1840 | 23.1073 | 27 | HN103 | HN123 |
| 1841 | 24.6007 | 24 | HN097 | HN108 |  | 1841 | 25.7477 | 26 | HN103 | HN123 |
| 1842 | 22.2872 | 22 | HN097 | HN111 |  | 1842 | 28.2652 | 33 | HN103 | HN129 |
| 1843 | 25.3998 | 23 | HN097 | HN116 |  | 1843 | 26.6696 | 32 | HN103 | HN131 |
| 1844 | 21.5431 | 19 | HN097 | HN117 |  | 1844 | 21.1417 | 28 | HN103 | HN141 |
| 1845 | 20.3775 | 17 | HN097 | HN122 |  | 1845 | 17.1435 | 22 | HN103 | HN142 |
| 1846 | 21.6312 | 19 | HN097 | HN124 |  | 1846 | 22.2976 | 26 | HN103 | HN150 |
| 1847 | 23.1798 | 25 | HN097 | HN125 |  | 1847 | 24.2412 | 27 | HN104 | HN107 |
| 1848 | 25.0826 | 21 | HN097 | HN129 |  | 1848 | 24.6004 | 28 | HN104 | HN113 |
| 1849 | 22.8417 | 22 | HN097 | HN132 |  | 1849 | 25.2882 | 24 | HN104 | HN116 |
| 1850 | 25.5675 | 24 | HN097 | HN143 |  | 1850 | 18.906 | 22 | HN104 | HN128 |
| 1851 | 23.024 | 20 | HN097 | HN145 |  | 1851 | 21.6544 | 27 | HN104 | HN130 |
| 1852 | 21.5988 | 20 | HN097 | HN145 |  | 1852 | 19.669 | 19 | HN104 | HN134 |
| 1853 | 21.6807 | 27 | HN098 | HN110 |  | 1853 | 18.6633 | 19 | HN104 | HN147 |
| 1854 | 19.3232 | 22 | HN098 | HN122 |  | 1854 | 23.187 | 28 | HN105 | HN110 |
| 1855 | 22.7818 | 25 | HN098 | HN132 |  | 1855 | 26.4969 | 26 | HN105 | HN111 |
| 1856 | 21.7537 | 24 | HN101 | HN105 |  | 1856 | 22.8896 | 28 | HN105 | HN112 |
| 1857 | 22.1894 | 22 | HN101 | HN106 |  | 1857 | 25.9861 | 27 | HN105 | HN113 |
| 1858 | 22.7609 | 26 | HN101 | HN107 |  | 1858 | 26.1993 | 30 | HN105 | HN125 |
| 1859 | 21.3019 | 23 | HN101 | HN107 |  | 1859 | 24.0924 | 30 | HN105 | HN128 |
| 1860 | 26.2166 | 31 | HN101 | HN129 |  | 1860 | 21.4733 | 25 | HN105 | HN137 |
| 1861 | 20.6818 | 25 | HN101 | HN130 |  | 1861 | 23.3382 | 26 | HN105 | HN137 |
| 1862 | 21.7362 | 29 | HN101 | HN131 |  | 1862 | 23.3517 | 30 | HN105 | HN138 |
| 1863 | 17.4445 | 21 | HN101 | HN150 |  | 1863 | 20.0434 | 25 | HN105 | HN143 |
| 1864 | 20.8577 | 23 | HN102 | HN104 |  | 1864 | 21.0448 | 18 | HN106 | HN116 |
| 1865 | 27.6989 | 32 | HN102 | HN129 |  | 1865 | 23.2837 | 22 | HN106 | HN122 |
| 1866 | 22.4912 | 28 | HN102 | HN131 |  | 1866 | 23.3064 | 22 | HN106 | HN130 |
| 1867 | 21.5637 | 26 | HN102 | HN131 |  | 1867 | 23.9152 | 21 | HN106 | HN130 |
| 1868 | 27.9935 | 32 | HN103 | HN105 |  | 1868 | 21.5509 | 19 | HN106 | HN142 |
| 1869 | 26.964 | 31 | HN103 | HN105 |  | 1869 | 24.4057 | 23 | HN107 | HN111 |
| 1870 | 21.4581 | 30 | HN103 | HN112 |  | 1870 | 25.9111 | 27 | HN107 | HN113 |
| 1871 | 22.0535 | 27 | HN103 | HN115 |  | 1871 | 19.1673 | 21 | HN107 | HN117 |
| 1872 | 27.3982 | 30 | HN103 | HN116 |  | 1872 | 22.2982 | 25 | HN107 | HN124 |
| 1873 | 21.3395 | 25 | HN103 | HN121 |  | 1873 | 23.3563 | 29 | HN107 | HN128 |
| 1874 | 24.2674 | 26 | HN103 | HN123 |  | 1874 | 23.5193 | 27 | HN107 | HN130 |
| 1875 | 26.2391 | 31 | HN103 | HN123 |  | 1875 | 20.5303 | 24 | HN107 | HN133 |
| 1876 | 23.0074 | 29 | HN103 | HN124 |  | 1876 | 20.3413 | 22 | HN107 | HN135 |
| 1877 | 18.5777 | 28 | HN103 | HN127 |  | 1877 | 23.6152 | 28 | HN107 | HN147 |
| 1878 | 24.9972 | 28 | HN103 | HN129 |  | 1878 | 21.5336 | 25 | HN108 | HN110 |
| 1879 | 22.345 | 28 | HN103 | HN137 |  | 1879 | 22.7563 | 23 | HN108 | HN114 |
| 1880 | 20.8524 | 27 | HN103 | HN141 |  | 1880 | 18.4584 | 20 | HN108 | HN117 |
| 1881 | 24.9966 | 27 | HN103 | HN144 |  | 1881 | 21.3928 | 24 | HN108 | HN117 |
| 1882 | 20.3092 | 28 | HN103 | HN149 |  | 1882 | 22.2442 | 24 | HN108 | HN118 |
| 1883 | 20.2256 | 26 | HN103 | HN150 |  | 1883 | 22.3801 | 25 | HN108 | HN127 |
| 1884 | 21.9979 | 22 | HN104 | HN108 |  | 1884 | 18.6142 | 22 | HN108 | HN138 |
| 1885 | 20.7636 | 21 | HN104 | HN113 |  | 1885 | 24.5813 | 26 | HN109 | HN111 |
| 1886 | 23.6107 | 24 | HN104 | HN116 |  | 1886 | 24.3092 | 28 | HN109 | HN116 |
| 1887 | 23.639 | 22 | HN104 | HN116 |  | 1887 | 18.1232 | 21 | HN109 | HN141 |
| 1888 | 23.1723 | 23 | HN104 | HN138 |  | 1888 | 21.7406 | 28 | HN110 | HN111 |
| 1889 | 21.2307 | 23 | HN104 | HN138 |  | 1889 | 25.2608 | 27 | HN110 | HN116 |
| 1890 | 25.0529 | 25 | HN104 | HN140 |  | 1890 | 23.7949 | 23 | HN110 | HN116 |
| 1891 | 21.3866 | 19 | HN104 | HN143 |  | 1891 | 24.8056 | 28 | HN110 | HN127 |
| 1892 | 27.7569 | 27 | HN105 | HN116 |  | 1892 | 20.976 | 28 | HN110 | HN129 |
| 1893 | 17.5495 | 21 | HN105 | HN122 |  | 1893 | 19.1664 | 22 | HN110 | HN140 |
| 1894 | 23.2145 | 27 | HN105 | HN124 |  | 1894 | 25.4915 | 30 | HN111 | HN118 |
| 1895 | 23.333 | 26 | HN105 | HN125 |  | 1895 | 18.6818 | 20 | HN111 | HN120 |
| 1896 | 15.0696 | 23 | HN105 | HN126 |  | 1896 | 24.1631 | 25 | HN111 | HN124 |
| 1897 | 23.6186 | 30 | HN105 | HN126 |  | 1897 | 25.1167 | 26 | HN111 | HN125 |
| 1898 | 22.5419 | 28 | HN105 | HN130 |  | 1898 | 25.1922 | 26 | HN111 | HN131 |
| 1899 | 21.5898 | 28 | HN105 | HN136 |  | 1899 | 21.8789 | 24 | HN111 | HN136 |
| 1900 | 22.8859 | 27 | HN105 | HN138 |  | 1900 | 19.0592 | 25 | HN111 | HN147 |
| 1901 | 22.6292 | 19 | HN106 | HN118 |  | 1901 | 23.7631 | 25 | HN112 | HN117 |
| 1902 | 19.5987 | 21 | HN106 | HN128 |  | 1902 | 22.019 | 27 | HN112 | HN118 |
| 1903 | 16.6152 | 19 | HN106 | HN128 |  | 1903 | 18.2006 | 21 | HN112 | HN122 |
| 1904 | 19.2623 | 22 | HN106 | HN131 |  | 1904 | 22.2464 | 27 | HN112 | HN125 |
| 1905 | 20.0822 | 20 | HN106 | HN149 |  | 1905 | 20.8775 | 27 | HN112 | HN128 |
| 1906 | 23.6371 | 24 | HN107 | HN111 |  | 1906 | 20.3378 | 26 | HN112 | HN144 |
| 1907 | 24.0833 | 25 | HN107 | HN113 |  | 1907 | 22.231 | 22 | HN112 | HN148 |
| 1908 | 19.6432 | 22 | HN107 | HN113 |  | 1908 | 19.9634 | 22 | HN112 | HN149 |
| 1909 | 20.2841 | 23 | HN107 | HN115 |  | 1909 | 21.5214 | 23 | HN113 | HN119 |
| 1910 | 23.0362 | 25 | HN107 | HN118 |  | 1910 | 21.0056 | 22 | HN113 | HN122 |
| 1911 | 21.8431 | 26 | HN107 | HN118 |  | 1911 | 21.5977 | 26 | HN113 | HN125 |
| 1912 | 26.7694 | 32 | HN107 | HN129 |  | 1912 | 24.9911 | 27 | HN113 | HN126 |
| 1913 | 18.8662 | 22 | HN107 | HN139 |  | 1913 | 24.7826 | 26 | HN113 | HN127 |
| 1914 | 21.3324 | 25 | HN107 | HN144 |  | 1914 | 26.7064 | 30 | HN113 | HN127 |
| 1915 | 18.5506 | 24 | HN108 | HN109 |  | 1915 | 27.5305 | 28 | HN113 | HN129 |
| 1916 | 21.2126 | 24 | HN108 | HN111 |  | 1916 | 27.1914 | 30 | HN113 | HN129 |
| 1917 | 24.0421 | 24 | HN108 | HN116 |  | 1917 | 22.113 | 22 | HN113 | HN132 |
| 1918 | 18.4554 | 22 | HN108 | HN124 |  | 1918 | 22.163 | 26 | HN113 | HN133 |
| 1919 | 23.7669 | 27 | HN108 | HN131 |  | 1919 | 22.0546 | 23 | HN113 | HN136 |
| 1920 | 19.059 | 21 | HN108 | HN132 |  | 1920 | 24.8805 | 24 | HN113 | HN137 |
| 1921 | 18.117 | 22 | HN108 | HN132 |  | 1921 | 27.267 | 28 | HN113 | HN138 |
| 1922 | 22.2948 | 23 | HN108 | HN144 |  | 1922 | 21.8593 | 21 | HN113 | HN140 |
| 1923 | 19.9186 | 21 | HN108 | HN144 |  | 1923 | 22.1325 | 22 | HN113 | HN144 |
| 1924 | 22.7087 | 26 | HN108 | HN149 |  | 1924 | 21.477 | 23 | HN113 | HN146 |
| 1925 | 20.3478 | 24 | HN109 | HN110 |  | 1925 | 21.6722 | 23 | HN113 | HN146 |
| 1926 | 22.1484 | 26 | HN109 | HN127 |  | 1926 | 19.1625 | 18 | HN113 | HN150 |
| 1927 | 18.5856 | 20 | HN109 | HN137 |  | 1927 | 25.1084 | 31 | HN114 | HN129 |
| 1928 | 21.8778 | 26 | HN109 | HN143 |  | 1928 | 19.1477 | 23 | HN114 | HN141 |
| 1929 | 25.2837 | 26 | HN110 | HN113 |  | 1929 | 26.8087 | 28 | HN115 | HN116 |
| 1930 | 23.5291 | 26 | HN110 | HN116 |  | 1930 | 21.0492 | 24 | HN115 | HN122 |
| 1931 | 18.5255 | 18 | HN110 | HN148 |  | 1931 | 21.1351 | 24 | HN115 | HN124 |
| 1932 | 25.7335 | 28 | HN111 | HN116 |  | 1932 | 22.0978 | 25 | HN115 | HN129 |
| 1933 | 23.791 | 23 | HN111 | HN116 |  | 1933 | 24.101 | 26 | HN115 | HN129 |
| 1934 | 22.1057 | 23 | HN111 | HN122 |  | 1934 | 23.6435 | 28 | HN115 | HN131 |
| 1935 | 23.5695 | 25 | HN111 | HN126 |  | 1935 | 22.0399 | 25 | HN115 | HN134 |
| 1936 | 22.7469 | 28 | HN111 | HN128 |  | 1936 | 22.4494 | 23 | HN116 | HN117 |
| 1937 | 26.414 | 29 | HN111 | HN129 |  | 1937 | 24.4595 | 24 | HN116 | HN118 |
| 1938 | 20.224 | 23 | HN111 | HN133 |  | 1938 | 25.8684 | 25 | HN116 | HN118 |
| 1939 | 22.0373 | 25 | HN111 | HN137 |  | 1939 | 24.5927 | 26 | HN116 | HN120 |
| 1940 | 26.3144 | 25 | HN111 | HN144 |  | 1940 | 27.1633 | 26 | HN116 | HN123 |
| 1941 | 25.9512 | 28 | HN112 | HN116 |  | 1941 | 24.7515 | 29 | HN116 | HN124 |
| 1942 | 18.6964 | 27 | HN112 | HN129 |  | 1942 | 27.1331 | 28 | HN116 | HN125 |
| 1943 | 22.3108 | 29 | HN112 | HN131 |  | 1943 | 21.6209 | 25 | HN116 | HN126 |
| 1944 | 16.2979 | 20 | HN112 | HN142 |  | 1944 | 24.9182 | 26 | HN116 | HN128 |
| 1945 | 19.0839 | 24 | HN112 | HN143 |  | 1945 | 26.9631 | 26 | HN116 | HN129 |
| 1946 | 19.2791 | 25 | HN112 | HN143 |  | 1946 | 24.6035 | 28 | HN116 | HN130 |
| 1947 | 22.4521 | 23 | HN113 | HN117 |  | 1947 | 27.2716 | 29 | HN116 | HN130 |
| 1948 | 21.7913 | 23 | HN113 | HN117 |  | 1948 | 28.5685 | 29 | HN116 | HN131 |
| 1949 | 22.0781 | 24 | HN113 | HN118 |  | 1949 | 26.7332 | 27 | HN116 | HN132 |
| 1950 | 25.9602 | 27 | HN113 | HN118 |  | 1950 | 25.5247 | 29 | HN116 | HN137 |
| 1951 | 23.103 | 23 | HN113 | HN118 |  | 1951 | 25.1163 | 27 | HN116 | HN140 |
| 1952 | 27.8065 | 27 | HN113 | HN123 |  | 1952 | 23.3085 | 25 | HN116 | HN141 |
| 1953 | 24.8125 | 28 | HN113 | HN125 |  | 1953 | 17.4256 | 21 | HN116 | HN146 |
| 1954 | 20.5001 | 25 | HN113 | HN127 |  | 1954 | 23.7731 | 25 | HN116 | HN146 |
| 1955 | 27.3796 | 28 | HN113 | HN131 |  | 1955 | 24.6105 | 27 | HN116 | HN147 |
| 1956 | 23.2704 | 26 | HN113 | HN133 |  | 1956 | 23.1222 | 21 | HN116 | HN148 |
| 1957 | 25.1702 | 27 | HN113 | HN133 |  | 1957 | 23.2736 | 22 | HN116 | HN149 |
| 1958 | 23.9019 | 27 | HN113 | HN138 |  | 1958 | 21.1594 | 24 | HN117 | HN121 |
| 1959 | 26.814 | 29 | HN113 | HN138 |  | 1959 | 21.8029 | 19 | HN117 | HN123 |
| 1960 | 23.3848 | 24 | HN113 | HN148 |  | 1960 | 21.558 | 21 | HN117 | HN124 |
| 1961 | 23.1028 | 23 | HN113 | HN150 |  | 1961 | 26.7273 | 26 | HN117 | HN129 |
| 1962 | 23.3319 | 26 | HN114 | HN116 |  | 1962 | 24.5688 | 28 | HN117 | HN130 |
| 1963 | 22.6268 | 26 | HN114 | HN125 |  | 1963 | 24.1651 | 25 | HN117 | HN131 |
| 1964 | 22.2933 | 28 | HN114 | HN130 |  | 1964 | 27.0031 | 27 | HN117 | HN131 |
| 1965 | 25.1892 | 30 | HN114 | HN131 |  | 1965 | 26.5374 | 26 | HN117 | HN131 |
| 1966 | 20.6511 | 25 | HN114 | HN137 |  | 1966 | 22.7263 | 23 | HN117 | HN140 |
| 1967 | 22.4245 | 26 | HN114 | HN137 |  | 1967 | 21.7001 | 22 | HN117 | HN143 |
| 1968 | 19.2708 | 21 | HN114 | HN148 |  | 1968 | 24.245 | 26 | HN117 | HN144 |
| 1969 | 22.7834 | 28 | HN115 | HN125 |  | 1969 | 19.978 | 25 | HN117 | HN146 |
| 1970 | 22.8252 | 27 | HN115 | HN129 |  | 1970 | 21.1336 | 22 | HN117 | HN147 |
| 1971 | 20.3156 | 22 | HN115 | HN148 |  | 1971 | 20.7151 | 22 | HN118 | HN128 |
| 1972 | 19.8825 | 21 | HN115 | HN148 |  | 1972 | 22.2018 | 22 | HN118 | HN129 |
| 1973 | 21.413 | 24 | HN116 | HN118 |  | 1973 | 25.4328 | 27 | HN118 | HN131 |
| 1974 | 22.8665 | 23 | HN116 | HN120 |  | 1974 | 22.477 | 25 | HN118 | HN134 |
| 1975 | 21.8535 | 20 | HN116 | HN121 |  | 1975 | 24.2575 | 25 | HN118 | HN140 |
| 1976 | 23.9347 | 25 | HN116 | HN122 |  | 1976 | 24.6755 | 24 | HN118 | HN141 |
| 1977 | 23.9059 | 27 | HN116 | HN125 |  | 1977 | 19.2561 | 27 | HN119 | HN131 |
| 1978 | 23.1816 | 26 | HN116 | HN125 |  | 1978 | 19.0791 | 22 | HN119 | HN143 |
| 1979 | 26.8474 | 30 | HN116 | HN129 |  | 1979 | 20.1045 | 26 | HN120 | HN128 |
| 1980 | 28.808 | 27 | HN116 | HN129 |  | 1980 | 23.5024 | 27 | HN120 | HN131 |
| 1981 | 24.6575 | 26 | HN116 | HN129 |  | 1981 | 21.5692 | 25 | HN120 | HN131 |
| 1982 | 24.7684 | 28 | HN116 | HN129 |  | 1982 | 17.6385 | 22 | HN120 | HN141 |
| 1983 | 25.978 | 31 | HN116 | HN130 |  | 1983 | 20.0599 | 24 | HN120 | HN145 |
| 1984 | 20.9301 | 26 | HN116 | HN130 |  | 1984 | 20.4996 | 24 | HN121 | HN125 |
| 1985 | 24.9716 | 27 | HN116 | HN131 |  | 1985 | 17.6494 | 24 | HN121 | HN147 |
| 1986 | 23.5849 | 25 | HN116 | HN133 |  | 1986 | 22.193 | 26 | HN122 | HN125 |
| 1987 | 23.1295 | 24 | HN116 | HN136 |  | 1987 | 26.0614 | 30 | HN122 | HN129 |
| 1988 | 21.7013 | 26 | HN116 | HN136 |  | 1988 | 19.2889 | 25 | HN122 | HN137 |
| 1989 | 24.6739 | 26 | HN116 | HN137 |  | 1989 | 20.4347 | 18 | HN122 | HN148 |
| 1990 | 24.4211 | 26 | HN116 | HN137 |  | 1990 | 22.9061 | 30 | HN123 | HN129 |
| 1991 | 26.9636 | 28 | HN116 | HN137 |  | 1991 | 22.6942 | 29 | HN123 | HN131 |
| 1992 | 22.883 | 23 | HN116 | HN139 |  | 1992 | 18.8953 | 25 | HN123 | HN131 |
| 1993 | 22.5112 | 24 | HN116 | HN140 |  | 1993 | 21.836 | 26 | HN123 | HN137 |
| 1994 | 25.7978 | 27 | HN116 | HN140 |  | 1994 | 17.8904 | 19 | HN123 | HN142 |
| 1995 | 19.7224 | 21 | HN117 | HN123 |  | 1995 | 17.386 | 23 | HN123 | HN146 |
| 1996 | 27.6117 | 30 | HN117 | HN129 |  | 1996 | 21.6564 | 27 | HN124 | HN127 |
| 1997 | 22.0435 | 23 | HN117 | HN130 |  | 1997 | 22.6242 | 26 | HN124 | HN132 |
| 1998 | 20.0507 | 21 | HN117 | HN145 |  | 1998 | 21.7265 | 25 | HN124 | HN141 |
| 1999 | 24.6845 | 27 | HN118 | HN127 |  | 1999 | 19.3417 | 21 | HN124 | HN148 |
| 2000 | 26.6489 | 31 | HN118 | HN129 |  | 2000 | 19.7919 | 22 | HN124 | HN149 |
| 2001 | 23.6295 | 28 | HN118 | HN130 |  | 2001 | 22.8258 | 26 | HN125 | HN132 |
| 2002 | 23.7236 | 25 | HN118 | HN132 |  | 2002 | 22.1773 | 26 | HN125 | HN133 |
| 2003 | 21.0474 | 24 | HN118 | HN133 |  | 2003 | 22.7863 | 27 | HN125 | HN144 |
| 2004 | 22.3291 | 23 | HN118 | HN137 |  | 2004 | 18.9671 | 22 | HN125 | HN145 |
| 2005 | 18.9566 | 24 | HN120 | HN132 |  | 2005 | 22.0393 | 25 | HN125 | HN147 |
| 2006 | 20.7288 | 23 | HN120 | HN132 |  | 2006 | 21.5853 | 25 | HN125 | HN150 |
| 2007 | 18.7398 | 22 | HN120 | HN149 |  | 2007 | 24.6181 | 24 | HN126 | HN129 |
| 2008 | 17.9661 | 22 | HN121 | HN131 |  | 2008 | 23.2163 | 26 | HN126 | HN131 |
| 2009 | 20.2182 | 23 | HN122 | HN123 |  | 2009 | 18.8981 | 20 | HN126 | HN135 |
| 2010 | 22.4344 | 26 | HN122 | HN129 |  | 2010 | 21.3087 | 25 | HN126 | HN137 |
| 2011 | 25.865 | 29 | HN122 | HN131 |  | 2011 | 16.4144 | 23 | HN126 | HN138 |
| 2012 | 20.3142 | 24 | HN122 | HN137 |  | 2012 | 22.4148 | 25 | HN126 | HN140 |
| 2013 | 21.3962 | 19 | HN122 | HN144 |  | 2013 | 16.6316 | 21 | HN126 | HN143 |
| 2014 | 22.4527 | 27 | HN123 | HN126 |  | 2014 | 20.6349 | 23 | HN126 | HN144 |
| 2015 | 21.5388 | 25 | HN123 | HN126 |  | 2015 | 25.8833 | 30 | HN127 | HN129 |
| 2016 | 21.1789 | 23 | HN123 | HN129 |  | 2016 | 25.0366 | 31 | HN127 | HN129 |
| 2017 | 21.1739 | 23 | HN123 | HN132 |  | 2017 | 22.7174 | 28 | HN127 | HN130 |
| 2018 | 23.1205 | 28 | HN124 | HN127 |  | 2018 | 24.5253 | 29 | HN127 | HN131 |
| 2019 | 24.3415 | 27 | HN125 | HN129 |  | 2019 | 23.7345 | 25 | HN127 | HN142 |
| 2020 | 25.9674 | 29 | HN125 | HN138 |  | 2020 | 18.0859 | 24 | HN127 | HN144 |
| 2021 | 19.0431 | 26 | HN126 | HN137 |  | 2021 | 24.083 | 24 | HN127 | HN148 |
| 2022 | 24.1877 | 31 | HN127 | HN131 |  | 2022 | 24.5045 | 26 | HN128 | HN129 |
| 2023 | 26.546 | 31 | HN127 | HN131 |  | 2023 | 26.2431 | 31 | HN129 | HN130 |
| 2024 | 20.2141 | 23 | HN127 | HN137 |  | 2024 | 26.7694 | 32 | HN129 | HN132 |
| 2025 | 24.9449 | 28 | HN127 | HN141 |  | 2025 | 23.261 | 27 | HN129 | HN136 |
| 2026 | 22.5764 | 24 | HN127 | HN143 |  | 2026 | 23.1872 | 31 | HN129 | HN147 |
| 2027 | 20.7565 | 26 | HN127 | HN146 |  | 2027 | 25.4317 | 27 | HN129 | HN148 |
| 2028 | 21.5938 | 29 | HN128 | HN131 |  | 2028 | 24.7782 | 29 | HN130 | HN131 |
| 2029 | 20.4434 | 24 | HN128 | HN136 |  | 2029 | 23.7791 | 27 | HN130 | HN132 |
| 2030 | 22.4021 | 28 | HN129 | HN131 |  | 2030 | 23.6246 | 31 | HN130 | HN134 |
| 2031 | 21.1546 | 28 | HN129 | HN138 |  | 2031 | 20.9858 | 23 | HN130 | HN144 |
| 2032 | 24.771 | 28 | HN129 | HN140 |  | 2032 | 22.4687 | 26 | HN131 | HN135 |
| 2033 | 21.2613 | 21 | HN130 | HN132 |  | 2033 | 26.588 | 29 | HN131 | HN140 |
| 2034 | 19.5365 | 21 | HN130 | HN137 |  | 2034 | 24.9763 | 29 | HN131 | HN141 |
| 2035 | 23.0193 | 29 | HN130 | HN137 |  | 2035 | 21.6721 | 28 | HN131 | HN144 |
| 2036 | 25.1932 | 28 | HN130 | HN138 |  | 2036 | 23.6452 | 28 | HN131 | HN146 |
| 2037 | 25.5889 | 30 | HN130 | HN138 |  | 2037 | 24.9324 | 26 | HN131 | HN148 |
| 2038 | 20.2642 | 23 | HN131 | HN132 |  | 2038 | 21.3996 | 24 | HN132 | HN142 |
| 2039 | 24.0368 | 28 | HN131 | HN132 |  | 2039 | 19.9655 | 22 | HN132 | HN145 |
| 2040 | 25.017 | 28 | HN131 | HN133 |  | 2040 | 22.4084 | 23 | HN132 | HN148 |
| 2041 | 21.9478 | 25 | HN131 | HN134 |  | 2041 | 21.1839 | 23 | HN132 | HN149 |
| 2042 | 22.8908 | 27 | HN131 | HN137 |  | 2042 | 20.3025 | 24 | HN133 | HN137 |
| 2043 | 22.0805 | 27 | HN131 | HN141 |  | 2043 | 18.6408 | 22 | HN133 | HN140 |
| 2044 | 22.9883 | 29 | HN131 | HN146 |  | 2044 | 17.7311 | 20 | HN133 | HN149 |
| 2045 | 22.1339 | 27 | HN131 | HN146 |  | 2045 | 17.1144 | 21 | HN134 | HN146 |
| 2046 | 18.9008 | 24 | HN131 | HN147 |  | 2046 | 22.6766 | 24 | HN135 | HN143 |
| 2047 | 24.0756 | 29 | HN131 | HN149 |  | 2047 | 17.4117 | 19 | HN135 | HN147 |
| 2048 | 21.1202 | 23 | HN132 | HN134 |  | 2048 | 19.4377 | 23 | HN135 | HN148 |
| 2049 | 21.4498 | 23 | HN132 | HN144 |  | 2049 | 19.9519 | 23 | HN136 | HN144 |
| 2050 | 19.6782 | 21 | HN132 | HN144 |  | 2050 | 16.6053 | 21 | HN136 | HN150 |
| 2051 | 19.2921 | 25 | HN134 | HN136 |  | 2051 | 19.7615 | 22 | HN138 | HN149 |
| 2052 | 16.914 | 20 | HN135 | HN144 |  | 2052 | 20.11 | 23 | HN138 | HN150 |
| 2053 | 21.949 | 24 | HN136 | HN138 |  | 2053 | 22.5153 | 21 | HN139 | HN148 |
| 2054 | 23.2519 | 26 | HN137 | HN144 |  | 2054 | 19.9672 | 19 | HN142 | HN148 |
| 2055 | 17.4976 | 22 | HN138 | HN143 |  | 2055 | 17.5668 | 22 | HN143 | HN144 |
| 2056 | 19.3778 | 21 | HN140 | HN144 |  | 2056 | 18.4595 | 23 | HN144 | HN146 |
| 2057 | 16.9243 | 22 | HN141 | HN146 |  | 2057 | 22.547 | 24 | HN144 | HN149 |
| 2058 | 22.3946 | 27 | HN143 | HN147 |  | 2058 | 19.5449 | 22 | HN147 | HN150 |
| 2059 | 19.0629 | 19 | HN148 | HN149 |  | 2059 | 19.7207 | 19 | HN148 | HN149 |

**Supplementary Table 10.** Simulated breeding results for an F_2_ population size of 800.

| Method-Ped | | | | |  | Method-Bulk | | | | |
| --- | --- | --- | --- | --- | --- | --- | --- | --- | --- | --- |
| Individual | GenoValue | NSA | SCP1 | SCP2 |  | Individual | GenoValue | NSA | SCP1 | SCP2 |
| 1 | 15.335 | 21 | HN002 | HN021 |  | 1 | 24.0821 | 22 | HN002 | HN004 |
| 2 | 23.7823 | 28 | HN002 | HN034 |  | 2 | 22.428 | 24 | HN002 | HN006 |
| 3 | 20.2052 | 18 | HN002 | HN040 |  | 3 | 19.6147 | 23 | HN002 | HN017 |
| 4 | 21.5649 | 21 | HN002 | HN040 |  | 4 | 19.3247 | 20 | HN002 | HN022 |
| 5 | 23.3916 | 26 | HN002 | HN041 |  | 5 | 20.5294 | 20 | HN002 | HN030 |
| 6 | 23.8405 | 21 | HN002 | HN043 |  | 6 | 21.4286 | 24 | HN002 | HN050 |
| 7 | 18.5132 | 20 | HN002 | HN045 |  | 7 | 22.7018 | 23 | HN002 | HN066 |
| 8 | 18.975 | 23 | HN002 | HN046 |  | 8 | 21.9512 | 21 | HN002 | HN072 |
| 9 | 22.0209 | 26 | HN002 | HN060 |  | 9 | 22.0199 | 21 | HN002 | HN082 |
| 10 | 23.4949 | 25 | HN002 | HN065 |  | 10 | 21.2816 | 28 | HN002 | HN083 |
| 11 | 23.8291 | 26 | HN002 | HN065 |  | 11 | 20.5705 | 27 | HN002 | HN096 |
| 12 | 21.0697 | 22 | HN002 | HN066 |  | 12 | 22.3419 | 26 | HN002 | HN108 |
| 13 | 21.7004 | 24 | HN002 | HN083 |  | 13 | 19.0363 | 21 | HN002 | HN108 |
| 14 | 21.5424 | 20 | HN002 | HN093 |  | 14 | 22.7386 | 23 | HN002 | HN113 |
| 15 | 15.8 | 20 | HN002 | HN104 |  | 15 | 20.9867 | 22 | HN002 | HN113 |
| 16 | 17.3285 | 16 | HN002 | HN106 |  | 16 | 22.8697 | 28 | HN002 | HN115 |
| 17 | 23.2183 | 27 | HN002 | HN109 |  | 17 | 22.072 | 23 | HN002 | HN126 |
| 18 | 16.3532 | 24 | HN002 | HN109 |  | 18 | 21.2126 | 27 | HN002 | HN129 |
| 19 | 25.823 | 23 | HN002 | HN113 |  | 19 | 23.9528 | 26 | HN002 | HN130 |
| 20 | 22.4288 | 24 | HN002 | HN113 |  | 20 | 19.1688 | 22 | HN002 | HN145 |
| 21 | 22.6101 | 22 | HN002 | HN116 |  | 21 | 21.2552 | 17 | HN003 | HN004 |
| 22 | 21.2533 | 22 | HN002 | HN117 |  | 22 | 22.4117 | 22 | HN003 | HN016 |
| 23 | 25.9137 | 28 | HN002 | HN125 |  | 23 | 21.4606 | 24 | HN003 | HN024 |
| 24 | 17.8921 | 21 | HN002 | HN128 |  | 24 | 21.2141 | 20 | HN003 | HN026 |
| 25 | 20.2614 | 18 | HN003 | HN004 |  | 25 | 22.7975 | 19 | HN003 | HN032 |
| 26 | 19.4307 | 18 | HN003 | HN007 |  | 26 | 20.1465 | 20 | HN003 | HN033 |
| 27 | 21.3134 | 24 | HN003 | HN009 |  | 27 | 19.8412 | 25 | HN003 | HN042 |
| 28 | 20.9919 | 23 | HN003 | HN009 |  | 28 | 20.87 | 19 | HN003 | HN048 |
| 29 | 21.6187 | 21 | HN003 | HN012 |  | 29 | 24.0223 | 27 | HN003 | HN050 |
| 30 | 22.9426 | 23 | HN003 | HN016 |  | 30 | 19.1608 | 21 | HN003 | HN053 |
| 31 | 21.2844 | 19 | HN003 | HN020 |  | 31 | 20.6393 | 24 | HN003 | HN056 |
| 32 | 21.0241 | 20 | HN003 | HN021 |  | 32 | 22.2344 | 23 | HN003 | HN061 |
| 33 | 23.7926 | 21 | HN003 | HN022 |  | 33 | 25.4023 | 27 | HN003 | HN065 |
| 34 | 22.3815 | 20 | HN003 | HN022 |  | 34 | 21.1378 | 24 | HN003 | HN065 |
| 35 | 21.855 | 26 | HN003 | HN024 |  | 35 | 23.3915 | 22 | HN003 | HN069 |
| 36 | 21.0633 | 22 | HN003 | HN033 |  | 36 | 18.1834 | 18 | HN003 | HN076 |
| 37 | 21.6166 | 21 | HN003 | HN058 |  | 37 | 22.2619 | 23 | HN003 | HN085 |
| 38 | 22.3484 | 24 | HN003 | HN060 |  | 38 | 24.5904 | 20 | HN003 | HN097 |
| 39 | 21.3409 | 27 | HN003 | HN065 |  | 39 | 21.0987 | 18 | HN003 | HN097 |
| 40 | 26.6776 | 29 | HN003 | HN065 |  | 40 | 21.4056 | 21 | HN003 | HN101 |
| 41 | 21.155 | 22 | HN003 | HN068 |  | 41 | 22.2399 | 27 | HN003 | HN105 |
| 42 | 20.9313 | 22 | HN003 | HN069 |  | 42 | 22.534 | 23 | HN003 | HN110 |
| 43 | 25.1337 | 25 | HN003 | HN073 |  | 43 | 22.2177 | 23 | HN003 | HN110 |
| 44 | 24.6701 | 26 | HN003 | HN073 |  | 44 | 22.0114 | 24 | HN003 | HN115 |
| 45 | 18.6291 | 23 | HN003 | HN075 |  | 45 | 23.0564 | 24 | HN003 | HN118 |
| 46 | 21.4382 | 20 | HN003 | HN093 |  | 46 | 21.3988 | 23 | HN003 | HN118 |
| 47 | 21.5373 | 22 | HN003 | HN093 |  | 47 | 21.401 | 22 | HN003 | HN123 |
| 48 | 23.7141 | 25 | HN003 | HN094 |  | 48 | 20.9709 | 22 | HN003 | HN127 |
| 49 | 20.6986 | 26 | HN003 | HN094 |  | 49 | 23.2518 | 24 | HN003 | HN130 |
| 50 | 21.7114 | 20 | HN003 | HN097 |  | 50 | 24.5275 | 22 | HN004 | HN007 |
| 51 | 20.305 | 23 | HN003 | HN102 |  | 51 | 20.8525 | 21 | HN004 | HN009 |
| 52 | 20.9 | 19 | HN003 | HN110 |  | 52 | 22.8179 | 22 | HN004 | HN018 |
| 53 | 26.3036 | 25 | HN003 | HN113 |  | 53 | 23.3054 | 23 | HN004 | HN019 |
| 54 | 25.1928 | 26 | HN003 | HN113 |  | 54 | 25.5492 | 20 | HN004 | HN022 |
| 55 | 22.6312 | 22 | HN003 | HN115 |  | 55 | 23.9982 | 23 | HN004 | HN029 |
| 56 | 19.0121 | 22 | HN003 | HN124 |  | 56 | 23.0404 | 20 | HN004 | HN030 |
| 57 | 20.7677 | 24 | HN003 | HN124 |  | 57 | 23.8013 | 25 | HN004 | HN034 |
| 58 | 22.288 | 28 | HN003 | HN129 |  | 58 | 23.1778 | 21 | HN004 | HN035 |
| 59 | 22.0688 | 28 | HN003 | HN131 |  | 59 | 22.1103 | 21 | HN004 | HN044 |
| 60 | 21.3018 | 23 | HN003 | HN131 |  | 60 | 22.3363 | 25 | HN004 | HN046 |
| 61 | 18.1286 | 17 | HN003 | HN134 |  | 61 | 23.7259 | 22 | HN004 | HN053 |
| 62 | 19.6033 | 20 | HN003 | HN134 |  | 62 | 21.191 | 20 | HN004 | HN055 |
| 63 | 21.6379 | 23 | HN003 | HN136 |  | 63 | 21.6284 | 27 | HN004 | HN057 |
| 64 | 23.7479 | 20 | HN004 | HN007 |  | 64 | 25.4275 | 24 | HN004 | HN064 |
| 65 | 23.8693 | 24 | HN004 | HN007 |  | 65 | 25.2059 | 25 | HN004 | HN064 |
| 66 | 24.2325 | 25 | HN004 | HN017 |  | 66 | 21.0277 | 23 | HN004 | HN066 |
| 67 | 22.2147 | 21 | HN004 | HN017 |  | 67 | 23.6525 | 23 | HN004 | HN068 |
| 68 | 21.5296 | 22 | HN004 | HN019 |  | 68 | 24.2022 | 24 | HN004 | HN069 |
| 69 | 24.207 | 22 | HN004 | HN020 |  | 69 | 22.5602 | 23 | HN004 | HN069 |
| 70 | 21.4841 | 22 | HN004 | HN020 |  | 70 | 21.5889 | 21 | HN004 | HN073 |
| 71 | 26.1435 | 26 | HN004 | HN020 |  | 71 | 23.8202 | 22 | HN004 | HN082 |
| 72 | 25.2428 | 22 | HN004 | HN022 |  | 72 | 24.8918 | 28 | HN004 | HN083 |
| 73 | 27.7509 | 24 | HN004 | HN022 |  | 73 | 23.0514 | 25 | HN004 | HN090 |
| 74 | 19.5728 | 23 | HN004 | HN024 |  | 74 | 21.3007 | 24 | HN004 | HN090 |
| 75 | 19.8048 | 19 | HN004 | HN025 |  | 75 | 25.1788 | 30 | HN004 | HN096 |
| 76 | 26.6901 | 24 | HN004 | HN026 |  | 76 | 20.8459 | 19 | HN004 | HN098 |
| 77 | 26.4159 | 23 | HN004 | HN026 |  | 77 | 23.4021 | 24 | HN004 | HN101 |
| 78 | 26.9212 | 25 | HN004 | HN026 |  | 78 | 22.573 | 22 | HN004 | HN104 |
| 79 | 21.2396 | 23 | HN004 | HN032 |  | 79 | 22.8385 | 25 | HN004 | HN105 |
| 80 | 21.015 | 21 | HN004 | HN033 |  | 80 | 22.9837 | 21 | HN004 | HN111 |
| 81 | 22.5464 | 25 | HN004 | HN039 |  | 81 | 23.3829 | 23 | HN004 | HN119 |
| 82 | 23.0128 | 22 | HN004 | HN039 |  | 82 | 21.3045 | 21 | HN004 | HN121 |
| 83 | 18.5161 | 20 | HN004 | HN042 |  | 83 | 27.8675 | 28 | HN004 | HN123 |
| 84 | 17.8224 | 21 | HN004 | HN042 |  | 84 | 21.3149 | 22 | HN004 | HN125 |
| 85 | 23.8168 | 24 | HN004 | HN046 |  | 85 | 23.0201 | 23 | HN004 | HN126 |
| 86 | 19.9892 | 20 | HN004 | HN050 |  | 86 | 25.5069 | 23 | HN004 | HN129 |
| 87 | 24.4089 | 28 | HN004 | HN050 |  | 87 | 22.5251 | 20 | HN004 | HN133 |
| 88 | 25.224 | 25 | HN004 | HN050 |  | 88 | 19.8219 | 23 | HN004 | HN135 |
| 89 | 24.926 | 28 | HN004 | HN051 |  | 89 | 21.7313 | 21 | HN004 | HN148 |
| 90 | 20.1995 | 22 | HN004 | HN057 |  | 90 | 20.9302 | 24 | HN004 | HN150 |
| 91 | 18.7152 | 18 | HN004 | HN058 |  | 91 | 21.042 | 22 | HN005 | HN007 |
| 92 | 23.6557 | 25 | HN004 | HN058 |  | 92 | 20.3603 | 24 | HN005 | HN012 |
| 93 | 25.4331 | 29 | HN004 | HN060 |  | 93 | 21.8586 | 27 | HN005 | HN024 |
| 94 | 26.4352 | 26 | HN004 | HN060 |  | 94 | 15.0522 | 18 | HN005 | HN038 |
| 95 | 23.4033 | 25 | HN004 | HN060 |  | 95 | 21.868 | 26 | HN005 | HN060 |
| 96 | 25.5476 | 24 | HN004 | HN061 |  | 96 | 17.7798 | 24 | HN005 | HN079 |
| 97 | 22.9675 | 22 | HN004 | HN061 |  | 97 | 22.5361 | 27 | HN005 | HN082 |
| 98 | 21.5751 | 24 | HN004 | HN062 |  | 98 | 13.4601 | 18 | HN005 | HN091 |
| 99 | 23.6549 | 26 | HN004 | HN062 |  | 99 | 17.8057 | 19 | HN005 | HN097 |
| 100 | 25.5693 | 25 | HN004 | HN064 |  | 100 | 20.7763 | 25 | HN005 | HN111 |
| 101 | 24.728 | 23 | HN004 | HN066 |  | 101 | 21.8537 | 24 | HN005 | HN113 |
| 102 | 25.0223 | 27 | HN004 | HN067 |  | 102 | 21.6906 | 29 | HN005 | HN129 |
| 103 | 22.7501 | 21 | HN004 | HN067 |  | 103 | 12.7499 | 16 | HN005 | HN142 |
| 104 | 18.5654 | 19 | HN004 | HN072 |  | 104 | 21.4899 | 24 | HN006 | HN027 |
| 105 | 23.1721 | 24 | HN004 | HN072 |  | 105 | 19.5701 | 28 | HN006 | HN037 |
| 106 | 27.7581 | 26 | HN004 | HN073 |  | 106 | 18.3389 | 24 | HN006 | HN047 |
| 107 | 28.2131 | 28 | HN004 | HN073 |  | 107 | 23.2732 | 28 | HN006 | HN060 |
| 108 | 22.9727 | 23 | HN004 | HN075 |  | 108 | 18.6009 | 22 | HN006 | HN061 |
| 109 | 23.5603 | 24 | HN004 | HN075 |  | 109 | 20.6113 | 23 | HN006 | HN068 |
| 110 | 20.847 | 18 | HN004 | HN080 |  | 110 | 25.9548 | 29 | HN006 | HN073 |
| 111 | 24.4793 | 25 | HN004 | HN080 |  | 111 | 22.5728 | 23 | HN006 | HN082 |
| 112 | 23.6395 | 25 | HN004 | HN085 |  | 112 | 23.7835 | 25 | HN006 | HN082 |
| 113 | 25.0532 | 26 | HN004 | HN090 |  | 113 | 13.9044 | 17 | HN006 | HN086 |
| 114 | 23.4268 | 24 | HN004 | HN090 |  | 114 | 16.6902 | 20 | HN006 | HN088 |
| 115 | 22.4451 | 23 | HN004 | HN090 |  | 115 | 20.3857 | 25 | HN006 | HN090 |
| 116 | 22.4803 | 23 | HN004 | HN090 |  | 116 | 17.337 | 21 | HN006 | HN124 |
| 117 | 19.9574 | 21 | HN004 | HN092 |  | 117 | 20.9058 | 25 | HN006 | HN125 |
| 118 | 23.2646 | 23 | HN004 | HN093 |  | 118 | 20.0582 | 25 | HN006 | HN130 |
| 119 | 21.2479 | 21 | HN004 | HN093 |  | 119 | 18.379 | 24 | HN006 | HN137 |
| 120 | 26.0328 | 25 | HN004 | HN111 |  | 120 | 25.9146 | 27 | HN007 | HN011 |
| 121 | 20.9693 | 25 | HN004 | HN112 |  | 121 | 25.294 | 27 | HN007 | HN012 |
| 122 | 23.856 | 25 | HN004 | HN114 |  | 122 | 24.6745 | 24 | HN007 | HN021 |
| 123 | 24.091 | 26 | HN004 | HN115 |  | 123 | 22.9337 | 25 | HN007 | HN023 |
| 124 | 22.2254 | 22 | HN004 | HN116 |  | 124 | 25.0804 | 25 | HN007 | HN034 |
| 125 | 20.0278 | 21 | HN004 | HN117 |  | 125 | 27.1468 | 29 | HN007 | HN051 |
| 126 | 18.5506 | 17 | HN004 | HN121 |  | 126 | 19.9633 | 21 | HN007 | HN067 |
| 127 | 20.6184 | 22 | HN004 | HN136 |  | 127 | 25.6041 | 24 | HN007 | HN070 |
| 128 | 23.6264 | 25 | HN004 | HN138 |  | 128 | 22.01 | 23 | HN007 | HN073 |
| 129 | 21.4355 | 25 | HN004 | HN138 |  | 129 | 25.0864 | 24 | HN007 | HN085 |
| 130 | 21.2763 | 22 | HN004 | HN139 |  | 130 | 23.3525 | 26 | HN007 | HN094 |
| 131 | 21.5375 | 22 | HN004 | HN141 |  | 131 | 24.2185 | 25 | HN007 | HN096 |
| 132 | 21.5431 | 21 | HN004 | HN144 |  | 132 | 24.5959 | 21 | HN007 | HN097 |
| 133 | 18.9448 | 17 | HN004 | HN144 |  | 133 | 21.9426 | 22 | HN007 | HN105 |
| 134 | 19.4283 | 20 | HN004 | HN149 |  | 134 | 18.1898 | 18 | HN007 | HN106 |
| 135 | 23.1107 | 25 | HN005 | HN026 |  | 135 | 23.7905 | 22 | HN007 | HN106 |
| 136 | 15.1412 | 20 | HN005 | HN033 |  | 136 | 20.4861 | 24 | HN007 | HN112 |
| 137 | 18.3089 | 23 | HN005 | HN052 |  | 137 | 26.2348 | 26 | HN007 | HN114 |
| 138 | 22.3752 | 24 | HN005 | HN118 |  | 138 | 23.4624 | 22 | HN007 | HN118 |
| 139 | 22.8598 | 26 | HN005 | HN140 |  | 139 | 24.7648 | 28 | HN007 | HN127 |
| 140 | 18.4096 | 23 | HN006 | HN008 |  | 140 | 25.6259 | 28 | HN007 | HN127 |
| 141 | 21.9192 | 26 | HN006 | HN010 |  | 141 | 26.299 | 28 | HN007 | HN130 |
| 142 | 18.2504 | 22 | HN006 | HN012 |  | 142 | 23.5308 | 28 | HN007 | HN131 |
| 143 | 16.8411 | 22 | HN006 | HN018 |  | 143 | 23.0944 | 24 | HN007 | HN137 |
| 144 | 17.2308 | 20 | HN006 | HN030 |  | 144 | 23.3272 | 24 | HN007 | HN137 |
| 145 | 20.1769 | 23 | HN006 | HN043 |  | 145 | 21.5479 | 21 | HN007 | HN144 |
| 146 | 15.7993 | 21 | HN006 | HN044 |  | 146 | 22.8972 | 25 | HN007 | HN146 |
| 147 | 20.1032 | 24 | HN006 | HN058 |  | 147 | 20.1776 | 20 | HN007 | HN150 |
| 148 | 23.4265 | 27 | HN006 | HN061 |  | 148 | 21.5527 | 27 | HN008 | HN009 |
| 149 | 15.2646 | 19 | HN006 | HN061 |  | 149 | 22.0608 | 27 | HN008 | HN017 |
| 150 | 20.4042 | 25 | HN006 | HN079 |  | 150 | 23.4819 | 24 | HN008 | HN021 |
| 151 | 23.382 | 25 | HN006 | HN082 |  | 151 | 20.4515 | 22 | HN008 | HN026 |
| 152 | 22.8145 | 29 | HN006 | HN083 |  | 152 | 21.3206 | 24 | HN008 | HN043 |
| 153 | 21.7739 | 25 | HN006 | HN083 |  | 153 | 17.8901 | 23 | HN008 | HN054 |
| 154 | 20.6732 | 23 | HN006 | HN094 |  | 154 | 21.7837 | 25 | HN008 | HN068 |
| 155 | 24.0232 | 26 | HN006 | HN118 |  | 155 | 18.9596 | 22 | HN008 | HN072 |
| 156 | 17.7305 | 22 | HN006 | HN125 |  | 156 | 19.16 | 25 | HN008 | HN075 |
| 157 | 20.5831 | 25 | HN006 | HN127 |  | 157 | 21.5058 | 23 | HN008 | HN080 |
| 158 | 21.3646 | 25 | HN006 | HN131 |  | 158 | 19.0454 | 22 | HN008 | HN086 |
| 159 | 20.9575 | 19 | HN007 | HN014 |  | 159 | 21.3455 | 24 | HN008 | HN087 |
| 160 | 19.7006 | 23 | HN007 | HN016 |  | 160 | 19.2122 | 24 | HN008 | HN090 |
| 161 | 24.6291 | 25 | HN007 | HN019 |  | 161 | 24.0364 | 28 | HN008 | HN096 |
| 162 | 25.0825 | 25 | HN007 | HN019 |  | 162 | 18.3285 | 22 | HN008 | HN102 |
| 163 | 22.3212 | 21 | HN007 | HN021 |  | 163 | 20.3602 | 27 | HN008 | HN105 |
| 164 | 23.4875 | 23 | HN007 | HN021 |  | 164 | 18.4691 | 24 | HN008 | HN109 |
| 165 | 18.6017 | 20 | HN007 | HN021 |  | 165 | 21.688 | 23 | HN008 | HN113 |
| 166 | 25.4709 | 22 | HN007 | HN022 |  | 166 | 17.3639 | 22 | HN008 | HN115 |
| 167 | 23.1328 | 25 | HN007 | HN024 |  | 167 | 25.5081 | 28 | HN008 | HN116 |
| 168 | 26.1657 | 28 | HN007 | HN024 |  | 168 | 23.2681 | 28 | HN008 | HN130 |
| 169 | 20.6393 | 21 | HN007 | HN030 |  | 169 | 20.7622 | 26 | HN008 | HN131 |
| 170 | 18.9853 | 21 | HN007 | HN031 |  | 170 | 21.2352 | 26 | HN008 | HN132 |
| 171 | 21.5013 | 21 | HN007 | HN032 |  | 171 | 21.2288 | 25 | HN008 | HN137 |
| 172 | 24.3046 | 24 | HN007 | HN035 |  | 172 | 19.7445 | 21 | HN008 | HN145 |
| 173 | 18.5732 | 20 | HN007 | HN036 |  | 173 | 16.8837 | 21 | HN008 | HN149 |
| 174 | 24.3341 | 26 | HN007 | HN037 |  | 174 | 23.3446 | 28 | HN009 | HN011 |
| 175 | 19.6039 | 22 | HN007 | HN040 |  | 175 | 22.4139 | 27 | HN009 | HN019 |
| 176 | 22.4133 | 23 | HN007 | HN041 |  | 176 | 24.1665 | 26 | HN009 | HN022 |
| 177 | 22.2199 | 21 | HN007 | HN043 |  | 177 | 22.8041 | 25 | HN009 | HN025 |
| 178 | 23.6818 | 24 | HN007 | HN047 |  | 178 | 20.4199 | 25 | HN009 | HN029 |
| 179 | 21.5145 | 19 | HN007 | HN049 |  | 179 | 19.9778 | 22 | HN009 | HN029 |
| 180 | 25.9261 | 27 | HN007 | HN050 |  | 180 | 20.4404 | 24 | HN009 | HN031 |
| 181 | 25.5016 | 27 | HN007 | HN050 |  | 181 | 22.052 | 24 | HN009 | HN032 |
| 182 | 25.6148 | 27 | HN007 | HN060 |  | 182 | 25.3626 | 29 | HN009 | HN034 |
| 183 | 17.549 | 23 | HN007 | HN074 |  | 183 | 21.9879 | 25 | HN009 | HN047 |
| 184 | 21.9826 | 21 | HN007 | HN077 |  | 184 | 18.8513 | 21 | HN009 | HN049 |
| 185 | 25.0324 | 27 | HN007 | HN080 |  | 185 | 25.2065 | 30 | HN009 | HN050 |
| 186 | 21.5387 | 22 | HN007 | HN082 |  | 186 | 22.5848 | 27 | HN009 | HN050 |
| 187 | 24.437 | 27 | HN007 | HN083 |  | 187 | 21.3013 | 25 | HN009 | HN053 |
| 188 | 25.1274 | 31 | HN007 | HN083 |  | 188 | 20.4501 | 25 | HN009 | HN063 |
| 189 | 22.7374 | 22 | HN007 | HN086 |  | 189 | 23.495 | 26 | HN009 | HN065 |
| 190 | 22.2078 | 24 | HN007 | HN096 |  | 190 | 23.8602 | 28 | HN009 | HN068 |
| 191 | 18.0646 | 16 | HN007 | HN097 |  | 191 | 24.5907 | 27 | HN009 | HN068 |
| 192 | 23.726 | 20 | HN007 | HN097 |  | 192 | 22.9847 | 26 | HN009 | HN071 |
| 193 | 17.8908 | 15 | HN007 | HN106 |  | 193 | 23.6708 | 27 | HN009 | HN071 |
| 194 | 22.0494 | 19 | HN007 | HN106 |  | 194 | 23.0242 | 27 | HN009 | HN072 |
| 195 | 21.0069 | 21 | HN007 | HN106 |  | 195 | 21.4342 | 26 | HN009 | HN077 |
| 196 | 21.5632 | 23 | HN007 | HN109 |  | 196 | 22.6729 | 27 | HN009 | HN080 |
| 197 | 22.5743 | 22 | HN007 | HN113 |  | 197 | 24.4996 | 28 | HN009 | HN082 |
| 198 | 19.3412 | 27 | HN007 | HN115 |  | 198 | 22.3682 | 25 | HN009 | HN084 |
| 199 | 22.5435 | 23 | HN007 | HN116 |  | 199 | 21.1705 | 25 | HN009 | HN085 |
| 200 | 21.7769 | 24 | HN007 | HN116 |  | 200 | 25.2704 | 28 | HN009 | HN087 |
| 201 | 23.9933 | 25 | HN007 | HN117 |  | 201 | 21.7536 | 23 | HN009 | HN093 |
| 202 | 23.6428 | 25 | HN007 | HN126 |  | 202 | 23.2221 | 26 | HN009 | HN105 |
| 203 | 24.1401 | 29 | HN007 | HN129 |  | 203 | 21.9914 | 27 | HN009 | HN110 |
| 204 | 23.5747 | 28 | HN007 | HN130 |  | 204 | 23.3978 | 26 | HN009 | HN112 |
| 205 | 22.8147 | 26 | HN007 | HN131 |  | 205 | 22.1572 | 26 | HN009 | HN115 |
| 206 | 20.8817 | 22 | HN007 | HN136 |  | 206 | 26.9833 | 29 | HN009 | HN116 |
| 207 | 19.3618 | 22 | HN007 | HN146 |  | 207 | 24.814 | 28 | HN009 | HN118 |
| 208 | 19.8907 | 24 | HN008 | HN035 |  | 208 | 22.0591 | 26 | HN009 | HN124 |
| 209 | 21.0748 | 23 | HN008 | HN049 |  | 209 | 22.5384 | 29 | HN009 | HN126 |
| 210 | 22.3098 | 28 | HN008 | HN050 |  | 210 | 21.9669 | 28 | HN009 | HN127 |
| 211 | 20.3302 | 24 | HN008 | HN051 |  | 211 | 23.3419 | 28 | HN009 | HN129 |
| 212 | 19.8592 | 23 | HN008 | HN054 |  | 212 | 24.5267 | 28 | HN009 | HN129 |
| 213 | 23.1501 | 29 | HN008 | HN094 |  | 213 | 22.4177 | 28 | HN009 | HN130 |
| 214 | 16.8187 | 23 | HN008 | HN101 |  | 214 | 23.603 | 28 | HN009 | HN131 |
| 215 | 18.7351 | 24 | HN008 | HN102 |  | 215 | 21.8384 | 27 | HN009 | HN137 |
| 216 | 23.1872 | 26 | HN008 | HN116 |  | 216 | 23.2049 | 27 | HN009 | HN141 |
| 217 | 19.136 | 25 | HN008 | HN125 |  | 217 | 19.8145 | 20 | HN009 | HN148 |
| 218 | 23.0947 | 24 | HN008 | HN129 |  | 218 | 21.7395 | 22 | HN010 | HN018 |
| 219 | 23.6301 | 29 | HN008 | HN130 |  | 219 | 23.6625 | 22 | HN010 | HN018 |
| 220 | 18.093 | 21 | HN008 | HN142 |  | 220 | 26.2195 | 29 | HN010 | HN043 |
| 221 | 22.9771 | 28 | HN009 | HN012 |  | 221 | 24.473 | 25 | HN010 | HN043 |
| 222 | 23.5773 | 28 | HN009 | HN018 |  | 222 | 18.6198 | 24 | HN010 | HN046 |
| 223 | 19.6636 | 22 | HN009 | HN019 |  | 223 | 19.5308 | 26 | HN010 | HN047 |
| 224 | 18.5528 | 24 | HN009 | HN019 |  | 224 | 23.5018 | 27 | HN010 | HN051 |
| 225 | 23.5117 | 24 | HN009 | HN021 |  | 225 | 22.1931 | 23 | HN010 | HN058 |
| 226 | 21.6682 | 24 | HN009 | HN022 |  | 226 | 25.8788 | 30 | HN010 | HN061 |
| 227 | 20.7262 | 28 | HN009 | HN024 |  | 227 | 20.5815 | 24 | HN010 | HN066 |
| 228 | 20.2208 | 24 | HN009 | HN025 |  | 228 | 21.3966 | 23 | HN010 | HN067 |
| 229 | 22.402 | 22 | HN009 | HN025 |  | 229 | 24.763 | 28 | HN010 | HN072 |
| 230 | 22.9616 | 26 | HN009 | HN030 |  | 230 | 23.3085 | 24 | HN010 | HN082 |
| 231 | 20.9292 | 26 | HN009 | HN033 |  | 231 | 20.5786 | 26 | HN010 | HN094 |
| 232 | 22.9919 | 28 | HN009 | HN034 |  | 232 | 25.8039 | 29 | HN010 | HN103 |
| 233 | 24.8218 | 27 | HN009 | HN034 |  | 233 | 22.7245 | 21 | HN010 | HN104 |
| 234 | 21.1436 | 26 | HN009 | HN034 |  | 234 | 25.0518 | 23 | HN010 | HN106 |
| 235 | 23.3224 | 26 | HN009 | HN034 |  | 235 | 22.3345 | 27 | HN010 | HN107 |
| 236 | 26.0025 | 27 | HN009 | HN043 |  | 236 | 21.3742 | 21 | HN010 | HN108 |
| 237 | 21.5238 | 26 | HN009 | HN046 |  | 237 | 21.2039 | 26 | HN010 | HN109 |
| 238 | 24.1385 | 28 | HN009 | HN047 |  | 238 | 23.3657 | 29 | HN010 | HN112 |
| 239 | 20.4149 | 23 | HN009 | HN048 |  | 239 | 23.8615 | 25 | HN010 | HN113 |
| 240 | 25.1969 | 30 | HN009 | HN050 |  | 240 | 23.4575 | 25 | HN010 | HN114 |
| 241 | 24.585 | 30 | HN009 | HN050 |  | 241 | 22.9225 | 24 | HN010 | HN117 |
| 242 | 22.3633 | 27 | HN009 | HN051 |  | 242 | 25.3676 | 30 | HN010 | HN120 |
| 243 | 23.7336 | 28 | HN009 | HN051 |  | 243 | 23.229 | 28 | HN010 | HN127 |
| 244 | 19.5723 | 25 | HN009 | HN056 |  | 244 | 21.6919 | 25 | HN010 | HN134 |
| 245 | 20.8186 | 26 | HN009 | HN057 |  | 245 | 25.3974 | 30 | HN010 | HN135 |
| 246 | 21.6997 | 27 | HN009 | HN060 |  | 246 | 19.9683 | 25 | HN010 | HN136 |
| 247 | 21.2947 | 24 | HN009 | HN062 |  | 247 | 19.0691 | 26 | HN010 | HN145 |
| 248 | 20.97 | 23 | HN009 | HN062 |  | 248 | 20.338 | 25 | HN010 | HN147 |
| 249 | 20.9796 | 25 | HN009 | HN064 |  | 249 | 26.1996 | 31 | HN011 | HN016 |
| 250 | 22.7179 | 28 | HN009 | HN064 |  | 250 | 17.5071 | 25 | HN011 | HN053 |
| 251 | 22.7663 | 26 | HN009 | HN068 |  | 251 | 21.1912 | 27 | HN011 | HN065 |
| 252 | 22.7435 | 25 | HN009 | HN069 |  | 252 | 24.0685 | 25 | HN011 | HN082 |
| 253 | 21.3016 | 24 | HN009 | HN069 |  | 253 | 20.3617 | 26 | HN011 | HN136 |
| 254 | 20.9801 | 26 | HN009 | HN072 |  | 254 | 18.9573 | 23 | HN011 | HN137 |
| 255 | 21.8494 | 25 | HN009 | HN073 |  | 255 | 23.3236 | 25 | HN012 | HN014 |
| 256 | 21.5156 | 25 | HN009 | HN074 |  | 256 | 26.1921 | 29 | HN012 | HN022 |
| 257 | 21.4852 | 25 | HN009 | HN075 |  | 257 | 21.674 | 26 | HN012 | HN033 |
| 258 | 18.1096 | 21 | HN009 | HN076 |  | 258 | 26.5815 | 30 | HN012 | HN034 |
| 259 | 21.0015 | 26 | HN009 | HN079 |  | 259 | 26.8235 | 30 | HN012 | HN035 |
| 260 | 23.3659 | 26 | HN009 | HN080 |  | 260 | 25.4333 | 30 | HN012 | HN039 |
| 261 | 21.5627 | 24 | HN009 | HN081 |  | 261 | 21.3135 | 25 | HN012 | HN044 |
| 262 | 23.6955 | 24 | HN009 | HN082 |  | 262 | 22.7826 | 29 | HN012 | HN051 |
| 263 | 21.6793 | 28 | HN009 | HN083 |  | 263 | 25.2613 | 29 | HN012 | HN053 |
| 264 | 20.2968 | 24 | HN009 | HN091 |  | 264 | 23.3753 | 30 | HN012 | HN054 |
| 265 | 26.3641 | 30 | HN009 | HN093 |  | 265 | 25.0059 | 28 | HN012 | HN061 |
| 266 | 25.6621 | 29 | HN009 | HN103 |  | 266 | 24.5002 | 27 | HN012 | HN064 |
| 267 | 24.4984 | 25 | HN009 | HN104 |  | 267 | 21.4449 | 24 | HN012 | HN071 |
| 268 | 21.1086 | 27 | HN009 | HN105 |  | 268 | 20.9677 | 26 | HN012 | HN072 |
| 269 | 21.5762 | 25 | HN009 | HN115 |  | 269 | 23.3953 | 24 | HN012 | HN074 |
| 270 | 20.9991 | 25 | HN009 | HN116 |  | 270 | 23.4033 | 29 | HN012 | HN095 |
| 271 | 25.9223 | 27 | HN009 | HN116 |  | 271 | 21.6776 | 27 | HN012 | HN095 |
| 272 | 22.0171 | 26 | HN009 | HN123 |  | 272 | 28.5444 | 32 | HN012 | HN096 |
| 273 | 22.8168 | 27 | HN009 | HN125 |  | 273 | 25.1763 | 21 | HN012 | HN097 |
| 274 | 24.5902 | 28 | HN009 | HN125 |  | 274 | 25.3933 | 22 | HN012 | HN097 |
| 275 | 22.2999 | 27 | HN009 | HN132 |  | 275 | 24.7079 | 20 | HN012 | HN097 |
| 276 | 20.3469 | 25 | HN009 | HN133 |  | 276 | 24.0276 | 28 | HN012 | HN107 |
| 277 | 20.0208 | 23 | HN009 | HN137 |  | 277 | 22.6513 | 28 | HN012 | HN109 |
| 278 | 23.0839 | 26 | HN009 | HN141 |  | 278 | 21.1519 | 27 | HN012 | HN109 |
| 279 | 22.0327 | 25 | HN009 | HN142 |  | 279 | 24.3131 | 29 | HN012 | HN109 |
| 280 | 21.696 | 24 | HN009 | HN148 |  | 280 | 21.0888 | 26 | HN012 | HN110 |
| 281 | 19.6717 | 23 | HN009 | HN149 |  | 281 | 24.0286 | 29 | HN012 | HN112 |
| 282 | 26.4024 | 25 | HN010 | HN014 |  | 282 | 22.6713 | 27 | HN012 | HN114 |
| 283 | 21.8651 | 25 | HN010 | HN019 |  | 283 | 25.7598 | 29 | HN012 | HN116 |
| 284 | 25.4202 | 23 | HN010 | HN022 |  | 284 | 25.4457 | 27 | HN012 | HN116 |
| 285 | 15.4808 | 21 | HN010 | HN023 |  | 285 | 26.3782 | 28 | HN012 | HN116 |
| 286 | 18.5571 | 20 | HN010 | HN025 |  | 286 | 20.6581 | 26 | HN012 | HN118 |
| 287 | 24.3972 | 26 | HN010 | HN026 |  | 287 | 23.1206 | 31 | HN012 | HN129 |
| 288 | 22.5315 | 23 | HN010 | HN032 |  | 288 | 21.6272 | 25 | HN012 | HN136 |
| 289 | 23.4615 | 25 | HN010 | HN034 |  | 289 | 25.0281 | 28 | HN012 | HN138 |
| 290 | 22.0363 | 27 | HN010 | HN035 |  | 290 | 21.9076 | 24 | HN012 | HN141 |
| 291 | 26.358 | 27 | HN010 | HN043 |  | 291 | 22.9417 | 26 | HN012 | HN143 |
| 292 | 23.7051 | 28 | HN010 | HN045 |  | 292 | 21.6553 | 25 | HN012 | HN147 |
| 293 | 20.6232 | 25 | HN010 | HN051 |  | 293 | 22.5937 | 25 | HN014 | HN017 |
| 294 | 23.5256 | 26 | HN010 | HN061 |  | 294 | 23.7264 | 25 | HN014 | HN017 |
| 295 | 25.4515 | 25 | HN010 | HN068 |  | 295 | 27.8002 | 27 | HN014 | HN026 |
| 296 | 24.1505 | 27 | HN010 | HN083 |  | 296 | 23.83 | 22 | HN014 | HN030 |
| 297 | 22.5954 | 22 | HN010 | HN084 |  | 297 | 27.3076 | 27 | HN014 | HN034 |
| 298 | 16.8338 | 23 | HN010 | HN087 |  | 298 | 22.8526 | 22 | HN014 | HN040 |
| 299 | 19.6969 | 26 | HN010 | HN094 |  | 299 | 21.5627 | 22 | HN014 | HN046 |
| 300 | 21.3208 | 25 | HN010 | HN094 |  | 300 | 25.5908 | 25 | HN014 | HN046 |
| 301 | 26.4759 | 25 | HN010 | HN097 |  | 301 | 24.5444 | 23 | HN014 | HN046 |
| 302 | 20.5881 | 24 | HN010 | HN101 |  | 302 | 22.1509 | 24 | HN014 | HN053 |
| 303 | 25.3164 | 29 | HN010 | HN105 |  | 303 | 24.6172 | 24 | HN014 | HN055 |
| 304 | 25.3164 | 29 | HN010 | HN105 |  | 304 | 23.6996 | 22 | HN014 | HN057 |
| 305 | 22.8683 | 25 | HN010 | HN108 |  | 305 | 25.4662 | 27 | HN014 | HN060 |
| 306 | 21.9882 | 27 | HN010 | HN112 |  | 306 | 22.8579 | 24 | HN014 | HN063 |
| 307 | 24.0797 | 24 | HN010 | HN113 |  | 307 | 25.3797 | 26 | HN014 | HN064 |
| 308 | 22.8526 | 21 | HN010 | HN116 |  | 308 | 24.8183 | 22 | HN014 | HN075 |
| 309 | 21.8676 | 23 | HN010 | HN116 |  | 309 | 23.9399 | 21 | HN014 | HN097 |
| 310 | 25.0903 | 26 | HN010 | HN125 |  | 310 | 23.1618 | 24 | HN014 | HN101 |
| 311 | 23.5175 | 28 | HN010 | HN127 |  | 311 | 22.8897 | 25 | HN014 | HN102 |
| 312 | 24.1241 | 28 | HN010 | HN127 |  | 312 | 25.1978 | 24 | HN014 | HN107 |
| 313 | 14.5392 | 16 | HN010 | HN134 |  | 313 | 23.7215 | 24 | HN014 | HN109 |
| 314 | 16.1088 | 20 | HN010 | HN135 |  | 314 | 25.0668 | 20 | HN014 | HN116 |
| 315 | 20.7536 | 24 | HN010 | HN135 |  | 315 | 22.8418 | 24 | HN014 | HN122 |
| 316 | 24.8772 | 25 | HN010 | HN140 |  | 316 | 23.6295 | 26 | HN014 | HN126 |
| 317 | 21.2339 | 22 | HN010 | HN143 |  | 317 | 25.7159 | 24 | HN014 | HN129 |
| 318 | 17.8339 | 20 | HN010 | HN145 |  | 318 | 27.4094 | 29 | HN014 | HN131 |
| 319 | 20.4353 | 21 | HN010 | HN148 |  | 319 | 24.1084 | 26 | HN014 | HN132 |
| 320 | 21.6655 | 27 | HN010 | HN149 |  | 320 | 21.7661 | 22 | HN014 | HN137 |
| 321 | 16.3617 | 19 | HN010 | HN150 |  | 321 | 24.435 | 23 | HN014 | HN142 |
| 322 | 25.0543 | 28 | HN011 | HN016 |  | 322 | 21.851 | 25 | HN014 | HN147 |
| 323 | 21.2494 | 24 | HN011 | HN018 |  | 323 | 20.2752 | 21 | HN015 | HN021 |
| 324 | 18.4698 | 23 | HN011 | HN020 |  | 324 | 25.1433 | 28 | HN015 | HN034 |
| 325 | 23.8515 | 23 | HN011 | HN022 |  | 325 | 23.7272 | 25 | HN015 | HN043 |
| 326 | 23.7155 | 27 | HN011 | HN022 |  | 326 | 17.4418 | 21 | HN015 | HN045 |
| 327 | 21.0148 | 24 | HN011 | HN030 |  | 327 | 18.4774 | 22 | HN015 | HN048 |
| 328 | 25.2232 | 27 | HN011 | HN034 |  | 328 | 20.9878 | 25 | HN015 | HN051 |
| 329 | 19.2692 | 25 | HN011 | HN050 |  | 329 | 20.0199 | 22 | HN015 | HN056 |
| 330 | 19.1975 | 25 | HN011 | HN054 |  | 330 | 16.5618 | 22 | HN015 | HN059 |
| 331 | 24.598 | 30 | HN011 | HN067 |  | 331 | 19.2455 | 22 | HN015 | HN072 |
| 332 | 20.4231 | 29 | HN011 | HN083 |  | 332 | 18.5728 | 23 | HN015 | HN077 |
| 333 | 21.4175 | 25 | HN011 | HN103 |  | 333 | 22.1165 | 25 | HN015 | HN096 |
| 334 | 22.1744 | 24 | HN011 | HN113 |  | 334 | 19.9208 | 24 | HN015 | HN105 |
| 335 | 21.2017 | 22 | HN011 | HN113 |  | 335 | 18.6384 | 21 | HN015 | HN108 |
| 336 | 25.0969 | 28 | HN011 | HN116 |  | 336 | 24.0629 | 24 | HN015 | HN111 |
| 337 | 23.2614 | 29 | HN011 | HN136 |  | 337 | 17.1392 | 18 | HN015 | HN128 |
| 338 | 20.7432 | 22 | HN011 | HN140 |  | 338 | 24.6627 | 28 | HN015 | HN129 |
| 339 | 21.5328 | 26 | HN012 | HN019 |  | 339 | 24.6627 | 28 | HN015 | HN129 |
| 340 | 27.0371 | 27 | HN012 | HN022 |  | 340 | 21.5416 | 25 | HN015 | HN130 |
| 341 | 27.5016 | 27 | HN012 | HN022 |  | 341 | 18.5374 | 20 | HN015 | HN139 |
| 342 | 26.2201 | 28 | HN012 | HN026 |  | 342 | 26.6906 | 27 | HN016 | HN022 |
| 343 | 27.9179 | 31 | HN012 | HN026 |  | 343 | 24.734 | 28 | HN016 | HN024 |
| 344 | 27.3945 | 29 | HN012 | HN034 |  | 344 | 21.3174 | 22 | HN016 | HN029 |
| 345 | 24.437 | 26 | HN012 | HN035 |  | 345 | 24.2798 | 26 | HN016 | HN034 |
| 346 | 24.483 | 30 | HN012 | HN037 |  | 346 | 25.1399 | 29 | HN016 | HN037 |
| 347 | 22.1379 | 27 | HN012 | HN037 |  | 347 | 22.0111 | 25 | HN016 | HN041 |
| 348 | 19.7573 | 23 | HN012 | HN040 |  | 348 | 22.2829 | 26 | HN016 | HN042 |
| 349 | 24.258 | 28 | HN012 | HN041 |  | 349 | 24.0035 | 24 | HN016 | HN043 |
| 350 | 18.2585 | 24 | HN012 | HN042 |  | 350 | 23.5254 | 28 | HN016 | HN054 |
| 351 | 21.318 | 22 | HN012 | HN048 |  | 351 | 24.898 | 28 | HN016 | HN054 |
| 352 | 23.734 | 29 | HN012 | HN051 |  | 352 | 19.9316 | 23 | HN016 | HN055 |
| 353 | 22.7019 | 27 | HN012 | HN057 |  | 353 | 20.2761 | 24 | HN016 | HN056 |
| 354 | 22.5733 | 25 | HN012 | HN058 |  | 354 | 21.2365 | 24 | HN016 | HN064 |
| 355 | 21.5996 | 26 | HN012 | HN060 |  | 355 | 22.2264 | 24 | HN016 | HN066 |
| 356 | 25.3039 | 31 | HN012 | HN065 |  | 356 | 26.7205 | 28 | HN016 | HN068 |
| 357 | 20.4911 | 25 | HN012 | HN065 |  | 357 | 26.7205 | 28 | HN016 | HN068 |
| 358 | 22.3202 | 29 | HN012 | HN065 |  | 358 | 23.7382 | 25 | HN016 | HN070 |
| 359 | 23.2459 | 28 | HN012 | HN065 |  | 359 | 25.0922 | 26 | HN016 | HN073 |
| 360 | 22.8364 | 25 | HN012 | HN067 |  | 360 | 22.8231 | 22 | HN016 | HN076 |
| 361 | 24.8943 | 30 | HN012 | HN069 |  | 361 | 27.1879 | 22 | HN016 | HN097 |
| 362 | 24.3059 | 29 | HN012 | HN073 |  | 362 | 26.0365 | 24 | HN016 | HN097 |
| 363 | 22.9286 | 25 | HN012 | HN074 |  | 363 | 24.1029 | 27 | HN016 | HN098 |
| 364 | 24.5946 | 30 | HN012 | HN079 |  | 364 | 25.3212 | 26 | HN016 | HN104 |
| 365 | 24.6762 | 28 | HN012 | HN079 |  | 365 | 23.966 | 30 | HN016 | HN105 |
| 366 | 19.8504 | 24 | HN012 | HN079 |  | 366 | 21.5389 | 25 | HN016 | HN107 |
| 367 | 20.8098 | 27 | HN012 | HN083 |  | 367 | 23.1893 | 26 | HN016 | HN112 |
| 368 | 27.1349 | 32 | HN012 | HN090 |  | 368 | 25.0196 | 27 | HN016 | HN113 |
| 369 | 23.4995 | 30 | HN012 | HN090 |  | 369 | 23.0932 | 23 | HN016 | HN116 |
| 370 | 21.6547 | 27 | HN012 | HN093 |  | 370 | 22.2949 | 26 | HN016 | HN117 |
| 371 | 26.0045 | 30 | HN012 | HN094 |  | 371 | 23.9998 | 26 | HN016 | HN130 |
| 372 | 23.1851 | 29 | HN012 | HN094 |  | 372 | 22.0479 | 26 | HN016 | HN131 |
| 373 | 24.9575 | 32 | HN012 | HN094 |  | 373 | 24.9536 | 29 | HN016 | HN137 |
| 374 | 19.4893 | 25 | HN012 | HN095 |  | 374 | 24.7994 | 27 | HN016 | HN137 |
| 375 | 26.1766 | 29 | HN012 | HN096 |  | 375 | 21.2365 | 24 | HN016 | HN143 |
| 376 | 26.9445 | 31 | HN012 | HN096 |  | 376 | 19.6411 | 23 | HN016 | HN150 |
| 377 | 26.5216 | 32 | HN012 | HN096 |  | 377 | 26.2722 | 27 | HN017 | HN021 |
| 378 | 21.2487 | 26 | HN012 | HN101 |  | 378 | 20.3891 | 26 | HN017 | HN023 |
| 379 | 20.9765 | 26 | HN012 | HN101 |  | 379 | 23.3582 | 28 | HN017 | HN025 |
| 380 | 20.9655 | 21 | HN012 | HN104 |  | 380 | 25.2758 | 28 | HN017 | HN026 |
| 381 | 23.1672 | 27 | HN012 | HN105 |  | 381 | 23.2819 | 26 | HN017 | HN032 |
| 382 | 24.3383 | 28 | HN012 | HN105 |  | 382 | 23.7096 | 30 | HN017 | HN037 |
| 383 | 23.1596 | 25 | HN012 | HN108 |  | 383 | 21.3458 | 26 | HN017 | HN039 |
| 384 | 23.3232 | 28 | HN012 | HN109 |  | 384 | 21.5524 | 26 | HN017 | HN039 |
| 385 | 24.1111 | 28 | HN012 | HN113 |  | 385 | 21.5716 | 27 | HN017 | HN047 |
| 386 | 23.4655 | 26 | HN012 | HN113 |  | 386 | 23.5703 | 28 | HN017 | HN050 |
| 387 | 21.9247 | 26 | HN012 | HN114 |  | 387 | 23.555 | 28 | HN017 | HN058 |
| 388 | 21.0022 | 24 | HN012 | HN118 |  | 388 | 19.7786 | 26 | HN017 | HN060 |
| 389 | 22.1331 | 28 | HN012 | HN118 |  | 389 | 22.9474 | 28 | HN017 | HN062 |
| 390 | 23.6583 | 26 | HN012 | HN123 |  | 390 | 24.7985 | 26 | HN017 | HN068 |
| 391 | 20.6639 | 24 | HN012 | HN124 |  | 391 | 25.3664 | 30 | HN017 | HN068 |
| 392 | 21.6623 | 25 | HN012 | HN124 |  | 392 | 25.7099 | 29 | HN017 | HN072 |
| 393 | 26.1037 | 30 | HN012 | HN125 |  | 393 | 25.6589 | 31 | HN017 | HN073 |
| 394 | 22.8079 | 27 | HN012 | HN125 |  | 394 | 24.602 | 26 | HN017 | HN073 |
| 395 | 26.7694 | 32 | HN012 | HN129 |  | 395 | 19.6436 | 21 | HN017 | HN088 |
| 396 | 22.87 | 28 | HN012 | HN129 |  | 396 | 20.8439 | 27 | HN017 | HN096 |
| 397 | 24.2884 | 30 | HN012 | HN130 |  | 397 | 24.3784 | 29 | HN017 | HN096 |
| 398 | 28.7474 | 32 | HN012 | HN131 |  | 398 | 24.1566 | 31 | HN017 | HN103 |
| 399 | 26.2734 | 31 | HN012 | HN131 |  | 399 | 24.1681 | 32 | HN017 | HN112 |
| 400 | 19.835 | 24 | HN012 | HN134 |  | 400 | 17.6241 | 24 | HN017 | HN120 |
| 401 | 18.8042 | 23 | HN012 | HN134 |  | 401 | 23.2117 | 28 | HN017 | HN124 |
| 402 | 21.1651 | 25 | HN012 | HN137 |  | 402 | 22.0023 | 27 | HN017 | HN128 |
| 403 | 22.192 | 25 | HN012 | HN137 |  | 403 | 24.8962 | 30 | HN017 | HN129 |
| 404 | 17.885 | 21 | HN012 | HN137 |  | 404 | 21.6408 | 27 | HN017 | HN132 |
| 405 | 22.5753 | 26 | HN012 | HN143 |  | 405 | 19.2515 | 25 | HN017 | HN135 |
| 406 | 22.2895 | 26 | HN012 | HN150 |  | 406 | 20.5835 | 26 | HN017 | HN136 |
| 407 | 21.6498 | 22 | HN014 | HN015 |  | 407 | 19.8214 | 22 | HN018 | HN021 |
| 408 | 25.8473 | 25 | HN014 | HN016 |  | 408 | 21.7587 | 26 | HN018 | HN024 |
| 409 | 25.1395 | 23 | HN014 | HN016 |  | 409 | 23.7226 | 26 | HN018 | HN026 |
| 410 | 24.7629 | 25 | HN014 | HN016 |  | 410 | 18.359 | 19 | HN018 | HN031 |
| 411 | 22.3026 | 23 | HN014 | HN017 |  | 411 | 28.1264 | 29 | HN018 | HN034 |
| 412 | 21.3804 | 23 | HN014 | HN017 |  | 412 | 26.6042 | 28 | HN018 | HN034 |
| 413 | 22.5899 | 21 | HN014 | HN018 |  | 413 | 16.6491 | 17 | HN018 | HN035 |
| 414 | 23.1239 | 22 | HN014 | HN021 |  | 414 | 18.1188 | 21 | HN018 | HN036 |
| 415 | 24.8257 | 24 | HN014 | HN022 |  | 415 | 24.7071 | 25 | HN018 | HN043 |
| 416 | 20.9235 | 21 | HN014 | HN025 |  | 416 | 24.9671 | 26 | HN018 | HN043 |
| 417 | 27.4504 | 25 | HN014 | HN026 |  | 417 | 18.8933 | 21 | HN018 | HN057 |
| 418 | 22.5676 | 21 | HN014 | HN026 |  | 418 | 23.049 | 25 | HN018 | HN057 |
| 419 | 26.2344 | 22 | HN014 | HN027 |  | 419 | 21.456 | 23 | HN018 | HN060 |
| 420 | 24.6829 | 22 | HN014 | HN027 |  | 420 | 21.8145 | 24 | HN018 | HN060 |
| 421 | 15.6978 | 16 | HN014 | HN029 |  | 421 | 23.1981 | 26 | HN018 | HN065 |
| 422 | 20.6861 | 20 | HN014 | HN030 |  | 422 | 21.0216 | 25 | HN018 | HN066 |
| 423 | 22.6276 | 23 | HN014 | HN034 |  | 423 | 20.8354 | 23 | HN018 | HN068 |
| 424 | 26.7174 | 23 | HN014 | HN034 |  | 424 | 17.9376 | 22 | HN018 | HN071 |
| 425 | 20.9298 | 18 | HN014 | HN037 |  | 425 | 19.6735 | 21 | HN018 | HN072 |
| 426 | 19.0801 | 17 | HN014 | HN043 |  | 426 | 21.6323 | 27 | HN018 | HN072 |
| 427 | 18.5093 | 18 | HN014 | HN044 |  | 427 | 25.4819 | 27 | HN018 | HN073 |
| 428 | 20.3948 | 23 | HN014 | HN044 |  | 428 | 24.0222 | 27 | HN018 | HN079 |
| 429 | 20.6649 | 23 | HN014 | HN045 |  | 429 | 20.9014 | 20 | HN018 | HN085 |
| 430 | 24.5752 | 29 | HN014 | HN050 |  | 430 | 25.7448 | 27 | HN018 | HN094 |
| 431 | 21.9807 | 20 | HN014 | HN051 |  | 431 | 22.3288 | 23 | HN018 | HN111 |
| 432 | 22.2387 | 25 | HN014 | HN060 |  | 432 | 19.9493 | 22 | HN018 | HN114 |
| 433 | 23.7688 | 22 | HN014 | HN061 |  | 433 | 21.7425 | 24 | HN018 | HN114 |
| 434 | 17.5636 | 20 | HN014 | HN064 |  | 434 | 22.7805 | 22 | HN018 | HN115 |
| 435 | 21.2108 | 23 | HN014 | HN067 |  | 435 | 22.6052 | 22 | HN018 | HN116 |
| 436 | 21.551 | 22 | HN014 | HN068 |  | 436 | 18.9749 | 19 | HN018 | HN122 |
| 437 | 21.3929 | 21 | HN014 | HN068 |  | 437 | 24.1653 | 29 | HN018 | HN127 |
| 438 | 22.4502 | 21 | HN014 | HN068 |  | 438 | 21.5698 | 24 | HN018 | HN130 |
| 439 | 22.8105 | 22 | HN014 | HN080 |  | 439 | 23.3287 | 24 | HN018 | HN132 |
| 440 | 17.6681 | 15 | HN014 | HN084 |  | 440 | 25.2159 | 27 | HN019 | HN020 |
| 441 | 19.5799 | 20 | HN014 | HN087 |  | 441 | 21.4083 | 24 | HN019 | HN032 |
| 442 | 19.1264 | 21 | HN014 | HN087 |  | 442 | 19.488 | 25 | HN019 | HN037 |
| 443 | 21.4978 | 24 | HN014 | HN087 |  | 443 | 17.912 | 21 | HN019 | HN049 |
| 444 | 23.448 | 25 | HN014 | HN090 |  | 444 | 22.6509 | 27 | HN019 | HN060 |
| 445 | 24.1613 | 25 | HN014 | HN093 |  | 445 | 21.1359 | 23 | HN019 | HN067 |
| 446 | 24.3535 | 22 | HN014 | HN097 |  | 446 | 19.3521 | 24 | HN019 | HN072 |
| 447 | 21.2937 | 23 | HN014 | HN098 |  | 447 | 21.9951 | 25 | HN019 | HN075 |
| 448 | 19.8908 | 19 | HN014 | HN102 |  | 448 | 21.9076 | 26 | HN019 | HN079 |
| 449 | 22.4968 | 26 | HN014 | HN103 |  | 449 | 22.942 | 26 | HN019 | HN082 |
| 450 | 20.1605 | 16 | HN014 | HN106 |  | 450 | 24.742 | 28 | HN019 | HN083 |
| 451 | 21.0544 | 17 | HN014 | HN108 |  | 451 | 21.3908 | 26 | HN019 | HN096 |
| 452 | 27.0775 | 27 | HN014 | HN114 |  | 452 | 21.3555 | 27 | HN019 | HN103 |
| 453 | 25.9561 | 25 | HN014 | HN116 |  | 453 | 19.516 | 24 | HN019 | HN105 |
| 454 | 23.1119 | 23 | HN014 | HN116 |  | 454 | 18.2636 | 23 | HN019 | HN110 |
| 455 | 22.6167 | 24 | HN014 | HN118 |  | 455 | 20.3947 | 23 | HN019 | HN117 |
| 456 | 24.1334 | 25 | HN014 | HN118 |  | 456 | 21.8672 | 27 | HN019 | HN118 |
| 457 | 26.8575 | 26 | HN014 | HN118 |  | 457 | 20.8574 | 26 | HN019 | HN131 |
| 458 | 22.2554 | 20 | HN014 | HN124 |  | 458 | 22.251 | 26 | HN019 | HN132 |
| 459 | 22.3965 | 24 | HN014 | HN124 |  | 459 | 15.7443 | 21 | HN019 | HN136 |
| 460 | 21.3084 | 25 | HN014 | HN125 |  | 460 | 24.6943 | 27 | HN020 | HN022 |
| 461 | 20.2 | 24 | HN014 | HN129 |  | 461 | 22.3501 | 24 | HN020 | HN026 |
| 462 | 26.9867 | 26 | HN014 | HN131 |  | 462 | 22.3501 | 24 | HN020 | HN026 |
| 463 | 25.126 | 25 | HN014 | HN132 |  | 463 | 23.6302 | 24 | HN020 | HN027 |
| 464 | 21.4946 | 22 | HN014 | HN135 |  | 464 | 22.7679 | 24 | HN020 | HN027 |
| 465 | 22.2386 | 23 | HN014 | HN137 |  | 465 | 20.7741 | 19 | HN020 | HN029 |
| 466 | 25.2138 | 27 | HN014 | HN143 |  | 466 | 22.5566 | 23 | HN020 | HN030 |
| 467 | 25.4652 | 25 | HN014 | HN144 |  | 467 | 23.6378 | 22 | HN020 | HN032 |
| 468 | 20.3986 | 19 | HN014 | HN148 |  | 468 | 26.4486 | 28 | HN020 | HN034 |
| 469 | 21.3537 | 21 | HN014 | HN150 |  | 469 | 22.7018 | 24 | HN020 | HN035 |
| 470 | 19.5441 | 20 | HN015 | HN018 |  | 470 | 20.6125 | 26 | HN020 | HN037 |
| 471 | 21.4247 | 23 | HN015 | HN021 |  | 471 | 24.0792 | 28 | HN020 | HN039 |
| 472 | 21.9943 | 23 | HN015 | HN022 |  | 472 | 23.2865 | 27 | HN020 | HN042 |
| 473 | 18.836 | 18 | HN015 | HN030 |  | 473 | 22.844 | 27 | HN020 | HN055 |
| 474 | 17.6589 | 20 | HN015 | HN047 |  | 474 | 21.6863 | 26 | HN020 | HN057 |
| 475 | 18.3163 | 21 | HN015 | HN052 |  | 475 | 25.517 | 27 | HN020 | HN060 |
| 476 | 20.1182 | 21 | HN015 | HN061 |  | 476 | 24.9394 | 26 | HN020 | HN060 |
| 477 | 22.7971 | 26 | HN015 | HN064 |  | 477 | 20.837 | 25 | HN020 | HN060 |
| 478 | 22.1316 | 26 | HN015 | HN068 |  | 478 | 26.7185 | 28 | HN020 | HN065 |
| 479 | 19.5151 | 24 | HN015 | HN075 |  | 479 | 23.794 | 27 | HN020 | HN068 |
| 480 | 16.1881 | 19 | HN015 | HN081 |  | 480 | 20.559 | 22 | HN020 | HN081 |
| 481 | 23.9067 | 23 | HN015 | HN082 |  | 481 | 26.4657 | 26 | HN020 | HN084 |
| 482 | 23.2766 | 27 | HN015 | HN083 |  | 482 | 21.1792 | 25 | HN020 | HN085 |
| 483 | 22.5439 | 22 | HN015 | HN097 |  | 483 | 26.0932 | 29 | HN020 | HN090 |
| 484 | 17.7442 | 22 | HN015 | HN101 |  | 484 | 25.9683 | 26 | HN020 | HN097 |
| 485 | 17.0786 | 20 | HN015 | HN102 |  | 485 | 22.6063 | 25 | HN020 | HN102 |
| 486 | 21.1232 | 23 | HN015 | HN123 |  | 486 | 21.8319 | 24 | HN020 | HN106 |
| 487 | 24.0506 | 27 | HN015 | HN123 |  | 487 | 23.2494 | 25 | HN020 | HN108 |
| 488 | 20.7352 | 22 | HN015 | HN125 |  | 488 | 20.4465 | 23 | HN020 | HN108 |
| 489 | 21.3623 | 26 | HN015 | HN125 |  | 489 | 20.9085 | 26 | HN020 | HN109 |
| 490 | 20.2954 | 25 | HN015 | HN130 |  | 490 | 21.3808 | 24 | HN020 | HN110 |
| 491 | 19.4523 | 17 | HN015 | HN140 |  | 491 | 27.7612 | 26 | HN020 | HN111 |
| 492 | 19.6526 | 23 | HN015 | HN145 |  | 492 | 20.2598 | 26 | HN020 | HN112 |
| 493 | 18.9164 | 19 | HN015 | HN148 |  | 493 | 27.3458 | 28 | HN020 | HN113 |
| 494 | 23.758 | 24 | HN016 | HN018 |  | 494 | 24.9597 | 26 | HN020 | HN114 |
| 495 | 26.0347 | 28 | HN016 | HN020 |  | 495 | 24.6908 | 27 | HN020 | HN114 |
| 496 | 23.7238 | 27 | HN016 | HN020 |  | 496 | 27.968 | 28 | HN020 | HN116 |
| 497 | 24.0293 | 24 | HN016 | HN022 |  | 497 | 22.9064 | 23 | HN020 | HN122 |
| 498 | 22.1013 | 26 | HN016 | HN023 |  | 498 | 22.327 | 23 | HN020 | HN124 |
| 499 | 20.9472 | 23 | HN016 | HN023 |  | 499 | 28.4356 | 30 | HN020 | HN125 |
| 500 | 21.8126 | 29 | HN016 | HN024 |  | 500 | 24.0183 | 29 | HN020 | HN130 |
| 501 | 24.1373 | 25 | HN016 | HN025 |  | 501 | 23.1718 | 24 | HN020 | HN139 |
| 502 | 21.3106 | 24 | HN016 | HN029 |  | 502 | 23.3354 | 25 | HN020 | HN141 |
| 503 | 20.961 | 23 | HN016 | HN029 |  | 503 | 22.9724 | 24 | HN020 | HN143 |
| 504 | 22.4146 | 24 | HN016 | HN030 |  | 504 | 21.1191 | 22 | HN020 | HN145 |
| 505 | 21.6146 | 25 | HN016 | HN034 |  | 505 | 23.6627 | 26 | HN020 | HN147 |
| 506 | 21.2218 | 24 | HN016 | HN038 |  | 506 | 25.0322 | 24 | HN021 | HN022 |
| 507 | 21.4353 | 25 | HN016 | HN040 |  | 507 | 25.2802 | 27 | HN021 | HN023 |
| 508 | 25.5647 | 24 | HN016 | HN043 |  | 508 | 27.4569 | 26 | HN021 | HN026 |
| 509 | 24.9601 | 28 | HN016 | HN043 |  | 509 | 27.4569 | 26 | HN021 | HN026 |
| 510 | 23.127 | 24 | HN016 | HN048 |  | 510 | 25.6172 | 24 | HN021 | HN027 |
| 511 | 21.6281 | 27 | HN016 | HN056 |  | 511 | 28.4892 | 29 | HN021 | HN034 |
| 512 | 20.5816 | 25 | HN016 | HN056 |  | 512 | 22.0201 | 23 | HN021 | HN045 |
| 513 | 19.0822 | 24 | HN016 | HN056 |  | 513 | 21.2707 | 25 | HN021 | HN045 |
| 514 | 19.9318 | 23 | HN016 | HN057 |  | 514 | 24.2526 | 25 | HN021 | HN045 |
| 515 | 21.8459 | 26 | HN016 | HN059 |  | 515 | 22.6845 | 22 | HN021 | HN048 |
| 516 | 21.9014 | 26 | HN016 | HN059 |  | 516 | 22.4632 | 25 | HN021 | HN054 |
| 517 | 23.6576 | 28 | HN016 | HN060 |  | 517 | 26.0091 | 29 | HN021 | HN055 |
| 518 | 24.1364 | 24 | HN016 | HN061 |  | 518 | 23.0198 | 23 | HN021 | HN057 |
| 519 | 22.6393 | 23 | HN016 | HN061 |  | 519 | 26.7232 | 27 | HN021 | HN058 |
| 520 | 23.217 | 25 | HN016 | HN062 |  | 520 | 23.9754 | 26 | HN021 | HN073 |
| 521 | 21.2365 | 24 | HN016 | HN064 |  | 521 | 19.3721 | 22 | HN021 | HN076 |
| 522 | 26.3764 | 27 | HN016 | HN065 |  | 522 | 26.528 | 27 | HN021 | HN082 |
| 523 | 23.5042 | 25 | HN016 | HN069 |  | 523 | 30.4349 | 34 | HN021 | HN083 |
| 524 | 25.7712 | 29 | HN016 | HN070 |  | 524 | 20.8724 | 24 | HN021 | HN084 |
| 525 | 18.9278 | 23 | HN016 | HN070 |  | 525 | 17.9126 | 19 | HN021 | HN092 |
| 526 | 19.7345 | 22 | HN016 | HN070 |  | 526 | 25.6765 | 28 | HN021 | HN096 |
| 527 | 19.651 | 25 | HN016 | HN071 |  | 527 | 24.0305 | 29 | HN021 | HN098 |
| 528 | 23.4697 | 26 | HN016 | HN073 |  | 528 | 23.1866 | 28 | HN021 | HN109 |
| 529 | 24.1558 | 27 | HN016 | HN073 |  | 529 | 23.5281 | 24 | HN021 | HN114 |
| 530 | 21.6188 | 24 | HN016 | HN073 |  | 530 | 22.5907 | 22 | HN021 | HN118 |
| 531 | 21.8567 | 24 | HN016 | HN075 |  | 531 | 25.808 | 30 | HN021 | HN125 |
| 532 | 22.688 | 23 | HN016 | HN076 |  | 532 | 24.8461 | 26 | HN021 | HN129 |
| 533 | 22.7209 | 23 | HN016 | HN080 |  | 533 | 22.1482 | 25 | HN021 | HN130 |
| 534 | 22.8409 | 22 | HN016 | HN082 |  | 534 | 21.8996 | 20 | HN021 | HN148 |
| 535 | 20.7351 | 24 | HN016 | HN082 |  | 535 | 24.5115 | 26 | HN021 | HN149 |
| 536 | 23.5616 | 29 | HN016 | HN083 |  | 536 | 26.55 | 25 | HN022 | HN025 |
| 537 | 24.3469 | 30 | HN016 | HN083 |  | 537 | 21.409 | 22 | HN022 | HN029 |
| 538 | 19.9836 | 24 | HN016 | HN086 |  | 538 | 27.3744 | 25 | HN022 | HN032 |
| 539 | 16.5979 | 21 | HN016 | HN087 |  | 539 | 25.5941 | 27 | HN022 | HN033 |
| 540 | 21.4336 | 23 | HN016 | HN091 |  | 540 | 23.2562 | 22 | HN022 | HN036 |
| 541 | 25.4018 | 22 | HN016 | HN097 |  | 541 | 27.0812 | 26 | HN022 | HN039 |
| 542 | 25.0699 | 27 | HN016 | HN103 |  | 542 | 28.0951 | 28 | HN022 | HN039 |
| 543 | 19.0501 | 25 | HN016 | HN105 |  | 543 | 25.547 | 27 | HN022 | HN041 |
| 544 | 21.348 | 24 | HN016 | HN107 |  | 544 | 28.506 | 26 | HN022 | HN043 |
| 545 | 21.3531 | 23 | HN016 | HN111 |  | 545 | 25.503 | 24 | HN022 | HN046 |
| 546 | 24.0645 | 25 | HN016 | HN111 |  | 546 | 25.4342 | 26 | HN022 | HN047 |
| 547 | 23.1959 | 27 | HN016 | HN111 |  | 547 | 24.8035 | 24 | HN022 | HN048 |
| 548 | 21.2657 | 22 | HN016 | HN113 |  | 548 | 24.6354 | 24 | HN022 | HN052 |
| 549 | 18.2803 | 22 | HN016 | HN113 |  | 549 | 26.3935 | 27 | HN022 | HN054 |
| 550 | 21.7545 | 25 | HN016 | HN115 |  | 550 | 21.7614 | 25 | HN022 | HN056 |
| 551 | 24.4291 | 27 | HN016 | HN125 |  | 551 | 26.0751 | 26 | HN022 | HN058 |
| 552 | 24.7633 | 28 | HN016 | HN125 |  | 552 | 24.8615 | 27 | HN022 | HN060 |
| 553 | 22.5224 | 26 | HN016 | HN126 |  | 553 | 27.5694 | 32 | HN022 | HN060 |
| 554 | 25.1113 | 28 | HN016 | HN127 |  | 554 | 27.1737 | 30 | HN022 | HN060 |
| 555 | 22.2849 | 23 | HN016 | HN129 |  | 555 | 26.1402 | 26 | HN022 | HN061 |
| 556 | 24.5557 | 28 | HN016 | HN129 |  | 556 | 23.4066 | 23 | HN022 | HN062 |
| 557 | 21.0589 | 24 | HN016 | HN132 |  | 557 | 27.0515 | 30 | HN022 | HN065 |
| 558 | 21.2895 | 24 | HN016 | HN132 |  | 558 | 25.7791 | 28 | HN022 | HN072 |
| 559 | 22.0919 | 23 | HN016 | HN141 |  | 559 | 22.1658 | 24 | HN022 | HN075 |
| 560 | 21.7098 | 26 | HN016 | HN143 |  | 560 | 24.6141 | 24 | HN022 | HN076 |
| 561 | 19.4281 | 23 | HN016 | HN146 |  | 561 | 23.6446 | 24 | HN022 | HN081 |
| 562 | 24.1948 | 28 | HN016 | HN147 |  | 562 | 25.5917 | 33 | HN022 | HN083 |
| 563 | 22.2626 | 23 | HN016 | HN148 |  | 563 | 24.9548 | 23 | HN022 | HN084 |
| 564 | 21.095 | 23 | HN017 | HN019 |  | 564 | 25.6395 | 25 | HN022 | HN085 |
| 565 | 21.4183 | 24 | HN017 | HN020 |  | 565 | 24.4389 | 22 | HN022 | HN085 |
| 566 | 18.3199 | 22 | HN017 | HN033 |  | 566 | 21.481 | 21 | HN022 | HN086 |
| 567 | 23.664 | 29 | HN017 | HN034 |  | 567 | 23.1819 | 22 | HN022 | HN087 |
| 568 | 17.5458 | 23 | HN017 | HN036 |  | 568 | 21.2126 | 23 | HN022 | HN087 |
| 569 | 24.3525 | 29 | HN017 | HN037 |  | 569 | 22.2875 | 25 | HN022 | HN091 |
| 570 | 19.6939 | 25 | HN017 | HN040 |  | 570 | 23.8868 | 22 | HN022 | HN093 |
| 571 | 21.1883 | 27 | HN017 | HN047 |  | 571 | 25.0121 | 24 | HN022 | HN098 |
| 572 | 19.994 | 22 | HN017 | HN048 |  | 572 | 24.0253 | 25 | HN022 | HN101 |
| 573 | 19.3818 | 22 | HN017 | HN049 |  | 573 | 26.96 | 26 | HN022 | HN103 |
| 574 | 23.0543 | 29 | HN017 | HN050 |  | 574 | 25.4704 | 28 | HN022 | HN105 |
| 575 | 22.1237 | 29 | HN017 | HN052 |  | 575 | 28.6187 | 26 | HN022 | HN111 |
| 576 | 18.6902 | 25 | HN017 | HN052 |  | 576 | 24.8804 | 26 | HN022 | HN118 |
| 577 | 21.4265 | 27 | HN017 | HN053 |  | 577 | 26.5295 | 26 | HN022 | HN118 |
| 578 | 20.8455 | 27 | HN017 | HN055 |  | 578 | 24.7602 | 27 | HN022 | HN121 |
| 579 | 20.687 | 24 | HN017 | HN058 |  | 579 | 25.9485 | 27 | HN022 | HN121 |
| 580 | 24.485 | 29 | HN017 | HN061 |  | 580 | 22.7154 | 24 | HN022 | HN122 |
| 581 | 22.9605 | 28 | HN017 | HN062 |  | 581 | 25.444 | 26 | HN022 | HN123 |
| 582 | 23.2279 | 28 | HN017 | HN062 |  | 582 | 25.33 | 28 | HN022 | HN129 |
| 583 | 20.8859 | 27 | HN017 | HN064 |  | 583 | 25.9743 | 32 | HN022 | HN131 |
| 584 | 25.6922 | 30 | HN017 | HN065 |  | 584 | 27.237 | 29 | HN022 | HN131 |
| 585 | 23.575 | 29 | HN017 | HN065 |  | 585 | 23.9349 | 23 | HN022 | HN138 |
| 586 | 23.976 | 29 | HN017 | HN068 |  | 586 | 26.2494 | 27 | HN022 | HN141 |
| 587 | 24.6148 | 30 | HN017 | HN072 |  | 587 | 26.0027 | 26 | HN022 | HN142 |
| 588 | 24.59 | 27 | HN017 | HN073 |  | 588 | 22.6878 | 24 | HN022 | HN142 |
| 589 | 22.3295 | 24 | HN017 | HN080 |  | 589 | 21.7318 | 25 | HN022 | HN143 |
| 590 | 22.6653 | 28 | HN017 | HN082 |  | 590 | 25.7201 | 25 | HN022 | HN144 |
| 591 | 23.4698 | 29 | HN017 | HN083 |  | 591 | 25.5171 | 29 | HN023 | HN034 |
| 592 | 19.2257 | 20 | HN017 | HN084 |  | 592 | 22.547 | 24 | HN023 | HN043 |
| 593 | 20.1375 | 24 | HN017 | HN087 |  | 593 | 20.8106 | 25 | HN023 | HN043 |
| 594 | 21.6684 | 27 | HN017 | HN090 |  | 594 | 20.2943 | 24 | HN023 | HN051 |
| 595 | 20.7475 | 26 | HN017 | HN094 |  | 595 | 18.2067 | 24 | HN023 | HN054 |
| 596 | 25.4321 | 31 | HN017 | HN096 |  | 596 | 18.7222 | 23 | HN023 | HN062 |
| 597 | 25.1822 | 30 | HN017 | HN096 |  | 597 | 19.8455 | 26 | HN023 | HN072 |
| 598 | 24.2217 | 26 | HN017 | HN097 |  | 598 | 19.2303 | 26 | HN023 | HN103 |
| 599 | 22.3538 | 20 | HN017 | HN097 |  | 599 | 20.8138 | 24 | HN023 | HN116 |
| 600 | 26.066 | 31 | HN017 | HN103 |  | 600 | 22.8955 | 26 | HN023 | HN118 |
| 601 | 20.8861 | 25 | HN017 | HN109 |  | 601 | 23.4923 | 27 | HN023 | HN131 |
| 602 | 23.6827 | 26 | HN017 | HN111 |  | 602 | 25.3548 | 27 | HN024 | HN027 |
| 603 | 19.8571 | 25 | HN017 | HN112 |  | 603 | 20.2459 | 24 | HN024 | HN030 |
| 604 | 24.1977 | 24 | HN017 | HN113 |  | 604 | 18.0331 | 22 | HN024 | HN031 |
| 605 | 24.2617 | 28 | HN017 | HN113 |  | 605 | 20.0745 | 23 | HN024 | HN044 |
| 606 | 23.6569 | 26 | HN017 | HN113 |  | 606 | 21.8792 | 25 | HN024 | HN048 |
| 607 | 27.0882 | 28 | HN017 | HN116 |  | 607 | 22.0508 | 28 | HN024 | HN051 |
| 608 | 25.6626 | 30 | HN017 | HN116 |  | 608 | 23.3803 | 30 | HN024 | HN053 |
| 609 | 24.7243 | 26 | HN017 | HN116 |  | 609 | 21.5499 | 28 | HN024 | HN054 |
| 610 | 27.0882 | 28 | HN017 | HN116 |  | 610 | 23.1179 | 27 | HN024 | HN058 |
| 611 | 21.9513 | 28 | HN017 | HN117 |  | 611 | 18.8538 | 25 | HN024 | HN060 |
| 612 | 20.5966 | 26 | HN017 | HN118 |  | 612 | 23.9189 | 26 | HN024 | HN068 |
| 613 | 23.2216 | 27 | HN017 | HN122 |  | 613 | 19.0783 | 22 | HN024 | HN081 |
| 614 | 20.8812 | 25 | HN017 | HN122 |  | 614 | 25.8827 | 28 | HN024 | HN082 |
| 615 | 22.9852 | 28 | HN017 | HN123 |  | 615 | 21.2726 | 28 | HN024 | HN094 |
| 616 | 22.7071 | 29 | HN017 | HN125 |  | 616 | 18.2878 | 23 | HN024 | HN101 |
| 617 | 18.9934 | 25 | HN017 | HN125 |  | 617 | 22.9114 | 28 | HN024 | HN105 |
| 618 | 22.6909 | 29 | HN017 | HN127 |  | 618 | 21.0908 | 21 | HN024 | HN106 |
| 619 | 20.0278 | 24 | HN017 | HN128 |  | 619 | 21.2668 | 25 | HN024 | HN111 |
| 620 | 24.2504 | 30 | HN017 | HN131 |  | 620 | 22.157 | 27 | HN024 | HN115 |
| 621 | 21.3895 | 26 | HN017 | HN131 |  | 621 | 20.1344 | 27 | HN024 | HN120 |
| 622 | 18.2779 | 23 | HN017 | HN133 |  | 622 | 21.0418 | 23 | HN024 | HN123 |
| 623 | 20.7753 | 27 | HN017 | HN136 |  | 623 | 20.8484 | 27 | HN024 | HN124 |
| 624 | 21.865 | 25 | HN017 | HN140 |  | 624 | 20.3878 | 28 | HN024 | HN134 |
| 625 | 20.0946 | 24 | HN017 | HN148 |  | 625 | 20.9411 | 26 | HN024 | HN134 |
| 626 | 19.9572 | 24 | HN017 | HN150 |  | 626 | 21.6327 | 25 | HN024 | HN144 |
| 627 | 18.7457 | 22 | HN018 | HN021 |  | 627 | 21.7931 | 19 | HN025 | HN032 |
| 628 | 20.9693 | 21 | HN018 | HN021 |  | 628 | 21.0352 | 23 | HN025 | HN039 |
| 629 | 19.9291 | 18 | HN018 | HN027 |  | 629 | 21.7938 | 22 | HN025 | HN049 |
| 630 | 19.8646 | 19 | HN018 | HN027 |  | 630 | 19.8662 | 27 | HN025 | HN063 |
| 631 | 25.2296 | 23 | HN018 | HN027 |  | 631 | 23.6512 | 24 | HN025 | HN066 |
| 632 | 20.399 | 21 | HN018 | HN032 |  | 632 | 25.8454 | 26 | HN025 | HN073 |
| 633 | 25.9823 | 28 | HN018 | HN039 |  | 633 | 17.3377 | 17 | HN025 | HN084 |
| 634 | 25.1734 | 27 | HN018 | HN039 |  | 634 | 22.1353 | 22 | HN025 | HN097 |
| 635 | 24.5535 | 26 | HN018 | HN058 |  | 635 | 14.1859 | 18 | HN025 | HN100 |
| 636 | 21.3598 | 26 | HN018 | HN060 |  | 636 | 19.4355 | 22 | HN025 | HN101 |
| 637 | 21.873 | 22 | HN018 | HN061 |  | 637 | 23.8082 | 24 | HN025 | HN118 |
| 638 | 23.1962 | 24 | HN018 | HN061 |  | 638 | 22.5042 | 27 | HN025 | HN131 |
| 639 | 20.7034 | 22 | HN018 | HN064 |  | 639 | 21.0045 | 22 | HN026 | HN027 |
| 640 | 20.4891 | 22 | HN018 | HN067 |  | 640 | 21.1469 | 22 | HN026 | HN027 |
| 641 | 18.9598 | 19 | HN018 | HN070 |  | 641 | 23.9355 | 23 | HN026 | HN030 |
| 642 | 26.9462 | 30 | HN018 | HN073 |  | 642 | 23.4141 | 23 | HN026 | HN030 |
| 643 | 20.8908 | 26 | HN018 | HN079 |  | 643 | 24.4285 | 24 | HN026 | HN030 |
| 644 | 19.6766 | 22 | HN018 | HN080 |  | 644 | 20.9495 | 21 | HN026 | HN036 |
| 645 | 22.6377 | 25 | HN018 | HN082 |  | 645 | 24.4756 | 24 | HN026 | HN038 |
| 646 | 25.0527 | 28 | HN018 | HN083 |  | 646 | 23.3269 | 23 | HN026 | HN039 |
| 647 | 18.5097 | 20 | HN018 | HN085 |  | 647 | 26.8432 | 25 | HN026 | HN043 |
| 648 | 22.3653 | 24 | HN018 | HN087 |  | 648 | 24.6515 | 27 | HN026 | HN046 |
| 649 | 19.4761 | 21 | HN018 | HN093 |  | 649 | 23.5103 | 24 | HN026 | HN047 |
| 650 | 26.6219 | 30 | HN018 | HN094 |  | 650 | 21.0871 | 26 | HN026 | HN054 |
| 651 | 22.6846 | 25 | HN018 | HN096 |  | 651 | 22.8478 | 30 | HN026 | HN055 |
| 652 | 20.8923 | 23 | HN018 | HN101 |  | 652 | 24.7488 | 27 | HN026 | HN057 |
| 653 | 18.1959 | 21 | HN018 | HN108 |  | 653 | 25.0578 | 26 | HN026 | HN060 |
| 654 | 21.395 | 22 | HN018 | HN111 |  | 654 | 24.4923 | 25 | HN026 | HN061 |
| 655 | 23.7329 | 25 | HN018 | HN115 |  | 655 | 23.8175 | 26 | HN026 | HN061 |
| 656 | 24.9777 | 27 | HN018 | HN116 |  | 656 | 23.8029 | 23 | HN026 | HN066 |
| 657 | 24.5474 | 26 | HN018 | HN116 |  | 657 | 27.1091 | 27 | HN026 | HN068 |
| 658 | 22.9004 | 23 | HN018 | HN118 |  | 658 | 24.7593 | 30 | HN026 | HN072 |
| 659 | 17.1103 | 19 | HN018 | HN122 |  | 659 | 23.2154 | 24 | HN026 | HN075 |
| 660 | 22.7221 | 27 | HN018 | HN125 |  | 660 | 24.9185 | 25 | HN026 | HN080 |
| 661 | 21.6201 | 25 | HN018 | HN125 |  | 661 | 23.5992 | 24 | HN026 | HN081 |
| 662 | 20.9617 | 23 | HN018 | HN126 |  | 662 | 20.9097 | 24 | HN026 | HN081 |
| 663 | 22.8755 | 26 | HN018 | HN130 |  | 663 | 25.1079 | 30 | HN026 | HN083 |
| 664 | 24.6348 | 27 | HN018 | HN131 |  | 664 | 27.2551 | 32 | HN026 | HN083 |
| 665 | 22.1792 | 25 | HN018 | HN132 |  | 665 | 26.6518 | 26 | HN026 | HN087 |
| 666 | 22.0142 | 25 | HN018 | HN136 |  | 666 | 25.5034 | 26 | HN026 | HN087 |
| 667 | 22.0703 | 26 | HN018 | HN138 |  | 667 | 23.8885 | 21 | HN026 | HN092 |
| 668 | 19.8453 | 21 | HN018 | HN149 |  | 668 | 22.98 | 24 | HN026 | HN093 |
| 669 | 23.3604 | 27 | HN019 | HN034 |  | 669 | 24.289 | 29 | HN026 | HN094 |
| 670 | 20.4044 | 25 | HN019 | HN050 |  | 670 | 22.1775 | 20 | HN026 | HN097 |
| 671 | 23.6393 | 26 | HN019 | HN060 |  | 671 | 23.1201 | 24 | HN026 | HN101 |
| 672 | 21.5452 | 26 | HN019 | HN060 |  | 672 | 23.0702 | 25 | HN026 | HN101 |
| 673 | 14.9977 | 21 | HN019 | HN062 |  | 673 | 28.2508 | 30 | HN026 | HN103 |
| 674 | 20.3991 | 23 | HN019 | HN073 |  | 674 | 21.6208 | 22 | HN026 | HN110 |
| 675 | 25.7931 | 26 | HN019 | HN082 |  | 675 | 26.15 | 28 | HN026 | HN112 |
| 676 | 22.8623 | 28 | HN019 | HN083 |  | 676 | 27.0037 | 24 | HN026 | HN113 |
| 677 | 21.8922 | 27 | HN019 | HN083 |  | 677 | 23.904 | 23 | HN026 | HN114 |
| 678 | 23.9824 | 28 | HN019 | HN083 |  | 678 | 22.3241 | 26 | HN026 | HN115 |
| 679 | 19.7612 | 22 | HN019 | HN092 |  | 679 | 23.9741 | 25 | HN026 | HN116 |
| 680 | 20.8333 | 25 | HN019 | HN093 |  | 680 | 24.9303 | 26 | HN026 | HN119 |
| 681 | 21.3914 | 27 | HN019 | HN094 |  | 681 | 24.1303 | 25 | HN026 | HN122 |
| 682 | 19.8474 | 22 | HN019 | HN110 |  | 682 | 25.9811 | 27 | HN026 | HN123 |
| 683 | 21.2472 | 22 | HN019 | HN111 |  | 683 | 26.7432 | 30 | HN026 | HN125 |
| 684 | 22.7342 | 25 | HN019 | HN116 |  | 684 | 22.7894 | 25 | HN026 | HN126 |
| 685 | 23.8788 | 27 | HN019 | HN116 |  | 685 | 26.0177 | 28 | HN026 | HN130 |
| 686 | 23.3708 | 27 | HN019 | HN116 |  | 686 | 25.241 | 25 | HN026 | HN131 |
| 687 | 21.8644 | 25 | HN019 | HN138 |  | 687 | 24.4244 | 25 | HN026 | HN134 |
| 688 | 15.9317 | 20 | HN019 | HN140 |  | 688 | 17.8872 | 18 | HN026 | HN134 |
| 689 | 21.3427 | 23 | HN019 | HN143 |  | 689 | 23.7419 | 25 | HN026 | HN145 |
| 690 | 23.6808 | 25 | HN020 | HN021 |  | 690 | 23.5661 | 22 | HN026 | HN148 |
| 691 | 23.0494 | 25 | HN020 | HN021 |  | 691 | 25.7254 | 25 | HN026 | HN149 |
| 692 | 23.7867 | 24 | HN020 | HN021 |  | 692 | 22.4438 | 23 | HN026 | HN150 |
| 693 | 26.478 | 26 | HN020 | HN022 |  | 693 | 18.9538 | 22 | HN027 | HN029 |
| 694 | 20.1171 | 23 | HN020 | HN023 |  | 694 | 20.455 | 22 | HN027 | HN040 |
| 695 | 19.8448 | 23 | HN020 | HN023 |  | 695 | 20.3302 | 21 | HN027 | HN045 |
| 696 | 21.9818 | 28 | HN020 | HN023 |  | 696 | 21.7449 | 23 | HN027 | HN058 |
| 697 | 22.2324 | 26 | HN020 | HN024 |  | 697 | 23.3132 | 23 | HN027 | HN065 |
| 698 | 20.5316 | 20 | HN020 | HN031 |  | 698 | 22.7669 | 23 | HN027 | HN076 |
| 699 | 22.7626 | 27 | HN020 | HN033 |  | 699 | 23.859 | 24 | HN027 | HN080 |
| 700 | 25.9009 | 28 | HN020 | HN034 |  | 700 | 22.1789 | 22 | HN027 | HN084 |
| 701 | 21.4595 | 24 | HN020 | HN035 |  | 701 | 19.8579 | 25 | HN027 | HN086 |
| 702 | 21.3755 | 23 | HN020 | HN036 |  | 702 | 21.4894 | 23 | HN027 | HN098 |
| 703 | 25.0177 | 25 | HN020 | HN039 |  | 703 | 17.1728 | 18 | HN027 | HN100 |
| 704 | 22.188 | 23 | HN020 | HN040 |  | 704 | 17.083 | 21 | HN027 | HN102 |
| 705 | 20.8858 | 23 | HN020 | HN041 |  | 705 | 20.7394 | 22 | HN027 | HN102 |
| 706 | 22.4346 | 24 | HN020 | HN041 |  | 706 | 22.7003 | 26 | HN027 | HN105 |
| 707 | 25.9696 | 24 | HN020 | HN043 |  | 707 | 22.2184 | 25 | HN027 | HN112 |
| 708 | 23.9076 | 26 | HN020 | HN045 |  | 708 | 21.3715 | 20 | HN027 | HN113 |
| 709 | 21.4622 | 21 | HN020 | HN048 |  | 709 | 20.3026 | 23 | HN027 | HN114 |
| 710 | 19.9895 | 22 | HN020 | HN054 |  | 710 | 27.1836 | 26 | HN027 | HN116 |
| 711 | 23.7019 | 27 | HN020 | HN054 |  | 711 | 20.1183 | 20 | HN027 | HN123 |
| 712 | 18.7167 | 24 | HN020 | HN055 |  | 712 | 21.4385 | 22 | HN027 | HN128 |
| 713 | 24.7098 | 26 | HN020 | HN055 |  | 713 | 20.95 | 22 | HN027 | HN136 |
| 714 | 22.1263 | 26 | HN020 | HN057 |  | 714 | 20.3312 | 23 | HN027 | HN136 |
| 715 | 23.7328 | 26 | HN020 | HN058 |  | 715 | 21.2389 | 27 | HN027 | HN147 |
| 716 | 22.7561 | 28 | HN020 | HN060 |  | 716 | 20.6022 | 21 | HN027 | HN148 |
| 717 | 23.6703 | 25 | HN020 | HN061 |  | 717 | 22.9649 | 22 | HN027 | HN149 |
| 718 | 22.9276 | 26 | HN020 | HN064 |  | 718 | 19.0927 | 19 | HN029 | HN043 |
| 719 | 26.1421 | 30 | HN020 | HN065 |  | 719 | 21.1133 | 21 | HN029 | HN073 |
| 720 | 22.1695 | 24 | HN020 | HN066 |  | 720 | 25.6848 | 26 | HN029 | HN082 |
| 721 | 21.7237 | 25 | HN020 | HN066 |  | 721 | 16.9893 | 17 | HN029 | HN084 |
| 722 | 23.0195 | 26 | HN020 | HN067 |  | 722 | 16.8479 | 17 | HN029 | HN138 |
| 723 | 24.8008 | 26 | HN020 | HN067 |  | 723 | 17.7405 | 23 | HN029 | HN138 |
| 724 | 24.4175 | 25 | HN020 | HN067 |  | 724 | 25.0098 | 24 | HN030 | HN034 |
| 725 | 21.5838 | 25 | HN020 | HN067 |  | 725 | 23.4973 | 25 | HN030 | HN039 |
| 726 | 26.0008 | 28 | HN020 | HN068 |  | 726 | 20.5804 | 20 | HN030 | HN043 |
| 727 | 23.0472 | 26 | HN020 | HN068 |  | 727 | 18.851 | 22 | HN030 | HN044 |
| 728 | 19.5388 | 21 | HN020 | HN069 |  | 728 | 22.7844 | 26 | HN030 | HN051 |
| 729 | 19.887 | 23 | HN020 | HN070 |  | 729 | 21.4542 | 23 | HN030 | HN058 |
| 730 | 24.6527 | 27 | HN020 | HN072 |  | 730 | 24.0113 | 24 | HN030 | HN061 |
| 731 | 25.6411 | 28 | HN020 | HN072 |  | 731 | 17.6706 | 17 | HN030 | HN070 |
| 732 | 16.7142 | 20 | HN020 | HN076 |  | 732 | 24.8682 | 25 | HN030 | HN073 |
| 733 | 27.0339 | 29 | HN020 | HN080 |  | 733 | 16.6264 | 17 | HN030 | HN076 |
| 734 | 17.8932 | 20 | HN020 | HN080 |  | 734 | 26.6547 | 27 | HN030 | HN082 |
| 735 | 21.433 | 22 | HN020 | HN081 |  | 735 | 25.0727 | 26 | HN030 | HN090 |
| 736 | 22.5605 | 24 | HN020 | HN081 |  | 736 | 25.5692 | 28 | HN030 | HN094 |
| 737 | 23.0966 | 25 | HN020 | HN082 |  | 737 | 21.8835 | 20 | HN030 | HN098 |
| 738 | 26.8463 | 24 | HN020 | HN082 |  | 738 | 20.7777 | 20 | HN030 | HN101 |
| 739 | 22.8619 | 25 | HN020 | HN083 |  | 739 | 16.7629 | 18 | HN030 | HN102 |
| 740 | 25.3991 | 28 | HN020 | HN083 |  | 740 | 20.6707 | 22 | HN030 | HN125 |
| 741 | 25.8701 | 29 | HN020 | HN083 |  | 741 | 18.2955 | 20 | HN030 | HN133 |
| 742 | 23.051 | 24 | HN020 | HN085 |  | 742 | 17.0708 | 19 | HN030 | HN134 |
| 743 | 26.5995 | 30 | HN020 | HN094 |  | 743 | 20.3426 | 21 | HN030 | HN137 |
| 744 | 19.7888 | 28 | HN020 | HN096 |  | 744 | 17.6946 | 16 | HN030 | HN148 |
| 745 | 23.5791 | 27 | HN020 | HN096 |  | 745 | 16.735 | 18 | HN030 | HN150 |
| 746 | 25.6817 | 29 | HN020 | HN098 |  | 746 | 25.6115 | 28 | HN031 | HN034 |
| 747 | 21.971 | 25 | HN020 | HN102 |  | 747 | 17.9086 | 21 | HN031 | HN037 |
| 748 | 21.4065 | 27 | HN020 | HN103 |  | 748 | 19.0934 | 26 | HN031 | HN045 |
| 749 | 24.237 | 28 | HN020 | HN105 |  | 749 | 21.0621 | 23 | HN031 | HN060 |
| 750 | 23.8052 | 26 | HN020 | HN107 |  | 750 | 19.2572 | 24 | HN031 | HN079 |
| 751 | 24.3618 | 27 | HN020 | HN112 |  | 751 | 19.3387 | 21 | HN031 | HN080 |
| 752 | 28.8274 | 30 | HN020 | HN116 |  | 752 | 16.1377 | 16 | HN031 | HN097 |
| 753 | 25.5983 | 27 | HN020 | HN116 |  | 753 | 17.6845 | 21 | HN031 | HN098 |
| 754 | 23.8042 | 25 | HN020 | HN120 |  | 754 | 19.0827 | 18 | HN031 | HN104 |
| 755 | 22.6163 | 24 | HN020 | HN124 |  | 755 | 21.6509 | 25 | HN031 | HN105 |
| 756 | 19.327 | 23 | HN020 | HN126 |  | 756 | 21.3145 | 25 | HN031 | HN108 |
| 757 | 22.5781 | 28 | HN020 | HN129 |  | 757 | 16.2361 | 20 | HN031 | HN110 |
| 758 | 24.8349 | 27 | HN020 | HN130 |  | 758 | 16.5122 | 18 | HN031 | HN120 |
| 759 | 24.8489 | 30 | HN020 | HN130 |  | 759 | 17.7467 | 21 | HN031 | HN124 |
| 760 | 23.3423 | 24 | HN020 | HN132 |  | 760 | 25.3275 | 28 | HN031 | HN129 |
| 761 | 25.6212 | 30 | HN020 | HN137 |  | 761 | 21.0543 | 24 | HN031 | HN129 |
| 762 | 23.3797 | 22 | HN020 | HN140 |  | 762 | 19.0282 | 24 | HN031 | HN130 |
| 763 | 20.1329 | 23 | HN020 | HN147 |  | 763 | 20.7798 | 23 | HN032 | HN059 |
| 764 | 23.8145 | 26 | HN020 | HN149 |  | 764 | 17.9698 | 22 | HN032 | HN063 |
| 765 | 25.9693 | 26 | HN021 | HN022 |  | 765 | 23.2823 | 25 | HN032 | HN064 |
| 766 | 20.7149 | 20 | HN021 | HN032 |  | 766 | 26.9582 | 24 | HN032 | HN073 |
| 767 | 28.3252 | 26 | HN021 | HN034 |  | 767 | 19.8803 | 20 | HN032 | HN091 |
| 768 | 23.5011 | 24 | HN021 | HN034 |  | 768 | 24.0139 | 25 | HN032 | HN094 |
| 769 | 23.6789 | 25 | HN021 | HN035 |  | 769 | 20.4714 | 20 | HN032 | HN095 |
| 770 | 19.6607 | 20 | HN021 | HN036 |  | 770 | 25.1185 | 27 | HN032 | HN098 |
| 771 | 17.518 | 21 | HN021 | HN042 |  | 771 | 19.8399 | 20 | HN032 | HN101 |
| 772 | 23.2538 | 26 | HN021 | HN051 |  | 772 | 21.8408 | 26 | HN032 | HN103 |
| 773 | 21.9618 | 25 | HN021 | HN051 |  | 773 | 22.4111 | 24 | HN032 | HN104 |
| 774 | 27.2927 | 28 | HN021 | HN058 |  | 774 | 22.1052 | 23 | HN032 | HN108 |
| 775 | 23.7795 | 26 | HN021 | HN061 |  | 775 | 20.5219 | 23 | HN032 | HN114 |
| 776 | 20.5009 | 23 | HN021 | HN064 |  | 776 | 23.6637 | 24 | HN032 | HN116 |
| 777 | 23.7364 | 27 | HN021 | HN065 |  | 777 | 19.5438 | 20 | HN032 | HN121 |
| 778 | 18.5609 | 19 | HN021 | HN066 |  | 778 | 22.9186 | 25 | HN032 | HN125 |
| 779 | 24.7576 | 25 | HN021 | HN068 |  | 779 | 18.8727 | 20 | HN032 | HN126 |
| 780 | 22.0157 | 23 | HN021 | HN072 |  | 780 | 23.4779 | 23 | HN032 | HN140 |
| 781 | 26.3402 | 29 | HN021 | HN073 |  | 781 | 21.4985 | 20 | HN032 | HN143 |
| 782 | 26.122 | 24 | HN021 | HN073 |  | 782 | 17.5814 | 22 | HN032 | HN144 |
| 783 | 22.1613 | 26 | HN021 | HN080 |  | 783 | 23.2557 | 25 | HN032 | HN147 |
| 784 | 23.8215 | 25 | HN021 | HN080 |  | 784 | 21.8025 | 25 | HN033 | HN039 |
| 785 | 23.8215 | 25 | HN021 | HN080 |  | 785 | 22.4333 | 26 | HN033 | HN039 |
| 786 | 27.2204 | 27 | HN021 | HN082 |  | 786 | 18.8043 | 22 | HN033 | HN047 |
| 787 | 28.6179 | 27 | HN021 | HN082 |  | 787 | 22.7354 | 26 | HN033 | HN065 |
| 788 | 23.9934 | 28 | HN021 | HN083 |  | 788 | 23.8756 | 24 | HN033 | HN073 |
| 789 | 20.9958 | 21 | HN021 | HN085 |  | 789 | 19.6952 | 22 | HN033 | HN075 |
| 790 | 21.4285 | 27 | HN021 | HN103 |  | 790 | 18.6865 | 21 | HN033 | HN091 |
| 791 | 18.6564 | 20 | HN021 | HN104 |  | 791 | 21.1218 | 24 | HN033 | HN093 |
| 792 | 26.3279 | 31 | HN021 | HN105 |  | 792 | 22.5264 | 27 | HN033 | HN094 |
| 793 | 26.1576 | 28 | HN021 | HN105 |  | 793 | 23.9355 | 21 | HN033 | HN097 |
| 794 | 23.4188 | 23 | HN021 | HN113 |  | 794 | 20.8452 | 25 | HN033 | HN108 |
| 795 | 23.6453 | 27 | HN021 | HN113 |  | 795 | 21.1856 | 24 | HN033 | HN112 |
| 796 | 25.3028 | 26 | HN021 | HN113 |  | 796 | 24.4437 | 28 | HN033 | HN125 |
| 797 | 24.8662 | 26 | HN021 | HN116 |  | 797 | 19.0091 | 24 | HN033 | HN126 |
| 798 | 27.2258 | 27 | HN021 | HN116 |  | 798 | 17.5575 | 19 | HN033 | HN134 |
| 799 | 24.8585 | 24 | HN021 | HN116 |  | 799 | 18.369 | 24 | HN033 | HN146 |
| 800 | 24.1383 | 25 | HN021 | HN122 |  | 800 | 22.8863 | 25 | HN034 | HN035 |
| 801 | 24.5104 | 26 | HN021 | HN126 |  | 801 | 23.1185 | 26 | HN034 | HN036 |
| 802 | 24.559 | 29 | HN021 | HN127 |  | 802 | 26.5823 | 29 | HN034 | HN037 |
| 803 | 21.5322 | 23 | HN021 | HN128 |  | 803 | 23.736 | 26 | HN034 | HN040 |
| 804 | 21.0984 | 24 | HN021 | HN129 |  | 804 | 23.9698 | 25 | HN034 | HN044 |
| 805 | 27.7175 | 31 | HN021 | HN129 |  | 805 | 21.3181 | 22 | HN034 | HN048 |
| 806 | 25.2181 | 27 | HN021 | HN129 |  | 806 | 22.8845 | 24 | HN034 | HN049 |
| 807 | 21.1355 | 24 | HN021 | HN133 |  | 807 | 24.4229 | 28 | HN034 | HN050 |
| 808 | 24.3391 | 25 | HN021 | HN133 |  | 808 | 25.9724 | 28 | HN034 | HN051 |
| 809 | 24.2993 | 26 | HN021 | HN137 |  | 809 | 25.5924 | 28 | HN034 | HN054 |
| 810 | 21.0608 | 22 | HN021 | HN138 |  | 810 | 24.9924 | 28 | HN034 | HN055 |
| 811 | 21.6081 | 25 | HN021 | HN138 |  | 811 | 24.6884 | 28 | HN034 | HN059 |
| 812 | 19.1813 | 20 | HN021 | HN139 |  | 812 | 25.1772 | 29 | HN034 | HN059 |
| 813 | 23.9644 | 25 | HN022 | HN023 |  | 813 | 27.1252 | 28 | HN034 | HN060 |
| 814 | 23.3157 | 26 | HN022 | HN023 |  | 814 | 25.9162 | 28 | HN034 | HN061 |
| 815 | 26.062 | 28 | HN022 | HN024 |  | 815 | 25.2531 | 27 | HN034 | HN062 |
| 816 | 26.2333 | 28 | HN022 | HN024 |  | 816 | 24.3184 | 26 | HN034 | HN064 |
| 817 | 26.0027 | 26 | HN022 | HN024 |  | 817 | 26.254 | 28 | HN034 | HN065 |
| 818 | 25.4076 | 27 | HN022 | HN026 |  | 818 | 24.7726 | 28 | HN034 | HN065 |
| 819 | 23.0133 | 22 | HN022 | HN026 |  | 819 | 25.8253 | 28 | HN034 | HN067 |
| 820 | 22.0689 | 18 | HN022 | HN027 |  | 820 | 24.8181 | 25 | HN034 | HN069 |
| 821 | 21.0007 | 18 | HN022 | HN027 |  | 821 | 27.4816 | 31 | HN034 | HN069 |
| 822 | 24.9209 | 23 | HN022 | HN030 |  | 822 | 27.513 | 29 | HN034 | HN071 |
| 823 | 24.4149 | 23 | HN022 | HN030 |  | 823 | 26.7579 | 25 | HN034 | HN073 |
| 824 | 26.3783 | 25 | HN022 | HN032 |  | 824 | 25.1291 | 27 | HN034 | HN074 |
| 825 | 22.5566 | 21 | HN022 | HN032 |  | 825 | 25.7406 | 26 | HN034 | HN075 |
| 826 | 24.5721 | 24 | HN022 | HN032 |  | 826 | 22.1646 | 28 | HN034 | HN079 |
| 827 | 25.3267 | 27 | HN022 | HN033 |  | 827 | 22.1607 | 27 | HN034 | HN079 |
| 828 | 23.0492 | 24 | HN022 | HN035 |  | 828 | 22.6165 | 25 | HN034 | HN082 |
| 829 | 22.3717 | 22 | HN022 | HN035 |  | 829 | 23.7061 | 27 | HN034 | HN090 |
| 830 | 26.387 | 27 | HN022 | HN036 |  | 830 | 21.8966 | 23 | HN034 | HN093 |
| 831 | 26.6022 | 28 | HN022 | HN037 |  | 831 | 25.1558 | 29 | HN034 | HN094 |
| 832 | 20.8732 | 22 | HN022 | HN039 |  | 832 | 26.4055 | 32 | HN034 | HN103 |
| 833 | 25.6612 | 29 | HN022 | HN039 |  | 833 | 24.3568 | 27 | HN034 | HN105 |
| 834 | 25.009 | 27 | HN022 | HN040 |  | 834 | 21.194 | 25 | HN034 | HN108 |
| 835 | 19.5024 | 23 | HN022 | HN041 |  | 835 | 29.9455 | 29 | HN034 | HN111 |
| 836 | 26.522 | 26 | HN022 | HN041 |  | 836 | 24.2552 | 24 | HN034 | HN111 |
| 837 | 22.9071 | 24 | HN022 | HN041 |  | 837 | 26.1058 | 25 | HN034 | HN113 |
| 838 | 19.7093 | 18 | HN022 | HN041 |  | 838 | 27.1644 | 27 | HN034 | HN113 |
| 839 | 23.3887 | 24 | HN022 | HN042 |  | 839 | 26.3584 | 28 | HN034 | HN116 |
| 840 | 26.3328 | 26 | HN022 | HN045 |  | 840 | 23.5635 | 27 | HN034 | HN122 |
| 841 | 21.7901 | 27 | HN022 | HN045 |  | 841 | 25.3341 | 26 | HN034 | HN123 |
| 842 | 25.5081 | 28 | HN022 | HN046 |  | 842 | 26.7119 | 29 | HN034 | HN125 |
| 843 | 22.114 | 26 | HN022 | HN047 |  | 843 | 22.37 | 30 | HN034 | HN127 |
| 844 | 21.0398 | 25 | HN022 | HN047 |  | 844 | 26.7265 | 30 | HN034 | HN129 |
| 845 | 22.5145 | 23 | HN022 | HN048 |  | 845 | 22.8118 | 28 | HN034 | HN137 |
| 846 | 22.3783 | 22 | HN022 | HN048 |  | 846 | 23.0285 | 26 | HN034 | HN138 |
| 847 | 25.4412 | 26 | HN022 | HN050 |  | 847 | 26.8479 | 28 | HN034 | HN141 |
| 848 | 27.8655 | 30 | HN022 | HN050 |  | 848 | 24.7071 | 26 | HN034 | HN141 |
| 849 | 26.065 | 28 | HN022 | HN051 |  | 849 | 25.3563 | 27 | HN034 | HN141 |
| 850 | 22.9558 | 28 | HN022 | HN053 |  | 850 | 24.5641 | 24 | HN034 | HN142 |
| 851 | 21.2942 | 25 | HN022 | HN055 |  | 851 | 24.2855 | 27 | HN034 | HN143 |
| 852 | 26.8094 | 32 | HN022 | HN055 |  | 852 | 22.4665 | 26 | HN034 | HN144 |
| 853 | 25.7346 | 29 | HN022 | HN055 |  | 853 | 23.7822 | 25 | HN034 | HN145 |
| 854 | 24.3001 | 22 | HN022 | HN058 |  | 854 | 23.9236 | 27 | HN034 | HN146 |
| 855 | 21.8098 | 25 | HN022 | HN059 |  | 855 | 20.2 | 25 | HN034 | HN147 |
| 856 | 25.3584 | 26 | HN022 | HN059 |  | 856 | 21.9704 | 26 | HN034 | HN147 |
| 857 | 22.3459 | 23 | HN022 | HN059 |  | 857 | 26.6415 | 30 | HN034 | HN148 |
| 858 | 23.3999 | 26 | HN022 | HN060 |  | 858 | 22.1222 | 24 | HN034 | HN150 |
| 859 | 23.4777 | 25 | HN022 | HN060 |  | 859 | 20.899 | 24 | HN035 | HN046 |
| 860 | 21.7481 | 24 | HN022 | HN063 |  | 860 | 20.5782 | 23 | HN035 | HN047 |
| 861 | 23.5371 | 29 | HN022 | HN063 |  | 861 | 23.7301 | 27 | HN035 | HN050 |
| 862 | 23.8831 | 26 | HN022 | HN064 |  | 862 | 19.0728 | 23 | HN035 | HN052 |
| 863 | 24.9457 | 25 | HN022 | HN064 |  | 863 | 22.2931 | 26 | HN035 | HN057 |
| 864 | 27.5555 | 27 | HN022 | HN065 |  | 864 | 21.1279 | 24 | HN035 | HN065 |
| 865 | 25.8799 | 27 | HN022 | HN065 |  | 865 | 21.3547 | 23 | HN035 | HN073 |
| 866 | 27.5515 | 30 | HN022 | HN065 |  | 866 | 19.7599 | 21 | HN035 | HN075 |
| 867 | 25.6023 | 26 | HN022 | HN067 |  | 867 | 26.3592 | 26 | HN035 | HN082 |
| 868 | 24.0529 | 25 | HN022 | HN067 |  | 868 | 25.7529 | 31 | HN035 | HN083 |
| 869 | 23.1049 | 24 | HN022 | HN067 |  | 869 | 19.8929 | 22 | HN035 | HN084 |
| 870 | 26.1422 | 24 | HN022 | HN068 |  | 870 | 22.0168 | 22 | HN035 | HN101 |
| 871 | 27.0986 | 27 | HN022 | HN068 |  | 871 | 22.8497 | 24 | HN035 | HN105 |
| 872 | 27.5702 | 28 | HN022 | HN068 |  | 872 | 20.4672 | 22 | HN035 | HN111 |
| 873 | 25.8871 | 24 | HN022 | HN069 |  | 873 | 27.836 | 24 | HN035 | HN116 |
| 874 | 26.1687 | 27 | HN022 | HN069 |  | 874 | 26.2023 | 25 | HN035 | HN116 |
| 875 | 24.0997 | 24 | HN022 | HN069 |  | 875 | 23.7885 | 21 | HN035 | HN117 |
| 876 | 25.327 | 26 | HN022 | HN069 |  | 876 | 20.7431 | 25 | HN035 | HN124 |
| 877 | 22.0645 | 21 | HN022 | HN070 |  | 877 | 27.1582 | 29 | HN035 | HN125 |
| 878 | 22.646 | 22 | HN022 | HN070 |  | 878 | 20.6238 | 20 | HN035 | HN143 |
| 879 | 21.5367 | 21 | HN022 | HN070 |  | 879 | 19.1881 | 22 | HN036 | HN050 |
| 880 | 24.6876 | 26 | HN022 | HN071 |  | 880 | 21.0015 | 26 | HN036 | HN079 |
| 881 | 25.9894 | 24 | HN022 | HN072 |  | 881 | 18.5397 | 19 | HN036 | HN080 |
| 882 | 27.4444 | 30 | HN022 | HN073 |  | 882 | 26.5982 | 30 | HN036 | HN129 |
| 883 | 18.9993 | 20 | HN022 | HN074 |  | 883 | 19.9493 | 20 | HN036 | HN132 |
| 884 | 21.7765 | 22 | HN022 | HN074 |  | 884 | 20.0267 | 22 | HN037 | HN057 |
| 885 | 23.634 | 22 | HN022 | HN076 |  | 885 | 24.0267 | 28 | HN037 | HN060 |
| 886 | 26.5565 | 26 | HN022 | HN077 |  | 886 | 23.479 | 28 | HN037 | HN060 |
| 887 | 23.7825 | 26 | HN022 | HN079 |  | 887 | 23.1343 | 29 | HN037 | HN060 |
| 888 | 26.0092 | 26 | HN022 | HN079 |  | 888 | 23.7943 | 26 | HN037 | HN064 |
| 889 | 26.9863 | 28 | HN022 | HN080 |  | 889 | 21.5052 | 24 | HN037 | HN067 |
| 890 | 24.9209 | 23 | HN022 | HN080 |  | 890 | 20.8133 | 25 | HN037 | HN072 |
| 891 | 25.607 | 24 | HN022 | HN080 |  | 891 | 27.0963 | 28 | HN037 | HN082 |
| 892 | 25.6289 | 25 | HN022 | HN080 |  | 892 | 22.8599 | 30 | HN037 | HN085 |
| 893 | 24.8663 | 26 | HN022 | HN081 |  | 893 | 20.9487 | 20 | HN037 | HN093 |
| 894 | 25.5121 | 32 | HN022 | HN083 |  | 894 | 18.8111 | 22 | HN037 | HN100 |
| 895 | 27.1756 | 33 | HN022 | HN083 |  | 895 | 23.4455 | 30 | HN037 | HN105 |
| 896 | 23.7455 | 24 | HN022 | HN085 |  | 896 | 23.3997 | 27 | HN037 | HN108 |
| 897 | 27.0851 | 26 | HN022 | HN087 |  | 897 | 23.3997 | 27 | HN037 | HN108 |
| 898 | 23.6226 | 26 | HN022 | HN087 |  | 898 | 22.3905 | 27 | HN037 | HN115 |
| 899 | 23.0935 | 20 | HN022 | HN088 |  | 899 | 23.7943 | 28 | HN037 | HN116 |
| 900 | 26.095 | 25 | HN022 | HN090 |  | 900 | 24.4175 | 24 | HN037 | HN116 |
| 901 | 23.5307 | 24 | HN022 | HN091 |  | 901 | 23.6564 | 27 | HN037 | HN117 |
| 902 | 26.4619 | 30 | HN022 | HN094 |  | 902 | 21.5953 | 28 | HN037 | HN120 |
| 903 | 24.756 | 25 | HN022 | HN094 |  | 903 | 24.1975 | 27 | HN037 | HN123 |
| 904 | 24.6349 | 25 | HN022 | HN095 |  | 904 | 23.2941 | 29 | HN037 | HN127 |
| 905 | 25.4639 | 27 | HN022 | HN096 |  | 905 | 20.4127 | 26 | HN037 | HN128 |
| 906 | 26.8674 | 28 | HN022 | HN096 |  | 906 | 20.1405 | 23 | HN037 | HN136 |
| 907 | 25.9361 | 25 | HN022 | HN097 |  | 907 | 19.6371 | 26 | HN037 | HN141 |
| 908 | 23.8532 | 23 | HN022 | HN100 |  | 908 | 22.8141 | 28 | HN037 | HN143 |
| 909 | 23.8779 | 22 | HN022 | HN102 |  | 909 | 19.3517 | 25 | HN037 | HN146 |
| 910 | 27.2811 | 24 | HN022 | HN104 |  | 910 | 22.8974 | 21 | HN038 | HN043 |
| 911 | 21.6744 | 20 | HN022 | HN104 |  | 911 | 18.9461 | 19 | HN038 | HN048 |
| 912 | 21.5301 | 28 | HN022 | HN105 |  | 912 | 20.8442 | 27 | HN038 | HN050 |
| 913 | 25.786 | 19 | HN022 | HN106 |  | 913 | 23.2357 | 27 | HN038 | HN050 |
| 914 | 22.09 | 23 | HN022 | HN108 |  | 914 | 23.2357 | 27 | HN038 | HN050 |
| 915 | 23.5438 | 24 | HN022 | HN108 |  | 915 | 17.799 | 24 | HN038 | HN054 |
| 916 | 23.2399 | 24 | HN022 | HN109 |  | 916 | 19.546 | 23 | HN038 | HN061 |
| 917 | 26.8525 | 29 | HN022 | HN109 |  | 917 | 23.3059 | 25 | HN038 | HN064 |
| 918 | 26.9431 | 26 | HN022 | HN109 |  | 918 | 19.5063 | 22 | HN038 | HN068 |
| 919 | 23.2557 | 22 | HN022 | HN110 |  | 919 | 24.3804 | 26 | HN038 | HN082 |
| 920 | 28.5185 | 27 | HN022 | HN111 |  | 920 | 25.159 | 30 | HN038 | HN094 |
| 921 | 23.0817 | 24 | HN022 | HN111 |  | 921 | 18.9885 | 23 | HN038 | HN102 |
| 922 | 25.9303 | 23 | HN022 | HN111 |  | 922 | 17.6318 | 21 | HN038 | HN104 |
| 923 | 22.0244 | 26 | HN022 | HN112 |  | 923 | 20.0335 | 23 | HN038 | HN105 |
| 924 | 22.1593 | 26 | HN022 | HN112 |  | 924 | 24.2395 | 30 | HN038 | HN105 |
| 925 | 27.561 | 30 | HN022 | HN113 |  | 925 | 18.3456 | 22 | HN038 | HN115 |
| 926 | 28.6106 | 27 | HN022 | HN113 |  | 926 | 22.5333 | 28 | HN038 | HN130 |
| 927 | 29.5532 | 28 | HN022 | HN113 |  | 927 | 24.8766 | 28 | HN039 | HN043 |
| 928 | 26.8294 | 23 | HN022 | HN113 |  | 928 | 19.8628 | 21 | HN039 | HN043 |
| 929 | 26.3578 | 25 | HN022 | HN114 |  | 929 | 22.1187 | 24 | HN039 | HN048 |
| 930 | 27.0057 | 28 | HN022 | HN115 |  | 930 | 24.9678 | 29 | HN039 | HN050 |
| 931 | 24.6467 | 27 | HN022 | HN116 |  | 931 | 22.9849 | 25 | HN039 | HN065 |
| 932 | 27.4794 | 27 | HN022 | HN116 |  | 932 | 20.8765 | 25 | HN039 | HN068 |
| 933 | 24.4075 | 25 | HN022 | HN116 |  | 933 | 25.0485 | 31 | HN039 | HN073 |
| 934 | 27.2926 | 27 | HN022 | HN116 |  | 934 | 15.6567 | 25 | HN039 | HN079 |
[truncated: 162,294 more chars]
